# Heterarchy of transcription factors driving basal and luminal cell phenotypes in human urothelium

**DOI:** 10.1038/cdd.2017.10

**Published:** 2017-03-10

**Authors:** Carl Fishwick, Janet Higgins, Lawrence Percival-Alwyn, Arianna Hustler, Joanna Pearson, Sarah Bastkowski, Simon Moxon, David Swarbreck, Chris D Greenman, Jennifer Southgate

**Affiliations:** 1 Jack Birch Unit for Molecular Carcinogenesis, Department of Biology, University of York, York YO10 5DD, UK; 2 Earlham Institute, Norwich Research Park, Norwich NR4 7UZ, UK; 3 School of Computing Sciences, University of East Anglia, Norwich NR4 7TJ, UK

## Abstract

Cell differentiation is affected by complex networks of transcription factors that co-ordinate re-organisation of the chromatin landscape. The hierarchies of these relationships can be difficult to dissect. During *in vitro* differentiation of normal human uro-epithelial cells, formaldehyde-assisted isolation of regulatory elements (FAIRE-seq) and RNA-seq was used to identify alterations in chromatin accessibility and gene expression changes following activation of the nuclear receptor peroxisome proliferator-activated receptor gamma (PPARγ) as a differentiation-initiating event. Regions of chromatin identified by FAIRE-seq, as having altered accessibility during differentiation, were found to be enriched with sequence-specific binding motifs for transcription factors predicted to be involved in driving basal and differentiated urothelial cell phenotypes, including forkhead box A1 (FOXA1), P63, GRHL2, CTCF and GATA-binding protein 3 (GATA3). In addition, co-occurrence of GATA3 motifs was observed within subsets of differentiation-specific peaks containing P63 or FOXA1. Changes in abundance of GRHL2, GATA3 and P63 were observed in immunoblots of chromatin-enriched extracts. Transient siRNA knockdown of P63 revealed that P63 favoured a basal-like phenotype by inhibiting differentiation and promoting expression of basal marker genes. GATA3 siRNA prevented differentiation-associated downregulation of P63 protein and transcript, and demonstrated positive feedback of GATA3 on *PPARG* transcript, but showed no effect on FOXA1 transcript or protein expression. This approach indicates that as a transcriptionally regulated programme, urothelial differentiation operates as a heterarchy, wherein GATA3 is able to co-operate with FOXA1 to drive expression of luminal marker genes, but that P63 has potential to transrepress expression of the same genes.

The nuclear receptor peroxisome proliferator-activated receptor gamma (PPARγ) is widely known as an essential and sufficient driver of adipogenesis,^1, 2^ but it also plays roles in M1 to M2 polarisation of macrophages^3^ and differentiation of human urothelial cells of the bladder and associated urinary tract.^4, 5, 6^ When grown *in vitro* in the absence of serum or other nuclear receptor signalling, non-immortalised normal human urothelial (NHU) cells acquire a proliferative, autocrine epidermal growth factor receptor (EGFR)-regulated squamous cell phenotype.^7, 8^ RNA microarray studies of NHU cell cultures have shown that when downstream EGFR signalling is blocked, exogenous ligand activation of PPARγ induces expression of intermediary transcription factors required for specifying the differentiated urothelial cell phenotype, including forkhead box A1 (FOXA1), interferon regulatory factor 1 (IRF1), GATA-binding protein 3 (GATA3) and E74-like ETS transcription factor 3 (ELF3).^9, 10^ Of these, FOXA1 and GATA3 are recognised as pioneer factors capable of driving changes in chromatin organisation and accessibility.^11^ In urothelial carcinoma, *FOXA1* and *GATA3* have been associated with differentiation status^12, 13^ and 8% of tumours were found to carry *ELF3* mutations.^14^ Mouse studies have identified other transcription factors as determinants of urothelial specification, including Grainyhead-like transcription factor 3 (Grhl3),^15^ Kruppel-like factor (Klf5),^16^ and Gata4 and Gata6,^17^ but it remains unclear what role these factors play in human urothelium.

Formaldehyde-assisted isolation of regulatory elements coupled with next-generation sequencing (FAIRE-seq)^18^ exploits the propensity of nucleosome-depleted DNA, or ‘open' chromatin, to shear from adjacent nucleosomes during sonication of nuclear material from formaldehyde-fixed cells. Isolating this sheared DNA from nucleosomal DNA by phase separation enables characterisation of the relative extent of chromatin accessibility in a genome-wide manner. As transcription factors bind dynamically to nucleosome-depleted regions, motif matching within open chromatin, as identified by FAIRE, can be used to classify transcription factors that actively associate with chromatin and define cell phenotype.^19, 20, 21, 22, 23^ FAIRE identifies a complementary but partially distinct set of putative enhancer regions outside of gene promoters, as compared to DNase-seq,^19^ which uses DNase I enzyme to cleave regions of open chromatin. FAIRE-seq DNA has been shown to be enriched relative to DNase-seq for potential FOXA1-binding sites, which are known to contribute to urothelial differentiation,^9^ and chromatin-associated histone H3 monomethylated on lysine 4 (H3K4me1), which is associated with genomic enhancers specific to cell type.

To obtain a genome-wide picture of the transcriptional drivers of different urothelial cell phenotypes, RNA-seq and FAIRE-seq were performed on serially propagated NHU cell cultures from three independent donors at 24 and 144 h time points after concurrent EGFR blockade and PPARγ activation to induce differentiation,^4^ alongside time-matched non-differentiated vehicle controls. Open chromatin regions unique to treated and control libraries were searched for matches to known sequence-specific transcription factor-binding motifs, both on a genome-wide basis and proximal to differentially expressed genes. Selected candidate transcriptional regulators were validated as modulators of urothelial differentiation using immunoblots of chromatin-enriched extracts and siRNA knockdown to investigate effects on urothelial phenotype.

## Results

### Differentially expressed genes and FAIRE-seq peak genomic distribution

Results obtained from the analysis of RNA-seq data identified 559 and 463 genes that were upregulated, and 467 and 158 genes that were downregulated in differentiation-induced cells relative to time-matched controls at the 24 and 144 h time points (false discovery rate (FDR)<0.1), respectively (Supplementary Figure S1, Supplementary Tables 1A and 1B). Transcripts upregulated at both time points included the urothelium-restricted differentiation markers uroplakin 1A (*UPK1A*) and *UPK2*.^24, 25, 26^ Gene ontology analysis, performed using the GOrilla tool,^27^ showed that the 122 genes upregulated at both time points included genes involved in lipid metabolism (*P*=1.16 × 10^−5^) and water homeostasis (*P*=8.09 × 10 ^× 5^; Supplementary Table 2), with the latter likely reflecting the role of urothelium as a barrier to urinary solutes.

**Figure 1:**
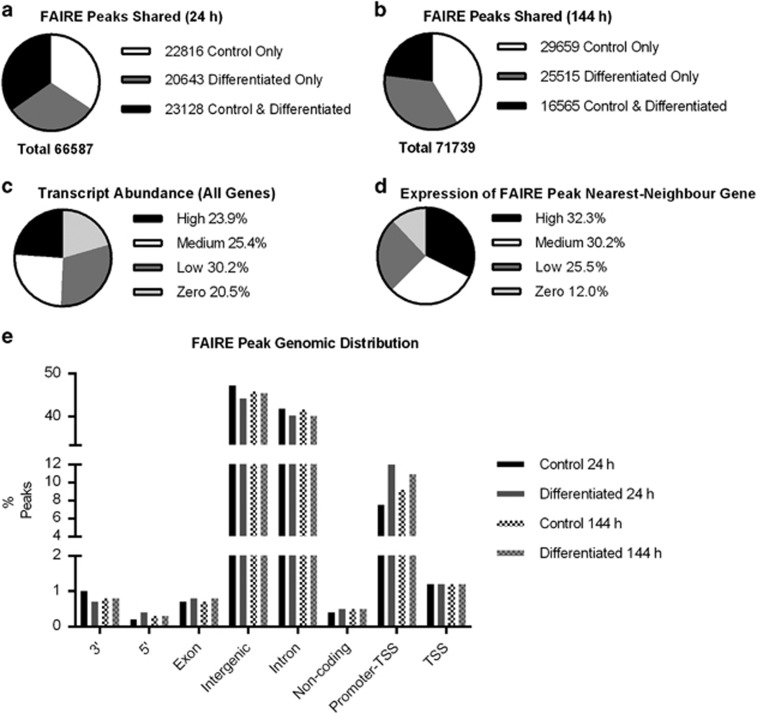
(**a** and **b**) Numbers of overlapping FAIRE peaks between control and differentiation-induced cells at 24 and 144 h. (**c**) Genes for the 24 h control sample were split into quartiles based on reads per kilobase per mapped million (RPKM) in RNA-seq data, high RPKM ≥10, medium RPKM ≥1<10, low RPKM >0<1, zero RPKM=0. (**d**) FAIRE peaks were labelled based on the high, medium or zero expression of the nearest-neighbour gene. FAIRE peaks were in this case more often near genes with expression above zero. Data representative of all time points. (**e**) Position of FAIRE peaks relative to annotated genomic features demonstrated that the majority of peaks were intronic or intergenic. The greatest variation between samples was seen within the proportion of peaks at promoters directly upstream of a transcription start site, with increases in the proportion of FAIRE peaks at these sites in both differentiated time points relative to their non-differentiated controls

Peak calling using the MACS algorithm on FAIRE-seq data pooled for the three donor cell lines gave >66 000 total peaks rising to >71 000 at 144 h, with a near equal distribution between proportions of distinct (control or differentiated) and overlapping peaks at each time point (Figures 1a and b). Consistent with other investigations into the relationship between DNA enriched by FAIRE and gene expression,^19, 20^ when genes were split into quartiles based on normalised RNA-seq read counts (Figure 1c and Supplementary Table 3), a greater proportion of nearest-neighbour genes to FAIRE peaks had reads per kilobase per mapped million (RPKM) values above zero as compared to total genes (Figure 1d). In addition, most FAIRE peaks were intronic or intergenic, and a slight increase in the proportion of peaks associated with promoters was noted in differentiation-induced cells at both time points (Figure 1e and Supplementary Table 3).

### Transcription factor motifs enriched in FAIRE peaks

To uncover transcription factors driving cell phenotype in differentiated and non-differentiated urothelial cells, sequence-specific transcription factor-binding motifs enriched in non-overlapping FAIRE peaks at each time point were identified using the motif discovery tool HOMER.^28^ Motif searching was conducted using control-specific peak sets as the background for the differentiation-specific peak set and vice versa.

**Figure 2:**
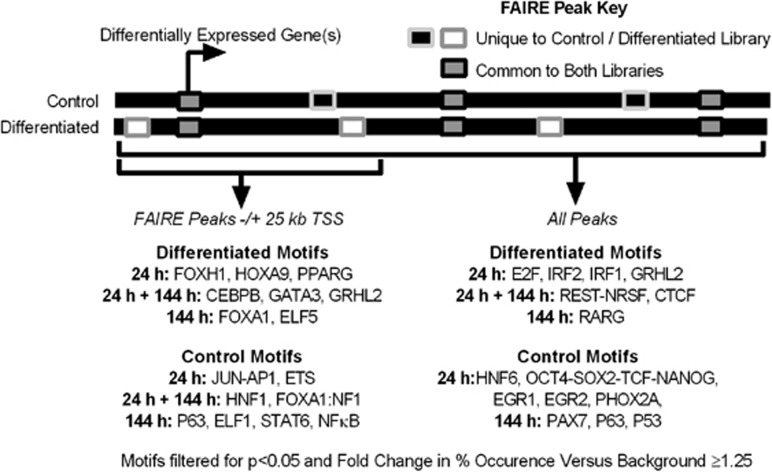
Summary of known motifs from the HOMER database matched in FAIRE-seq peaks specific to control and differentiation-induced NHU cells. FAIRE-seq peaks from pooled donor data were compared between control and differentiation-induced cells at 24 and 144 h time points, and peaks unique (non-overlapping) to each library were searched for known sequence motifs in HOMER to generate a genome-wide comparison for all peaks. The same comparison was performed using only peaks found within ±25 kb of the TSS of genes upregulated or downregulated during differentiation at the respective time points

Previous transcription factor motif matching studies using open chromatin isolation techniques have observed that particular motifs tend to be enriched at sites distal to genes,^29^ and that within promoter regions, transcription start sites (TSSs) have fewer differences in transcription factor motifs than the rest of the genome.^20^ As such, FAIRE peaks in TSS promoter regions (−1 kb to +100 bp) were excluded from all analyses. To highlight any differences between motifs enriched proximal to genes and those found across the genome, control-specific and differentiation-specific FAIRE peaks were compared as either genome-wide groups, or analysis was restricted to those located within ±25 kb of the TSS of differentially regulated genes. Motifs matched by HOMER were filtered for those which occurred in at least ≥1.25-fold of the total percentage of regions in the target set as compared to the background set, in order to focus on motifs significantly enriched in each experimental situation.^20, 30^ This approach identified divergent groups of transcription factor motifs across the different regions, with each group containing matches to motifs from both previously described urothelium-associated factors and others not previously associated with urothelium (Figure 2 and Supplementary Tables 4–12). *De novo* motif analysis was less successful than matching to known motifs, as most matches that were not similar to those found in the HOMER database were in low percentages of peaks (data not shown).

Motifs with the highest fold change in abundance in peaks specific to control libraries and around downregulated genes at 24 h were dominated by cell cycle-associated transcription factors such as ETS family factors, JUN-AP1, EGR1 and EGR2, and a motif associated with combined binding of the OCT4-SOX2-TCF-NANOG pluripotency factors in mouse embryonic stem cells.^31^ *OCT4* transcripts are expressed by NHU cells, but the pluripotency-associated isoform OCT4A is not.^32^ P63, a transcription factor associated with a non-differentiated ‘basal-like' urothelial cell phenotype in normal cells and carcinoma,^33, 34, 35, 36, 37^ was enriched both proximal to downregulated genes and across the genome at 144 h, whereas STAT6 and ETS motifs were specifically associated with peaks ±25 kb of downregulated genes at this time point.

Motifs from urothelial differentiation-associated transcription factors FOXA1,^9^ GATA3^10, 12^ and PPARγ^4^ were enriched in differentiation-specific FAIRE peaks within ±25 kb of the TSS of genes with expression upregulated during differentiation. PPARγ motifs were only enriched around genes upregulated at 24 h, in agreement with observations that it drives early events during *in vitro* urothelial differentiation upstream of FOXA1,^9^ motifs from which were matched at 144 h. GATA3, CEBPB and GRHL2 motifs were enriched around upregulated genes at both time points. GRHL2 has been implicated in regulation of tight junction complex genes, which are central to barrier formation in several epithelia,^38^ including urothelium,^6, 39^ whereas the closely related GRHL3 has been associated with urothelial differentiation in the mouse.^15^ CEBPB plays a key role in orchestrating *CEBPA* and *PPARG* expression during adipogenesis.^2^ CEBPB has no known role in normal human urothelial biology, although other groups have shown the CEBPB motif to be enriched in promoters of urothelial carcinoma gene sets,^40^ and it has been associated with urothelial differentiation in mouse.^41^ ELF5 and ELF1 motifs were enriched in regions proximal to up- and downregulated genes at 144 h, respectively. Although neither of these has been previously associated with urothelial biology, the closely related ELF3, whose motif is not in the HOMER database used here, is a driver of differentiation.^10^

Across the genome, in differentiation-induced cells, motifs from the known urothelium-associated transcription factor IRF1^9^ and the closely related motif for IRF2 were enriched at 24 h, as were those from CTCF at both time points. As none of these motifs were enriched proximal to differentially regulated genes, these observations agree with previous studies that showed CTCF and IRF1 preferentially bind to regions distal to expressed genes.^29^

### Co-occurrence of transcription factor motifs in open chromatin

Lineage-determining transcription factors have been observed to bind in regions proximal to one another during differentiation.^28^ Pioneer factors such as FOXA1, which can open repressed regions of chromatin, often bind proximally to differentiation-inducing nuclear receptors.^42, 43, 44^ To determine whether there was co-occurrence of differentiation-associated transcription factor motifs within FAIRE-seq peaks, P63 and FOXA1 motif-containing open chromatin regions specific to control and differentiated cells at each time point were searched separately for enriched motifs using the same approach as for the genome-wide investigation. P63- and FOXA1-containing peaks were enriched with motifs that overlapped the genome-wide set of peaks, but with significant differences (Supplementary Figures 2A, 2B and Supplementary Tables 13–20).

Motifs co-occurring within P63- and FOXA1-containing peaks were largely distinct from one another, but with notable exceptions such as GATA3, GRHL2, P63 and IRF motifs, which co-occurred with both FOXA1 and P63 in differentiation-specific peaks (Supplementary Figure 3).

### Chromatin binding of transcription factors with enriched motifs

**Figure 3:**
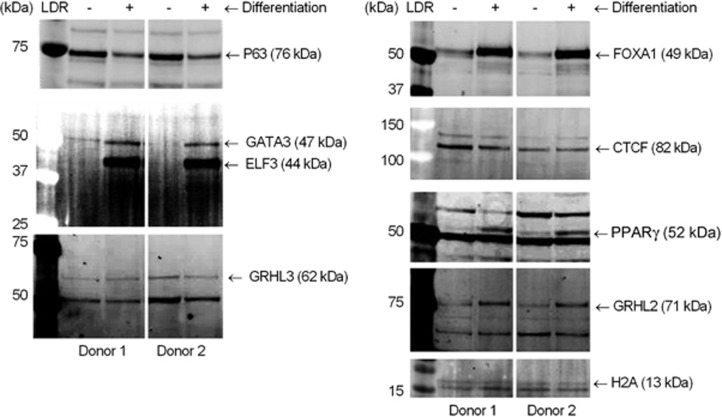
Chromatin extracts showing bound transcription factors that changed in abundance during differentiation. Factors with motifs detected as enriched in differentiation-specific FAIRE peaks, including GRHL2, GATA3, FOXA1 and PPARγ, were upregulated in chromatin extracts from differentiation-induced NHU cells from two independent donors. CTCF and GRHL3 did not change in abundance with differentiation. P63 abundance was reduced after induction of differentiation. Histone H2A is included as a loading control

To determine whether transcription factors with enriched motifs and other putative urothelial phenotype orchestrators reported in the literature were enriched in urothelial chromatin, immunoblots of chromatin extracts were generated using urothelial cell cultures from independent lines. PPARγ, FOXA1, GRHL2 and GATA3 were enriched in chromatin extracted from differentiated cell cultures, whereas basal-associated P63 was more abundant in non-differentiated cultures (Figure 3). CTCF and GRHL3 had similar abundance on chromatin from control and differentiated cultures. ELF5 and ELF1 detection was not possible due to poor antibody specificity, but ELF3 was observed to be associated with chromatin from differentiated cells.

### Differentiation-associated transcription factors in native urothelium

**Figure 4:**
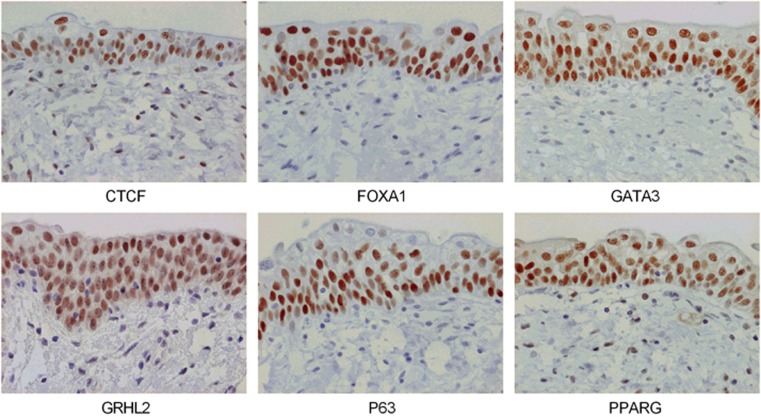
Native human urothelium showed nuclear localisation of differentiation-associated transcription factors CTCF, FOXA1, GATA3, GRHL2 and PPARγ in all stratified layers. P63 was observed predominantly in basal and intermediate cells. Occasional cells in the urothelium with condensed nuclei that do not label for most transcription factors are consistent, morphologically, with infiltrating lymphocytes

To determine whether transcription factors with motifs matched to the non-differentiated or differentiated NHU cell phenotypes were expressed by normal urothelium *in situ*, immunohistochemistry was performed on human urothelial tissue sections (Figure 4). P63 demonstrated a basal–intermediate cell distribution, with markedly reduced labelling of the most differentiated superficial cells. PPARγ, CTCF, GATA3, GRHL2 and FOXA1 were observed to be nuclear in all layers of the urothelium, with GRHL2 and FOXA1 showing particularly intense labelling of the most differentiated superficial cell layer.

### siRNA knockdown of P63 and GATA3

**Figure 5:**
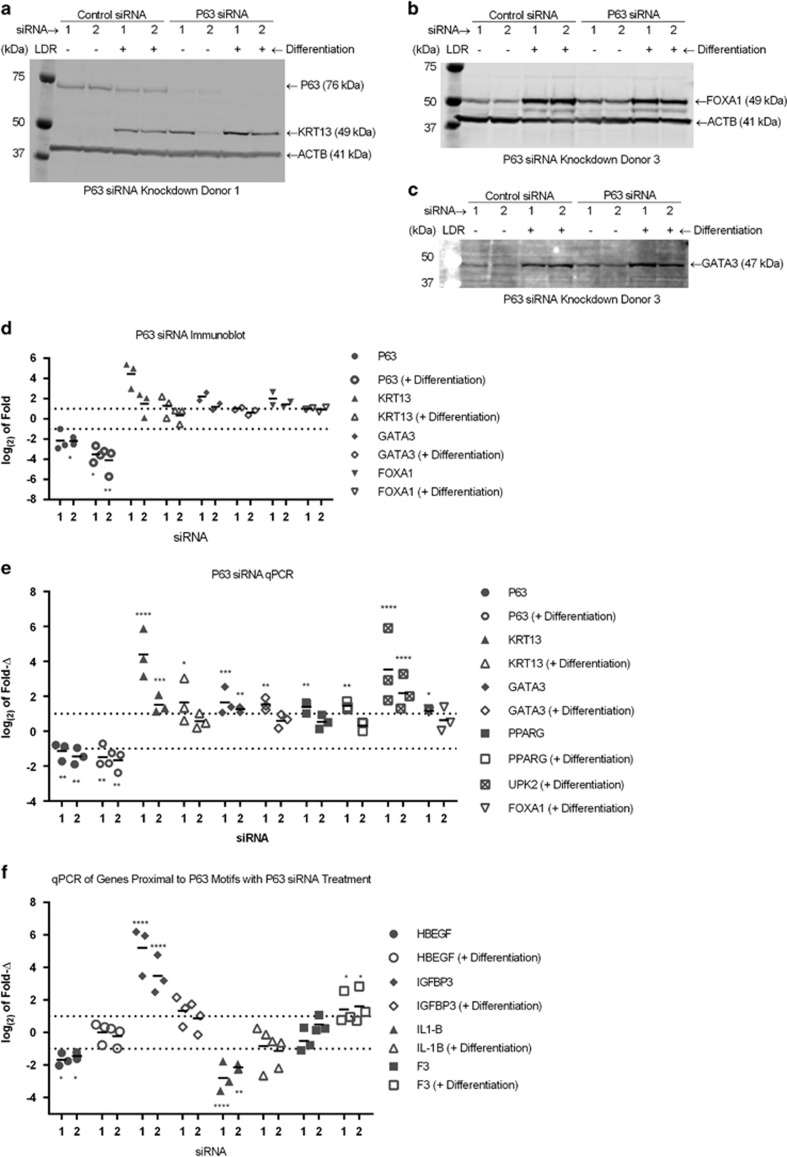
Immunoblot of whole-cell lysates from representative NHU cell donors showing effect of P63 siRNA on (**a**) P63 and KRT13, (**b**) FOXA1 and (**c**) GATA3 protein expression, with (+) and without (−) differentiation induction at 48 h. ACTB=β actin loading control. FOXA1 and GATA3 were on the same membrane and normalised to the ACTB shown with FOXA1. (**d**) Densitometry measurements from immunoblots showing log_(2)_ fold change of intensity in immunoblotting for three independent donors for P63 and KRT13, and two independent donors for GATA3 and FOXA1 following P63 siRNA, relative to control siRNA. Statistical test performed where material from three donors was measured was a Repeated Measures one-way ANOVA with Greenhous–Geisser correction and Sidak's multiple comparison post test, with *P*-values indicated by *P≤0.05, **P≤0.01, ***P≤0.001 and ****P<0.0001. (**e** and **f**) RT-qPCR results from NHU cells from three independent donors showing change in abundance of RNA transcript after exposure to P63 siRNA either with or without induction of differentiation for 48 h for (**e**) urothelial differentiation-associated, and (**f**) genes associated with P63 motif-containing FAIRE peaks. Log_(2)_ fold change measured relative to control siRNA with or without differentiation induction. All qPCR transcript relative abundance measurements were normalised internally to GAPDH. Statistics was performed using a two-way ANOVA with Dunnett's multiple comparison post test, with *P*-values indicated by *P≤0.05, **P≤0.01, ***P≤0.001 and ****P<0.0001

To further ascertain whether chromatin-associated proteins identified by FAIRE played a role in the differentiation of urothelial cells, the effects of siRNA knockdown of *P63* and *GATA3* on the expression of urothelial differentiation markers were investigated 48 h after transfection with siRNA in conjunction with differentiation or control treatment in independent NHU cell lines. In non-differentiated cells, expression of P63 protein was reduced ≥2-fold in all donors following P63 siRNA knockdown, and was reduced further following induction of differentiation (Figures 5a, d and Supplementary Figure 4). Expression of cytokeratin 13 (KRT13), which is expressed by basal and intermediate cell layers of normal human urothelium *in situ* and provides an objective marker of the switch from the basal-like squamous to a urothelial transitional epithelial differentiation programme,^5^ was increased following knockdown of P63 (siRNA 1) in all donors in both non-differentiated and PPARγ-activated conditions, although statistical significance was not reached due to a large variation in the fold increase between different donor cell lines (Figures 5a, d and supplementary Figure 4). GATA3 and FOXA1 protein (immunoblotted in two NHU cell lines) increased ~2-fold in cells treated with P63 siRNA in both non-differentiated and differentiated states (Figures 5b–d and supplementary Figure 4).

At the transcript level, P63 siRNA stimulated the expression of *KRT13* and transcription factors *PPARG* and *GATA3* in non-differentiated cells, and further increased the expression of *KRT13, PPARG, GATA3, FOXA1* and *UPK2* transcripts following induction of differentiation (Figure 5e).

P63 is a key driver of genes associated with basal-type urothelial carcinomas.^33, 36, 37, 45^ To further investigate these links, lists of genes proximal to P63-containing motifs at the 24 h time point that overlapped genes observed to be differentially regulated in a P63 knockdown model in urothelial carcinoma cell lines^36^ were compared (Supplementary Tables 21–25). Of the genes that overlapped between the P63-containing FAIRE peaks and P63 knockdown in carcinoma-derived cell lines, *F3, HBEGF, IGFBP3* and *IL1B* were further investigated by RT-qPCR in P63 siRNA-treated NHU cells. In RNA-seq data from differentiation at 24 h, *F3* and *HBEGF* were significantly downregulated, whereas *IGFBP3* was upregulated (Supplementary Table 1A). Only *IGFBP3* was significantly upregulated at 144 h (Supplementary Table 1B). P63 siRNA downregulated *HBEGF* and *IL1B* in the absence of differentiation-inducing signals, but this effect was not observed in differentiation-induced cells for either gene (Figure 5f). *IGFBP3* was strikingly upregulated in P63 siRNA-treated cells without differentiation, but only marginally upregulated in P63 siRNA cells induced to differentiate. *F3* expression was not significantly altered by P63 siRNA in undifferentiated cells, but had weakly significantly increased expression when cells were differentiated in the presence of P63 siRNA.

**Figure 6:**
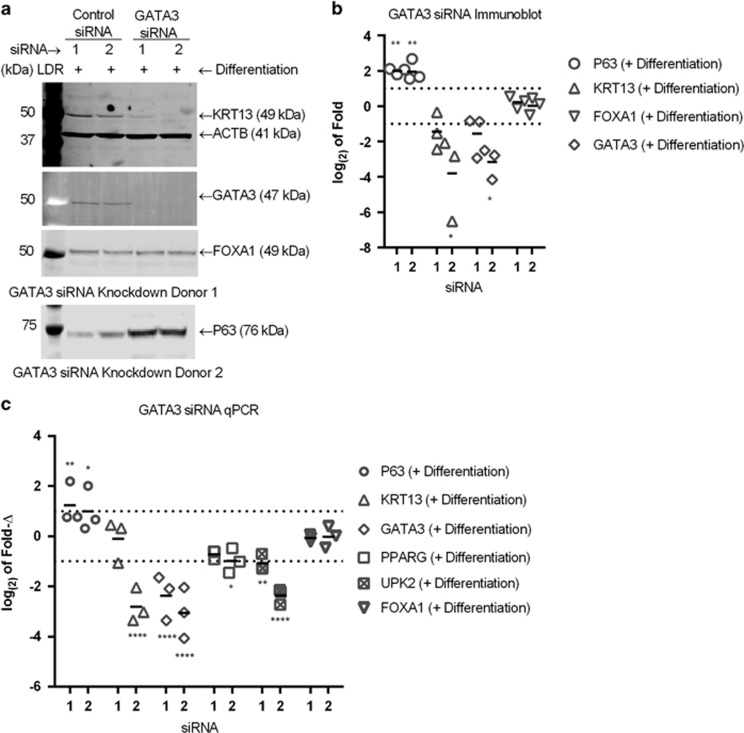
(**a**) Representative immunoblots of NHU whole-cell lysate showing GATA3, KRT13, FOXA1 and P63 protein expression after differentiation induction for 48 h following transfection with GATA3 siRNA. ACTB=β actin loading control. KRT13, FOXA1 and GATA3 were on the same membrane and normalised to the ACTB shown, and the P63 was on a separate membrane and normalised to a separate ACTB as shown in the supplementary data. (**b**) Densitometry measurements from immunoblots of three donors showing log_(2)_ fold change in the expression of GATA3, KRT13 and FOXA1 in 48 h differentiation-induced NHU cells following transfection with GATA3 siRNA relative to control siRNA. Signals for P63 and KRT13 were normalised for loading to β-actin (ACTB) and fold change determined relative to the equivalent control siRNA transfection results. Statistical test performed was a repeated measures one-way ANOVA with Greenhous-Geisser correction and Sidak's multiple comparison post test, with *P*-values indicated by *P≤0.05, **P≤0.01, ***P≤0.001 and ****P<0.0001. (**c**) RT-qPCR results combined from NHU cells from three independent donors showing change in abundance of RNA transcript for P63 and differentiation-associated genes after transfection with GATA3 siRNA followed by differentiation for 48 h. Log_(2)_ fold change shown relative to control siRNA transfection with followed by 48 h differentiation. Statistics was performed using a two-way ANOVA with Dunnett's multiple comparison post test, with *P*-values indicated by *P≤0.05, **P≤0.01, ***P≤0.001 and ****P<0.0001

GATA3 siRNA achieved a 1.7–7.6-fold reduction in GATA3 protein expression in differentiation-induced NHU cells, with GATA3 siRNA 2 effectively abrogating the differentiation-induced increase in KRT13 protein expression (Figures 6a, b and Supplementary Figure 5). P63 protein expression was significantly upregulated in the presence of GATA3 siRNA, whereas FOXA1 protein expression was not affected.

GATA3 siRNA significantly attenuated transcript expression of *GATA3* and the differentiation marker *UPK2* (Figure 6c). *KRT13* transcript was only reduced significantly by GATA3 siRNA 2, as with the protein. *P63* showed increases in transcript and protein expression with both GATA3 siRNA oligonucleotides. Neither GATA3 siRNA sequence had an effect on *FOXA1* transcript abundance and only siRNA 2 showed a small inhibitory effect on *PPARG* transcript expression

## Discussion

By comparing transcription factor-binding motifs matched within open chromatin regions in normal human urothelial cells in non-differentiated versus differentiated states, this study provides new insight into the identity and operational relationships between transcriptional drivers of urothelial cell phenotype. Of major significance, P63 drives the non-differentiated squamous phenotype assumed by normal human urothelial cells maintained in serum-free culture conditions in the absence of nuclear receptor signalling. siRNA knockdown revealed that P63 maintains this primitive or ‘basal-like' phenotype at least in part by inhibiting expression of transitional epithelial lineage genes including *KRT13* and *PPARG*.

P63 plays an essential role in epithelial tissues during development, where its absence causes severe dysgenesis of epithelial tissues, as described in mouse epidermis.^46^ Changes in expression and somatic mutations of P63 have been associated with clinically relevant subtypes of bladder cancer, with P63 identified as a driver of the basal-like cell phenotype in urothelial carcinoma.^36^ These authors showed that knockdown of P63 in the established bladder cancer-derived UM-UC14 cell line affected expression of *PPARG*-influenced genes, including *HBEGF*, *IGFBP3* and *IL1B*.^36^ Here these same genes were differentially affected by siRNA knockdown of P63 in NHU cells, implying usage of the same gene networks by normal and cancer cells.

In urothelium, PPARγ has been identified as a nuclear receptor whose activation mediates the transition from squamous to a differentiated transitional (urothelial) phenotype. This involves a major shift in gene expression, implying a change in genomic organisation to reflect the transcriptional landscape of urothelium. We have previously identified a network of PPARγ-regulated intermediary transcription factors that mediate the differentiated urothelial programme, although inter-relationships within the network have yet to be established. In other tissues, such as breast, a role has been identified for the so-called pioneer factors FOXA and GATA in defining the tissue-specific genomic organisation. GATA3 and FOXA1 have been shown to act co-operatively in promoting ESR1-driven transcription in MCF7 cells, with GATA3 lying upstream of FOXA1.^44^ In the current study, GATA3 siRNA in combination with PPARγ stimulation prevented downregulation of P63 and attenuated expression of intermediate to late differentiation markers, but did not alter FOXA1 expression. As FOXA1, P63 and GATA3 motifs were all co-enriched within the same open chromatin associated specifically with differentiation, this establishes a basis for a model of the interaction of all three factors in determining urothelial phenotype wherein P63 outcompetes FOXA1 for chromatin-binding sites in the absence of GATA3. The results from modulating GATA3 expression point to the existence of a heterarchical relationship between differentiation drivers, in which transcription factors such as GATA3 are capable of influencing the expression of phenotypic drivers such as P63 independently of other key determining intermediary transcription factors in the network, including FOXA1.

The motif matching performed here identified transcription factors not previously associated with urothelial differentiation, including CTCF. CTCF was not enriched at the protein level in chromatin extracts after induction of differentiation, most probably because CTCF is a constitutive chromatin-associated protein that, among other functions, facilitates looping of chromatin.^47, 48, 49, 50, 51^ The results in this study add to the weight of evidence that CTCF binding, although widespread and well-conserved in many genomic regions,^47, 48, 49, 50, 51^ shows tissue-specific binding around genes associated with cell phenotype.

Our initial analysis of differentially expressed gene transcripts indicated a potential role for *GRHL3* in differentiation of human urothelium. However, no differentiation-associated changes in GRHL3 protein abundance or localisation were seen during differentiation, whereas the constitutively expressed *GRHL2* gene showed increased protein abundance and relocation onto the chromatin of differentiating cells. Taken with the nuclear localisation of GRHL2 *in situ*, these data implicate GRHL2 as the more important factor and further illustrate that not all differentiation-associated events are transcriptionally regulated. GRHL2 has been observed to be expressed by human urothelium in another recent study^52^ and is known to reside within a genomic region that is commonly amplified in aggressive urothelial carcinoma.^53^

Klf5 is reported to be upstream of Pparg and Grhl3 in mouse urothelial development,^16^ however KLF5 was not significantly differentially expressed in urothelium as determined by RNA-seq, nor were KLF5 motifs enriched in FAIRE peaks analysed herein. This suggests if KLF5 has a role in human urothelial development, it may function in early urothelial specification and not be directly associated with regulating genes associated with mature differentiation stages. Gata4 has been associated with urothelial differentiation in mouse.^17^ However, *GATA4* was not detected in RNA-seq data in the current study, where *GATA3* transcript was the most highly expressed GATA gene family member detected and, in addition, was the only GATA member to be upregulated upon differentiation and associated with post-differentiation chromatin. These data implicate GATA3 rather than GATA4 in the differentiation of human urothelium.

## Materials and methods

### *In vitro* growth and differentiation of normal human urothelial cells

NHU cells were maintained as finite, serially passaged cell lines, as described previously.^54^ Cultures were subcultured by trypsinisation and maintained in keratinocyte serum-free medium containing bovine pituitary extract and epidermal growth factor (Gibco, Paisley, UK) and further supplemented with 30 ng/ml cholera toxin (Sigma-Aldrich, Dorset, UK). Differentiation was induced in just-confluent cell cultures using 1 *μ*M troglitazone as PPARγ ligand with concurrent 1 *μ*M PD153035 to block epidermal growth factor receptor activation.^4^ Non-differentiated vehicle control (0.1% DMSO) cultures were maintained in parallel and used at the same time points (24 and 144 h).

### RNA-seq sample and library preparation

Cell monolayers were solubilised in Trizol (Invitrogen, Paisley, UK), using the manufacturer's protocol for chloroform and isopropanol extraction, and DNA was digested using RNAse-free DNase I (Ambion, Foster City, CA, USA). Library construction was performed using TruSeq RNA Sample Prep Kit v2 (Illumina United Kingdom, Great Chesterford, UK). Sequencing was performed using an Illumina HiSeq 2500 sequencer and reads aligned using RSEM^55^ to the reference UCSC hg19 human genome. Differential gene expression was performed between control and differentiation-induced cells at 24 and 144 h time points using DESeq.^56^ The results obtained from three independent cell lines were treated as replicates and genes with a FDR cutoff <0.1 were called significant.

### FAIRE-seq sample and library preparation

Cell monolayers were fixed in 1% formaldehyde for 10 min before quenching by addition of glycine to 125 mM for 5 min and scrape-harvesting in ice-cold PBS with added protease inhibitors. Approximately 5 × 10^6^ cells were lysed and sheared, and open chromatin extracted as described in the FAIRE protocol.^57^

### Motif searching

MACS peak-calling algorithm^58^ was used to call FAIRE-enriched peaks. Non-overlapping peaks between control and differentiated samples at each time point were identified using bedtools. HOMER motif discovery software^28^ was used to discover motifs over-represented in each treatment condition, using peaks uniquely present in control cells as the background when searching the differentiation-induced specific peaks, and vice versa. Motifs identified by HOMER as enriched were further filtered by fold change as percentage enrichment above background of ≥1.25.

### Chromatin enrichment

Cells were fixed and scrape-harvested as for FAIRE, then pelleted cells were subjected to a chromatin enrichment protocol^59^ with optional RNase digestion step included.

### Antibodies

Anti-FOXA1 (Santa Cruz Biotechnology, Santa Cruz, CA, USA, catalogue no. sc-101058) used at 1:250 for IHC and 1:400 for immunoblot. Anti-CTCF (Cell Signaling, Beverley, MA, USA, catalogue no. 2899) used at 1:250 for IHC and 1:1000 for immunoblot. Anti-P63 (Santa Cruz Biotechnologies, catalogue no. sc-8431) used at 1:1000 for IHC and 1:500 for immunoblot. Anti-GRHL2 (Abcam, catalogue no. ab88631) used at 1:150 for IHC and 1:400 for immunoblot. Anti-PPARγ (Santa Cruz, catalogue no. 7273) used at 1:2000 for IHC and 1:500 for immunoblot. Anti-GATA3 (Cell Signalling, catalogue no. 5852) used at 1:800 for IHC and 1:1000 for immunoblot. Anti-GRHL3 (Abcam, Cambridge, UK, catalogue no. ab57612) used at 1:500 for immunoblot. Anti-KRT13 (Abnova, Taipei City, Taiwan, catalogue no. MAB1864) used at 1:1000 for immunoblot. Anti-BACT (Sigma-Aldrich, St. Louis, MO, USA, catalogue no. AC5441) used at 1:250 000 for immunoblot).
