# Supplementary material for: Heterarchy of transcription factors driving basal and luminal cell phenotypes in human urothelium
Source: Cell Death Differ. 2017 Mar 10;24(5):809–18. doi: 10.1038/cdd.2017.10 (PMC5423105; doi:10.1038/cdd.2017.10)
Supplement: Supplementary Methods [file cdd201710x1.docx]

**Supplementary Methods**

*Immunohistochemistry*

Human urothelial tissue samples were collected with National Health Service Research Ethics Committee approval and required informed consent from urological procedures that excluded urothelial neoplasia. Tissue for immunohistology was fixed for 24h in 10% (v/v) formalin, dehydrated and processed into paraffin wax. De-waxed 5μM tissue sections were blocked for endogenous peroxidase activity with 3% (v/v) hydrogen peroxide for 10 minutes and an immunoperoxidase detection system was used, with details for the respective antibodies given below. In all cases, appropriate positive and negative controls were included to exclude artefact.

Antigen retrieval. For FOXA1, P63, GATA3, CTCF and GRHL2, antigen retrieval was performed by microwave boiling of tissue sections in a 10mM citric acid buffer (pH 6.0) for 10 minutes followed by 10 minutes cooling on ice. For PPARγ antigen retrieval was performed by incubation of tissue sections in 0.1% (w/v) trypsin solution before boiling of tissue sections in a 10 mM citric acid buffer (pH 6.0), for 10 minutes followed by 10 minutes cooling on ice. Tissue sections were then rinsed in 10mM Tris-Buffered Saline (TBS), pH 7.6.

Antibody detection. For FOXA1, CTCF, P63 and GRHL2 antibodies an avidin/biotin-based detection system was used. Endogenous avidin & biotin sites were blocked with an Avidin/Biotin blocking kit (Vector labs, Peterborough, UK), before applying 10 % (v/v) rabbit serum (for mouse primary antibodies) or 10 % (v/v) goat serum (for rabbit primary antibodies), for 5 minutes to prevent non-specific binding of the secondary antibody. Primary antibodies (see Methods), were applied for 16 hours at 4 oC and after washing three times in TBS, biotinylated Rabbit anti-Mouse or Goat anti-Rabbit secondary antibodies (1/400 and 1/800 respectively, Dako Cytomation Ltd, Ely, UK), were applied for 30 minutes at ambient temperature. Binding sites were detected using a Vectastain Elite ABC kit (Vector labs, Peterborough, UK), as described in the supplied protocol.

For PPARγ and GATA3, a high sensitivity enzyme micropolymer kit was used. Tissue sections were blocked with 2.5% (v/v) horse serum for 20 minutes at ambient temperature before applying primary antibodies overnight at 4oC. After washing in TBS, appropriate rabbit or mouse amplifier antibodies were applied for 30 minutes at ambient temperature and binding sites were detected using ImmPRESS™ Excel Polymer detection kit (Vector labs, Peterborough, UK) according to manufacturer’s instructions.

All tissue sections were briefly counterstained in Mayer’s haematoxylin, dehydrated through graded ethanol washes into xylene, and mounted in DPX (Sigma Aldrich).

*Cell Culture*

NHU cell lines were established as detailed elsewhere ([1](#_ENREF_1)). For routine propagation cultures were maintained as monolayers in low calcium (0.09mM) KSFM containing BPE and EGF (Invitrogen) and further supplemented with cholera toxin (Sigma). Cultures were sub-cultured by trypsinisation at just confluence and used for experiments between passages 1 and 3. To induce differentiation cultures were treated with 1uM TZ and 1uM PD153035 ([2](#_ENREF_2)). Vehicle control non-differentiated cultures were maintained in parallel in KSFMc with 0.1% DMSO and used at the same time points. Independent sets of unique donor cells were used for sequencing, chromatin extracts, and siRNA experiments.

*Knock down of p63 and GATA3 by siRNA*

Urothelial cells in KSFM (no supplements) were transfected with 100nM siRNA purchased from Thermo Fisher Scientific from their Silencer^®^ Select pre-designed and validated siRNA product range (see table) at 80% confluent using Lipofectamine RNAiMAX transfection reagent (Invitrogen). Cells were incubated for 4 hr at 37 ^o^C at 5% CO_2_ in air after which the medium was supplemented with BPE, EGF and cholera toxin plus 1 µM PD153035 and 1 µM TZ to induce differentiation. The cells were incubated as such for 24 hours at 37 ^o^C at 5% CO_2_ in air. The medium was changed to KSFMc plus 1 μM PD153035 and incubated at 37 ^o^C at 5% CO_2_ in air for a further 24 hours when RNA and protein were harvested. P63 and GATA3 siRNA experiments were carried out using independent donor NHU cells.

*siRNA Oligomers*

| **siRNA Name (Catalogue #)** | **siRNA Name** |
| --- | --- |
| Silencer^®^ Select Negative Control #1 siRNA (4390843) | CTRL-siRNA1 |
| Silencer^®^ Select Negative Control #2 siRNA (4390846) | CTRL-siRNA2 |
| GATA3 Silencer^®^ Select siRNA (S5600) | GATA3-siRNA1 |
| GATA3 Silencer^®^ Select siRNA (S5601) | GATA3-siRNA2 |
| TP63 Silencer^®^ Select siRNA (S229399) | P63-siRNA1 |
| TP63 Silencer^®^ Select siRNA (S229400) | P63-siRNA2 |

*q-RTPCR Primers*

| **Target** | **Forward (5’ 🡪 3’)** | **Reverse (5’ 🡪 3’)** |
| --- | --- | --- |
| CK13 | TGTTGACTTTGGTGCTTGTGATG | GTTCTGCATGGTGATCTTCTCATT |
| FOXA1 | CAAGAGTTGCTTGACCGAAAGTT | TGTTCCCAGGGCCATCTGT |
| GAPDH | CAAGGTCATCCATGACAACTTTG | GGGCCATCCACAGTCTTCTG |
| GATA3 | TCATCACAAAATGAACGGACAGAA | TGTGGTTGTGGTGGTCTGACA |
| P63 | ATGGACCAGCAGATTCAGAAC | TGCGCGTGGTCTGTGTTATAG |
| PPARγ | GAACAGATCCAGTGGTTGCAG | CAGGCTCCACTTTGATTGCAC |
| UPK2 | CAGTGCCTCACCTTCCAACA | TGGTAAAATGGGAGGAAAGTCAA |
| IGFBP3 | CCCTGCCGTAGAGAAATGGA | GGACTCAGCACATTGAGGAACTT |
| IL1B | GCCGCGTCAGTTGTTGTG | TCTGTGGGCAGGGAACCA |
| F3 | CGACGAGATTGTGAAGGATGTG | CCTGCCGGGTAGGAGAAGAC |
| HBEGF | GGAGCTGACTGTTCTTGGTAACTG | TCCCCACCTCCAACCTTCTC |

*Real-time quantitative PCR*

Cultures for RNA extraction were lysed in situ with TRIzol (Life Technologies) and prepared according to the manufacturers protocol. RNA samples were treated with DNA-free kit (Ambion) and quantified by UV spectrometry. cDNA was synthesised from 1ug of total RNA using superscript first strand synthesis system (Life Technologies) with random hexamers (Thermo Scientific). For semi-quantitative analysis, template cDNA was mixed with SYBR-Green PCR Master Mix (ABI) and 300mM of each forward and reverse target gene primers (see table) and analysed on an ABI StepOnePlus Real Time PCR system. The thermal profile was: 20s hold at 95oC, followed by 40 cycles of denaturation at 95oC (3s) and elongation at 60oC (30s). Dissociation curves were performed to confirm presence of a single amplified product and the absence of primer dimers for each primer set. Assay efficiency, validated using the CT slope method prior to use, confirmed that both test and endogenous assays were of equivalent efficiency (within tolerance range). GAPDH was used as the internal control for comparison on every plate. To analyse the resulting data, cycle threshold (C_T_) values were extracted, and normalised to GAPDH expression. Fold change was calculated relative to a calibrator value (ie. the control scrambled sample).

Expression was plotted as Log_(2)_ of fold change, relative to scrambled-vector control cells after normalisation to GAPDH. Each data point is the average of three NHU cell lines from independent donors. Bars represent mean. Statistics: * P<0.1, ** P<0.01, *** P<0.001, **** P<0.0001 calculated using a 2 Way Anova with Dunnetts multiple comparison test.

*Immunoblotting*

Whole protein lysates were generated using a [sodium dodecyl sulfate](https://en.wikipedia.org/wiki/Sodium_dodecyl_sulfate) (SDS) lysis buffer containing 20% (v/v) glycerol, 2% (w/v) SDS, 125 mM Tris-HCl (pH 6.8), 200 mM NaF, 0.1 mM Na_3_PO_4_, 33 mM Na_3_PO_4_, freshly added 13 mM DTT and 1:100 dilution of protease inhibitor cocktail (Sigma Aldrich, Cat # P8340), and protein content quantified by a coomasie assay. 20ug protein was resolved on 4-12% gradient bis-Tris acrylamide NuPAGE gels (Life Technologies) and electrotransferred onto 0.45 µM PVDF-FL membranes (Merck Millipore). Membranes were probed with primary antibodies for 16h at 4^o^C. Bound antibody was detected with goat anti-rabbit Ig conjugated to IRDye 800 (1:10,000 dilution, Rockland Immunochemicals) or anti-mouse immunoglobulins conjugated to Alexa Fluor 680 (1:10,000 dilution, Life Technologies) as appropriate. Immunolabelled protein bands were visualised and relative quantifications generated using Odyssey infrared imaging system (LiCor). Densitometry analysis was completed using the accompanying Li-Cor software (Image Studio Lite version 5.0). Western blots were probed with β-actin which were used as loading controls for comparison.

Densitometry analysis of Western blot bands was normalised to β-actin and plotted as the log_(2)_ fold change of the NHU culture treated with target siRNA relative to control siRNA under equivalent conditions. All quantitation was performed on antibody labelling performed on the same blot. Blotting was performed sequentially for all antibodies where multiple proteins are labelled on the same blot. Labelling on figure 6 and supplementary figure 2 bands are shown on different images of the same blot, because either sequential blotting with stripping of antibodies (Alpha Diagnostic International, Catalogue # 90100) was performed as in the case of FOXA1, or secondary antobodies fluorescing in different wavelengths were used as in the case of GATA3. Statistics: * P<0.1, ** P<0.01, *** P<0.001, **** P<0.0001 calculated by performing a 1 way Repeated Measures Anova with Greenhous-Geisser correction and Sidaks multiple comparison test with individual variances computed for each comparison

*Sequencing Sample Quality Control*

FAIRE DNA sample QC was performed by checking fragment-size distribution using the Bioanalyzer DNA HS assay (Agilent Technologies) and further quantified using the Qubit dsDNA HS Assay Kit (Life Technologies). Small DNA fragments <50 bp were removed from FAIRE samples by size-selecting fragments at 250 bp using an EZ-Gel size select (Life Technologies), and recovered fragment size was confirmed using a LabChip GX (Perkin Elmer). RNA sample QC was performed using the Bioanalyzer RNA 6000 Nano Assay (Agilent Technologies, Inc, CA) and further quantified using the Qubit RNA Assay Kit (Life Technologies).

*Library Quality Control*

All libraries were QC checked with the Bioanalyzer DNA HS assay system (Agilent Technologies Inc., CA) and quantified by Qubit dsDNA HS Assay Kit (Life Technologies, Carlsbad, CA) before pooling. Pooled libraries were further quantified by qPCR using a KAPA Library Quantification Kit from Illumina/ABI Prism (Kapa Biosystems, Inc. Wilmington, MA) on a StepOnePlus™ Real-Time PCR System (Life Technologies. Carlsbad, CA).

*RNA-seq library preparation and sequencing*

Library construction was performed using TruSeq RNA Sample Prep Kit v2 according to the manufacturer’s instructions (Illumina, Inc). Single-end sequencing (51 and 101bp) with a 1% PhiX spike was performed using an Illumina HiSeq 2500 sequencer.

*RNA-seq Data Processing*

51bp reads were aligned to the UCSC hg19 human genome using RSEM v1.2.8 ([3](#_ENREF_3)). The RefSeq genes annotations (downloaded on August 5, 2014 from UCSC) were used as the reference transcript set. The annotation was filtered to remove genes located on non-standard chromosomes to give a set of 47969 transcripts representing 25609 genes. RSEM was run using default parameters using Bowtie v1.0.0 for the alignment. Differential expression analysis was carried out using DESeq v.1.14.0 ([4](#_ENREF_4)). The expected_count obtained from RSEM was rounded down to the nearest whole number for inputting into DESeq. The per-condition method was used to estimate dispersion and the nbinomTest was used for differential expression analysis.

*Gene Ontology Analysis*

Lists of genes found to be differentially expressed by RNA-seq were submitted to the GOrilla tool (http://cbl-gorilla.cs.technion.ac.il/) as two unranked lists, with differentially regulated genes as target list and all genes as background, with the “process” ontology and default parameters selected.

*FAIRE-seq library preparation and sequencing*

FAIRE library construction was performed on 10 ng using a TruSeq ChIP Sample prep Kit according to the manufacturer’s instructions (Illumina, Inc). FAIRE-seq was performed using paired-end sequencing (2 x101 bp), with a 1% PhiX spike, on an Illumina HiSeq 2500 sequencer.

*FAIRE-seq data processing*

Reads for each sample were aligned to the Human genome reference UCSC hg19 (standard chromosomes) using Bowtie v2.0.6 ([5](#_ENREF_5)) using the options --no-discordant --no-mixed to suppress discordant and unpaired alignments for paired reads with a default fragment length of 500bp. The bam files were merged for the three patients for each time point and treatment, then peaks were called using MACS version 1.4.2 using default parameters and p-value cutoff = 1.00e-03 ([6](#_ENREF_6)).

*Finding Differential FAIRE-seq Peaks and Motif Matching with HOMER*

The peak files were annotated with the nearest gene using HOMER. Peaks annotated with promoter_TSS were removed from the peak files (7.5 to 12% of peaks). Using bedtools, peak lists were obtained which were unique in the control sample when compared with the treated sample and unique in the treated when compared to the control at 24 h and 144 h. These four peak lists were submitted to HOMER using the opposing control or differentiated peak lists at the relevant time point as the custom background, using the additional –mask –size300 options. A further set of peaks were obtained by selecting only those peaks within ±25kb of differentially expressed genes at each of the time-points relative to 24 h control. These four peak lists were submitted to HOMER as described above.

**References**

1. Southgate J, Hutton KA, Thomas DF, Trejdosiewicz LK. Normal human urothelial cells in vitro: proliferation and induction of stratification. Lab Invest. 1994;71(4):583-94.

2. Varley CL, Stahlschmidt J, Lee WC, Holder J, Diggle C, Selby PJ, et al. Role of PPARgamma and EGFR signalling in the urothelial terminal differentiation programme. J Cell Sci. 2004;117(Pt 10):2029-36.

3. Li B, Dewey CN. RSEM: accurate transcript quantification from RNA-Seq data with or without a reference genome. BMC Bioinformatics. 2011;12(1):323.

4. Anders S, Huber W. Differential expression analysis for sequence count data. Genome Biol. 2010;11(10):R106.

5. Langmead B, Salzberg SL. Fast gapped-read alignment with Bowtie 2. Nat Methods. 2012;9(4):357-9.

6. Feng J, Liu T, Zhang Y. Using MACS to Identify Peaks from ChIP‐Seq Data. Current Protocols in Bioinformatics. 2011:2.14. 1-2.
