# Supplementary figures and images for: Heterarchy of transcription factors driving basal and luminal cell phenotypes in human urothelium
Source: Cell Death Differ. 2017 Mar 10;24(5):809–18. doi: 10.1038/cdd.2017.10 (PMC5423105; doi:10.1038/cdd.2017.10)

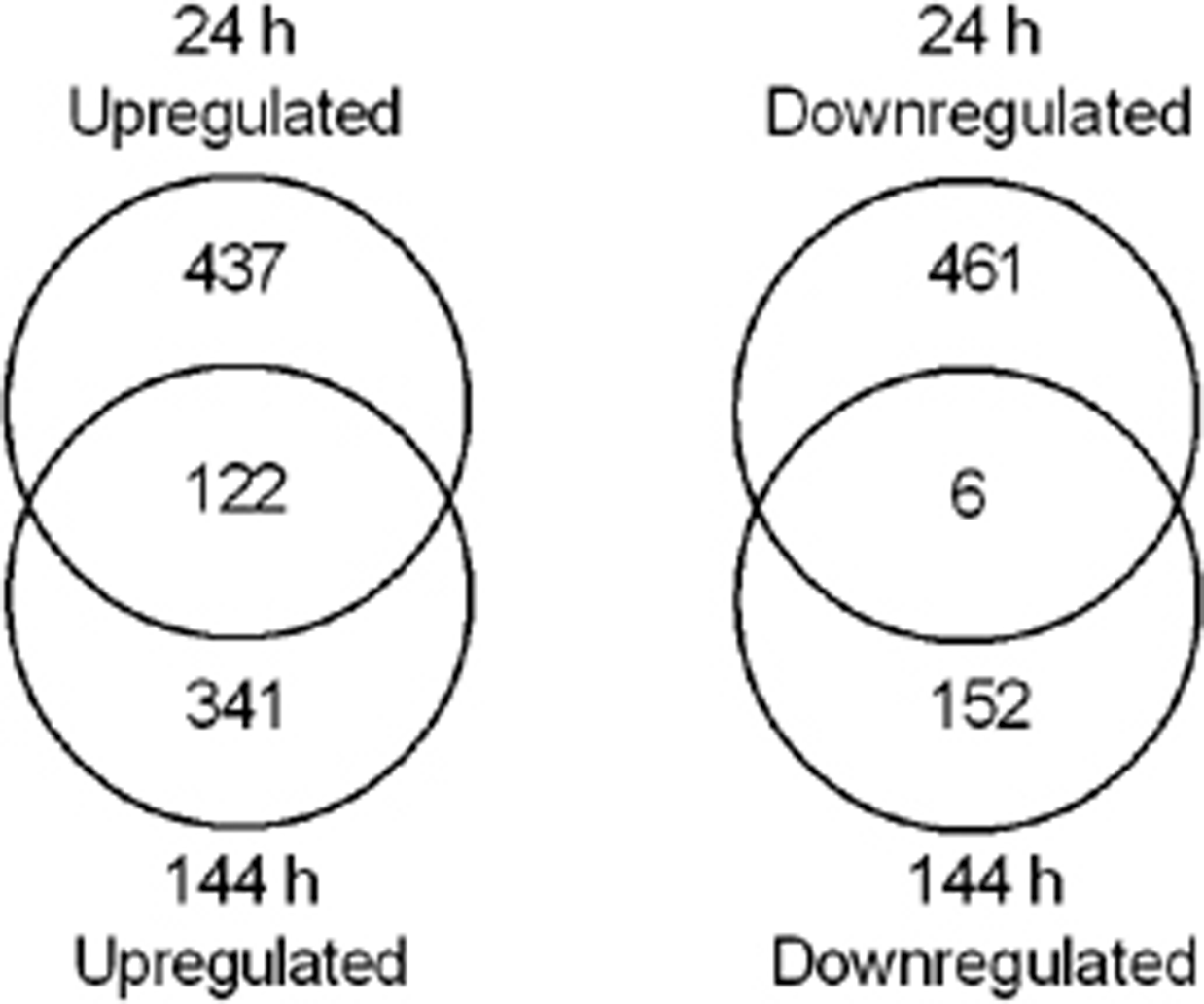

Supplement: Supplementary Figure 1 [file cdd201710x2.tif]

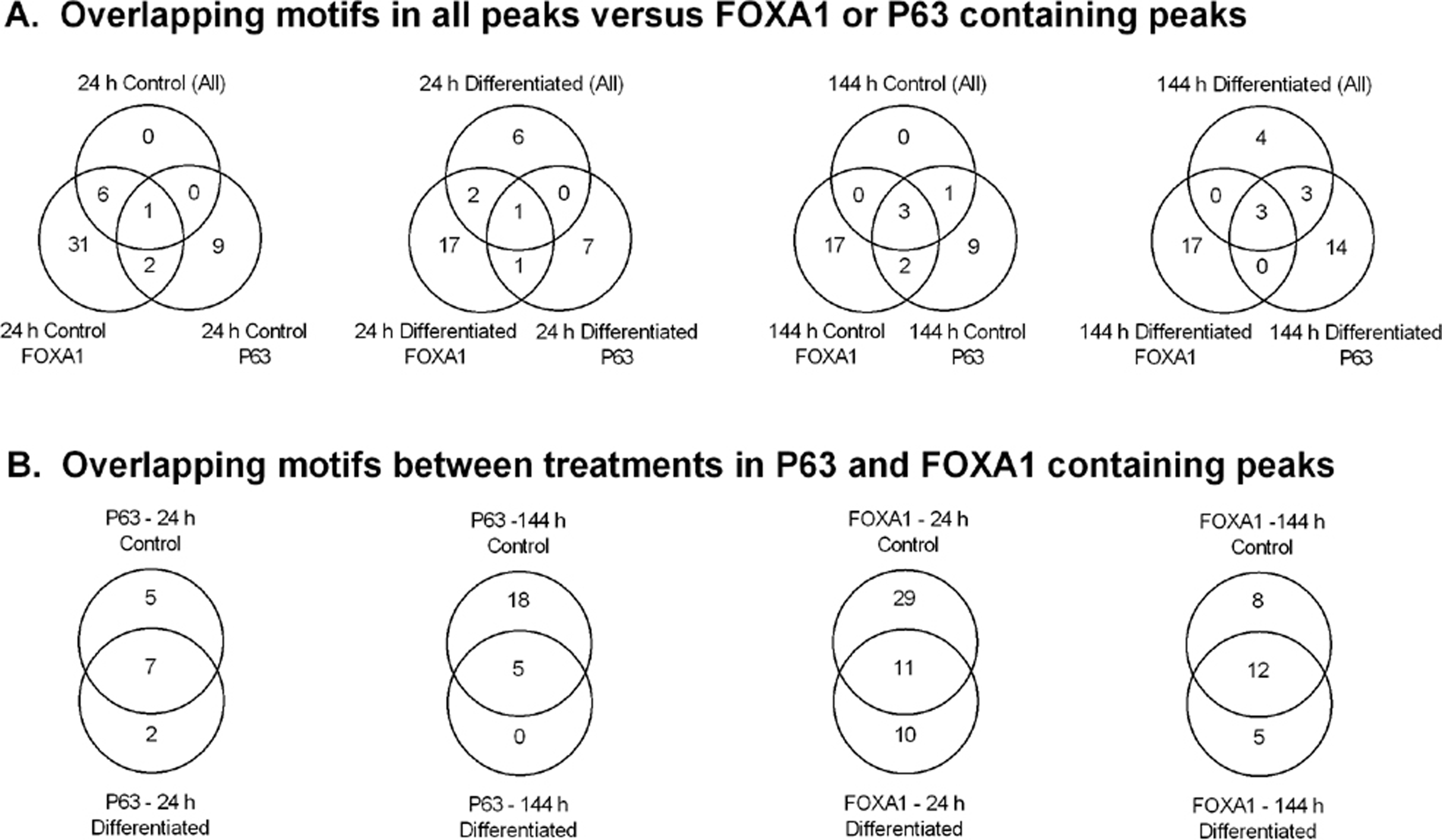

Supplement: Supplementary Figure 2 [file cdd201710x3.tif]

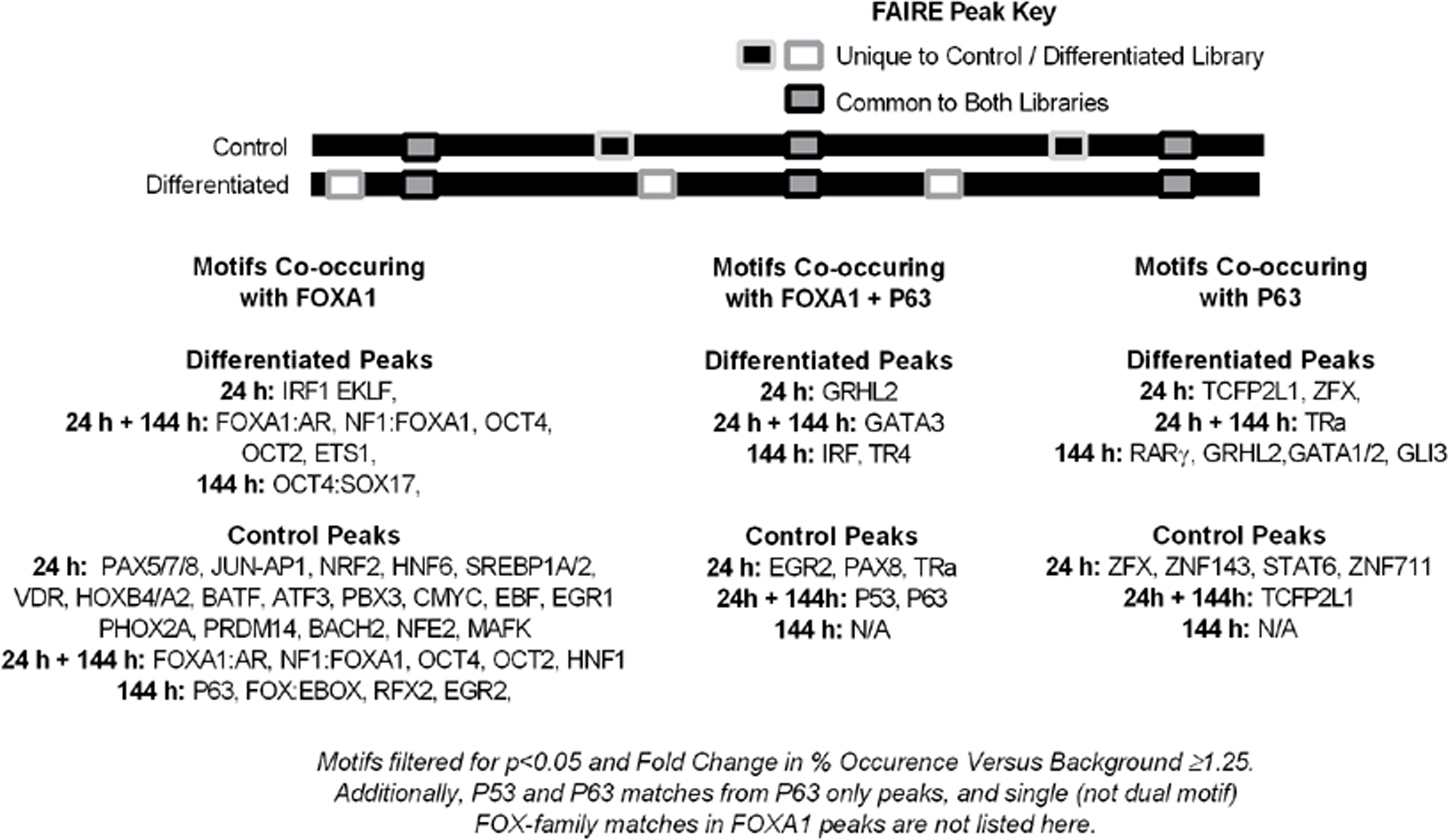

Supplement: Supplementary Figure 3 [file cdd201710x4.tif]

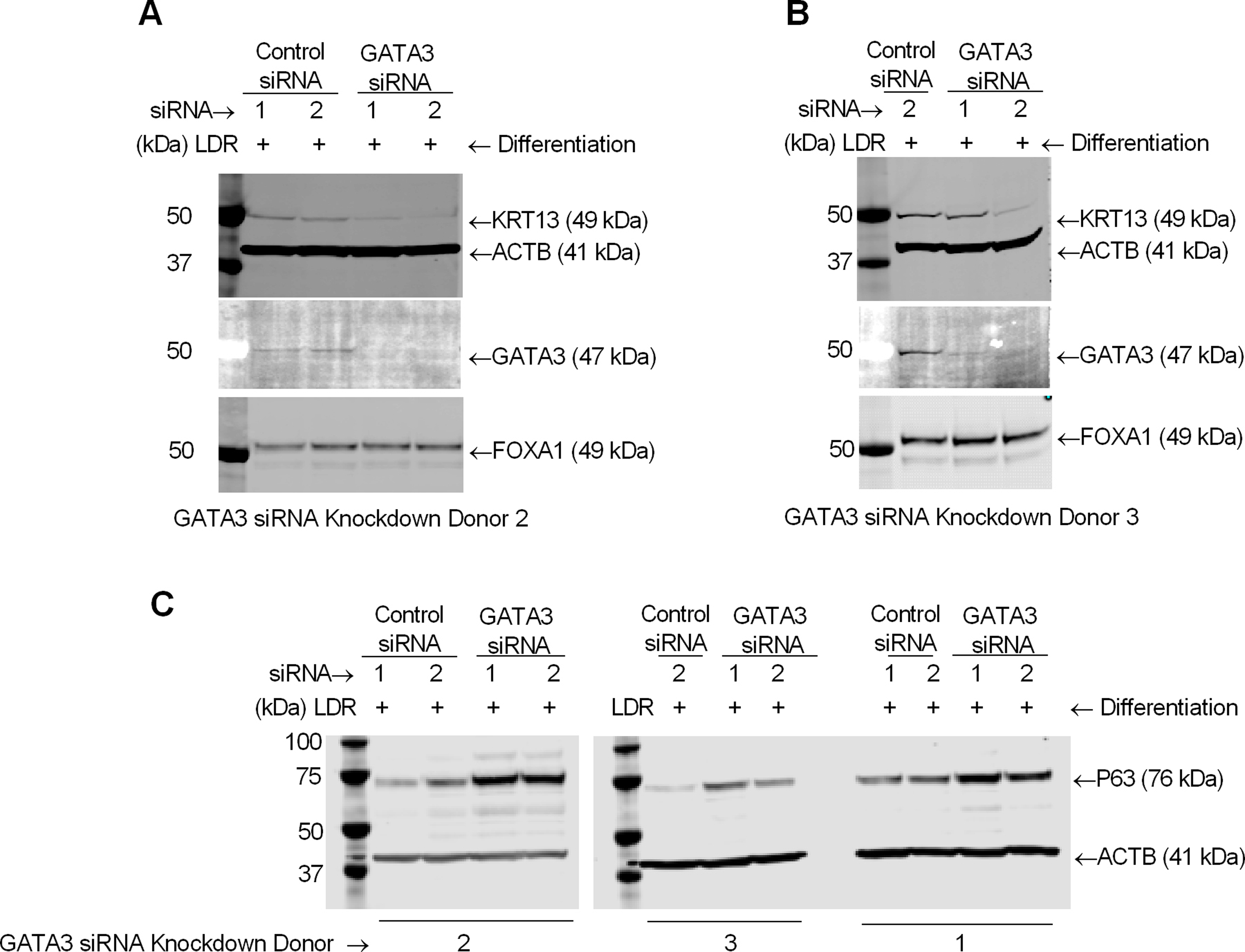

Supplement: Supplementary Figure 5 [file cdd201710x6.tif]
