# Supplementary material for: Heterarchy of transcription factors driving basal and luminal cell phenotypes in human urothelium
Source: Cell Death Differ. 2017 Mar 10;24(5):809–18. doi: 10.1038/cdd.2017.10 (PMC5423105; doi:10.1038/cdd.2017.10)
Supplement: Supplementary Tables 1-4 [file cdd201710x7.docx]

Supplementary Table 1A. Genes differentially regulated at 24 h.

| Gene ID | 24 h Control RPKM | 24 h Differentiated RPKM | log2 Fold Change | Adjusted p-value |
| --- | --- | --- | --- | --- |
| EGR3 | 86.89 | 0.34 | -8.00 | 8.53E-12 |
| DUSP6 | 6020.24 | 84.39 | -6.16 | 2.97E-08 |
| SPRY4 | 746.46 | 13.44 | -5.80 | 3.13E-07 |
| CSF2 | 18.61 | 0.35 | -5.73 | 0.012009641 |
| EGR1 | 4746.23 | 100.43 | -5.56 | 9.42E-42 |
| LINC00704 | 441.55 | 9.58 | -5.53 | 4.76E-08 |
| IL24 | 15.92 | 0.40 | -5.31 | 0.035093405 |
| SAMD3 | 14.14 | 0.40 | -5.14 | 0.063075285 |
| ETV5 | 761.65 | 24.76 | -4.94 | 0.01188421 |
| ETV4 | 542.11 | 18.43 | -4.88 | 7.68E-28 |
| C20orf197 | 299.77 | 11.17 | -4.75 | 0.023173537 |
| MT2A | 21078.81 | 814.82 | -4.69 | 8.98E-06 |
| G0S2 | 11600.11 | 449.36 | -4.69 | 0.001727726 |
| CTGF | 2365.46 | 95.50 | -4.63 | 3.24E-08 |
| MYH16 | 245.47 | 10.52 | -4.54 | 1.90E-15 |
| RPSAP52 | 113.79 | 5.16 | -4.46 | 9.62E-11 |
| DUSP5 | 2435.25 | 111.04 | -4.45 | 1.23E-30 |
| FOXA2 | 56.54 | 2.58 | -4.45 | 4.33E-05 |
| FOSL1 | 1604.71 | 75.35 | -4.41 | 5.76E-05 |
| NAV3 | 150.29 | 7.32 | -4.36 | 1.22E-10 |
| MMP9 | 865.90 | 45.50 | -4.25 | 1.48E-11 |
| TFPI2 | 6078.14 | 321.53 | -4.24 | 4.85E-05 |
| TGM2 | 4167.85 | 231.66 | -4.17 | 7.36E-10 |
| DOK7 | 54.93 | 3.22 | -4.09 | 6.57E-05 |
| MMP1 | 3176.46 | 193.35 | -4.04 | 0.03610619 |
| ARHGAP25 | 124.90 | 7.88 | -3.99 | 1.52E-05 |
| DSCAM | 40.45 | 2.91 | -3.80 | 0.001014511 |
| ADAM8 | 1374.79 | 101.85 | -3.75 | 2.78E-12 |
| DUSP2 | 51.34 | 3.94 | -3.70 | 0.039880231 |
| GJA4 | 21.83 | 1.82 | -3.58 | 0.040935635 |
| SYNE3 | 48.02 | 4.06 | -3.57 | 0.001074677 |
| MMP10 | 1596.92 | 135.02 | -3.56 | 0.007533927 |
| PLAT | 18318.95 | 1629.20 | -3.49 | 4.58E-24 |
| PCDHGC5 | 52.42 | 4.88 | -3.42 | 0.000288734 |
| IL7R | 186.43 | 17.53 | -3.41 | 0.097520424 |
| HEY1 | 22.09 | 2.13 | -3.38 | 0.072918728 |
| CYP27B1 | 438.65 | 42.92 | -3.35 | 5.80E-08 |
| UPP1 | 3701.58 | 369.43 | -3.32 | 1.96E-16 |
| SMOC1 | 1156.87 | 116.02 | -3.32 | 5.14E-11 |
| SPDEF | 334.93 | 33.75 | -3.31 | 0.000263173 |
| PHACTR3 | 70.67 | 7.65 | -3.21 | 0.025437409 |
| HMGA2 | 6184.92 | 671.71 | -3.20 | 3.48E-05 |
| PTPRE | 597.84 | 67.75 | -3.14 | 1.53E-09 |
| GPR3 | 41.58 | 4.79 | -3.12 | 0.004414855 |
| RHEBL1 | 25.12 | 2.93 | -3.10 | 0.047088981 |
| MCTP1 | 117.45 | 14.46 | -3.02 | 4.41E-06 |
| LOC100506178 | 26.82 | 3.31 | -3.02 | 0.076045514 |
| CSMD3 | 120.54 | 14.91 | -3.01 | 1.29E-06 |
| XDH | 3315.96 | 417.19 | -2.99 | 5.15E-09 |
| STAMBPL1 | 48.67 | 6.16 | -2.98 | 0.002867772 |
| CNIH3 | 35.16 | 4.52 | -2.96 | 0.04472036 |
| KCNMA1 | 670.21 | 88.59 | -2.92 | 3.98E-07 |
| PTX3 | 91.58 | 12.12 | -2.92 | 0.086632808 |
| FOS | 1110.48 | 150.06 | -2.89 | 9.35E-05 |
| SLCO4A1 | 60.71 | 8.30 | -2.87 | 0.013211534 |
| MT1L | 1175.38 | 161.00 | -2.87 | 9.90E-07 |
| SOX9 | 943.76 | 129.34 | -2.87 | 1.51E-10 |
| ERRFI1 | 8612.53 | 1184.66 | -2.86 | 6.17E-10 |
| SERTAD4 | 98.61 | 13.58 | -2.86 | 0.00933774 |
| GJA3 | 39.04 | 5.44 | -2.84 | 0.012474087 |
| TRPV2 | 84.35 | 12.18 | -2.79 | 0.000524765 |
| ZBED2 | 1946.61 | 283.54 | -2.78 | 4.07E-07 |
| CYR61 | 6582.04 | 978.38 | -2.75 | 1.71E-06 |
| FAM132B | 82.77 | 12.50 | -2.73 | 0.084640059 |
| SERTAD4-AS1 | 47.70 | 7.23 | -2.72 | 0.061607731 |
| NTM | 823.83 | 128.30 | -2.68 | 0.006027884 |
| POU2F2 | 741.98 | 116.48 | -2.67 | 0.006176684 |
| SPRY2 | 215.17 | 34.01 | -2.66 | 0.001322045 |
| SH2D2A | 52.94 | 8.53 | -2.63 | 0.01188421 |
| INHBA | 2554.72 | 413.96 | -2.63 | 0.002675144 |
| CCNE2 | 108.14 | 17.70 | -2.61 | 0.000345106 |
| C11orf91 | 57.53 | 9.69 | -2.57 | 0.007602406 |
| DUSP4 | 2703.84 | 458.51 | -2.56 | 0.005254533 |
| IL11 | 150.81 | 25.69 | -2.55 | 4.86E-06 |
| DTL | 501.01 | 85.49 | -2.55 | 0.004381626 |
| ANTXR2 | 4500.08 | 773.50 | -2.54 | 6.17E-10 |
| SUSD4 | 43.29 | 7.46 | -2.54 | 0.020306043 |
| KHDRBS3 | 87.00 | 15.27 | -2.51 | 0.040986967 |
| SLC6A14 | 554.30 | 99.60 | -2.48 | 7.58E-10 |
| NLRP3 | 152.09 | 27.46 | -2.47 | 0.00236131 |
| IER3 | 6086.51 | 1099.05 | -2.47 | 9.90E-08 |
| SH2D5 | 1031.95 | 190.66 | -2.44 | 1.26E-08 |
| CDCA7L | 524.77 | 97.07 | -2.43 | 0.000694356 |
| ADAMTS6 | 780.93 | 145.36 | -2.43 | 0.008624004 |
| IL1A | 10093.48 | 1904.06 | -2.41 | 0.006935262 |
| HBEGF | 1201.78 | 234.28 | -2.36 | 1.12E-09 |
| STEAP1B | 89.69 | 17.60 | -2.35 | 0.015482349 |
| PLK3 | 839.28 | 166.69 | -2.33 | 0.000521378 |
| MCM10 | 265.72 | 53.50 | -2.31 | 0.009675961 |
| CITED4 | 1188.83 | 240.83 | -2.30 | 7.56E-07 |
| SLC20A1 | 3682.14 | 748.15 | -2.30 | 6.46E-11 |
| PTHLH | 5929.15 | 1206.24 | -2.30 | 3.96E-07 |
| F3 | 16063.87 | 3289.59 | -2.29 | 5.14E-09 |
| ARL4C | 5675.33 | 1170.91 | -2.28 | 8.27E-07 |
| ZNF367 | 124.55 | 25.74 | -2.27 | 0.001442478 |
| E2F7 | 1043.13 | 217.60 | -2.26 | 0.013469836 |
| LBH | 354.80 | 74.37 | -2.25 | 0.022911927 |
| TAGLN3 | 142.37 | 29.89 | -2.25 | 0.000403534 |
| EDNRA | 1044.18 | 221.05 | -2.24 | 3.36E-07 |
| MAR04 | 233.05 | 49.89 | -2.22 | 0.086632808 |
| ADTRP | 267.70 | 57.37 | -2.22 | 2.31E-06 |
| NCALD | 85.79 | 18.39 | -2.22 | 0.00231899 |
| OSBPL6 | 266.84 | 57.41 | -2.22 | 0.063075285 |
| CEMIP | 242.87 | 52.56 | -2.21 | 0.000249502 |
| PLEK2 | 2712.56 | 587.84 | -2.21 | 6.66E-08 |
| KIF21B | 222.11 | 48.45 | -2.20 | 0.000403534 |
| BTBD19 | 76.86 | 16.88 | -2.19 | 0.0263145 |
| CXCL8 | 874.03 | 198.33 | -2.14 | 0.088978049 |
| E2F1 | 134.57 | 30.77 | -2.13 | 0.002255143 |
| FOXD1 | 250.82 | 58.27 | -2.11 | 1.93E-05 |
| KLRC1 | 69.02 | 16.11 | -2.10 | 0.010583041 |
| EXO1 | 209.26 | 48.87 | -2.10 | 0.001089828 |
| KCNC4 | 113.80 | 26.64 | -2.09 | 0.005794651 |
| CDC45 | 323.47 | 75.98 | -2.09 | 0.001109528 |
| TMEM158 | 39.54 | 9.31 | -2.09 | 0.083717371 |
| HES6 | 73.40 | 17.34 | -2.08 | 0.038096975 |
| CDC6 | 539.88 | 128.74 | -2.07 | 0.000326405 |
| ITGA2 | 9587.16 | 2292.19 | -2.06 | 2.21E-09 |
| LOC284080 | 174.18 | 42.10 | -2.05 | 0.001067545 |
| RAD51AP1 | 331.07 | 81.33 | -2.03 | 0.001094633 |
| LIF | 584.08 | 146.20 | -2.00 | 0.043493858 |
| MAMDC2 | 191.34 | 48.06 | -1.99 | 0.002350723 |
| C17orf51 | 548.33 | 138.30 | -1.99 | 9.86E-05 |
| IL1RL1 | 74.31 | 18.86 | -1.98 | 0.017003486 |
| CD274 | 1784.76 | 455.51 | -1.97 | 8.84E-06 |
| WDR62 | 190.21 | 48.63 | -1.97 | 0.018628351 |
| TGFBR2 | 9149.36 | 2343.34 | -1.97 | 4.00E-06 |
| EIF5A2 | 338.19 | 87.07 | -1.96 | 0.095580724 |
| INSC | 55.64 | 14.38 | -1.95 | 0.051040947 |
| LINC00152 | 334.04 | 87.28 | -1.94 | 0.097451035 |
| KLHL4 | 61.09 | 15.98 | -1.93 | 0.070456719 |
| PAQR5 | 116.67 | 30.63 | -1.93 | 0.017169407 |
| CORO1A | 468.30 | 123.70 | -1.92 | 0.012193468 |
| TSPAN4 | 840.42 | 222.72 | -1.92 | 6.80E-07 |
| TREM1 | 67.24 | 17.82 | -1.92 | 0.077751856 |
| ARHGDIB | 1214.99 | 322.68 | -1.91 | 0.035100512 |
| TMEM156 | 197.11 | 52.54 | -1.91 | 0.002890987 |
| TMEM204 | 124.47 | 33.21 | -1.91 | 0.010645934 |
| LAMA3 | 101324.16 | 27059.39 | -1.90 | 0.051189567 |
| GINS2 | 287.53 | 76.95 | -1.90 | 0.012055843 |
| BAMBI | 157.96 | 42.91 | -1.88 | 0.012529445 |
| PKMYT1 | 312.78 | 85.40 | -1.87 | 0.0233663 |
| MAFF | 939.83 | 257.34 | -1.87 | 1.11E-06 |
| LOC100129940 | 53.10 | 14.57 | -1.87 | 0.097803955 |
| SIGLEC15 | 67.61 | 18.58 | -1.86 | 0.033776219 |
| UBASH3B | 605.31 | 167.41 | -1.85 | 0.000166459 |
| CTNNAL1 | 1700.45 | 473.07 | -1.85 | 0.06996845 |
| CD83 | 68.12 | 19.02 | -1.84 | 0.029961337 |
| LRRC8C | 1513.79 | 423.16 | -1.84 | 0.00352942 |
| DSCC1 | 128.49 | 35.95 | -1.84 | 0.012486324 |
| CTH | 159.75 | 44.87 | -1.83 | 0.013287157 |
| ZNF697 | 520.73 | 147.32 | -1.82 | 0.000581579 |
| ODC1 | 2123.68 | 601.69 | -1.82 | 0.078585675 |
| GRK5 | 281.89 | 79.99 | -1.82 | 0.000142125 |
| FJX1 | 993.64 | 283.57 | -1.81 | 2.24E-06 |
| MT1E | 1311.88 | 374.64 | -1.81 | 0.003912116 |
| KIF26B | 301.49 | 86.19 | -1.81 | 0.000122072 |
| LPXN | 496.69 | 142.25 | -1.80 | 0.060207274 |
| LINC00941 | 80.71 | 23.15 | -1.80 | 0.039880231 |
| EMP3 | 705.28 | 202.53 | -1.80 | 0.000581579 |
| FAM111B | 250.99 | 72.46 | -1.79 | 0.003994714 |
| SNX10 | 122.45 | 35.51 | -1.79 | 0.007848097 |
| RRM2 | 1132.45 | 332.39 | -1.77 | 0.007698367 |
| VEGFC | 1245.60 | 365.61 | -1.77 | 0.000177467 |
| CDCA7 | 742.12 | 218.30 | -1.77 | 0.026870371 |
| PRDM8 | 203.27 | 59.89 | -1.76 | 0.00236131 |
| TRIP13 | 694.88 | 205.99 | -1.75 | 0.090760091 |
| STEAP1 | 216.09 | 64.15 | -1.75 | 0.059161184 |
| RAD51 | 227.37 | 67.62 | -1.75 | 0.024642974 |
| DOCK4 | 573.66 | 170.81 | -1.75 | 0.000246297 |
| TIMP1 | 1953.76 | 583.05 | -1.74 | 0.017002375 |
| PEX5L | 171.21 | 51.24 | -1.74 | 0.013776864 |
| DDIAS | 313.32 | 95.51 | -1.71 | 0.024642974 |
| CDCA5 | 597.21 | 182.31 | -1.71 | 0.009925524 |
| DNAJB5 | 781.94 | 239.22 | -1.71 | 8.10E-05 |
| MIR4435-1HG | 463.62 | 142.57 | -1.70 | 0.03707077 |
| CXCL3 | 285.81 | 87.94 | -1.70 | 0.044335603 |
| ABCG1 | 413.90 | 127.59 | -1.70 | 0.000155749 |
| CCDC15 | 81.16 | 25.26 | -1.68 | 0.03325942 |
| KIAA0040 | 1283.89 | 399.99 | -1.68 | 9.06E-06 |
| RAC2 | 1696.91 | 529.06 | -1.68 | 0.000117665 |
| SPRED1 | 723.92 | 225.93 | -1.68 | 4.33E-05 |
| CAPRIN2 | 1464.92 | 457.76 | -1.68 | 0.002986305 |
| MCM5 | 692.21 | 217.18 | -1.67 | 0.047023501 |
| PSMC3IP | 207.95 | 65.28 | -1.67 | 0.001493892 |
| SH3KBP1 | 1198.14 | 377.16 | -1.67 | 1.58E-05 |
| MICAL2 | 6019.27 | 1895.19 | -1.67 | 0.077702752 |
| LAMB3 | 77008.76 | 24256.40 | -1.67 | 0.007815981 |
| IL27RA | 245.59 | 77.47 | -1.66 | 0.004381626 |
| TNFRSF12A | 5486.34 | 1733.37 | -1.66 | 4.10E-06 |
| DDIT3 | 813.48 | 257.61 | -1.66 | 0.002066734 |
| THBS2 | 4129.67 | 1308.62 | -1.66 | 0.001109528 |
| CDC42EP2 | 322.44 | 102.47 | -1.65 | 0.000408364 |
| CLSPN | 275.20 | 87.50 | -1.65 | 0.000664235 |
| STX1A | 197.49 | 63.09 | -1.65 | 0.014631687 |
| IVNS1ABP | 4545.39 | 1458.20 | -1.64 | 4.77E-06 |
| BLM | 283.36 | 90.91 | -1.64 | 0.0130054 |
| SPRED3 | 157.41 | 50.52 | -1.64 | 0.008724026 |
| JAG1 | 9892.92 | 3176.35 | -1.64 | 0.000493437 |
| CSRNP1 | 375.21 | 120.82 | -1.63 | 0.001364028 |
| PMEPA1 | 6696.81 | 2156.70 | -1.63 | 0.000664235 |
| SMTN | 2761.48 | 891.98 | -1.63 | 0.002703741 |
| VEGFA | 8104.71 | 2626.00 | -1.63 | 0.000454309 |
| MYBL1 | 127.91 | 41.69 | -1.62 | 0.098741943 |
| LTBP2 | 7414.34 | 2419.90 | -1.62 | 6.40E-06 |
| ENC1 | 2341.32 | 769.31 | -1.61 | 0.030472782 |
| LAMC2 | 231018.37 | 76467.46 | -1.60 | 0.05634311 |
| PAG1 | 163.44 | 54.11 | -1.59 | 0.053244543 |
| TUBA1B | 25799.40 | 8607.05 | -1.58 | 0.04294334 |
| TUBB2A | 735.15 | 245.44 | -1.58 | 0.02263651 |
| CD44 | 28046.95 | 9528.20 | -1.56 | 1.37E-05 |
| EME1 | 147.87 | 50.39 | -1.55 | 0.033372921 |
| FANCB | 72.93 | 24.90 | -1.55 | 0.068868142 |
| SEMA7A | 1320.54 | 450.99 | -1.55 | 0.000597511 |
| CSGALNACT2 | 660.91 | 226.81 | -1.54 | 0.010206872 |
| CLMP | 71.41 | 24.56 | -1.54 | 0.081704299 |
| EIF4EBP1 | 1333.48 | 460.11 | -1.54 | 0.000104586 |
| DCBLD2 | 18152.66 | 6287.20 | -1.53 | 0.001109528 |
| ADAM19 | 1993.36 | 691.27 | -1.53 | 0.033734299 |
| PGBD5 | 342.83 | 120.17 | -1.51 | 0.042273903 |
| KIAA1549L | 658.49 | 232.13 | -1.50 | 0.012850411 |
| SSFA2 | 5843.68 | 2064.35 | -1.50 | 0.000708569 |
| FBXO5 | 282.09 | 99.81 | -1.50 | 0.009046426 |
| FSCN1 | 9424.16 | 3339.93 | -1.50 | 0.000233777 |
| WDR76 | 216.27 | 76.90 | -1.49 | 0.026098838 |
| LETM2 | 127.75 | 45.44 | -1.49 | 0.025196574 |
| RTTN | 1413.06 | 503.26 | -1.49 | 0.003946845 |
| MTHFD2 | 2839.31 | 1014.62 | -1.48 | 0.003089565 |
| CENPU | 486.80 | 174.01 | -1.48 | 0.03288251 |
| TRIM47 | 198.38 | 70.92 | -1.48 | 0.076263224 |
| PRNP | 10631.97 | 3822.71 | -1.48 | 0.001060901 |
| ETS1 | 6301.33 | 2267.25 | -1.47 | 0.039951966 |
| SMOX | 356.96 | 129.51 | -1.46 | 0.01695584 |
| PAQR4 | 397.73 | 144.66 | -1.46 | 0.012474087 |
| FAM107B | 588.04 | 216.48 | -1.44 | 0.00910808 |
| CSRP1 | 2554.84 | 942.75 | -1.44 | 0.018982936 |
| PHLDA2 | 1332.85 | 493.76 | -1.43 | 0.015954654 |
| PLK4 | 505.72 | 187.54 | -1.43 | 0.050661218 |
| MCM6 | 827.98 | 309.85 | -1.42 | 0.004003752 |
| SPAG1 | 182.69 | 68.37 | -1.42 | 0.099822767 |
| HELLS | 220.37 | 82.65 | -1.41 | 0.008737009 |
| FERMT1 | 3259.57 | 1224.93 | -1.41 | 0.007463061 |
| CDK5R1 | 123.82 | 46.56 | -1.41 | 0.039489535 |
| CCDC68 | 149.15 | 56.21 | -1.41 | 0.033964425 |
| NUAK1 | 1159.04 | 436.89 | -1.41 | 0.007134801 |
| EPHA2 | 6244.80 | 2364.52 | -1.40 | 0.000665996 |
| GALNT18 | 505.33 | 191.49 | -1.40 | 0.002768338 |
| BRCA1 | 775.79 | 294.92 | -1.40 | 0.014263901 |
| STK17A | 3211.28 | 1224.00 | -1.39 | 0.000356478 |
| TRIB1 | 591.70 | 225.61 | -1.39 | 0.012796739 |
| SLC35F3 | 203.28 | 77.76 | -1.39 | 0.012529445 |
| SPRED2 | 903.45 | 346.26 | -1.38 | 0.000876706 |
| C3orf67 | 130.61 | 50.16 | -1.38 | 0.070000804 |
| LINC00467 | 136.89 | 52.74 | -1.38 | 0.035392033 |
| ITGA6 | 24054.55 | 9310.73 | -1.37 | 0.016742308 |
| FEN1 | 656.86 | 254.51 | -1.37 | 0.007242033 |
| EVA1A | 383.00 | 148.40 | -1.37 | 0.004451106 |
| BICD1 | 211.37 | 82.18 | -1.36 | 0.015058184 |
| UAP1 | 1706.44 | 665.57 | -1.36 | 0.029260995 |
| ORC6 | 366.81 | 143.82 | -1.35 | 0.061464284 |
| RFC3 | 412.97 | 162.07 | -1.35 | 0.02608615 |
| PLGRKT | 541.10 | 212.43 | -1.35 | 0.002478887 |
| RPL21 | 358.94 | 142.30 | -1.33 | 0.014167643 |
| PLAUR | 847.56 | 336.10 | -1.33 | 0.00548295 |
| INPP4B | 1023.87 | 406.02 | -1.33 | 0.001658985 |
| TUBE1 | 281.73 | 111.87 | -1.33 | 0.057885168 |
| FKBP9P1 | 270.42 | 107.46 | -1.33 | 0.031121204 |
| UBALD2 | 787.63 | 313.01 | -1.33 | 0.00188505 |
| SPDL1 | 542.21 | 215.81 | -1.33 | 0.014998956 |
| NRCAM | 1602.49 | 640.26 | -1.32 | 0.003964479 |
| HS3ST1 | 144.68 | 57.90 | -1.32 | 0.043698372 |
| WDHD1 | 746.77 | 299.34 | -1.32 | 0.001887569 |
| RFC2 | 427.72 | 172.10 | -1.31 | 0.009046426 |
| CKLF | 395.93 | 159.55 | -1.31 | 0.005708401 |
| TGFA | 4074.13 | 1643.19 | -1.31 | 0.01241895 |
| MCM4 | 1663.87 | 672.65 | -1.31 | 0.009341962 |
| JUN | 3457.52 | 1398.90 | -1.31 | 0.03504272 |
| NAGS | 173.82 | 70.43 | -1.30 | 0.071987589 |
| MEIS3P1 | 193.36 | 78.54 | -1.30 | 0.029420534 |
| ZWINT | 782.30 | 318.09 | -1.30 | 0.0233663 |
| KIAA0754 | 399.53 | 162.87 | -1.29 | 0.027294521 |
| CDK17 | 2056.09 | 838.74 | -1.29 | 0.015954654 |
| PRIM1 | 226.92 | 92.74 | -1.29 | 0.01803837 |
| ZGRF1 | 223.73 | 91.82 | -1.28 | 0.020940804 |
| CCND1 | 6821.52 | 2808.04 | -1.28 | 0.001526894 |
| MYO10 | 5327.36 | 2197.77 | -1.28 | 0.011269542 |
| MCM2 | 690.21 | 285.65 | -1.27 | 0.043698372 |
| FIGNL1 | 392.18 | 162.97 | -1.27 | 0.032179937 |
| FANCI | 1488.86 | 619.54 | -1.26 | 0.040120812 |
| MCM3 | 1331.77 | 554.43 | -1.26 | 0.013846788 |
| EXTL2 | 343.37 | 143.16 | -1.26 | 0.010863336 |
| FOXL1 | 398.33 | 166.25 | -1.26 | 0.0097454 |
| AKAP2 | 2097.30 | 879.23 | -1.25 | 0.084579375 |
| ARG2 | 541.11 | 226.88 | -1.25 | 0.046671662 |
| LYAR | 408.89 | 171.72 | -1.25 | 0.048492445 |
| RUNX1 | 1313.97 | 553.13 | -1.25 | 0.012033314 |
| ERN1 | 314.82 | 132.90 | -1.24 | 0.082630827 |
| C6orf1 | 398.99 | 168.78 | -1.24 | 0.012053349 |
| MCM8 | 441.40 | 186.73 | -1.24 | 0.009341962 |
| GSDMC | 258.00 | 109.28 | -1.24 | 0.02225828 |
| C12orf75 | 1715.74 | 727.02 | -1.24 | 0.002694415 |
| FHOD1 | 477.67 | 202.93 | -1.24 | 0.00910808 |
| DUSP1 | 1702.31 | 723.70 | -1.23 | 0.045718547 |
| CHEK1 | 699.76 | 298.59 | -1.23 | 0.050785203 |
| ELK3 | 1287.31 | 549.53 | -1.23 | 0.003328508 |
| SORL1 | 3995.66 | 1707.27 | -1.23 | 0.007095321 |
| CEP135 | 267.10 | 114.14 | -1.23 | 0.030891677 |
| PPP1R15A | 1843.74 | 789.34 | -1.22 | 0.025811104 |
| NAB2 | 892.30 | 382.29 | -1.22 | 0.023723406 |
| SETBP1 | 231.66 | 99.88 | -1.21 | 0.036077894 |
| SSH1 | 2918.06 | 1258.40 | -1.21 | 0.029260995 |
| STIL | 458.59 | 198.20 | -1.21 | 0.056235407 |
| NR2C2AP | 263.42 | 114.12 | -1.21 | 0.025700268 |
| FGD6 | 2820.21 | 1225.04 | -1.20 | 0.015229037 |
| CMTM7 | 713.73 | 310.64 | -1.20 | 0.00748494 |
| TUBA4A | 5045.47 | 2198.68 | -1.20 | 0.082630827 |
| HMGB3 | 2108.77 | 920.61 | -1.20 | 0.043777835 |
| STK17B | 898.44 | 392.47 | -1.19 | 0.035822451 |
| TUBA1C | 11194.98 | 4893.18 | -1.19 | 0.098093827 |
| PSMD2 | 10365.46 | 4531.86 | -1.19 | 0.002379995 |
| RECQL4 | 313.74 | 137.22 | -1.19 | 0.085139422 |
| CDCP1 | 6044.69 | 2644.34 | -1.19 | 0.022912259 |
| WDR4 | 264.51 | 115.90 | -1.19 | 0.032021286 |
| HSPA13 | 1288.65 | 566.99 | -1.18 | 0.03656605 |
| SH2B3 | 738.91 | 327.09 | -1.18 | 0.009792941 |
| SLC39A13 | 921.37 | 407.95 | -1.18 | 0.030891677 |
| GSTO1 | 2496.81 | 1105.58 | -1.18 | 0.003955824 |
| FAM86A | 202.64 | 89.91 | -1.17 | 0.05747497 |
| TEX30 | 173.42 | 77.06 | -1.17 | 0.059751403 |
| ZNF598 | 1279.63 | 569.64 | -1.17 | 0.035266111 |
| ARL13B | 285.17 | 127.09 | -1.17 | 0.035093405 |
| ARHGAP18 | 681.21 | 303.97 | -1.16 | 0.059511816 |
| MSN | 15109.08 | 6748.58 | -1.16 | 0.022046473 |
| ITGB5 | 2115.06 | 949.46 | -1.16 | 0.005403641 |
| ASAP2 | 2945.33 | 1322.62 | -1.16 | 0.014677134 |
| SP4 | 151.34 | 68.06 | -1.15 | 0.093525911 |
| SPIN4 | 137.79 | 61.97 | -1.15 | 0.098421779 |
| MTHFD1L | 1275.72 | 573.89 | -1.15 | 0.071679483 |
| RFC4 | 511.43 | 231.41 | -1.14 | 0.042555999 |
| ATAD5 | 288.89 | 131.30 | -1.14 | 0.039652734 |
| ZNF215 | 184.69 | 83.95 | -1.14 | 0.067632491 |
| TCOF1 | 1602.35 | 734.16 | -1.13 | 0.014310069 |
| ANXA2 | 106498.23 | 48813.44 | -1.13 | 0.005155098 |
| FANCA | 445.47 | 205.21 | -1.12 | 0.035690983 |
| NRIP3 | 167.99 | 77.53 | -1.12 | 0.09810801 |
| TNFRSF10A | 453.94 | 209.84 | -1.11 | 0.027058949 |
| SGTB | 366.30 | 169.39 | -1.11 | 0.086642277 |
| B4GALT6 | 226.84 | 105.10 | -1.11 | 0.058160903 |
| JUNB | 4661.79 | 2160.39 | -1.11 | 0.006858953 |
| DUSP7 | 3092.59 | 1434.40 | -1.11 | 0.008595848 |
| ASL | 600.20 | 278.80 | -1.11 | 0.020420131 |
| MDFI | 1712.54 | 796.77 | -1.10 | 0.00933774 |
| CHAF1B | 257.82 | 120.18 | -1.10 | 0.049188998 |
| CDA | 405.73 | 189.47 | -1.10 | 0.051403319 |
| DNMT1 | 1667.17 | 779.60 | -1.10 | 0.027725032 |
| FAM167A | 613.68 | 287.13 | -1.10 | 0.095673072 |
| SH3TC1 | 1390.43 | 650.99 | -1.09 | 0.014310069 |
| CD276 | 1425.81 | 668.53 | -1.09 | 0.013169252 |
| SMURF2 | 1384.64 | 650.08 | -1.09 | 0.012850411 |
| FHL2 | 1653.13 | 776.21 | -1.09 | 0.067407697 |
| BOP1 | 835.95 | 392.71 | -1.09 | 0.01622409 |
| ZFP36L2 | 6583.57 | 3105.84 | -1.08 | 0.033720062 |
| PHC2 | 2129.94 | 1004.98 | -1.08 | 0.011984469 |
| SPATS2 | 2536.76 | 1200.13 | -1.08 | 0.047683492 |
| CENPJ | 367.26 | 174.25 | -1.08 | 0.087381822 |
| KCNJ15 | 2693.23 | 1283.90 | -1.07 | 0.010777956 |
| ZWILCH | 771.80 | 368.66 | -1.07 | 0.086642277 |
| KDELR3 | 389.69 | 186.17 | -1.07 | 0.07477089 |
| WNT7A | 1706.50 | 818.10 | -1.06 | 0.069158424 |
| CD59 | 18603.05 | 8922.70 | -1.06 | 0.009188444 |
| HRH1 | 615.39 | 295.54 | -1.06 | 0.029786578 |
| COPRS | 325.71 | 156.69 | -1.06 | 0.054105383 |
| PPP1R18 | 2719.90 | 1308.64 | -1.06 | 0.012850411 |
| ZNF641 | 270.12 | 130.63 | -1.05 | 0.076417631 |
| PNP | 1797.65 | 871.26 | -1.04 | 0.069158424 |
| ARHGEF1 | 1428.87 | 693.93 | -1.04 | 0.017116998 |
| ST3GAL3 | 235.90 | 114.59 | -1.04 | 0.091808267 |
| SLC35F2 | 1045.03 | 509.10 | -1.04 | 0.021775846 |
| CRY1 | 348.84 | 170.29 | -1.03 | 0.061913459 |
| RELT | 238.48 | 116.51 | -1.03 | 0.081549305 |
| CENPO | 421.16 | 205.87 | -1.03 | 0.047572167 |
| NPM3 | 840.71 | 412.49 | -1.03 | 0.065904166 |
| LDLR | 6793.73 | 3335.96 | -1.03 | 0.028171375 |
| ANXA5 | 5231.55 | 2572.81 | -1.02 | 0.01433614 |
| PFAS | 451.56 | 222.19 | -1.02 | 0.051133909 |
| POLR3G | 277.20 | 136.43 | -1.02 | 0.076595726 |
| RFTN1 | 658.82 | 325.35 | -1.02 | 0.03656605 |
| TMC7 | 363.56 | 179.68 | -1.02 | 0.091867333 |
| TOMM34 | 1274.39 | 630.42 | -1.02 | 0.024966515 |
| ERCC1 | 1868.58 | 925.18 | -1.01 | 0.023485199 |
| FAS | 827.07 | 411.85 | -1.01 | 0.030891677 |
| ITPR1 | 352.79 | 175.73 | -1.01 | 0.067839273 |
| SNX24 | 453.27 | 225.93 | -1.00 | 0.052918507 |
| SOX7 | 870.05 | 433.90 | -1.00 | 0.037085737 |
| EHD1 | 1321.69 | 661.85 | -1.00 | 0.030472782 |
| PFN1 | 21293.08 | 10674.43 | -1.00 | 0.018068187 |
| MCL1 | 16660.62 | 8356.16 | -1.00 | 0.084659714 |
| PIK3CD | 653.07 | 327.70 | -0.99 | 0.047773071 |
| DNAJC9 | 816.13 | 409.75 | -0.99 | 0.034844075 |
| KLF6 | 5014.79 | 2520.04 | -0.99 | 0.085387641 |
| MICALCL | 581.67 | 292.34 | -0.99 | 0.051403319 |
| MCM7 | 1781.97 | 897.62 | -0.99 | 0.077702752 |
| C11orf24 | 556.27 | 280.51 | -0.99 | 0.048984662 |
| TUBB6 | 8408.33 | 4246.40 | -0.99 | 0.06515547 |
| SMYD2 | 894.57 | 452.65 | -0.98 | 0.040896973 |
| PC | 994.83 | 503.69 | -0.98 | 0.041469366 |
| TPBG | 4060.36 | 2059.74 | -0.98 | 0.051733479 |
| CLTCL1 | 372.94 | 189.30 | -0.98 | 0.083178926 |
| PHF19 | 642.39 | 327.85 | -0.97 | 0.063926868 |
| CTPS1 | 1367.21 | 698.40 | -0.97 | 0.035093405 |
| GTF2H2C_2 | 431.41 | 220.59 | -0.97 | 0.068423973 |
| CENPN | 623.09 | 318.89 | -0.97 | 0.063557049 |
| GOLIM4 | 774.86 | 397.00 | -0.96 | 0.045718547 |
| FERMT2 | 452.43 | 231.96 | -0.96 | 0.076938459 |
| RAD18 | 481.22 | 247.41 | -0.96 | 0.063926868 |
| HPRT1 | 984.92 | 507.26 | -0.96 | 0.039880231 |
| C19orf48 | 1136.19 | 585.80 | -0.96 | 0.067468667 |
| NT5C | 645.08 | 333.28 | -0.95 | 0.054947669 |
| CAD | 1300.82 | 672.18 | -0.95 | 0.039951966 |
| FARSB | 1269.91 | 658.65 | -0.95 | 0.04294334 |
| MAP4K4 | 5851.93 | 3039.97 | -0.94 | 0.047683492 |
| TUBG1 | 1302.43 | 676.71 | -0.94 | 0.040935635 |
| MIS18A | 392.63 | 204.10 | -0.94 | 0.09810801 |
| SH2D3A | 814.45 | 424.51 | -0.94 | 0.062408223 |
| MYL12A | 7577.81 | 3960.01 | -0.94 | 0.035093405 |
| S100A13 | 2049.15 | 1071.43 | -0.94 | 0.036637368 |
| NLN | 743.36 | 390.39 | -0.93 | 0.063075285 |
| NCAPD3 | 1034.45 | 544.04 | -0.93 | 0.06758632 |
| FMNL2 | 2341.63 | 1233.14 | -0.93 | 0.063075285 |
| MCFD2 | 3854.89 | 2035.63 | -0.92 | 0.067819661 |
| SLC39A14 | 1744.98 | 922.91 | -0.92 | 0.056584592 |
| KIFC3 | 1075.13 | 573.69 | -0.91 | 0.068051581 |
| EBNA1BP2 | 1921.01 | 1026.04 | -0.90 | 0.052830765 |
| ARHGAP19 | 652.64 | 348.98 | -0.90 | 0.084976078 |
| BCAR1 | 1418.64 | 761.07 | -0.90 | 0.069082045 |
| SLC16A3 | 8018.35 | 4303.14 | -0.90 | 0.08639735 |
| UCK2 | 1479.61 | 794.09 | -0.90 | 0.059725226 |
| IPO4 | 598.31 | 321.49 | -0.90 | 0.097308183 |
| CDCA4 | 859.49 | 463.61 | -0.89 | 0.078643624 |
| AIDA | 2817.81 | 1521.76 | -0.89 | 0.052918507 |
| TSTA3 | 850.21 | 462.34 | -0.88 | 0.090760091 |
| HMGA1 | 9002.33 | 4895.95 | -0.88 | 0.063275757 |
| PROCR | 818.11 | 445.71 | -0.88 | 0.082630827 |
| PAICS | 3869.15 | 2115.11 | -0.87 | 0.054168822 |
| ELL2 | 5290.83 | 2893.23 | -0.87 | 0.061332978 |
| CAP1 | 17991.88 | 9842.52 | -0.87 | 0.057973705 |
| MST1R | 960.34 | 526.25 | -0.87 | 0.09142825 |
| BCAP29 | 1277.04 | 700.44 | -0.87 | 0.078643624 |
| TMSB10 | 43573.00 | 23940.66 | -0.86 | 0.054123275 |
| DRAP1 | 2490.69 | 1371.91 | -0.86 | 0.067839273 |
| ACY1 | 879.79 | 485.77 | -0.86 | 0.0948618 |
| KBTBD2 | 914.59 | 506.14 | -0.85 | 0.099794209 |
| HAT1 | 1141.86 | 632.57 | -0.85 | 0.084640059 |
| ATAD2 | 1183.46 | 656.07 | -0.85 | 0.084778643 |
| CLTB | 2578.61 | 1437.80 | -0.84 | 0.082630827 |
| ACTL6A | 1342.67 | 750.82 | -0.84 | 0.098420347 |
| ABL2 | 1259.42 | 704.84 | -0.84 | 0.099357853 |
| RANBP1 | 2015.65 | 1133.00 | -0.83 | 0.082630827 |
| TGFB1 | 1851.64 | 1041.39 | -0.83 | 0.092693902 |
| CLIC1 | 7225.18 | 4087.11 | -0.82 | 0.084640059 |
| FUS | 5253.41 | 3016.44 | -0.80 | 0.093213282 |
| KRT17 | 84253.29 | 144533.40 | 0.78 | 0.098093827 |
| PERP | 21477.72 | 36971.29 | 0.78 | 0.098421779 |
| GPC1 | 5734.68 | 10037.68 | 0.81 | 0.079527711 |
| DTX3L | 997.18 | 1751.48 | 0.81 | 0.090760091 |
| CLSTN3 | 1039.39 | 1828.68 | 0.82 | 0.084832937 |
| SLC25A29 | 441.35 | 784.85 | 0.83 | 0.098421779 |
| SEMA3F | 3478.67 | 6189.87 | 0.83 | 0.072593384 |
| TSPAN14 | 4676.76 | 8349.73 | 0.84 | 0.062255183 |
| LYN | 519.47 | 929.51 | 0.84 | 0.094507684 |
| FBLN1 | 1616.14 | 2895.32 | 0.84 | 0.068351585 |
| ULK1 | 731.35 | 1311.33 | 0.84 | 0.076611576 |
| DUSP16 | 622.02 | 1120.70 | 0.85 | 0.078131549 |
| PVRL1 | 7081.44 | 12797.70 | 0.85 | 0.063075285 |
| LRP1 | 3272.69 | 5917.73 | 0.85 | 0.099357853 |
| ZNF436 | 663.96 | 1202.63 | 0.86 | 0.072769003 |
| PWWP2B | 311.26 | 564.80 | 0.86 | 0.099986647 |
| ZNFX1 | 1325.26 | 2405.47 | 0.86 | 0.051122773 |
| BLVRB | 716.02 | 1300.84 | 0.86 | 0.069659149 |
| SMAD3 | 3113.20 | 5656.99 | 0.86 | 0.047605353 |
| SH3BP2 | 1336.44 | 2429.02 | 0.86 | 0.052918507 |
| TKT | 10181.09 | 18636.05 | 0.87 | 0.045718547 |
| TMEM47 | 411.74 | 754.23 | 0.87 | 0.084640059 |
| NCOA2 | 502.61 | 920.97 | 0.87 | 0.069158424 |
| SLC12A6 | 643.79 | 1180.24 | 0.87 | 0.064987117 |
| TOM1L2 | 1400.62 | 2569.06 | 0.88 | 0.05281765 |
| ANXA11 | 2100.58 | 3854.44 | 0.88 | 0.044707444 |
| ZBTB44 | 1203.49 | 2208.92 | 0.88 | 0.048949067 |
| SREBF1 | 2451.79 | 4501.81 | 0.88 | 0.051083081 |
| PCCA | 325.10 | 597.08 | 0.88 | 0.095602302 |
| ABCA12 | 322.38 | 593.16 | 0.88 | 0.0948618 |
| RBM47 | 679.42 | 1261.73 | 0.89 | 0.050785203 |
| GLTP | 1041.57 | 1934.48 | 0.89 | 0.047088981 |
| STK38 | 955.28 | 1777.59 | 0.90 | 0.04294334 |
| MDK | 730.47 | 1362.30 | 0.90 | 0.048025347 |
| SGSH | 203.26 | 379.09 | 0.90 | 0.097308183 |
| TRIM22 | 2353.78 | 4393.69 | 0.90 | 0.039652734 |
| RASSF6 | 1544.80 | 2887.10 | 0.90 | 0.038088128 |
| SETD1B | 476.17 | 891.18 | 0.90 | 0.051403319 |
| TOB1 | 1080.60 | 2022.97 | 0.90 | 0.044295804 |
| PDCD4 | 2344.71 | 4389.89 | 0.90 | 0.051133909 |
| SH3BGRL2 | 279.40 | 523.44 | 0.91 | 0.081711267 |
| NFKBIA | 1922.97 | 3603.89 | 0.91 | 0.084579375 |
| CLN3 | 334.91 | 629.29 | 0.91 | 0.063926868 |
| YPEL2 | 285.52 | 537.47 | 0.91 | 0.070456719 |
| PARP10 | 415.01 | 781.41 | 0.91 | 0.059161184 |
| NOTCH1 | 7067.79 | 13328.07 | 0.92 | 0.029118066 |
| P2RY2 | 728.99 | 1375.49 | 0.92 | 0.051133909 |
| KRT16 | 3177.26 | 6010.92 | 0.92 | 0.084729488 |
| PRCP | 876.60 | 1658.45 | 0.92 | 0.08639735 |
| ADCY6 | 1538.55 | 2921.46 | 0.93 | 0.032576791 |
| PTGR1 | 1256.93 | 2389.47 | 0.93 | 0.036498077 |
| MCCC1 | 389.35 | 742.40 | 0.93 | 0.051403319 |
| ACADSB | 367.77 | 703.62 | 0.94 | 0.052918507 |
| PSMB9 | 266.65 | 510.39 | 0.94 | 0.066921817 |
| AMFR | 1190.12 | 2279.08 | 0.94 | 0.027079482 |
| B4GALT5 | 1307.87 | 2505.16 | 0.94 | 0.032436758 |
| PPAP2A | 272.82 | 523.79 | 0.94 | 0.060456407 |
| CBX6 | 371.83 | 715.62 | 0.94 | 0.040935635 |
| TAPBP | 1926.77 | 3730.66 | 0.95 | 0.060207274 |
| RBPMS | 620.76 | 1202.93 | 0.95 | 0.032436758 |
| PLXNB2 | 6734.18 | 13065.38 | 0.96 | 0.018377053 |
| MAPK10 | 165.70 | 321.82 | 0.96 | 0.084302717 |
| RXRA | 2561.06 | 5008.02 | 0.97 | 0.020748773 |
| MGST1 | 3110.97 | 6090.54 | 0.97 | 0.0233663 |
| ENTPD3 | 467.91 | 916.19 | 0.97 | 0.035620747 |
| TMEM229B | 260.38 | 511.24 | 0.97 | 0.068553563 |
| ECHDC3 | 221.66 | 435.98 | 0.98 | 0.067819661 |
| LYPD3 | 1972.04 | 3880.08 | 0.98 | 0.021141653 |
| HOXB6 | 766.50 | 1511.39 | 0.98 | 0.022912259 |
| RDH10 | 228.23 | 451.37 | 0.98 | 0.052758222 |
| LPCAT3 | 798.51 | 1579.61 | 0.98 | 0.024642974 |
| DTX2 | 596.47 | 1181.06 | 0.99 | 0.023342292 |
| MYOF | 8934.67 | 17723.98 | 0.99 | 0.093213282 |
| FAM13A | 366.50 | 728.76 | 0.99 | 0.035093405 |
| NEDD4L | 1477.29 | 2939.12 | 0.99 | 0.014705041 |
| HOXD3 | 121.58 | 242.32 | 0.99 | 0.086632808 |
| C9orf3 | 157.98 | 314.89 | 1.00 | 0.067119093 |
| REPIN1 | 527.11 | 1051.07 | 1.00 | 0.022915172 |
| HIST1H1C | 156.04 | 311.22 | 1.00 | 0.06827842 |
| PLCD3 | 1935.87 | 3867.62 | 1.00 | 0.015058184 |
| ETNK2 | 394.00 | 787.73 | 1.00 | 0.027079482 |
| ZNF737 | 240.71 | 483.06 | 1.00 | 0.039880231 |
| CNDP2 | 2140.37 | 4296.72 | 1.01 | 0.012796739 |
| THSD1 | 639.99 | 1284.85 | 1.01 | 0.077702752 |
| MITF | 177.47 | 356.84 | 1.01 | 0.059095234 |
| TMEM140 | 214.59 | 431.84 | 1.01 | 0.039635423 |
| DNAJA4 | 372.50 | 752.27 | 1.01 | 0.025673483 |
| ERMP1 | 2821.72 | 5705.45 | 1.02 | 0.014677134 |
| IFI30 | 279.67 | 566.77 | 1.02 | 0.035592061 |
| RAB11FIP4 | 634.40 | 1286.25 | 1.02 | 0.072931987 |
| MSRB1 | 268.42 | 545.45 | 1.02 | 0.06308247 |
| PNRC1 | 667.93 | 1359.67 | 1.03 | 0.018171458 |
| MTRNR2L2 | 33589.43 | 68391.30 | 1.03 | 0.04884171 |
| FAT2 | 8158.16 | 16655.86 | 1.03 | 0.084640059 |
| LPAR6 | 353.03 | 721.28 | 1.03 | 0.026870371 |
| FAM46A | 122.48 | 251.04 | 1.04 | 0.073136934 |
| PITX1 | 378.59 | 779.67 | 1.04 | 0.093405541 |
| SLCO3A1 | 577.97 | 1192.08 | 1.04 | 0.037879805 |
| PSMB8 | 607.36 | 1252.99 | 1.04 | 0.014653645 |
| RUNX2 | 491.10 | 1013.53 | 1.05 | 0.029961337 |
| PLD1 | 382.36 | 789.32 | 1.05 | 0.04294334 |
| ANK3 | 472.63 | 977.81 | 1.05 | 0.014310069 |
| MAL2 | 1725.79 | 3573.68 | 1.05 | 0.008624004 |
| INPPL1 | 1735.14 | 3602.78 | 1.05 | 0.062408223 |
| KIAA1522 | 3941.03 | 8214.75 | 1.06 | 0.022447713 |
| SAMD9 | 644.08 | 1343.19 | 1.06 | 0.02642696 |
| EFNA5 | 177.98 | 371.43 | 1.06 | 0.059088502 |
| SPTBN2 | 500.88 | 1045.50 | 1.06 | 0.046671662 |
| SERINC5 | 1075.33 | 2245.37 | 1.06 | 0.009791791 |
| CKB | 430.52 | 899.36 | 1.06 | 0.081736369 |
| IFIH1 | 383.82 | 804.20 | 1.07 | 0.018152561 |
| PLIN2 | 289.54 | 606.70 | 1.07 | 0.081204016 |
| METRNL | 136.89 | 287.66 | 1.07 | 0.042555999 |
| UNC13D | 277.66 | 585.18 | 1.08 | 0.020606575 |
| SLC25A23 | 526.98 | 1110.75 | 1.08 | 0.013824903 |
| GM2A | 4498.99 | 9492.58 | 1.08 | 0.010363641 |
| CSTB | 4370.02 | 9221.99 | 1.08 | 0.007095321 |
| MALAT1 | 10828.86 | 22931.00 | 1.08 | 0.043777835 |
| C1orf213 | 110.00 | 233.76 | 1.09 | 0.075874563 |
| KLHL24 | 420.46 | 896.04 | 1.09 | 0.063075285 |
| MTRNR2L8 | 17835.60 | 38016.34 | 1.09 | 0.092697008 |
| RNF152 | 99.38 | 211.87 | 1.09 | 0.059885003 |
| LOC440434 | 264.95 | 566.86 | 1.10 | 0.015022737 |
| NBL1 | 97.49 | 208.79 | 1.10 | 0.059885003 |
| RAB27B | 571.22 | 1224.09 | 1.10 | 0.008673717 |
| ETV6 | 503.31 | 1080.56 | 1.10 | 0.024615676 |
| GLCCI1 | 144.24 | 309.87 | 1.10 | 0.032436758 |
| PNPLA2 | 665.75 | 1433.52 | 1.11 | 0.006703945 |
| CDR2L | 510.67 | 1101.04 | 1.11 | 0.009852296 |
| CD24 | 11504.58 | 24844.77 | 1.11 | 0.004097305 |
| TRIM16 | 819.53 | 1777.21 | 1.12 | 0.090260791 |
| PARP14 | 1535.05 | 3352.04 | 1.13 | 0.004266789 |
| PPL | 1472.03 | 3222.31 | 1.13 | 0.003946845 |
| CCDC80 | 790.92 | 1736.68 | 1.13 | 0.021484543 |
| XG | 143.98 | 316.33 | 1.14 | 0.067119093 |
| HDHD3 | 153.70 | 338.36 | 1.14 | 0.02263651 |
| STS | 134.88 | 297.00 | 1.14 | 0.035690983 |
| EVPL | 961.40 | 2118.34 | 1.14 | 0.014653645 |
| SCPEP1 | 853.29 | 1880.35 | 1.14 | 0.004494159 |
| DENND2D | 156.27 | 345.35 | 1.14 | 0.039880231 |
| ACOX1 | 951.48 | 2102.89 | 1.14 | 0.005058566 |
| NPEPPS | 2471.97 | 5487.81 | 1.15 | 0.002826513 |
| PRKCD | 901.33 | 2001.52 | 1.15 | 0.008325157 |
| PLIN4 | 401.85 | 897.16 | 1.16 | 0.005395708 |
| TP53INP1 | 1136.69 | 2538.21 | 1.16 | 0.003964479 |
| MTRNR2L1 | 4589.57 | 10263.85 | 1.16 | 0.02263651 |
| GABARAPL1 | 908.67 | 2034.66 | 1.16 | 0.003316859 |
| CCDC146 | 95.95 | 215.16 | 1.17 | 0.040082501 |
| VAV3 | 662.72 | 1489.78 | 1.17 | 0.021775846 |
| SQSTM1 | 4902.60 | 11021.88 | 1.17 | 0.006935262 |
| MTRNR2L10 | 1091.07 | 2454.19 | 1.17 | 0.072769003 |
| APOBR | 93.42 | 210.48 | 1.17 | 0.054168822 |
| B3GALT4 | 79.14 | 178.55 | 1.17 | 0.047704748 |
| EFNB3 | 124.24 | 281.08 | 1.18 | 0.0233663 |
| ATP9A | 656.61 | 1485.89 | 1.18 | 0.034616652 |
| PRRG4 | 510.48 | 1155.85 | 1.18 | 0.096071705 |
| COBLL1 | 1434.70 | 3260.99 | 1.18 | 0.051122773 |
| POR | 751.67 | 1712.34 | 1.19 | 0.002610767 |
| GABRE | 263.20 | 601.50 | 1.19 | 0.008254288 |
| HOXD9 | 103.37 | 236.42 | 1.19 | 0.024642974 |
| ACSF2 | 969.08 | 2218.06 | 1.19 | 0.009948845 |
| PUS10 | 106.20 | 243.84 | 1.20 | 0.024642974 |
| UNC93B1 | 360.23 | 831.71 | 1.21 | 0.008867801 |
| CCNJL | 377.48 | 873.33 | 1.21 | 0.010233354 |
| CHKA | 218.85 | 506.53 | 1.21 | 0.016850901 |
| B3GNT3 | 261.24 | 605.21 | 1.21 | 0.025700268 |
| A4GALT | 719.76 | 1668.22 | 1.21 | 0.00204603 |
| PGPEP1 | 263.22 | 611.75 | 1.22 | 0.00449039 |
| BPGM | 537.77 | 1251.43 | 1.22 | 0.009504508 |
| LRMP | 168.14 | 395.28 | 1.23 | 0.007769688 |
| PDLIM1 | 7378.12 | 17355.03 | 1.23 | 0.00236131 |
| RALGAPA2 | 461.10 | 1086.13 | 1.24 | 0.067119093 |
| HOXB-AS3 | 78.98 | 186.09 | 1.24 | 0.028900301 |
| TNFRSF21 | 3316.51 | 7847.36 | 1.24 | 0.002552287 |
| SLC22A5 | 266.61 | 631.24 | 1.24 | 0.003415075 |
| GALNT12 | 108.86 | 257.87 | 1.24 | 0.045712824 |
| HOXB4 | 269.00 | 638.88 | 1.25 | 0.003780583 |
| LAMP3 | 83.55 | 198.65 | 1.25 | 0.047605353 |
| ARHGAP23 | 2560.47 | 6092.29 | 1.25 | 0.029260995 |
| FAM83H-AS1 | 126.31 | 301.48 | 1.26 | 0.036305286 |
| SDCBP2 | 325.88 | 778.23 | 1.26 | 0.084640059 |
| FRAT2 | 132.54 | 317.99 | 1.26 | 0.0097454 |
| FOSL2 | 2353.14 | 5648.91 | 1.26 | 0.000691961 |
| DUOX1 | 1082.34 | 2610.90 | 1.27 | 0.001249372 |
| AIFM3 | 114.32 | 275.97 | 1.27 | 0.01188421 |
| TRAFD1 | 831.42 | 2012.06 | 1.28 | 0.00625341 |
| GRHL1 | 721.50 | 1749.25 | 1.28 | 0.06515547 |
| LNX1 | 72.63 | 176.14 | 1.28 | 0.033776219 |
| MIRLET7BHG | 113.85 | 277.54 | 1.29 | 0.012529445 |
| MEIS1 | 113.29 | 276.30 | 1.29 | 0.010953046 |
| BTN3A3 | 280.97 | 685.36 | 1.29 | 0.008867801 |
| RARG | 649.14 | 1584.74 | 1.29 | 0.077221798 |
| GATSL3 | 79.84 | 194.94 | 1.29 | 0.022353405 |
| HOXB5 | 225.38 | 551.77 | 1.29 | 0.002322606 |
| EVA1C | 60.53 | 148.19 | 1.29 | 0.07848959 |
| PIM3 | 777.36 | 1904.10 | 1.29 | 0.001203515 |
| PAQR7 | 1145.28 | 2840.41 | 1.31 | 0.050785203 |
| ERBB3 | 413.11 | 1026.72 | 1.31 | 0.018215334 |
| NOTCH3 | 1332.83 | 3313.59 | 1.31 | 0.035364354 |
| TLCD1 | 179.50 | 448.96 | 1.32 | 0.006817023 |
| TRIM29 | 3401.79 | 8636.86 | 1.34 | 0.024615676 |
| ATP1B1 | 1413.91 | 3605.11 | 1.35 | 0.045501327 |
| CAPN14 | 104.55 | 267.49 | 1.36 | 0.007157848 |
| UBA7 | 521.41 | 1334.80 | 1.36 | 0.005245244 |
| AKR1B1 | 2122.66 | 5452.28 | 1.36 | 0.000456801 |
| ZNF552 | 196.88 | 510.20 | 1.37 | 0.001520799 |
| CPA4 | 123.23 | 319.44 | 1.37 | 0.038755714 |
| SSPN | 135.27 | 350.83 | 1.37 | 0.040082501 |
| SOD2 | 1174.60 | 3053.79 | 1.38 | 0.01241895 |
| RRAGD | 46.85 | 121.84 | 1.38 | 0.084640059 |
| RHOU | 52.90 | 138.18 | 1.39 | 0.062111571 |
| NPTXR | 80.34 | 209.92 | 1.39 | 0.068051581 |
| ADM | 594.99 | 1557.70 | 1.39 | 0.010966992 |
| TTC9 | 258.73 | 683.05 | 1.40 | 0.00046953 |
| STARD13 | 304.62 | 804.89 | 1.40 | 0.091867333 |
| TNFSF10 | 1089.41 | 2879.72 | 1.40 | 0.027725032 |
| USP6 | 33.93 | 89.76 | 1.40 | 0.060883647 |
| MACROD2 | 39.83 | 105.38 | 1.40 | 0.047773071 |
| GOLGA6L5P | 56.48 | 149.88 | 1.41 | 0.040718439 |
| GATA3 | 445.85 | 1183.34 | 1.41 | 0.000238643 |
| HES2 | 2936.43 | 7801.96 | 1.41 | 0.0233663 |
| MECOM | 502.32 | 1338.80 | 1.41 | 0.013211534 |
| SYNGR1 | 90.59 | 242.76 | 1.42 | 0.004879494 |
| DBP | 70.82 | 189.79 | 1.42 | 0.008751712 |
| APOL6 | 985.47 | 2656.09 | 1.43 | 0.003420006 |
| DGAT2 | 344.37 | 928.72 | 1.43 | 0.001726015 |
| RASA4 | 104.92 | 282.96 | 1.43 | 0.040935635 |
| SARM1 | 25.89 | 69.86 | 1.43 | 0.09276361 |
| IL20RA | 104.46 | 283.33 | 1.44 | 0.005338986 |
| INPP1 | 523.97 | 1427.58 | 1.45 | 0.000172015 |
| ZSCAN31 | 92.20 | 251.66 | 1.45 | 0.007698367 |
| GSR | 862.16 | 2358.26 | 1.45 | 0.001340091 |
| KLHL31 | 44.03 | 120.46 | 1.45 | 0.088602714 |
| GPRC5C | 360.90 | 987.85 | 1.45 | 0.047431877 |
| PPP1R12B | 463.16 | 1269.86 | 1.46 | 0.000741122 |
| PPFIBP2 | 154.75 | 424.50 | 1.46 | 0.021520842 |
| FRAT1 | 32.12 | 88.17 | 1.46 | 0.072951049 |
| TMEM51-AS1 | 118.55 | 325.97 | 1.46 | 0.00625341 |
| LINC00673 | 434.83 | 1196.13 | 1.46 | 9.86E-05 |
| SIDT1 | 28.46 | 78.62 | 1.47 | 0.063926868 |
| CARD14 | 34.17 | 94.66 | 1.47 | 0.040891151 |
| TINCR | 69.60 | 194.34 | 1.48 | 0.015669017 |
| BNIPL | 28.90 | 80.76 | 1.48 | 0.05634311 |
| TP53INP2 | 253.51 | 709.89 | 1.49 | 0.001249234 |
| SLC15A2 | 81.37 | 228.19 | 1.49 | 0.009873717 |
| ALDH1A3 | 5709.30 | 16015.06 | 1.49 | 0.009410887 |
| IL9R | 33.64 | 94.68 | 1.49 | 0.035364354 |
| SEPP1 | 174.91 | 492.39 | 1.49 | 0.012517837 |
| DUOXA1 | 366.13 | 1030.79 | 1.49 | 0.001650748 |
| TBX3 | 1425.39 | 4019.12 | 1.50 | 1.65E-05 |
| RHOF | 57.05 | 161.31 | 1.50 | 0.007859071 |
| CDKN2B | 428.37 | 1213.28 | 1.50 | 5.47E-05 |
| UPK3BL | 441.52 | 1252.06 | 1.50 | 0.000479658 |
| KLHDC8B | 693.02 | 1966.00 | 1.50 | 0.020748773 |
| ABHD4 | 357.86 | 1017.21 | 1.51 | 0.013832476 |
| NFIX | 87.94 | 250.65 | 1.51 | 0.005188561 |
| PRSS22 | 461.58 | 1316.16 | 1.51 | 0.016742308 |
| GAREM | 196.95 | 562.20 | 1.51 | 0.000164662 |
| CTSH | 1702.07 | 4860.04 | 1.51 | 1.35E-05 |
| SYT17 | 21.91 | 62.70 | 1.52 | 0.0948618 |
| NBPF20 | 376.60 | 1078.62 | 1.52 | 0.006413876 |
| PCP4L1 | 22.02 | 63.44 | 1.53 | 0.089823727 |
| CDKN1C | 131.13 | 377.85 | 1.53 | 0.000664235 |
| CRABP2 | 1603.24 | 4628.30 | 1.53 | 0.040935635 |
| HSD17B14 | 39.54 | 114.27 | 1.53 | 0.047044667 |
| PVRL4 | 519.31 | 1501.83 | 1.53 | 0.000325215 |
| CITED2 | 277.23 | 802.11 | 1.53 | 0.022912259 |
| ARHGEF10L | 328.19 | 953.60 | 1.54 | 0.001549021 |
| RNF213 | 5403.23 | 15742.96 | 1.54 | 9.86E-05 |
| PPARGC1B | 144.65 | 422.31 | 1.55 | 0.000323094 |
| TSC22D3 | 1594.86 | 4686.75 | 1.56 | 0.007002574 |
| FOXA1 | 515.99 | 1528.28 | 1.57 | 0.008382718 |
| OCLN | 110.71 | 329.01 | 1.57 | 0.035822451 |
| HOXB8 | 155.22 | 463.87 | 1.58 | 0.000148182 |
| PIK3R3 | 333.85 | 997.91 | 1.58 | 0.000118255 |
| STEAP4 | 39.55 | 118.38 | 1.58 | 0.03876416 |
| EPHX1 | 308.35 | 927.48 | 1.59 | 8.49E-05 |
| CCDC88B | 169.08 | 512.70 | 1.60 | 0.000413893 |
| RASEF | 146.57 | 444.74 | 1.60 | 0.074853819 |
| KIAA0513 | 247.58 | 752.64 | 1.60 | 0.014653645 |
| ATP6V0E2 | 73.18 | 224.07 | 1.61 | 0.001051904 |
| BCL6 | 223.81 | 685.41 | 1.61 | 0.001409517 |
| CCDC69 | 227.37 | 696.58 | 1.62 | 0.001125793 |
| NEBL | 108.61 | 334.03 | 1.62 | 0.016666248 |
| ALDH2 | 600.97 | 1851.10 | 1.62 | 4.86E-06 |
| FAM84A | 599.76 | 1855.36 | 1.63 | 1.55E-05 |
| TLCD2 | 218.08 | 676.68 | 1.63 | 0.003912116 |
| C10orf54 | 329.86 | 1024.87 | 1.64 | 0.02509487 |
| TIMP3 | 739.75 | 2298.73 | 1.64 | 0.007937897 |
| BCL2L11 | 214.87 | 668.61 | 1.64 | 0.013701868 |
| PALM | 17.25 | 54.18 | 1.65 | 0.083113842 |
| LOC284837 | 112.99 | 354.98 | 1.65 | 0.01268886 |
| CXCL17 | 46.82 | 147.35 | 1.65 | 0.04884171 |
| ACSL5 | 210.12 | 661.97 | 1.66 | 0.000385768 |
| UGT1A6 | 2462.28 | 7767.57 | 1.66 | 1.11E-06 |
| TAPBPL | 117.59 | 371.50 | 1.66 | 0.005806448 |
| LOC653602 | 96.39 | 306.06 | 1.67 | 0.032692026 |
| CNGA1 | 94.93 | 302.14 | 1.67 | 0.009324854 |
| BAG1 | 580.29 | 1847.59 | 1.67 | 0.008913338 |
| CEACAM7 | 18.99 | 60.50 | 1.67 | 0.062408223 |
| PLA2G4E | 47.53 | 151.62 | 1.67 | 0.011903165 |
| CTSD | 5108.73 | 16331.34 | 1.68 | 0.024732292 |
| HS6ST1 | 1160.64 | 3712.74 | 1.68 | 0.001684285 |
| ACER2 | 194.65 | 623.03 | 1.68 | 0.000216899 |
| ZFHX2 | 19.30 | 61.87 | 1.68 | 0.085387641 |
| APOL2 | 1367.87 | 4407.24 | 1.69 | 0.012033314 |
| THEM6 | 324.77 | 1051.91 | 1.70 | 0.003307661 |
| PSMB10 | 287.01 | 931.74 | 1.70 | 3.43E-06 |
| CYB561A3 | 1012.12 | 3301.57 | 1.71 | 7.04E-07 |
| CLDN4 | 452.48 | 1477.47 | 1.71 | 0.004169551 |
| TACSTD2 | 9203.33 | 30102.87 | 1.71 | 0.032436758 |
| RGS16 | 21.95 | 72.11 | 1.72 | 0.040808498 |
| RNF207 | 131.79 | 434.03 | 1.72 | 2.97E-05 |
| HSPA1B | 919.51 | 3030.62 | 1.72 | 0.00739202 |
| PIR | 276.89 | 919.72 | 1.73 | 3.33E-06 |
| ETV7 | 24.38 | 81.22 | 1.74 | 0.02178134 |
| MAOA | 1772.19 | 5922.31 | 1.74 | 0.020868715 |
| SYTL2 | 95.99 | 320.88 | 1.74 | 0.057649551 |
| C5orf56 | 25.90 | 86.88 | 1.75 | 0.012977296 |
| DAPK1 | 64.30 | 216.80 | 1.75 | 0.000304918 |
| NTN4 | 957.82 | 3236.19 | 1.76 | 0.018941689 |
| SGPP2 | 47.36 | 160.44 | 1.76 | 0.00508749 |
| C6orf132 | 709.83 | 2406.47 | 1.76 | 1.36E-05 |
| LXN | 149.08 | 505.55 | 1.76 | 3.06E-05 |
| ACKR2 | 27.89 | 95.03 | 1.77 | 0.024615676 |
| GRHL3 | 354.04 | 1209.46 | 1.77 | 6.57E-07 |
| MAP3K8 | 62.54 | 213.69 | 1.77 | 0.023896431 |
| ACKR4 | 35.40 | 121.43 | 1.78 | 0.002826613 |
| LINC01279 | 31.51 | 108.42 | 1.78 | 0.010777956 |
| TMEM184A | 295.23 | 1019.15 | 1.79 | 0.004879494 |
| AKR1C3 | 1063.05 | 3685.09 | 1.79 | 6.65E-06 |
| NRG2 | 15.92 | 55.35 | 1.80 | 0.090166283 |
| FGD3 | 61.33 | 215.31 | 1.81 | 0.044436094 |
| NDRG2 | 241.85 | 849.45 | 1.81 | 0.027306283 |
| TRIM16L | 200.22 | 707.64 | 1.82 | 0.047128604 |
| CLU | 176.33 | 625.56 | 1.83 | 0.020748773 |
| ACSL1 | 943.07 | 3370.25 | 1.84 | 6.31E-05 |
| BHLHE41 | 764.77 | 2745.48 | 1.84 | 0.004031153 |
| GPR160 | 22.56 | 81.00 | 1.84 | 0.012486324 |
| IFIT3 | 312.24 | 1123.13 | 1.85 | 0.012486324 |
| TLR3 | 181.58 | 657.15 | 1.86 | 0.013310241 |
| ZSCAN16 | 26.08 | 94.59 | 1.86 | 0.005514405 |
| IMPA2 | 100.26 | 365.00 | 1.86 | 0.00684694 |
| PBX1 | 369.58 | 1358.93 | 1.88 | 7.25E-08 |
| SRRM3 | 20.57 | 76.46 | 1.89 | 0.012486324 |
| TTC39A | 30.89 | 115.45 | 1.90 | 0.014478431 |
| DTX4 | 953.21 | 3593.87 | 1.91 | 0.007037735 |
| ASS1 | 609.36 | 2312.36 | 1.92 | 0.026269544 |
| PRSS27 | 34.56 | 132.19 | 1.94 | 0.000714596 |
| SMIM5 | 27.64 | 106.36 | 1.94 | 0.009383858 |
| ASAP3 | 257.43 | 994.45 | 1.95 | 0.000388855 |
| PIK3C2B | 218.12 | 844.46 | 1.95 | 0.001394096 |
| SRGAP3 | 488.40 | 1891.38 | 1.95 | 0.001249372 |
| HSPB1 | 11154.17 | 43249.61 | 1.96 | 0.000613979 |
| SPIRE2 | 14.81 | 57.51 | 1.96 | 0.024120798 |
| ALPK3 | 12.72 | 49.40 | 1.96 | 0.035780951 |
| MERTK | 17.98 | 70.01 | 1.96 | 0.011727093 |
| SLC16A7 | 77.92 | 304.40 | 1.97 | 0.040740558 |
| OVCH2 | 16.95 | 66.39 | 1.97 | 0.014031564 |
| ACSM3 | 33.36 | 130.87 | 1.97 | 0.002867772 |
| SERPINB13 | 177.84 | 699.99 | 1.98 | 0.014655852 |
| RNF208 | 9.34 | 36.89 | 1.98 | 0.094507684 |
| LIPC | 23.06 | 91.39 | 1.99 | 0.013310241 |
| UCP2 | 652.64 | 2603.63 | 2.00 | 0.092067188 |
| APOL4 | 163.77 | 653.66 | 2.00 | 0.006817023 |
| FABP5 | 1559.05 | 6241.52 | 2.00 | 3.41E-05 |
| RALGPS1 | 47.68 | 191.05 | 2.00 | 0.002675144 |
| BMF | 29.54 | 119.43 | 2.02 | 0.047088981 |
| UGT1A1 | 29.50 | 119.57 | 2.02 | 0.00095651 |
| NHLH2 | 21.78 | 88.44 | 2.02 | 0.003627836 |
| MXRA5 | 1148.45 | 4671.57 | 2.02 | 0.004062503 |
| CXXC4 | 14.91 | 60.97 | 2.03 | 0.029576775 |
| GBP4 | 178.73 | 735.63 | 2.04 | 0.000374719 |
| SLC15A1 | 18.46 | 76.19 | 2.04 | 0.027422239 |
| FOXC1 | 154.91 | 640.10 | 2.05 | 7.04E-07 |
| GPRC5A | 1059.18 | 4410.66 | 2.06 | 0.000493437 |
| ANKRD35 | 25.47 | 106.35 | 2.06 | 0.013310241 |
| GLUL | 6208.52 | 26014.39 | 2.07 | 7.58E-10 |
| PHYHIP | 30.54 | 129.00 | 2.08 | 0.01241895 |
| PLEKHG6 | 138.48 | 587.63 | 2.09 | 0.012053349 |
| LOC100128770 | 24.54 | 104.15 | 2.09 | 0.007242033 |
| CCDC64B | 83.54 | 356.04 | 2.09 | 0.005055348 |
| ERICH5 | 13.20 | 56.29 | 2.09 | 0.024615676 |
| GPR110 | 482.17 | 2061.44 | 2.10 | 3.79E-09 |
| PRR15 | 11.84 | 50.68 | 2.10 | 0.023485199 |
| SELENBP1 | 103.27 | 443.41 | 2.10 | 0.001549021 |
| SOWAHB | 77.32 | 333.17 | 2.11 | 0.013227675 |
| EFCAB3 | 11.69 | 50.95 | 2.12 | 0.021520842 |
| RHBG | 12.89 | 56.46 | 2.13 | 0.015022737 |
| FAM46B | 83.50 | 367.99 | 2.14 | 0.012486324 |
| C1S | 50.35 | 222.92 | 2.15 | 0.012009641 |
| BCAS1 | 29.42 | 131.03 | 2.15 | 0.076045514 |
| LOC100507642 | 16.58 | 73.89 | 2.16 | 0.003880804 |
| ZBTB7C | 21.19 | 94.75 | 2.16 | 0.035670573 |
| SCNN1A | 552.06 | 2472.32 | 2.16 | 0.005589755 |
| GPLD1 | 22.42 | 100.70 | 2.17 | 0.003140518 |
| FAM83C | 15.51 | 70.06 | 2.18 | 0.011903165 |
| KRT15 | 835.81 | 3792.33 | 2.18 | 0.015058184 |
| RPTN | 8.67 | 39.76 | 2.20 | 0.074853819 |
| RHCG | 728.09 | 3366.87 | 2.21 | 0.004148802 |
| BCL2L14 | 12.62 | 59.28 | 2.23 | 0.081760701 |
| RBBP8NL | 15.69 | 73.92 | 2.24 | 0.002238541 |
| ALDH3B2 | 108.24 | 510.67 | 2.24 | 1.05E-07 |
| ZNF467 | 23.31 | 110.50 | 2.25 | 0.000177191 |
| APOL3 | 130.85 | 621.92 | 2.25 | 0.001243851 |
| APOL1 | 7454.13 | 35669.70 | 2.26 | 6.24E-11 |
| TMPRSS13 | 24.99 | 119.86 | 2.26 | 0.078643624 |
| RYR1 | 13.13 | 63.30 | 2.27 | 0.047572167 |
| SLC44A4 | 141.42 | 684.14 | 2.27 | 0.047795118 |
| VGLL1 | 122.65 | 594.84 | 2.28 | 0.085484972 |
| C11orf96 | 9.17 | 44.88 | 2.29 | 0.082630827 |
| RAB15 | 72.53 | 355.50 | 2.29 | 0.000802717 |
| DNM1P46 | 186.07 | 917.53 | 2.30 | 0.025149554 |
| RHOV | 236.77 | 1169.98 | 2.30 | 2.21E-09 |
| IFIT2 | 85.36 | 422.43 | 2.31 | 0.002292076 |
| SECTM1 | 49.03 | 243.86 | 2.31 | 0.020407233 |
| FRMD3 | 13.33 | 66.28 | 2.31 | 0.005172592 |
| DHRS3 | 581.21 | 2952.72 | 2.34 | 1.52E-05 |
| RIPK4 | 893.39 | 4569.88 | 2.35 | 1.23E-07 |
| GPX2 | 568.88 | 2913.92 | 2.36 | 0.014478431 |
| SGK2 | 9.93 | 51.18 | 2.37 | 0.008901439 |
| LAMA4 | 24.94 | 128.72 | 2.37 | 0.003711473 |
| ACE2 | 22.01 | 116.24 | 2.40 | 4.06E-05 |
| CEL | 78.45 | 417.53 | 2.41 | 0.009936904 |
| FGFR3 | 643.65 | 3428.88 | 2.41 | 0.000263173 |
| TRIM31 | 4.82 | 25.79 | 2.42 | 0.095602302 |
| TMPRSS2 | 52.83 | 287.44 | 2.44 | 0.084640059 |
| AKR1C1 | 929.39 | 5117.52 | 2.46 | 1.20E-12 |
| ACSBG1 | 6.07 | 33.61 | 2.47 | 0.032997602 |
| LYPD6B | 44.18 | 246.85 | 2.48 | 4.73E-07 |
| IRF1 | 442.23 | 2478.69 | 2.49 | 1.18E-05 |
| ILDR1 | 24.52 | 137.53 | 2.49 | 0.00569028 |
| OR7E14P | 6.07 | 34.06 | 2.49 | 0.028536914 |
| PP14571 | 19.14 | 108.79 | 2.51 | 0.039635423 |
| BAALC | 5.42 | 30.79 | 2.51 | 0.062255183 |
| VILL | 165.74 | 947.43 | 2.52 | 2.16E-06 |
| GOLGA2P9 | 24.09 | 137.77 | 2.52 | 0.008348069 |
| CYP3A5 | 210.01 | 1203.77 | 2.52 | 0.033212078 |
| HRNR | 13.45 | 77.14 | 2.52 | 0.083178926 |
| MSMB | 6.55 | 37.83 | 2.53 | 0.070740073 |
| SAMD12 | 98.94 | 574.28 | 2.54 | 0.00400462 |
| PRR15L | 15.27 | 88.70 | 2.54 | 0.000369387 |
| MPPED2 | 5.09 | 29.61 | 2.54 | 0.070883328 |
| SPINK1 | 11.38 | 66.13 | 2.54 | 0.001684285 |
| CYP2W1 | 4.78 | 27.80 | 2.54 | 0.063075285 |
| MUC20 | 130.62 | 768.96 | 2.56 | 0.040120812 |
| PLEKHA7 | 107.02 | 630.03 | 2.56 | 0.0007041 |
| AQP3 | 993.60 | 5869.87 | 2.56 | 1.70E-06 |
| DEFB1 | 133.31 | 793.87 | 2.57 | 0.001727726 |
| METTL7A | 189.63 | 1129.97 | 2.58 | 0.000420776 |
| GAS2 | 4.53 | 27.01 | 2.58 | 0.070834006 |
| SAA1 | 419.95 | 2517.09 | 2.58 | 0.000374469 |
| CEBPA | 40.04 | 240.48 | 2.59 | 0.004621482 |
| HS3ST6 | 6.26 | 37.70 | 2.59 | 0.053244543 |
| CYP2C8 | 3.89 | 23.85 | 2.61 | 0.079527711 |
| KLHDC7A | 10.89 | 66.88 | 2.62 | 0.042555999 |
| CBFA2T3 | 9.25 | 57.28 | 2.63 | 0.012529445 |
| LYNX1 | 24.93 | 156.16 | 2.65 | 1.85E-06 |
| HID1 | 64.93 | 407.93 | 2.65 | 0.001001361 |
| FA2H | 35.65 | 230.12 | 2.69 | 5.04E-09 |
| MYCL | 280.23 | 1809.59 | 2.69 | 1.90E-15 |
| DDIT4L | 4.28 | 27.95 | 2.71 | 0.04638381 |
| RTP4 | 31.62 | 210.57 | 2.74 | 1.58E-06 |
| TNNT2 | 13.82 | 92.49 | 2.74 | 2.68E-05 |
| EPB41L1 | 111.93 | 753.04 | 2.75 | 0.003093583 |
| SLC40A1 | 34.54 | 233.70 | 2.76 | 0.031851552 |
| SLC27A2 | 20.63 | 140.32 | 2.77 | 0.016850901 |
| FAM174B | 62.21 | 429.27 | 2.79 | 2.51E-11 |
| GBP2 | 217.96 | 1542.94 | 2.82 | 0.015773655 |
| AKR1C2 | 763.94 | 5483.22 | 2.84 | 2.15E-17 |
| STRA6 | 36.49 | 262.20 | 2.85 | 0.000963631 |
| PELI2 | 2.69 | 19.71 | 2.87 | 0.098282721 |
| NUPR1 | 320.12 | 2354.09 | 2.88 | 0.040740558 |
| SAA2 | 253.82 | 1869.76 | 2.88 | 3.87E-05 |
| SYTL5 | 20.49 | 151.02 | 2.88 | 2.36E-07 |
| SPTSSB | 37.35 | 275.30 | 2.88 | 7.75E-06 |
| MIR614 | 3.93 | 29.04 | 2.89 | 0.028480674 |
| GOLT1A | 3.58 | 26.68 | 2.90 | 0.034002279 |
| RARRES3 | 76.05 | 576.31 | 2.92 | 0.008751712 |
| MCF2L | 2.42 | 18.76 | 2.95 | 0.098420347 |
| LINC01133 | 4.21 | 32.79 | 2.96 | 0.012193468 |
| UPK2 | 12.52 | 98.15 | 2.97 | 0.00551444 |
| HMGCS2 | 20.30 | 161.42 | 2.99 | 0.061693139 |
| UGT1A4 | 2.44 | 19.80 | 3.02 | 0.073592699 |
| FGR | 3.65 | 29.75 | 3.03 | 0.015830697 |
| KLK9 | 14.58 | 121.29 | 3.06 | 0.081083431 |
| HPGD | 185.02 | 1539.48 | 3.06 | 0.035690983 |
| IFITM10 | 62.61 | 524.81 | 3.07 | 0.000521378 |
| RARRES1 | 24.53 | 206.91 | 3.08 | 9.79E-11 |
| SLC47A2 | 4.75 | 40.13 | 3.08 | 0.057467989 |
| TNFAIP2 | 696.12 | 5904.40 | 3.08 | 0.000249502 |
| MUC1 | 169.03 | 1470.58 | 3.12 | 6.13E-18 |
| SSPO | 2.46 | 21.40 | 3.12 | 0.051133439 |
| USP30-AS1 | 2.13 | 18.58 | 3.12 | 0.086276273 |
| LOC102659288 | 9.20 | 81.48 | 3.15 | 0.081760701 |
| CD36 | 6.07 | 53.97 | 3.15 | 0.000234174 |
| UGT2B17 | 2.41 | 21.49 | 3.16 | 0.057719078 |
| PLA2G10 | 3.88 | 34.65 | 3.16 | 0.034844075 |
| CFB | 186.20 | 1661.58 | 3.16 | 0.005123195 |
| PLEKHG7 | 2.73 | 24.92 | 3.19 | 0.026888662 |
| DUOX2 | 146.75 | 1340.19 | 3.19 | 1.23E-18 |
| LINC01004 | 335.66 | 3071.81 | 3.19 | 2.74E-13 |
| FBXO32 | 175.13 | 1609.46 | 3.20 | 0.058160903 |
| RAB26 | 23.43 | 215.88 | 3.20 | 5.30E-06 |
| ATP10B | 25.51 | 236.48 | 3.21 | 7.32E-07 |
| GGT6 | 41.33 | 393.01 | 3.25 | 5.58E-07 |
| FOXO6 | 4.79 | 46.82 | 3.29 | 0.000415245 |
| CYP4F3 | 6.25 | 62.12 | 3.31 | 0.006935262 |
| GNAO1 | 12.00 | 120.78 | 3.33 | 1.54E-07 |
| EPHA4 | 20.52 | 206.78 | 3.33 | 7.83E-11 |
| DUOXA2 | 20.93 | 213.97 | 3.35 | 2.99E-08 |
| CIITA | 7.53 | 77.50 | 3.36 | 7.41E-06 |
| EPHB6 | 11.09 | 114.85 | 3.37 | 1.48E-08 |
| KRT13 | 630.28 | 6648.98 | 3.40 | 1.68E-06 |
| SCNN1B | 7.75 | 82.84 | 3.42 | 0.058160903 |
| FOXN1 | 10.46 | 112.69 | 3.43 | 1.62E-06 |
| RCSD1 | 4.49 | 48.94 | 3.45 | 0.000581579 |
| LOC102724550 | 9.38 | 102.66 | 3.45 | 0.000315474 |
| CYP4F12 | 5.39 | 59.25 | 3.46 | 5.92E-05 |
| LY6D | 132.07 | 1488.45 | 3.49 | 1.64E-14 |
| PPARGC1A | 2.12 | 24.27 | 3.52 | 0.01803837 |
| SPON2 | 2.13 | 24.44 | 3.52 | 0.014478431 |
| FAM3B | 2.40 | 27.77 | 3.53 | 0.008624004 |
| LINC01213 | 1.49 | 17.68 | 3.57 | 0.054360024 |
| AKR1B10 | 60.72 | 722.35 | 3.57 | 0.002429254 |
| UPK3B | 113.44 | 1452.37 | 3.68 | 2.47E-08 |
| RNF223 | 2.09 | 27.38 | 3.71 | 0.005708401 |
| OLR1 | 17.95 | 238.97 | 3.73 | 9.92E-10 |
| UPK1A | 2.38 | 32.48 | 3.77 | 0.001481096 |
| LINC00273 | 7.32 | 102.17 | 3.80 | 4.47E-08 |
| RASSF2 | 3.59 | 52.78 | 3.88 | 3.87E-05 |
| TRPV6 | 1.79 | 27.35 | 3.93 | 0.006817023 |
| IGFBP3 | 199.61 | 3112.20 | 3.96 | 0.02263651 |
| FYB | 86.95 | 1357.55 | 3.96 | 0.000352336 |
| RARB | 6.27 | 101.32 | 4.01 | 0.011352077 |
| LOXL4 | 6.61 | 110.93 | 4.07 | 2.21E-09 |
| TNNT3 | 5.72 | 97.06 | 4.08 | 2.86E-09 |
| H19 | 504.94 | 8871.92 | 4.14 | 2.33E-33 |
| CXADRP2 | 0.91 | 16.26 | 4.17 | 0.04294334 |
| IDO1 | 4.48 | 81.47 | 4.18 | 7.38E-08 |
| ELF3 | 344.85 | 6337.76 | 4.20 | 6.01E-07 |
| ANKRD30BL | 6.17 | 116.94 | 4.24 | 0.098421779 |
| CSF3R | 1.20 | 24.73 | 4.36 | 0.003379431 |
| COLCA2 | 1.20 | 25.16 | 4.39 | 0.002537341 |
| UBD | 1.48 | 30.99 | 4.39 | 0.073630115 |
| ALDH3A1 | 8.14 | 270.85 | 5.06 | 1.42E-22 |
| CYP1B1 | 212.11 | 7412.79 | 5.13 | 8.87E-11 |
| TEX101 | 104.01 | 3652.48 | 5.13 | 0.001980148 |
| LOC101060351 | 1.81 | 64.96 | 5.16 | 2.88E-08 |
| LOC101060389 | 1.81 | 64.96 | 5.16 | 2.88E-08 |
| LOC102724862 | 1.81 | 64.96 | 5.16 | 2.88E-08 |
| CYP1A1 | 151.63 | 8460.13 | 5.80 | 1.47E-16 |
| SAA2-SAA4 | 0.31 | 30.88 | 6.63 | 3.06E-05 |
| FKBP1A-SDCBP2 | 0.00 | 27.75 | Inf | 1.20E-05 |
| GOLGA8EP | 0.00 | 10.15 | Inf | 0.052918507 |
| LOC101060376 | 0.00 | 28.45 | Inf | 1.79E-05 |
| MIR3687-1 | 0.00 | 10.85 | Inf | 0.040935635 |
| TBC1D3H | 0.00 | 22.38 | Inf | 0.000303842 |
| VWA5B1 | 0.00 | 8.70 | Inf | 0.095602302 |
| CPED1 | 18.14 | 0.00 | -Inf | 0.005022618 |
| EGR2 | 18.12 | 0.00 | -Inf | 0.005559889 |
| HSFX2 | 11.96 | 0.00 | -Inf | 0.067839273 |
| HSPE1-MOB4 | 13.16 | 0.00 | -Inf | 0.045712824 |
| LINC00705 | 13.29 | 0.00 | -Inf | 0.030891677 |
| SYS1-DBNDD2 | 28.18 | 0.00 | -Inf | 0.000143911 |

Supplementary Table 1B. Genes differentially expressed at 144 h.

| Gene ID | 144 h Control RPKM | 144 h Differentiated RPKM | log2 Fold Change | Adjusted p-value |
| --- | --- | --- | --- | --- |
| GTF2H2C | 36.96 | 0.00 | -Inf | 1.54E-06 |
| NUDT4P1 | 84.15 | 0.00 | -Inf | 1.03E-12 |
| TRIM73 | 17.92 | 0.00 | -Inf | 0.0088442 |
| BPIFA2 | 0.00 | 10.69 | Inf | 0.078375007 |
| GAGE5 | 0.00 | 16.13 | Inf | 0.010303675 |
| LOC101060376 | 0.00 | 38.38 | Inf | 7.14E-07 |
| TBC1D3F | 0.00 | 11.80 | Inf | 0.042880888 |
| TBC1D3H | 0.00 | 14.71 | Inf | 0.018885103 |
| ACSM6 | 0.25 | 19.65 | 6.31 | 0.007288252 |
| TLE6 | 7.18 | 435.14 | 5.92 | 4.26E-35 |
| VWA5B1 | 9.60 | 570.28 | 5.89 | 3.48E-39 |
| FKBP1A-SDCBP2 | 0.37 | 21.70 | 5.86 | 0.003774969 |
| FAM3B | 17.47 | 888.51 | 5.67 | 3.20E-44 |
| RASSF2 | 10.22 | 468.90 | 5.52 | 1.76E-33 |
| PM20D1 | 4.06 | 178.97 | 5.46 | 1.71E-05 |
| BMP3 | 23.66 | 1041.74 | 5.46 | 6.29E-25 |
| SCUBE2 | 11.32 | 475.69 | 5.39 | 1.42E-13 |
| LIN7A | 1.30 | 54.27 | 5.38 | 0.008572655 |
| MAL | 13.06 | 536.07 | 5.36 | 1.76E-33 |
| UBD | 5.88 | 237.26 | 5.33 | 6.57E-22 |
| PIGR | 73.67 | 2867.02 | 5.28 | 4.55E-09 |
| UPK3A | 7.00 | 266.72 | 5.25 | 9.40E-24 |
| HLA-DRB5 | 9.82 | 365.77 | 5.22 | 2.04E-05 |
| CYP4Z2P | 0.62 | 22.35 | 5.17 | 0.005045439 |
| ANKFN1 | 1.86 | 66.90 | 5.16 | 1.26E-05 |
| LONRF3 | 0.37 | 12.60 | 5.08 | 0.086568933 |
| SNX31 | 9.27 | 307.83 | 5.05 | 6.13E-13 |
| UPK2 | 71.78 | 2353.70 | 5.04 | 0.000223058 |
| HPGD | 1305.93 | 41083.87 | 4.98 | 9.09E-53 |
| LOC729966 | 2.96 | 89.51 | 4.92 | 0.024442184 |
| UPK1A | 50.50 | 1437.88 | 4.83 | 5.24E-06 |
| AOC1 | 15.57 | 441.10 | 4.82 | 3.12E-14 |
| DHRS2 | 602.60 | 16724.92 | 4.79 | 0.00929245 |
| C11orf53 | 3.45 | 94.11 | 4.77 | 0.005725969 |
| HLA-DRA | 71.78 | 1940.54 | 4.76 | 0.011468102 |
| ACSBG1 | 17.06 | 458.46 | 4.75 | 1.02E-27 |
| TRIM31 | 6.81 | 177.54 | 4.70 | 1.29E-15 |
| HLA-DRB1 | 65.21 | 1613.33 | 4.63 | 2.52E-05 |
| CABLES1 | 14.49 | 355.12 | 4.62 | 3.88E-23 |
| SLC4A4 | 0.95 | 22.90 | 4.59 | 0.006261591 |
| CRNN | 2.03 | 46.71 | 4.53 | 0.001720067 |
| CAPN8 | 5.36 | 122.19 | 4.51 | 2.39E-11 |
| CYP4F8 | 5.28 | 117.05 | 4.47 | 0.017115009 |
| SLAIN1 | 1.12 | 24.67 | 4.46 | 0.006682958 |
| TXK | 4.33 | 95.27 | 4.46 | 0.014725401 |
| LRRC31 | 2.86 | 62.37 | 4.45 | 0.004668735 |
| INHBB | 3.96 | 84.77 | 4.42 | 3.67E-08 |
| SCNN1G | 37.94 | 809.64 | 4.42 | 1.63E-29 |
| TMPRSS2 | 121.03 | 2539.04 | 4.39 | 1.34E-37 |
| ADIRF | 616.32 | 12803.64 | 4.38 | 3.54E-07 |
| PRR15 | 18.67 | 382.61 | 4.36 | 1.57E-22 |
| MCF2L | 11.31 | 230.12 | 4.35 | 4.52E-08 |
| PLCE1 | 81.11 | 1608.31 | 4.31 | 3.78E-07 |
| ASXL3 | 1.24 | 23.98 | 4.27 | 0.008843786 |
| SPINK1 | 86.65 | 1649.17 | 4.25 | 3.50E-33 |
| CP | 22.71 | 430.19 | 4.24 | 9.27E-05 |
| KLHDC7A | 57.38 | 1075.86 | 4.23 | 1.39E-08 |
| PDE1B | 2.65 | 49.46 | 4.22 | 0.001930574 |
| WFDC13 | 1.47 | 27.29 | 4.21 | 0.007860342 |
| KIF5C | 2.42 | 44.07 | 4.19 | 9.70E-05 |
| GPRIN3 | 1.20 | 21.37 | 4.16 | 0.026838458 |
| LGALS9 | 41.80 | 739.84 | 4.15 | 6.55E-28 |
| BCAS1 | 99.26 | 1748.87 | 4.14 | 2.64E-07 |
| RARRES1 | 145.83 | 2533.46 | 4.12 | 0.006585993 |
| LINC00675 | 3.36 | 58.24 | 4.12 | 0.008254364 |
| KCNK5 | 5.67 | 98.11 | 4.11 | 3.95E-08 |
| ANXA10 | 16.81 | 290.20 | 4.11 | 0.042680136 |
| HLA-DPB1 | 12.34 | 212.16 | 4.10 | 7.74E-09 |
| CLIC5 | 6.33 | 107.84 | 4.09 | 8.22E-05 |
| GPR160 | 60.34 | 1019.25 | 4.08 | 6.71E-08 |
| STK32A | 33.38 | 563.53 | 4.08 | 2.09E-21 |
| TFF1 | 6.08 | 101.79 | 4.07 | 0.088194167 |
| HS6ST3 | 4.99 | 82.79 | 4.05 | 5.10E-08 |
| PSCA | 1294.10 | 21430.40 | 4.05 | 1.37E-05 |
| HLA-DQA2 | 1.08 | 17.80 | 4.05 | 0.06011128 |
| FBP1 | 11.08 | 182.85 | 4.04 | 6.50E-09 |
| COL1A2 | 2.09 | 33.79 | 4.01 | 0.004345719 |
| NUP210 | 7.89 | 126.81 | 4.01 | 2.97E-06 |
| FER1L6 | 73.02 | 1162.15 | 3.99 | 0.076574254 |
| PLEKHB1 | 2.24 | 35.11 | 3.97 | 0.045908556 |
| RGL3 | 3.69 | 55.44 | 3.91 | 4.32E-05 |
| GOLT1A | 11.91 | 178.41 | 3.91 | 2.37E-12 |
| HMGCS2 | 179.48 | 2647.68 | 3.88 | 0.00013698 |
| SCNN1B | 71.99 | 1061.51 | 3.88 | 8.18E-27 |
| KSR2 | 8.31 | 122.13 | 3.88 | 0.001320477 |
| SPOCK2 | 26.50 | 380.18 | 3.84 | 0.064780062 |
| SPTSSB | 265.73 | 3804.39 | 3.84 | 8.40E-11 |
| TSPAN2 | 15.12 | 213.59 | 3.82 | 0.000188835 |
| PKD1L1 | 4.55 | 64.27 | 3.82 | 0.000987569 |
| HLA-DRB6 | 9.34 | 131.84 | 3.82 | 2.12E-09 |
| KCNQ3 | 9.96 | 139.45 | 3.81 | 1.36E-06 |
| TLR4 | 21.04 | 292.02 | 3.79 | 1.96E-16 |
| STRA6 | 65.32 | 903.20 | 3.79 | 0.033289833 |
| ITGB3 | 8.09 | 111.88 | 3.79 | 4.74E-08 |
| P2RX2 | 5.09 | 70.00 | 3.78 | 2.97E-06 |
| SERPINB11 | 40.51 | 549.49 | 3.76 | 0.0178238 |
| VSIG2 | 137.19 | 1795.58 | 3.71 | 0.000813854 |
| CD96 | 7.97 | 101.81 | 3.67 | 7.07E-08 |
| CHN2 | 16.02 | 204.39 | 3.67 | 0.097089502 |
| B3GALT5 | 17.70 | 224.80 | 3.67 | 8.46E-13 |
| SPIRE2 | 43.18 | 540.22 | 3.65 | 5.98E-19 |
| CAPN13 | 4.09 | 50.96 | 3.64 | 0.010914895 |
| PDK4 | 19.73 | 240.20 | 3.61 | 4.80E-13 |
| COLCA1 | 41.94 | 496.98 | 3.57 | 1.62E-14 |
| CYP4F22 | 2.92 | 34.40 | 3.56 | 0.007288252 |
| SRMS | 7.24 | 83.51 | 3.53 | 1.90E-05 |
| RAB15 | 121.94 | 1402.69 | 3.52 | 5.45E-08 |
| CLIC6 | 12.49 | 142.59 | 3.51 | 7.30E-08 |
| HLA-DMB | 16.83 | 190.44 | 3.50 | 6.66E-11 |
| SYTL5 | 121.56 | 1352.69 | 3.48 | 2.97E-06 |
| IL9R | 45.21 | 495.58 | 3.45 | 9.43E-17 |
| GKN1 | 8.54 | 93.58 | 3.45 | 6.54E-05 |
| ERN2 | 83.34 | 911.02 | 3.45 | 3.56E-13 |
| RHOU | 182.14 | 1971.56 | 3.44 | 6.40E-18 |
| TP53I11 | 84.73 | 904.83 | 3.42 | 2.31E-12 |
| PDE10A | 74.21 | 789.55 | 3.41 | 0.000569629 |
| GPX2 | 445.14 | 4731.51 | 3.41 | 0.070254565 |
| AGR2 | 326.89 | 3453.55 | 3.40 | 0.000264887 |
| LIMCH1 | 234.29 | 2459.03 | 3.39 | 5.03E-18 |
| CX3CL1 | 222.02 | 2315.65 | 3.38 | 2.42E-05 |
| LINC00930 | 2.96 | 29.42 | 3.31 | 0.026935895 |
| AGAP11 | 38.59 | 381.97 | 3.31 | 4.32E-14 |
| COL3A1 | 3.02 | 29.73 | 3.30 | 0.032980978 |
| FRY | 23.61 | 230.29 | 3.29 | 0.001153114 |
| PPP1R9A | 14.67 | 141.61 | 3.27 | 0.000109396 |
| ERICH5 | 13.40 | 129.17 | 3.27 | 1.04E-07 |
| PLEKHS1 | 5.51 | 51.54 | 3.23 | 0.016419276 |
| TBX2 | 3.02 | 27.67 | 3.20 | 0.046475771 |
| FAM174B | 197.34 | 1774.31 | 3.17 | 0.011205168 |
| RAB17 | 16.51 | 148.45 | 3.17 | 0.000914242 |
| C8orf4 | 347.21 | 3119.31 | 3.17 | 0.001288068 |
| KRT13 | 1592.13 | 14293.80 | 3.17 | 2.40E-09 |
| WNK2 | 57.46 | 513.60 | 3.16 | 1.75E-12 |
| NRXN3 | 42.34 | 375.61 | 3.15 | 0.014340076 |
| CLDN8 | 11.43 | 100.03 | 3.13 | 3.49E-06 |
| TLE2 | 133.35 | 1153.46 | 3.11 | 0.00750251 |
| ACSL5 | 370.68 | 3195.91 | 3.11 | 0.010714408 |
| MMEL1 | 2.61 | 22.41 | 3.10 | 0.092979239 |
| ACER2 | 381.95 | 3263.21 | 3.09 | 2.55E-12 |
| SLC34A2 | 7.26 | 61.07 | 3.07 | 0.001006909 |
| IYD | 31.64 | 261.02 | 3.04 | 2.72E-07 |
| TJP3 | 80.19 | 659.51 | 3.04 | 1.28E-05 |
| ENPP4 | 45.90 | 374.95 | 3.03 | 6.56E-07 |
| HLA-DPA1 | 88.12 | 717.96 | 3.03 | 0.011622253 |
| SLC44A4 | 286.66 | 2331.05 | 3.02 | 1.37E-05 |
| DEGS2 | 8.15 | 65.75 | 3.01 | 0.001183245 |
| ABCA13 | 56.00 | 448.98 | 3.00 | 0.000105529 |
| HID1 | 156.07 | 1249.03 | 3.00 | 0.001440438 |
| ABCC3 | 313.44 | 2476.40 | 2.98 | 7.99E-05 |
| FMO9P | 4.59 | 35.18 | 2.94 | 0.014291085 |
| HLA-DOA | 25.55 | 194.98 | 2.93 | 1.28E-05 |
| COLCA2 | 16.40 | 124.92 | 2.93 | 4.22E-06 |
| CACNA1H | 9.97 | 74.68 | 2.91 | 0.007924592 |
| ATP2A3 | 5.11 | 38.20 | 2.90 | 0.007736056 |
| DAPK1 | 186.96 | 1385.62 | 2.89 | 1.36E-16 |
| FAM46C | 17.94 | 132.65 | 2.89 | 3.35E-06 |
| RAB6B | 71.05 | 522.36 | 2.88 | 1.00E-14 |
| SLC41A2 | 48.47 | 351.26 | 2.86 | 3.36E-10 |
| SLC9A2 | 6.96 | 50.21 | 2.85 | 0.007039219 |
| CRACR2B | 23.04 | 163.95 | 2.83 | 5.65E-07 |
| BIRC3 | 241.59 | 1716.53 | 2.83 | 0.086158086 |
| MYEOV | 29.71 | 210.61 | 2.83 | 6.54E-05 |
| PLA2G2F | 70.13 | 488.54 | 2.80 | 0.08721079 |
| MGAT3 | 132.35 | 921.18 | 2.80 | 1.04E-07 |
| SPINK8 | 3.94 | 27.32 | 2.80 | 0.06171222 |
| FAM105A | 120.19 | 827.87 | 2.78 | 5.15E-16 |
| FREM1 | 12.19 | 82.50 | 2.76 | 0.029400948 |
| TMEM56 | 66.20 | 442.95 | 2.74 | 1.83E-10 |
| CYP4F24P | 4.72 | 31.39 | 2.73 | 0.068041886 |
| SRCIN1 | 11.49 | 76.26 | 2.73 | 0.000180357 |
| FAM3D | 26.94 | 177.23 | 2.72 | 1.36E-06 |
| ENTPD8 | 7.99 | 52.11 | 2.71 | 0.005916618 |
| ARRB1 | 35.40 | 228.69 | 2.69 | 1.59E-07 |
| CRISP3 | 229.46 | 1479.50 | 2.69 | 0.008148585 |
| USP30-AS1 | 4.53 | 28.85 | 2.67 | 0.099802166 |
| CPAMD8 | 28.03 | 175.57 | 2.65 | 0.010303675 |
| IDO1 | 13.60 | 85.10 | 2.65 | 0.000744621 |
| KCNE3 | 9.17 | 57.29 | 2.64 | 0.005615389 |
| APOD | 9.89 | 61.39 | 2.63 | 0.004668735 |
| CTSS | 294.68 | 1818.29 | 2.63 | 2.15E-05 |
| C12orf74 | 4.37 | 26.82 | 2.62 | 0.098516306 |
| B3GNT3 | 458.79 | 2812.19 | 2.62 | 0.000803545 |
| SMIM5 | 90.09 | 547.38 | 2.60 | 1.14E-11 |
| SMAD6 | 7.56 | 45.58 | 2.59 | 0.028457046 |
| CYP4F12 | 103.93 | 623.20 | 2.58 | 8.20E-11 |
| C21orf88 | 10.75 | 64.07 | 2.58 | 0.002770614 |
| CNIH3 | 9.37 | 55.45 | 2.56 | 0.009707591 |
| ENPP1 | 10.04 | 59.08 | 2.56 | 0.010945111 |
| AKAP12 | 202.17 | 1174.34 | 2.54 | 4.96E-05 |
| SPATA13 | 49.70 | 287.15 | 2.53 | 1.59E-08 |
| ELF3 | 2528.89 | 14587.22 | 2.53 | 0.01602595 |
| ST3GAL1 | 961.18 | 5542.68 | 2.53 | 8.22E-14 |
| MX2 | 137.39 | 790.58 | 2.52 | 7.82E-05 |
| CYP4B1 | 256.58 | 1456.07 | 2.50 | 0.098612901 |
| MUC20 | 460.95 | 2587.16 | 2.49 | 0.005748198 |
| CCSER1 | 36.41 | 204.04 | 2.49 | 0.001292058 |
| SEMA5A | 303.13 | 1690.18 | 2.48 | 0.006148493 |
| MMP10 | 116.96 | 651.38 | 2.48 | 0.031376545 |
| MYZAP | 103.70 | 572.69 | 2.47 | 1.06E-09 |
| FLJ22184 | 9.65 | 52.94 | 2.46 | 0.012281973 |
| NCCRP1 | 440.57 | 2412.15 | 2.45 | 1.32E-13 |
| IGFBP3 | 2493.72 | 13639.42 | 2.45 | 0.00235365 |
| PPFIBP2 | 625.90 | 3392.77 | 2.44 | 6.41E-10 |
| FAM131B | 13.35 | 71.65 | 2.42 | 0.007924592 |
| ALOX5AP | 94.74 | 508.11 | 2.42 | 5.48E-09 |
| CHPT1 | 174.26 | 930.54 | 2.42 | 4.22E-10 |
| VGLL1 | 416.75 | 2195.63 | 2.40 | 0.03231999 |
| CEACAM1 | 629.90 | 3272.01 | 2.38 | 0.003578445 |
| BPGM | 861.61 | 4455.28 | 2.37 | 0.020935385 |
| CAPN5 | 245.11 | 1258.28 | 2.36 | 8.22E-05 |
| RHBG | 42.03 | 215.07 | 2.36 | 1.95E-06 |
| DENND2D | 294.19 | 1491.79 | 2.34 | 3.75E-05 |
| TSPAN15 | 208.43 | 1056.25 | 2.34 | 0.000492104 |
| DOCK11 | 12.77 | 63.42 | 2.31 | 0.012834105 |
| PODXL | 20.72 | 102.22 | 2.30 | 0.00094641 |
| LAMA4 | 53.87 | 262.02 | 2.28 | 0.005921673 |
| BATF | 45.27 | 216.56 | 2.26 | 2.43E-06 |
| PIP5K1B | 12.94 | 61.83 | 2.26 | 0.006578878 |
| DTX4 | 1552.97 | 7289.28 | 2.23 | 0.034457603 |
| TMEM125 | 38.85 | 180.69 | 2.22 | 0.003774969 |
| ST3GAL4 | 383.02 | 1774.82 | 2.21 | 8.73E-10 |
| PARM1 | 34.15 | 157.55 | 2.21 | 0.000712613 |
| ACOXL | 22.15 | 102.08 | 2.20 | 0.002478923 |
| SIGLEC15 | 10.82 | 49.47 | 2.19 | 0.059737155 |
| ACSM3 | 120.19 | 549.25 | 2.19 | 0.036884338 |
| LOC102659288 | 101.67 | 460.15 | 2.18 | 0.070458646 |
| FOXO6 | 26.95 | 121.65 | 2.17 | 0.08562841 |
| SLC46A3 | 19.29 | 86.36 | 2.16 | 0.026838458 |
| TSPAN12 | 79.60 | 350.52 | 2.14 | 4.34E-06 |
| CCRL2 | 16.33 | 71.77 | 2.14 | 0.08562841 |
| HSH2D | 59.66 | 261.79 | 2.13 | 4.08E-06 |
| APOBEC3A | 17.15 | 74.52 | 2.12 | 0.034080347 |
| CXCL17 | 319.37 | 1385.22 | 2.12 | 1.78E-05 |
| HS3ST6 | 58.60 | 251.63 | 2.10 | 0.009414353 |
| TBC1D30 | 49.48 | 210.85 | 2.09 | 0.05546651 |
| WFDC2 | 22.24 | 94.26 | 2.08 | 0.007309036 |
| PLIN2 | 710.42 | 2968.56 | 2.06 | 0.000160381 |
| SH3TC2 | 649.12 | 2707.58 | 2.06 | 0.001322338 |
| HNMT | 206.01 | 859.14 | 2.06 | 0.09259379 |
| FRMD3 | 32.99 | 137.45 | 2.06 | 0.000847092 |
| NAALADL2 | 51.10 | 211.99 | 2.05 | 0.000111163 |
| UNC93B1 | 554.91 | 2296.62 | 2.05 | 0.001002802 |
| ACADM | 823.87 | 3396.40 | 2.04 | 0.000200144 |
| C2orf54 | 57.80 | 238.22 | 2.04 | 0.007288252 |
| LINC01207 | 24.94 | 102.69 | 2.04 | 0.002957366 |
| OAS1 | 410.77 | 1690.53 | 2.04 | 2.54E-05 |
| PRSS27 | 249.51 | 1010.70 | 2.02 | 0.000577456 |
| KRT4 | 472.86 | 1911.81 | 2.02 | 0.01694872 |
| SORCS2 | 187.52 | 754.66 | 2.01 | 1.16E-06 |
| ST6GALNAC1 | 63.14 | 250.82 | 1.99 | 0.000288704 |
| SEMA7A | 291.70 | 1152.80 | 1.98 | 2.24E-07 |
| SCCPDH | 1122.56 | 4422.90 | 1.98 | 3.19E-08 |
| ACOT11 | 155.91 | 612.57 | 1.97 | 0.062224486 |
| ABCC4 | 214.41 | 837.96 | 1.97 | 4.28E-07 |
| PLCB4 | 42.12 | 164.40 | 1.96 | 0.00018063 |
| AGMO | 38.12 | 147.27 | 1.95 | 0.047489471 |
| CLDN4 | 1335.98 | 5126.32 | 1.94 | 2.54E-05 |
| IRF1 | 670.18 | 2565.04 | 1.94 | 0.01694872 |
| LOC115110 | 59.98 | 224.51 | 1.90 | 7.15E-05 |
| STS | 356.78 | 1335.11 | 1.90 | 4.04E-08 |
| GPD1L | 347.65 | 1297.02 | 1.90 | 0.044082602 |
| ADAM28 | 180.12 | 669.47 | 1.89 | 0.065268032 |
| CARD9 | 21.48 | 79.64 | 1.89 | 0.060526244 |
| GLRB | 37.05 | 137.17 | 1.89 | 0.000700673 |
| HLA-DMA | 139.41 | 509.83 | 1.87 | 0.000214587 |
| PPM1H | 37.33 | 135.71 | 1.86 | 0.001034842 |
| PDE4C | 55.84 | 202.30 | 1.86 | 0.002308724 |
| NEDD9 | 365.08 | 1317.87 | 1.85 | 0.00013698 |
| FGD3 | 172.10 | 620.64 | 1.85 | 6.25E-06 |
| LOC283070 | 41.52 | 149.20 | 1.85 | 0.007924592 |
| TAPBPL | 278.85 | 993.37 | 1.83 | 0.025636017 |
| ANXA9 | 101.57 | 361.65 | 1.83 | 0.062396387 |
| FUT3 | 356.66 | 1267.98 | 1.83 | 0.005725969 |
| CGN | 662.73 | 2350.75 | 1.83 | 0.0972924 |
| NR1H3 | 118.57 | 419.93 | 1.82 | 0.000100755 |
| BSPRY | 57.58 | 203.88 | 1.82 | 0.028004916 |
| LMO2 | 32.33 | 114.45 | 1.82 | 0.006463922 |
| MMP7 | 51.26 | 181.11 | 1.82 | 0.076678749 |
| TNFRSF21 | 4924.70 | 17368.44 | 1.82 | 3.95E-08 |
| C9orf116 | 22.64 | 79.82 | 1.82 | 0.023026292 |
| MYCL | 1028.10 | 3619.16 | 1.82 | 5.40E-07 |
| MSMB | 60.84 | 213.83 | 1.81 | 0.024465575 |
| GDPD3 | 70.53 | 246.99 | 1.81 | 0.000744621 |
| LOC100128770 | 139.95 | 487.35 | 1.80 | 0.028302925 |
| MYLIP | 330.37 | 1150.20 | 1.80 | 3.75E-06 |
| ZNF737 | 453.74 | 1568.59 | 1.79 | 0.000175075 |
| PLS1 | 347.96 | 1199.18 | 1.79 | 0.01617795 |
| SAMD9L | 369.61 | 1261.71 | 1.77 | 0.053726706 |
| SLC15A1 | 60.05 | 204.92 | 1.77 | 0.003616456 |
| SAMD13 | 37.55 | 127.39 | 1.76 | 0.047002667 |
| AKAP7 | 55.54 | 186.34 | 1.75 | 0.003193648 |
| PLEKHA7 | 520.69 | 1744.77 | 1.74 | 0.010736896 |
| FUT9 | 22.13 | 73.75 | 1.74 | 0.065239481 |
| PRKACB | 674.46 | 2244.33 | 1.73 | 2.81E-06 |
| VSIG10 | 216.95 | 721.42 | 1.73 | 5.24E-06 |
| CYSRT1 | 39.74 | 132.04 | 1.73 | 0.005195922 |
| PSMB10 | 519.89 | 1726.96 | 1.73 | 0.052340928 |
| ZBTB7C | 158.52 | 526.51 | 1.73 | 6.31E-05 |
| CHKA | 355.10 | 1167.72 | 1.72 | 2.76E-05 |
| CD36 | 139.16 | 457.38 | 1.72 | 0.031208709 |
| PKIB | 124.19 | 407.82 | 1.72 | 0.000483279 |
| ARHGDIB | 418.49 | 1372.32 | 1.71 | 1.54E-06 |
| CARD11 | 472.02 | 1537.59 | 1.70 | 0.060220918 |
| CCDC64B | 520.73 | 1695.70 | 1.70 | 0.005172485 |
| TBX6 | 32.51 | 104.71 | 1.69 | 0.032065529 |
| RAB20 | 136.59 | 438.46 | 1.68 | 0.038456643 |
| MLPH | 499.00 | 1598.16 | 1.68 | 4.18E-05 |
| ACKR3 | 1070.14 | 3420.30 | 1.68 | 0.004515663 |
| PLEKHG6 | 404.89 | 1293.72 | 1.68 | 0.004509179 |
| ELF5 | 40.95 | 130.67 | 1.67 | 0.017963828 |
| FAM107B | 482.82 | 1535.79 | 1.67 | 0.005615389 |
| C4orf19 | 400.49 | 1273.41 | 1.67 | 0.091143946 |
| DOCK8 | 55.98 | 177.44 | 1.66 | 0.061629009 |
| PTGR1 | 1657.94 | 5248.75 | 1.66 | 0.011669215 |
| DDAH1 | 129.93 | 410.61 | 1.66 | 0.000914269 |
| DUOXA2 | 83.42 | 263.19 | 1.66 | 0.053726706 |
| PTGER4 | 81.62 | 255.26 | 1.64 | 0.003316911 |
| EHD3 | 411.24 | 1285.99 | 1.64 | 0.073914786 |
| PPAP2A | 415.03 | 1294.08 | 1.64 | 4.32E-05 |
| PPP1R12B | 873.97 | 2718.38 | 1.64 | 2.67E-05 |
| TMEM184A | 1360.02 | 4209.75 | 1.63 | 0.000288069 |
| RALGPS1 | 146.07 | 449.62 | 1.62 | 0.000406839 |
| GATA2 | 97.81 | 296.15 | 1.60 | 0.000625656 |
| MERTK | 76.94 | 231.81 | 1.59 | 0.022359954 |
| BCAT2 | 726.24 | 2179.41 | 1.59 | 0.009714989 |
| FAM149A | 82.62 | 246.77 | 1.58 | 0.004913919 |
| ATP8A1 | 57.26 | 170.57 | 1.57 | 0.035470186 |
| AQP3 | 3924.27 | 11540.06 | 1.56 | 6.31E-05 |
| ATP1B1 | 2793.15 | 8205.81 | 1.55 | 5.98E-06 |
| PLIN4 | 982.32 | 2865.22 | 1.54 | 2.31E-05 |
| MECOM | 748.62 | 2180.84 | 1.54 | 0.010047306 |
| EXOC6 | 102.48 | 297.92 | 1.54 | 0.004515663 |
| GBP3 | 1491.95 | 4309.16 | 1.53 | 0.052737641 |
| ABHD2 | 2654.75 | 7665.64 | 1.53 | 9.83E-05 |
| SH2D4A | 818.12 | 2360.72 | 1.53 | 9.01E-06 |
| IDH1 | 4029.83 | 11623.02 | 1.53 | 6.16E-05 |
| CAMK1D | 67.70 | 193.60 | 1.52 | 0.004286756 |
| OASL | 56.37 | 160.62 | 1.51 | 0.028004916 |
| GMDS | 502.86 | 1414.49 | 1.49 | 0.000402955 |
| SLC40A1 | 167.11 | 467.75 | 1.48 | 0.009714989 |
| SPOCD1 | 422.80 | 1182.80 | 1.48 | 0.01617795 |
| TNFRSF10C | 97.32 | 271.80 | 1.48 | 0.002432645 |
| APOL6 | 1867.61 | 5171.24 | 1.47 | 0.001024075 |
| SRD5A3 | 761.49 | 2095.77 | 1.46 | 0.001380247 |
| SPTBN5 | 45.61 | 124.99 | 1.45 | 0.026935895 |
| SLC29A3 | 97.98 | 267.57 | 1.45 | 0.009714989 |
| C5orf56 | 35.11 | 95.87 | 1.45 | 0.088100006 |
| NBEA | 75.57 | 206.19 | 1.45 | 0.046378332 |
| LIPE | 162.47 | 442.59 | 1.45 | 0.003109886 |
| EPS8 | 383.48 | 1040.31 | 1.44 | 0.000598225 |
| STK10 | 411.19 | 1113.46 | 1.44 | 0.009238975 |
| KRT7 | 35291.18 | 94373.07 | 1.42 | 0.031445696 |
| RILP | 80.08 | 213.56 | 1.42 | 0.068917181 |
| ADAM8 | 213.96 | 570.47 | 1.41 | 0.077724495 |
| IFI35 | 168.21 | 447.36 | 1.41 | 0.00350563 |
| LFNG | 52.09 | 138.45 | 1.41 | 0.067814868 |
| MSX2 | 151.04 | 401.31 | 1.41 | 0.002113859 |
| SLC52A3 | 78.50 | 208.31 | 1.41 | 0.029867679 |
| MGAT4A | 209.44 | 553.46 | 1.40 | 0.054929938 |
| ZNF552 | 395.14 | 1035.43 | 1.39 | 0.080984252 |
| TMCO4 | 273.82 | 715.89 | 1.39 | 0.015481809 |
| KRT18 | 3095.82 | 8032.24 | 1.38 | 0.05272417 |
| CDC14A | 67.98 | 175.97 | 1.37 | 0.048528783 |
| SAMD9 | 765.42 | 1981.39 | 1.37 | 0.052371762 |
| MCCC1 | 736.96 | 1902.37 | 1.37 | 0.001755856 |
| FAM213B | 742.27 | 1892.75 | 1.35 | 0.000396894 |
| MFAP3L | 488.91 | 1230.94 | 1.33 | 0.014589321 |
| GMNN | 392.42 | 985.78 | 1.33 | 0.00095211 |
| SPOCK1 | 196.54 | 492.37 | 1.32 | 0.013217314 |
| TBX3 | 2696.64 | 6741.58 | 1.32 | 0.000744486 |
| DUSP16 | 1328.12 | 3310.52 | 1.32 | 0.083823627 |
| ZSWIM4 | 273.83 | 679.79 | 1.31 | 0.001440438 |
| TNS3 | 573.69 | 1423.53 | 1.31 | 0.068882679 |
| CENPV | 92.77 | 228.27 | 1.30 | 0.039539809 |
| DNAJA4 | 853.65 | 2097.09 | 1.30 | 0.000490885 |
| CCNDBP1 | 609.73 | 1493.02 | 1.29 | 0.006682958 |
| MYRF | 117.15 | 286.79 | 1.29 | 0.072491474 |
| BCL6 | 863.78 | 2098.81 | 1.28 | 0.048616174 |
| RPH3AL | 253.17 | 610.61 | 1.27 | 0.057713808 |
| DLG3 | 442.72 | 1064.49 | 1.27 | 0.006122101 |
| SMOX | 232.06 | 556.25 | 1.26 | 0.014580176 |
| PTK2B | 590.06 | 1398.96 | 1.25 | 0.002523408 |
| FUT2 | 144.92 | 343.00 | 1.24 | 0.034457603 |
| CBX6 | 527.88 | 1248.61 | 1.24 | 0.002542261 |
| MCCC2 | 1764.23 | 4118.34 | 1.22 | 0.036021949 |
| HS6ST2 | 334.77 | 781.31 | 1.22 | 0.070901384 |
| ST3GAL5 | 177.85 | 414.64 | 1.22 | 0.060814592 |
| SOWAHB | 306.30 | 712.70 | 1.22 | 0.006246909 |
| ETV3 | 373.07 | 860.60 | 1.21 | 0.005804297 |
| INPP4B | 543.84 | 1252.20 | 1.20 | 0.011319818 |
| MANSC1 | 306.03 | 703.81 | 1.20 | 0.007291789 |
| CAB39L | 467.96 | 1072.48 | 1.20 | 0.00437632 |
| PSMB8 | 776.04 | 1773.31 | 1.19 | 0.072491474 |
| HLA-F | 269.07 | 613.02 | 1.19 | 0.034457603 |
| S100P | 19729.91 | 44545.25 | 1.17 | 0.004881169 |
| LY6E | 2175.43 | 4898.36 | 1.17 | 0.002683129 |
| FAM214A | 1031.11 | 2313.85 | 1.17 | 0.008572655 |
| NFE2L3 | 546.72 | 1225.77 | 1.16 | 0.025349599 |
| ABCD3 | 1599.48 | 3578.78 | 1.16 | 0.012877595 |
| TP53INP2 | 307.20 | 683.32 | 1.15 | 0.026838458 |
| ACOX1 | 2122.77 | 4719.52 | 1.15 | 0.003774969 |
| XBP1 | 3325.15 | 7390.97 | 1.15 | 0.065810612 |
| FAM171B | 157.10 | 348.31 | 1.15 | 0.044621705 |
| SHROOM1 | 292.12 | 646.92 | 1.15 | 0.021922309 |
| SLC17A5 | 577.25 | 1275.21 | 1.14 | 0.012550667 |
| GMIP | 355.34 | 783.24 | 1.14 | 0.011657943 |
| PRKCA | 245.63 | 540.33 | 1.14 | 0.049231989 |
| SP6 | 305.51 | 671.76 | 1.14 | 0.012247277 |
| LINC00963 | 206.69 | 452.56 | 1.13 | 0.026838458 |
| PEX11A | 335.22 | 733.76 | 1.13 | 0.030932971 |
| GTDC1 | 305.78 | 668.30 | 1.13 | 0.006261591 |
| PARD3B | 91.75 | 200.25 | 1.13 | 0.048616174 |
| ASCC2 | 1825.99 | 3979.70 | 1.12 | 0.009567559 |
| GRB7 | 387.38 | 844.14 | 1.12 | 0.011468102 |
| RAB11FIP1 | 3053.93 | 6625.03 | 1.12 | 0.004058386 |
| MEIS3P1 | 211.37 | 458.51 | 1.12 | 0.040908498 |
| LOC90246 | 124.08 | 268.78 | 1.12 | 0.084052775 |
| THEM6 | 585.87 | 1265.04 | 1.11 | 0.018591983 |
| C16orf45 | 179.56 | 382.74 | 1.09 | 0.084052775 |
| ACP6 | 411.31 | 876.18 | 1.09 | 0.061629009 |
| DYRK2 | 713.14 | 1514.53 | 1.09 | 0.007821974 |
| HLA-B | 4977.01 | 10559.83 | 1.09 | 0.006463922 |
| KIF21B | 158.78 | 334.46 | 1.07 | 0.046607818 |
| TSKU | 1349.95 | 2828.33 | 1.07 | 0.04074182 |
| STEAP2 | 385.49 | 803.61 | 1.06 | 0.078754603 |
| GAREM | 590.93 | 1221.90 | 1.05 | 0.014021222 |
| FOXC1 | 330.97 | 684.05 | 1.05 | 0.033122856 |
| DUSP4 | 1552.03 | 3197.97 | 1.04 | 0.057691761 |
| SLC9A8 | 353.68 | 720.80 | 1.03 | 0.04351812 |
| TMEM2 | 1931.12 | 3923.98 | 1.02 | 0.016068382 |
| SVIP | 217.39 | 440.44 | 1.02 | 0.036884338 |
| KDM1A | 3487.83 | 7045.27 | 1.01 | 0.01294688 |
| EFHD2 | 2361.23 | 4760.41 | 1.01 | 0.021946314 |
| TMEM45B | 1237.81 | 2485.66 | 1.01 | 0.01155056 |
| LIPH | 113.87 | 227.85 | 1.00 | 0.090773245 |
| ZNFX1 | 2439.48 | 4861.36 | 0.99 | 0.076902475 |
| NTN4 | 3446.75 | 6850.71 | 0.99 | 0.013429644 |
| ETFDH | 658.92 | 1307.78 | 0.99 | 0.034397829 |
| ACADSB | 877.91 | 1736.63 | 0.98 | 0.073978535 |
| IFIT3 | 366.78 | 724.72 | 0.98 | 0.096683983 |
| NT5C2 | 2740.03 | 5394.50 | 0.98 | 0.02446363 |
| TRIM14 | 1322.35 | 2594.98 | 0.97 | 0.062210188 |
| B2M | 17286.10 | 33867.18 | 0.97 | 0.025224148 |
| KRT8 | 8764.39 | 17047.23 | 0.96 | 0.040629404 |
| TRAK1 | 2891.79 | 5614.07 | 0.96 | 0.048616174 |
| RIPK4 | 2035.69 | 3938.49 | 0.95 | 0.038456643 |
| FUT8 | 305.55 | 590.72 | 0.95 | 0.071815941 |
| FBLN1 | 2984.12 | 5768.19 | 0.95 | 0.075683003 |
| NOL4L | 493.89 | 954.52 | 0.95 | 0.097339815 |
| PPAP2B | 198.05 | 382.04 | 0.95 | 0.083979307 |
| CD2AP | 4093.07 | 7811.41 | 0.93 | 0.035984267 |
| CHMP4B | 2474.02 | 4719.35 | 0.93 | 0.045649281 |
| ARHGEF10L | 1006.39 | 1917.56 | 0.93 | 0.055747335 |
| TC2N | 1635.98 | 3111.83 | 0.93 | 0.068917181 |
| SLC12A7 | 890.45 | 1682.63 | 0.92 | 0.088194167 |
| PNPLA2 | 1214.02 | 2289.97 | 0.92 | 0.070901384 |
| RAB27B | 1919.58 | 3613.41 | 0.91 | 0.051593103 |
| GOLM1 | 1195.22 | 2242.92 | 0.91 | 0.044519154 |
| ERBB3 | 1394.57 | 2615.20 | 0.91 | 0.067082341 |
| TMC4 | 1535.64 | 2859.41 | 0.90 | 0.048616174 |
| TTC39A | 273.25 | 508.28 | 0.90 | 0.078989612 |
| ECHS1 | 1595.72 | 2963.05 | 0.89 | 0.075493558 |
| GCN1L1 | 4067.08 | 7501.68 | 0.88 | 0.092135466 |
| PARP12 | 557.34 | 1016.98 | 0.87 | 0.098867304 |
| PRSS22 | 1297.56 | 2348.78 | 0.86 | 0.087560112 |
| UBL3 | 925.66 | 1628.30 | 0.81 | 0.079319439 |
| RAB3D | 1802.14 | 965.83 | -0.90 | 0.064440959 |
| TFAP2A | 3170.48 | 1671.14 | -0.92 | 0.075210528 |
| NLGN2 | 1323.74 | 695.78 | -0.93 | 0.07332314 |
| DHCR7 | 2460.52 | 1275.67 | -0.95 | 0.073644435 |
| IDI1 | 2318.86 | 1201.27 | -0.95 | 0.086640575 |
| SQLE | 2935.97 | 1511.05 | -0.96 | 0.051126831 |
| DHRS1 | 1956.22 | 1005.78 | -0.96 | 0.062117334 |
| SMIM3 | 332.97 | 170.00 | -0.97 | 0.095973435 |
| THSD4 | 3501.36 | 1782.99 | -0.97 | 0.057691761 |
| FKBP9 | 3491.25 | 1775.20 | -0.98 | 0.057758483 |
| CTDSPL | 3088.03 | 1568.26 | -0.98 | 0.075101424 |
| DUOX1 | 3439.82 | 1722.52 | -1.00 | 0.029017359 |
| BTBD11 | 739.32 | 368.81 | -1.00 | 0.060302771 |
| STXBP1 | 697.28 | 347.07 | -1.01 | 0.061118453 |
| IRF6 | 24208.12 | 12049.13 | -1.01 | 0.083558459 |
| LOC100506119 | 587.80 | 291.09 | -1.01 | 0.071220291 |
| CYBRD1 | 453.43 | 222.28 | -1.03 | 0.061629009 |
| CES2 | 4469.25 | 2189.96 | -1.03 | 0.019146307 |
| CD44 | 22353.98 | 10756.55 | -1.06 | 0.030110844 |
| PRRG4 | 1502.28 | 722.55 | -1.06 | 0.027071942 |
| NOL3 | 657.98 | 315.63 | -1.06 | 0.040749809 |
| CDH3 | 19746.16 | 9409.72 | -1.07 | 0.054929938 |
| LRP4 | 479.80 | 228.02 | -1.07 | 0.065268032 |
| SYNE2 | 2610.80 | 1238.82 | -1.08 | 0.089562614 |
| IL17RE | 418.47 | 197.71 | -1.08 | 0.088492397 |
| CD109 | 3994.96 | 1879.80 | -1.09 | 0.00994933 |
| BNC1 | 4409.49 | 2067.59 | -1.09 | 0.099524348 |
| LEPR | 814.64 | 379.11 | -1.10 | 0.039880767 |
| CERCAM | 2170.74 | 1004.90 | -1.11 | 0.012067092 |
| LYST | 2585.08 | 1189.28 | -1.12 | 0.057691761 |
| SLC9A9 | 195.52 | 88.96 | -1.14 | 0.084225392 |
| PLTP | 2250.26 | 1009.30 | -1.16 | 0.005057013 |
| MSMO1 | 5198.41 | 2325.68 | -1.16 | 0.00603459 |
| CCND2 | 6073.45 | 2697.64 | -1.17 | 0.003171651 |
| NRBP2 | 2396.47 | 1064.06 | -1.17 | 0.006463922 |
| SESN3 | 2749.06 | 1209.21 | -1.18 | 0.004286756 |
| CLIP4 | 2706.37 | 1183.24 | -1.19 | 0.034705284 |
| TMOD2 | 1169.81 | 510.58 | -1.20 | 0.045733149 |
| MYO1B | 17804.98 | 7765.24 | -1.20 | 0.005859901 |
| ST6GALNAC2 | 2848.44 | 1234.60 | -1.21 | 0.032475694 |
| KDELC2 | 306.61 | 132.89 | -1.21 | 0.030369714 |
| PRSS8 | 2593.52 | 1123.81 | -1.21 | 0.034944667 |
| TINCR | 585.47 | 250.22 | -1.23 | 0.043431338 |
| STARD4 | 1471.85 | 627.20 | -1.23 | 0.003821526 |
| PERP | 78765.59 | 33338.57 | -1.24 | 0.064556262 |
| MIR210HG | 1241.33 | 525.11 | -1.24 | 0.049952581 |
| BCAM | 4340.19 | 1834.05 | -1.24 | 0.009771852 |
| XG | 359.59 | 149.87 | -1.26 | 0.011194317 |
| ALDOC | 1069.23 | 444.97 | -1.26 | 0.005381909 |
| PAPLN | 1304.18 | 538.74 | -1.28 | 0.04074182 |
| REEP2 | 125.20 | 51.67 | -1.28 | 0.099524348 |
| SLC6A10P | 282.96 | 116.67 | -1.28 | 0.02560049 |
| IL6R | 1455.04 | 597.85 | -1.28 | 0.004304995 |
| DUOXA1 | 1518.97 | 619.13 | -1.29 | 0.022810105 |
| LSS | 2354.85 | 953.22 | -1.30 | 0.002331158 |
| FANCE | 456.92 | 184.67 | -1.31 | 0.063375803 |
| ANKRD35 | 263.25 | 105.52 | -1.32 | 0.098612901 |
| HMGCS1 | 3394.43 | 1350.32 | -1.33 | 0.018307176 |
| MTHFD2L | 141.44 | 56.25 | -1.33 | 0.062382901 |
| CCBL1 | 212.46 | 84.14 | -1.34 | 0.026838458 |
| SLC9A3 | 201.80 | 78.98 | -1.35 | 0.088453466 |
| ADM | 3007.85 | 1174.87 | -1.36 | 0.040425943 |
| MAP3K6 | 1205.37 | 468.95 | -1.36 | 0.039720853 |
| SCARA3 | 208.75 | 80.41 | -1.38 | 0.020065776 |
| CPA4 | 500.26 | 192.37 | -1.38 | 0.00368199 |
| FBXO17 | 493.97 | 188.50 | -1.39 | 0.018459058 |
| RIN1 | 1333.23 | 507.98 | -1.39 | 0.00054539 |
| CDKN2D | 223.63 | 84.85 | -1.40 | 0.033708954 |
| DSP | 87779.19 | 32595.88 | -1.43 | 0.07332314 |
| C10orf55 | 355.74 | 130.24 | -1.45 | 0.010606472 |
| PRODH | 1120.60 | 407.04 | -1.46 | 0.001254443 |
| PAK6 | 375.71 | 135.26 | -1.47 | 0.011205168 |
| NFASC | 103.23 | 36.25 | -1.51 | 0.090266869 |
| SULF1 | 180.44 | 63.21 | -1.51 | 0.054160658 |
| CALML3 | 375.51 | 131.47 | -1.51 | 0.05563554 |
| S1PR5 | 707.49 | 243.23 | -1.54 | 0.008277375 |
| NCR3LG1 | 133.89 | 45.57 | -1.55 | 0.031886791 |
| CXCL5 | 137.53 | 45.96 | -1.58 | 0.059856874 |
| SH3D21 | 692.89 | 227.94 | -1.60 | 0.003964547 |
| PROCR | 403.33 | 132.20 | -1.61 | 0.000622248 |
| H19 | 8138.88 | 2657.70 | -1.61 | 0.090510955 |
| C1S | 140.51 | 45.10 | -1.64 | 0.012247277 |
| STRIP2 | 558.77 | 177.49 | -1.65 | 0.000198909 |
| AS3MT | 143.94 | 44.49 | -1.69 | 0.007231863 |
| FAT2 | 21142.78 | 6510.12 | -1.70 | 0.000968929 |
| CTSV | 656.53 | 201.15 | -1.71 | 0.002211791 |
| CAMKK1 | 123.56 | 37.45 | -1.72 | 0.054929938 |
| MCTP1 | 112.35 | 33.80 | -1.73 | 0.012914616 |
| HSD17B3 | 70.51 | 20.87 | -1.76 | 0.043211695 |
| TRIM7 | 380.24 | 111.76 | -1.77 | 0.007231863 |
| LTB4R | 951.52 | 278.41 | -1.77 | 6.54E-05 |
| FTL | 32288.49 | 9390.84 | -1.78 | 0.000181015 |
| SIRPB2 | 104.79 | 30.35 | -1.79 | 0.02389953 |
| THEMIS2 | 331.60 | 95.93 | -1.79 | 0.024071746 |
| CT62 | 122.04 | 35.07 | -1.80 | 0.028388104 |
| NEB | 165.65 | 46.00 | -1.85 | 0.018591983 |
| CA2 | 1732.61 | 474.71 | -1.87 | 0.018371354 |
| MAMDC2 | 87.42 | 23.88 | -1.87 | 0.044621705 |
| IL12RB2 | 100.30 | 27.01 | -1.89 | 0.006261591 |
| C10orf99 | 3225.29 | 867.88 | -1.89 | 0.057691761 |
| LTB4R2 | 398.36 | 106.84 | -1.90 | 0.000270896 |
| MFI2 | 411.64 | 110.41 | -1.90 | 0.073191981 |
| USH1G | 188.83 | 49.00 | -1.95 | 0.010470875 |
| PHYHIP | 125.46 | 32.11 | -1.97 | 0.026838458 |
| ARHGEF4 | 2975.05 | 753.47 | -1.98 | 4.32E-05 |
| LDLR | 3350.57 | 830.42 | -2.01 | 4.50E-06 |
| CLCA2 | 12414.09 | 3062.64 | -2.02 | 1.71E-06 |
| PCP4L1 | 197.66 | 48.36 | -2.03 | 0.084009353 |
| ARSI | 63.96 | 15.56 | -2.04 | 0.06494046 |
| CD164L2 | 79.82 | 19.29 | -2.05 | 0.006924308 |
| CXCL2 | 393.84 | 93.36 | -2.08 | 0.088818719 |
| INSIG1 | 1057.25 | 249.59 | -2.08 | 2.17E-05 |
| B4GALNT2 | 86.00 | 19.91 | -2.11 | 0.012247277 |
| UAP1L1 | 247.01 | 56.90 | -2.12 | 0.047489471 |
| COL16A1 | 3587.05 | 817.20 | -2.13 | 0.005045439 |
| KRT5 | 494738.09 | 110529.74 | -2.16 | 0.040506669 |
| EMP3 | 108.84 | 24.03 | -2.18 | 0.018698495 |
| CKB | 1381.23 | 301.07 | -2.20 | 0.006585993 |
| SCD | 31171.38 | 6586.41 | -2.24 | 3.94E-09 |
| APOE | 2469.36 | 512.30 | -2.27 | 1.22E-08 |
| KLK10 | 530.46 | 108.91 | -2.28 | 2.17E-05 |
| TNNT2 | 194.08 | 39.47 | -2.30 | 4.16E-05 |
| PTGS1 | 236.59 | 48.03 | -2.30 | 0.001004612 |
| CWH43 | 1244.37 | 248.85 | -2.32 | 0.000223699 |
| ENO2 | 832.57 | 166.36 | -2.32 | 0.003654888 |
| LEMD1 | 46.02 | 9.18 | -2.33 | 0.04074182 |
| RBM20 | 37.57 | 7.42 | -2.34 | 0.062396387 |
| CASP14 | 8453.02 | 1633.74 | -2.37 | 0.009294209 |
| ECM1 | 318.34 | 60.22 | -2.40 | 5.47E-06 |
| VSNL1 | 313.86 | 58.77 | -2.42 | 0.005195922 |
| TP53AIP1 | 98.48 | 17.79 | -2.47 | 0.001227407 |
| SCN4B | 341.13 | 61.28 | -2.48 | 7.08E-05 |
| SAA1 | 1726.84 | 305.34 | -2.50 | 0.03007605 |
| CDHR1 | 622.25 | 108.69 | -2.52 | 0.02560049 |
| RRAD | 253.05 | 44.19 | -2.52 | 6.46E-07 |
| WFDC21P | 33.34 | 5.59 | -2.58 | 0.080253733 |
| ICAM5 | 74.19 | 12.02 | -2.63 | 0.002260421 |
| CYP2W1 | 39.61 | 6.35 | -2.64 | 0.024602431 |
| PCSK9 | 501.04 | 80.25 | -2.64 | 1.73E-09 |
| SERPINB7 | 1637.56 | 261.42 | -2.65 | 0.001670045 |
| KLK8 | 827.27 | 126.01 | -2.71 | 7.88E-05 |
| LOC650226 | 30.08 | 4.34 | -2.79 | 0.065437256 |
| COL8A2 | 27.80 | 3.89 | -2.84 | 0.060526244 |
| ADAMTSL4 | 1848.80 | 247.85 | -2.90 | 8.14E-09 |
| CIDEB | 40.39 | 5.21 | -2.95 | 0.008277375 |
| HKDC1 | 87.27 | 9.82 | -3.15 | 0.001670045 |
| FOXN1 | 152.89 | 16.38 | -3.22 | 0.009771852 |
| CD177 | 42.18 | 3.59 | -3.55 | 0.005172485 |
| CRYAB | 459.14 | 38.99 | -3.56 | 0.00296997 |
| CHI3L2 | 18.25 | 1.42 | -3.68 | 0.088943857 |
| KLK7 | 302.57 | 21.76 | -3.80 | 0.000150784 |
| KLK9 | 49.63 | 3.30 | -3.91 | 0.000387721 |
| FGFBP1 | 2087.18 | 110.57 | -4.24 | 0.00036426 |
| KLK5 | 311.62 | 12.14 | -4.68 | 2.59E-16 |
| LOC100093631 | 182.70 | 1.25 | -7.19 | 2.02E-10 |

Supplementary Table 2. GO terms

| **GO term** | **Description** | P-value | FDR q-value | Enrichment (N, B, n, b) | Genes |
| --- | --- | --- | --- | --- | --- |
| GO:0044255 | cellular lipid metabolic process | 1.16E-05 | 1.66E-01 | 3.20 (18299,903,114,18) |  |
|  |  |  |  |  | CYP4F12 - cytochrome p450, family 4, subfamily f, polypeptide 12 |
|  |  |  |  |  | ACSBG1 - acyl-coa synthetase bubblegum family member 1 |
|  |  |  |  |  | SPTSSB - serine palmitoyltransferase, small subunit b |
|  |  |  |  |  | ACOX1 - acyl-coa oxidase 1, palmitoyl |
|  |  |  |  |  | ACSL5 - acyl-coa synthetase long-chain family member 5 |
|  |  |  |  |  | PTGR1 - prostaglandin reductase 1 |
|  |  |  |  |  | STS - steroid sulfatase (microsomal), isozyme s |
|  |  |  |  |  | HPGD - hydroxyprostaglandin dehydrogenase 15-(nad) |
|  |  |  |  |  | SLC44A4 - solute carrier family 44, member 4 |
|  |  |  |  |  | PPAP2A - phosphatidic acid phosphatase type 2a |
|  |  |  |  |  | STRA6 - stimulated by retinoic acid 6 |
|  |  |  |  |  | HMGCS2 - 3-hydroxy-3-methylglutaryl-coa synthase 2 (mitochondrial) |
|  |  |  |  |  | ACER2 - alkaline ceramidase 2 |
|  |  |  |  |  | ERBB3 - v-erb-b2 avian erythroblastic leukemia viral oncogene homolog 3 |
|  |  |  |  |  | CHKA - choline kinase alpha |
|  |  |  |  |  | PNPLA2 - patatin-like phospholipase domain containing 2 |
|  |  |  |  |  | ACSM3 - acyl-coa synthetase medium-chain family member 3 |
|  |  |  |  |  | ACADSB - acyl-coa dehydrogenase, short/branched chain |
| [GO:0006629](http://www.godatabase.org/cgi-bin/amigo/go.cgi?query=GO:0006629&view=details) | lipid metabolic process | 8.13E-05 | 5.82E-01 | 2.66 (18299,1146,114,19) |  |
|  |  |  |  |  | CYP4F12 - cytochrome p450, family 4, subfamily f, polypeptide 12 |
|  |  |  |  |  | ACSBG1 - acyl-coa synthetase bubblegum family member 1 |
|  |  |  |  |  | SPTSSB - serine palmitoyltransferase, small subunit b |
|  |  |  |  |  | CD36 - cd36 molecule (thrombospondin receptor) |
|  |  |  |  |  | ACOX1 - acyl-coa oxidase 1, palmitoyl |
|  |  |  |  |  | ACSL5 - acyl-coa synthetase long-chain family member 5 |
|  |  |  |  |  | PTGR1 - prostaglandin reductase 1 |
|  |  |  |  |  | STS - steroid sulfatase (microsomal), isozyme s |
|  |  |  |  |  | HPGD - hydroxyprostaglandin dehydrogenase 15-(nad) |
|  |  |  |  |  | SLC44A4 - solute carrier family 44, member 4 |
|  |  |  |  |  | PPAP2A - phosphatidic acid phosphatase type 2a |
|  |  |  |  |  | STRA6 - stimulated by retinoic acid 6 |
|  |  |  |  |  | HMGCS2 - 3-hydroxy-3-methylglutaryl-coa synthase 2 (mitochondrial) |
|  |  |  |  |  | ACER2 - alkaline ceramidase 2 |
|  |  |  |  |  | ERBB3 - v-erb-b2 avian erythroblastic leukemia viral oncogene homolog 3 |
|  |  |  |  |  | CHKA - choline kinase alpha |
|  |  |  |  |  | PNPLA2 - patatin-like phospholipase domain containing 2 |
|  |  |  |  |  | ACSM3 - acyl-coa synthetase medium-chain family member 3 |
|  |  |  |  |  | ACADSB - acyl-coa dehydrogenase, short/branched chain |
| [GO:0030540](http://www.godatabase.org/cgi-bin/amigo/go.cgi?query=GO:0030540&view=details) | female genitalia development | 8.15E-05 | 3.89E-01 | 34.40 (18299,14,114,3) |  |
|  |  |  |  |  | STRA6 - stimulated by retinoic acid 6 |
|  |  |  |  |  | TBX3 - t-box 3 |
|  |  |  |  |  | MERTK - c-mer proto-oncogene tyrosine kinase |
| [GO:0070543](http://www.godatabase.org/cgi-bin/amigo/go.cgi?query=GO:0070543&view=details) | response to linoleic acid | 1.15E-04 | 4.11E-01 | 107.01 (18299,3,114,2) |  |
|  |  |  |  |  | HMGCS2 - 3-hydroxy-3-methylglutaryl-coa synthase 2 (mitochondrial) |
|  |  |  |  |  | CD36 - cd36 molecule (thrombospondin receptor) |
| [GO:0032787](http://www.godatabase.org/cgi-bin/amigo/go.cgi?query=GO:0032787&view=details) | monocarboxylic acid metabolic process | 2.90E-04 | 8.30E-01 | 3.53 (18299,500,114,11) |  |
|  |  |  |  |  | CYP4F12 - cytochrome p450, family 4, subfamily f, polypeptide 12 |
|  |  |  |  |  | ACSBG1 - acyl-coa synthetase bubblegum family member 1 |
|  |  |  |  |  | IDO1 - indoleamine 2,3-dioxygenase 1 |
|  |  |  |  |  | MCCC1 - methylcrotonoyl-coa carboxylase 1 (alpha) |
|  |  |  |  |  | ACOX1 - acyl-coa oxidase 1, palmitoyl |
|  |  |  |  |  | ACSL5 - acyl-coa synthetase long-chain family member 5 |
|  |  |  |  |  | PTGR1 - prostaglandin reductase 1 |
|  |  |  |  |  | HPGD - hydroxyprostaglandin dehydrogenase 15-(nad) |
|  |  |  |  |  | ACSM3 - acyl-coa synthetase medium-chain family member 3 |
|  |  |  |  |  | BPGM - 2,3-bisphosphoglycerate mutase |
|  |  |  |  |  | ACADSB - acyl-coa dehydrogenase, short/branched chain |
| [GO:0050891](http://www.godatabase.org/cgi-bin/amigo/go.cgi?query=GO:0050891&view=details) | multicellular organismal water homeostasis | 3.81E-04 | 9.10E-01 | 11.67 (18299,55,114,4) |  |
|  |  |  |  |  | AQP3 - aquaporin 3 (gill blood group) |
|  |  |  |  |  | CYP4F12 - cytochrome p450, family 4, subfamily f, polypeptide 12 |
|  |  |  |  |  | SCNN1B - sodium channel, non-voltage-gated 1, beta subunit |
|  |  |  |  |  | CLDN4 - claudin 4 |
| [GO:0006693](http://www.godatabase.org/cgi-bin/amigo/go.cgi?query=GO:0006693&view=details) | prostaglandin metabolic process | 4.33E-04 | 8.86E-01 | 20.06 (18299,24,114,3) |  |
|  |  |  |  |  | ACOX1 - acyl-coa oxidase 1, palmitoyl |
|  |  |  |  |  | PTGR1 - prostaglandin reductase 1 |
|  |  |  |  |  | HPGD - hydroxyprostaglandin dehydrogenase 15-(nad) |
| [GO:0006692](http://www.godatabase.org/cgi-bin/amigo/go.cgi?query=GO:0006692&view=details) | prostanoid metabolic process | 4.33E-04 | 7.75E-01 | 20.06 (18299,24,114,3) |  |
|  |  |  |  |  | ACOX1 - acyl-coa oxidase 1, palmitoyl |
|  |  |  |  |  | PTGR1 - prostaglandin reductase 1 |
|  |  |  |  |  | HPGD - hydroxyprostaglandin dehydrogenase 15-(nad) |
| [GO:0007162](http://www.godatabase.org/cgi-bin/amigo/go.cgi?query=GO:0007162&view=details) | negative regulation of cell adhesion | 4.57E-04 | 7.27E-01 | 5.11 (18299,220,114,7) |  |
|  |  |  |  |  | TNFRSF21 - tumor necrosis factor receptor superfamily, member 21 |
|  |  |  |  |  | IRF1 - interferon regulatory factor 1 |
|  |  |  |  |  | IDO1 - indoleamine 2,3-dioxygenase 1 |
|  |  |  |  |  | ACER2 - alkaline ceramidase 2 |
|  |  |  |  |  | ERBB3 - v-erb-b2 avian erythroblastic leukemia viral oncogene homolog 3 |
|  |  |  |  |  | BCL6 - b-cell cll/lymphoma 6 |
|  |  |  |  |  | FBLN1 - fibulin 1 |
| [GO:0048871](http://www.godatabase.org/cgi-bin/amigo/go.cgi?query=GO:0048871&view=details) | multicellular organismal homeostasis | 4.84E-04 | 6.92E-01 | 7.72 (18299,104,114,5) |  |
|  |  |  |  |  | AQP3 - aquaporin 3 (gill blood group) |
|  |  |  |  |  | CYP4F12 - cytochrome p450, family 4, subfamily f, polypeptide 12 |
|  |  |  |  |  | SCNN1B - sodium channel, non-voltage-gated 1, beta subunit |
|  |  |  |  |  | CLDN4 - claudin 4 |
|  |  |  |  |  | SLC40A1 - solute carrier family 40 (iron-regulated transporter), member 1 |
| [GO:0006631](http://www.godatabase.org/cgi-bin/amigo/go.cgi?query=GO:0006631&view=details) | fatty acid metabolic process | 4.99E-04 | 6.49E-01 | 4.38 (18299,293,114,8) |  |
|  |  |  |  |  | CYP4F12 - cytochrome p450, family 4, subfamily f, polypeptide 12 |
|  |  |  |  |  | ACSBG1 - acyl-coa synthetase bubblegum family member 1 |
|  |  |  |  |  | ACOX1 - acyl-coa oxidase 1, palmitoyl |
|  |  |  |  |  | ACSL5 - acyl-coa synthetase long-chain family member 5 |
|  |  |  |  |  | HPGD - hydroxyprostaglandin dehydrogenase 15-(nad) |
|  |  |  |  |  | PTGR1 - prostaglandin reductase 1 |
|  |  |  |  |  | ACSM3 - acyl-coa synthetase medium-chain family member 3 |
|  |  |  |  |  | ACADSB - acyl-coa dehydrogenase, short/branched chain |
| [GO:0097070](http://www.godatabase.org/cgi-bin/amigo/go.cgi?query=GO:0097070&view=details) | ductus arteriosus closure | 5.68E-04 | 6.77E-01 | 53.51 (18299,6,114,2) |  |
|  |  |  |  |  | STRA6 - stimulated by retinoic acid 6 |
|  |  |  |  |  | HPGD - hydroxyprostaglandin dehydrogenase 15-(nad) |
| [GO:0035023](http://www.godatabase.org/cgi-bin/amigo/go.cgi?query=GO:0035023&view=details) | regulation of Rho protein signal transduction | 5.75E-04 | 6.33E-01 | 7.43 (18299,108,114,5) |  |
|  |  |  |  |  | MCF2L - mcf.2 cell line derived transforming sequence-like |
|  |  |  |  |  | FGD3 - fyve, rhogef and ph domain containing 3 |
|  |  |  |  |  | ARHGEF10L - rho guanine nucleotide exchange factor (gef) 10-like |
|  |  |  |  |  | BCL6 - b-cell cll/lymphoma 6 |
|  |  |  |  |  | PLEKHG6 - pleckstrin homology domain containing, family g (with rhogef domain) member 6 |
| [GO:0019915](http://www.godatabase.org/cgi-bin/amigo/go.cgi?query=GO:0019915&view=details) | lipid storage | 6.18E-04 | 6.31E-01 | 17.84 (18299,27,114,3) |  |
|  |  |  |  |  | PLIN2 - perilipin 2 |
|  |  |  |  |  | CD36 - cd36 molecule (thrombospondin receptor) |
|  |  |  |  |  | PNPLA2 - patatin-like phospholipase domain containing 2 |
| [GO:0000038](http://www.godatabase.org/cgi-bin/amigo/go.cgi?query=GO:0000038&view=details) | very long-chain fatty acid metabolic process | 7.65E-04 | 7.30E-01 | 16.61 (18299,29,114,3) |  |
|  |  |  |  |  | CYP4F12 - cytochrome p450, family 4, subfamily f, polypeptide 12 |
|  |  |  |  |  | ACSBG1 - acyl-coa synthetase bubblegum family member 1 |
|  |  |  |  |  | ACOX1 - acyl-coa oxidase 1, palmitoyl |
| [GO:0030104](http://www.godatabase.org/cgi-bin/amigo/go.cgi?query=GO:0030104&view=details) | water homeostasis | 8.09E-04 | 7.24E-01 | 9.58 (18299,67,114,4) |  |
|  |  |  |  |  | AQP3 - aquaporin 3 (gill blood group) |
|  |  |  |  |  | CYP4F12 - cytochrome p450, family 4, subfamily f, polypeptide 12 |
|  |  |  |  |  | SCNN1B - sodium channel, non-voltage-gated 1, beta subunit |
|  |  |  |  |  | CLDN4 - claudin 4 |
| [GO:0055078](http://www.godatabase.org/cgi-bin/amigo/go.cgi?query=GO:0055078&view=details) | sodium ion homeostasis | 8.46E-04 | 7.12E-01 | 16.05 (18299,30,114,3) |  |
|  |  |  |  |  | CYP4F12 - cytochrome p450, family 4, subfamily f, polypeptide 12 |
|  |  |  |  |  | SCNN1B - sodium channel, non-voltage-gated 1, beta subunit |
|  |  |  |  |  | ATP1B1 - atpase, na+/k+ transporting, beta 1 polypeptide |
| [GO:0051250](http://www.godatabase.org/cgi-bin/amigo/go.cgi?query=GO:0051250&view=details) | negative regulation of lymphocyte activation | 8.59E-04 | 6.83E-01 | 6.80 (18299,118,114,5) |  |
|  |  |  |  |  | TNFRSF21 - tumor necrosis factor receptor superfamily, member 21 |
|  |  |  |  |  | IRF1 - interferon regulatory factor 1 |
|  |  |  |  |  | IDO1 - indoleamine 2,3-dioxygenase 1 |
|  |  |  |  |  | MERTK - c-mer proto-oncogene tyrosine kinase |
|  |  |  |  |  | BCL6 - b-cell cll/lymphoma 6 |
| [GO:0046578](http://www.godatabase.org/cgi-bin/amigo/go.cgi?query=GO:0046578&view=details) | regulation of Ras protein signal transduction | 9.78E-04 | 7.37E-01 | 5.29 (18299,182,114,6) |  |
|  |  |  |  |  | MCF2L - mcf.2 cell line derived transforming sequence-like |
|  |  |  |  |  | FGD3 - fyve, rhogef and ph domain containing 3 |
|  |  |  |  |  | ARHGEF10L - rho guanine nucleotide exchange factor (gef) 10-like |
|  |  |  |  |  | RALGPS1 - ral gef with ph domain and sh3 binding motif 1 |
|  |  |  |  |  | BCL6 - b-cell cll/lymphoma 6 |
|  |  |  |  |  | PLEKHG6 - pleckstrin homology domain containing, family g (with rhogef domain) member 6 |

Supplementary Table 3. Expression quartiles for all genes, and genes within 25 kb of FAIRE peaks.

| **Expression** | Control 24 h | Differentiated 24 h | Control 144 h | Differentiated 144 h |
| --- | --- | --- | --- | --- |
| **High** | 6116 | 6178 | 6295 | 6404 |
| **Medium** | 6513 | 6761 | 6511 | 6453 |
| **Low** | 7728 | 7505 | 8045 | 7761 |
| **Zero** | 5252 | 5165 | 4758 | 4991 |
| **Expression** | FAIRE peaks (25kb) Control 24 h | FAIRE peaks (25kb) Differentiated 24 h | FAIRE peaks (25kb) Control 144 h | FAIRE peaks (25kb) Differentiated 144 h |
| **High** | 3804 | 4376 | 4632 | 4234 |
| **Medium** | 3559 | 4012 | 4191 | 3655 |
| **Low** | 3001 | 2908 | 3580 | 2979 |
| **Zero** | 1416 | 1312 | 1445 | 1333 |

Supplementary Table 4. Summary of HOMER motif results for all FAIRE peaks and FAIRE peaks within -/+ 25 kb of differentially expressed genes.

| **Known Results All Control / Differentiation Specific Peaks (p≤0.05, Fold % ≥1.25)** | | | | | | | | |
| --- | --- | --- | --- | --- | --- | --- | --- | --- |
| **Enriched at 24 h in Control** | **P-value** | **% Targets** | | **% Background** | | **%> Background** | | **Fold %** |
| Pax7 (GSE25064) | 1.00E-34 | 2.95 | | 1.63 | | 1.32% | | 1.81 |
| Egr2(GSE34254) | 1.00E-06 | 0.77 | | 0.49 | | 0.28% | | 1.57 |
| Pax7 (GSE25064) | 1.00E-07 | 1.04 | | 0.67 | | 0.37% | | 1.55 |
| Egr1(Zf) (GSE32465) | 1.00E-10 | 3.05 | | 2.26 | | 0.79% | | 1.35 |
| OCT4-SOX2-TCF-NANOG (GSE11431) | 1.00E-18 | 5.53 | | 4.12 | | 1.41% | | 1.34 |
| HNF6(Homeobox) (ERP000394) | 1.00E-29 | 10.64 | | 8.14 | | 2.50% | | 1.31 |
| Phox2a(Homeobox) (GSE31456) | 1.00E-32 | 13.55 | | 10.63 | | 2.92% | | 1.27 |
|  |  |  | |  | |  | |  |
| **Enriched at 24 h in Differentiation-Induced** | **P-value** | **% Targets** | | **% Background** | | **%>Bkg** | | **Fold %** |
| REST-NRSF(Zf)/Jurkat-NRSF-ChIP-Seq/Homer | 0.01 | 0.1 | | 0.05 | | 0.05% | | 2 |
| IRF2(IRF)(GSE36985) | 1.00E-18 | 2.75 | | 1.74 | | 1.01% | | 1.58 |
| ISRE(IRF) (GSE23622) | 1.00E-10 | 1.76 | | 1.14 | | 0.62% | | 1.54 |
| E2F(E2F)/Cell-Cycle-Exp/Homer | 0.01 | 0.32 | | 0.22 | | 0.10% | | 1.45 |
| CTCF-SatelliteElement Homer | 0.01 | 0.37 | | 0.27 | | 0.10% | | 1.37 |
| CTCF (Zf) (Barski et al.) | 1.00E-29 | 10.67 | | 8.05 | | 2.62% | | 1.33 |
| GRHL2(CP2) (GSE46194) | 1.00E-24 | 9.34 | | 7.08 | | 2.26% | | 1.32 |
| BORIS(Zf) (GSE32465) | 1.00E-25 | 10.67 | | 2.4 | | 2.40% | | 1.31 |
| IRF1(IRF) (GSE43036) | 1.00E-07 | 3.43 | | 2.66 | | 0.77% | | 1.29 |
|  |  |  | |  | |  | |  |
| **Enriched at 144 h in Control** | **P-value** | **% Targets** | | **% Background** | | **%>Bkg** | | **Fold %** |
| p53(p53) (GSE15780) | 1.00E-61 | 5.44 | | 3.25 | | 2.19 | | 1.67 |
| p53(p53)/Saos-p53-ChIP-Seq/Homer | 1.00E-61 | 5.44 | | 3.25 | | 2.19 | | 1.67 |
| p53(p53) (GSE11431) | 1.00E-05 | 0.61 | | 0.41 | | 0.20 | | 1.49 |
| p63(p53) (GSE17611) | 1.00E-76 | 11.11 | | 7.56 | | 3.55 | | 1.47 |
|  |  |  | |  | |  | |  |
| **Enriched at 144h in Differentiation-Induced** | **P-value** | **% Targets** | | **% Background** | | **%>Bkg** | | **Fold %** |
| REST-NRSF(Zf)/Jurkat-NRSF-ChIP-Seq/Homer | 1.00E-02 | 0.13 | | 0.05 | | 0.08 | | 2.6 |
| CTCF-SatelliteElement (Barski et al.) | 1.00E-08 | 0.5 | | 0.21 | | 0.29 | | 2.38 |
| RARg (NR) (GSE30538) | 1.00E-05 | 0.36 | | 0.16 | | 0.20 | | 2.25 |
| BORIS(Zf) (GSE32465) | 1.00E-41 | 12.62 | | 8.77 | | 3.85 | | 1.44 |
| CTCF(Zf) (Barski et al.) | 1.00E-39 | 13.6 | | 9.69 | | 3.91 | | 1.4 |
| TR4 (NR/DR1) (GSE24685) | 1.00E-03 | 1.05 | | 0.77 | | 0.28 | | 1.36 |
| ISRE(IRF) (GSE23622) | 1.00E-04 | 1.65 | | 1.24 | | 0.41 | | 1.33 |
| GATA-IR4(Zf) (GSE20898) | 1.00E-04 | 2.12 | | 1.6 | | 0.52 | | 1.33 |
| IRF2(IRF) (GSE36985) | 1.00E-04 | 2.41 | | 1.9 | | 0.51 | | 1.27 |
| GATA-DR4(Zf) (GSE20898) | 1.00E-02 | 1.86 | | 1.49 | | 0.37 | | 1.25 |
| **Known Results Peaks -/+ 25 kb Differentially Expressed Genes (p≤0.05, Fold % ≥1.25)** | | | | | | | | |
| **Enriched at 24 h Around Downregulated** | **P-value** | | **% Targets** | | **% Background** | | **%>Bkg** | **Fold %** |
| Hnf1(Homeobox) (GSE25694) | 1.00E-02 | | 4.63% | | 2.63% | | 2.00% | 1.76 |
| Ets1-distal(ETS) (Barski et al.) | 1.00E-03 | | 10.06% | | 5.99% | | 4.07% | 1.68 |
| Elk4(ETS) (GSE31477) | 1.00E-03 | | 11.67% | | 7.42% | | 4.25% | 1.57 |
| EWS:FLI1-fusion(ETS) (SRA014231) | 1.00E-02 | | 13.68% | | 10.15% | | 3.53% | 1.35 |
| EWS:ERG-fusion(ETS) (SRA014231) | 1.00E-03 | | 20.93% | | 15.53% | | 5.40% | 1.35 |
| Fli1(ETS) (GSE20898) | 1.00E-02 | | 23.34% | | 18.14% | | 5.20% | 1.29 |
| GABPA(ETS) (GSE17954) | 1.00E-02 | | 18.91% | | 14.94% | | 3.97% | 1.27 |
| Jun-AP1(bZIP) (GSE31477) | 1.00E-02 | | 22.33% | | 17.67% | | 4.66% | 1.26 |
| BATF(bZIP) (GSE39756) | 1.00E-03 | | 42.45% | | 34.55% | | 7.90% | 1.23 |
|  |  | |  | |  | |  |  |
| **Enriched at 24 h Around Upregulated** | **P-value** | | **% Targets** | | **% Background** | | **%>Bkg** | **Fold %** |
| GRHL2 (GSE46194) | 1.00E-07 | | 13.37 | | 6.99 | | 6.38 | 1.91 |
| GATA-IR3(Zf) (GSE20898) | 1.00E-02 | | 3.39 | | 1.83 | | 1.56 | 1.85 |
| PPARE(NR/DR1) (GSE13511) |  | | 16.40 | | 11.71 | | 4.69 | 1.4 |
| CEBP(bZIP) (GSE21512) | 1.00E-03 | | 17.47 | | 12.63 | | 4.84 | 1.38 |
| Foxh1(Forkhead) (GSE29422) | 1.00E-02 | | 11.41 | | 8.32 | | 3.09 | 1.37 |
| HOXA9 (GSE33509) | 1.00E-02 | | 13.90 | | 10.16 | | 3.74 | 1.37 |
|  |  | |  | |  | |  |  |
| **Enriched at 144 h Around Downregulated** | **P-value** | | **% Targets** | | **% Background** | | **%>Bkg** | **Fold %** |
| NFkB-p65-Rel(RHD) (GSE23622) | 1.00E-02 | | 1.63% | | 0.84% | | 0.79% | 1.94 |
| NF1:FOXA1 (GSE27824) | 1.00E-02 | | 1.82% | | 0.96% | | 0.86% | 1.9 |
| ETS:RUNX (GSE17954) | 1.00E-02 | | 2.36% | | 1.38% | | 0.98% | 1.71 |
| Hnf1(Homeobox) (GSE25694) | 1.00E-02 | | 9.36% | | 6.38% | | 1.26% | 1.46 |
| Ets1-distal(ETS) (Barski et al.) | 1.00E-04 | | 4.00% | | 2.74% | | 2.98% | 1.47 |
| Elk4(ETS) (GSE31477) | 1.00E-02 | | 10.45% | | 7.88% | | 2.57% | 1.33 |
| ETS(ETS)(Homer) | 1.00E-02 | | 6.99% | | 5.29% | | 1.70% | 1.32 |
| Elk1(ETS) (GSE31477) | 1.00E-02 | | 10.81% | | 8.28% | | 2.53% | 1.31 |
| STAT6 (GSE38377) | 1.00E-02 | | 11.08% | | 8.54% | | 2.54% | 1.3 |
| STAT6(Stat) (GSE22104) | 1.00E-02 | | 11.08% | | 8.71% | | 2.37% | 1.27 |
| PU.1(ETS) (GSE21512) | 1.00E-02 | | 11.63% | | 9.25% | | 2.38% | 1.26 |
| p63(p53) (GSE17611) | 1.00E-02 | | 9.90% | | 7.89% | | 2.01% | 1.25 |
| ELF1(ETS) (SRA014231) | 1.00E-02 | | 9.99% | | 7.98% | | 2.01% | 1.25 |
|  |  | |  | |  | |  |  |
| **Enriched at 144h Around Upregulated** | **P-value** | | **% Targets** | | **% Background** | | **%>Bkg** | **Fold %** |
| LXRE (GSE21512) | 1.00E-02 | | 1.42% | | 0.57% | | 0.85 | 2.49 |
| GRHL2 (GSE46194) | 1.00E-15 | | 14.60% | | 6.98% | | 7.62 | 2.09 |
| GATA-IR3(Zf) (GSE20898) | 1.00E-03 | | 3.70% | | 2.02% | | 1.68 | 1.83 |
| FOXP1 (GSE31006) | 1.00E-03 | | 10.57% | | 7.27% | | 3.30 | 1.45 |
| NF1(CTF) (Unpublished) | 1.00E-03 | | 11.44% | | 8.03% | | 3.41% | 1.42 |
| FOXA1 (GSE26831) | 1.00E-05 | | 23.42% | | 17.21% | | 6.21% | 1.36 |
| FOXA1 (GSE27824) | 1.00E-06 | | 27.45% | | 20.52% | | 6.93% | 1.34 |
| Foxa2 (GSE25694) | 1.00E-04 | | 18.41% | | 13.82% | | 4.59% | 1.33 |
| ELF5(GSE30407) | 1.00E-04 | | 19.50% | | 14.79% | | 4.71% | 1.32 |
| CEBP (GSE21512) | 1.00E-03 | | 17.21% | | 13.53% | | 3.68% | 1.27 |
