# Supplementary material for: Heterarchy of transcription factors driving basal and luminal cell phenotypes in human urothelium
Source: Cell Death Differ. 2017 Mar 10;24(5):809–18. doi: 10.1038/cdd.2017.10 (PMC5423105; doi:10.1038/cdd.2017.10)
Supplement: Supplementary Tables 5-12 [file cdd201710x8.docx]

Supplementary Table 5. Motifs enriched in all FAIRE peaks unique to control cells at 24 h.

| All Peaks Unique to 24 Hour Control | P-value | Log P-value | q-value (Benjamini) | # of Target Sequences with Motif (of 17101) | % of Target Sequences with Motif | # of Background Sequences with Motif(of 27416) | % of Background Sequences with Motif | % Target >Background | Fold Target >Background |
| --- | --- | --- | --- | --- | --- | --- | --- | --- | --- |
| **Pax7(Paired/Homeobox)/Myoblast-Pax7-ChIP-Seq(GSE25064)/Homer** | **1.00E-34** | **-7.91E+01** | **0** | **505** | **2.95%** | **446.5** | **1.63%** | **1.32%** | **1.81** |
| **Egr2/Thymocytes-Egr2-ChIP-Seq(GSE34254)/Homer** | **1.00E-06** | **-1.39E+01** | **0** | **131** | **0.77%** | **134.8** | **0.49%** | **0.28%** | **1.57** |
| **Pax7-longest(Paired/Homeobox)/Myoblast-Pax7-ChIP-Seq(GSE25064)/Homer** | **1.00E-07** | **-1.75E+01** | **0** | **178** | **1.04%** | **184.9** | **0.67%** | **0.37%** | **1.55** |
| **Egr1(Zf)/K562-Egr1-ChIP-Seq(GSE32465)/Homer** | **1.00E-10** | **-2.47E+01** | **0** | **521** | **3.05%** | **618.6** | **2.26%** | **0.79%** | **1.35** |
| **OCT4-SOX2-TCF-NANOG((POU/Homeobox/HMG)/mES-Oct4-ChIP-Seq(GSE11431)/Homer** | **1.00E-18** | **-4.22E+01** | **0** | **946** | **5.53%** | **1129.1** | **4.12%** | **1.41%** | **1.34** |
| **HNF6(Homeobox)/Liver-Hnf6-ChIP-Seq(ERP000394)/Homer** | **1.00E-29** | **-6.90E+01** | **0** | **1820** | **10.64%** | **2232.4** | **8.14%** | **2.50%** | **1.31** |
| **Phox2a(Homeobox)/Neuron-Phox2a-ChIP-Seq(GSE31456)/Homer** | **1.00E-32** | **-7.47E+01** | **0** | **2318** | **13.55%** | **2915.8** | **10.63%** | **2.92%** | **1.27** |
| CArG(MADS)/PUER-Srf-ChIP-Seq(Sullivan et al.)/Homer | 1.00E-08 | -1.89E+01 | 0 | 709 | 4.15% | 914.6 | 3.34% | 0.81% | 1.24 |
| Hnf1(Homeobox)/Liver-Foxa2-Chip-Seq(GSE25694)/Homer | 1.00E-09 | -2.26E+01 | 0 | 894 | 5.23% | 1158.1 | 4.22% | 1.01% | 1.24 |
| EBF(EBF)/proBcell-EBF-ChIP-Seq(GSE21978)/Homer | 1.00E-02 | -5.01E+00 | 0.0202 | 155 | 0.91% | 202.3 | 0.74% | 0.17% | 1.23 |
| PAX3:FKHR-fusion(Paired/Homeobox)/Rh4-PAX3:FKHR-ChIP-Seq(GSE19063)/Homer | 1.00E-07 | -1.69E+01 | 0 | 736 | 4.30% | 966.3 | 3.52% | 0.78% | 1.22 |
| Elk1(ETS)/Hela-Elk1-ChIP-Seq(GSE31477)/Homer | 1.00E-09 | -2.24E+01 | 0 | 984 | 5.75% | 1290.7 | 4.71% | 1.04% | 1.22 |
| Elk4(ETS)/Hela-Elk4-ChIP-Seq(GSE31477)/Homer | 1.00E-08 | -1.97E+01 | 0 | 961 | 5.62% | 1275.5 | 4.65% | 0.97% | 1.21 |
| Ets1-distal(ETS)/CD4+-PolII-ChIP-Seq(Barski et al.)/Homer | 1.00E-07 | -1.65E+01 | 0 | 908 | 5.31% | 1221.2 | 4.45% | 0.86% | 1.19 |
| Srebp1a(HLH)/HepG2-Srebp1a-ChIP-Seq(GSE31477)/Homer | 1.00E-02 | -6.00E+00 | 0.0079 | 255 | 1.49% | 341.5 | 1.25% | 0.24% | 1.19 |
| Lhx2(Homeobox)/HFSC-Lhx2-ChIP-Seq(GSE48068)/Homer | 1.00E-37 | -8.73E+01 | 0 | 4741 | 27.72% | 6428.2 | 23.45% | 4.27% | 1.18 |
| TATA-Box(TBP)/Promoter/Homer | 1.00E-32 | -7.45E+01 | 0 | 4274 | 24.99% | 5809.7 | 21.19% | 3.80% | 1.18 |
| Hoxb4(Homeobox)/ES-Hoxb4-ChIP-Seq(GSE34014)/Homer | 1.00E-04 | -1.03E+01 | 0.0001 | 667 | 3.90% | 915.3 | 3.34% | 0.56% | 1.17 |
| STAT5(Stat)/mCD4+-Stat5-ChIP-Seq(GSE12346)/Homer | 1.00E-06 | -1.41E+01 | 0 | 1044 | 6.10% | 1443.6 | 5.27% | 0.83% | 1.16 |
| Cdx2(Homeobox)/mES-Cdx2-ChIP-Seq(GSE14586)/Homer | 1.00E-19 | -4.53E+01 | 0 | 3498 | 20.45% | 4859 | 17.72% | 2.73% | 1.15 |
| Mef2a(MADS)/HL1-Mef2a.biotin-ChIP-Seq(GSE21529/Homer | 1.00E-09 | -2.07E+01 | 0 | 1704 | 9.96% | 2370.9 | 8.65% | 1.31% | 1.15 |
| PRDM14(Zf)/H1-PRDM14-ChIP-Seq(GSE22767)/Homer | 1.00E-02 | -6.68E+00 | 0.0041 | 474 | 2.77% | 660.8 | 2.41% | 0.36% | 1.15 |
| Foxh1(Forkhead)/hESC-FOXH1-ChIP-Seq(GSE29422)/Homer | 1.00E-09 | -2.13E+01 | 0 | 1888 | 11.04% | 2642.7 | 9.64% | 1.40% | 1.15 |
| Mef2c(MADS)/GM12878-Mef2c-ChIP-Seq(GSE32465)/Homer | 1.00E-09 | -2.23E+01 | 0 | 2066 | 12.08% | 2901.1 | 10.58% | 1.50% | 1.14 |
| CTCF(Zf)/CD4+-CTCF-ChIP-Seq(Barski et al.)/Homer | 1.00E-05 | -1.32E+01 | 0 | 1191 | 6.96% | 1672.4 | 6.10% | 0.86% | 1.14 |
| HOXD13(Homeobox)/Chicken-Hoxd13-ChIP-Seq(GSE38910)/Homer | 1.00E-26 | -6.15E+01 | 0 | 5141 | 30.06% | 7231.2 | 26.37% | 3.69% | 1.14 |
| BORIS(Zf)/K562-CTCFL-ChIP-Seq(GSE32465)/Homer | 1.00E-04 | -1.07E+01 | 0.0001 | 1019 | 5.96% | 1439.9 | 5.25% | 0.71% | 1.14 |
| Lhx3(Homeobox)/Neuron-Lhx3-ChIP-Seq(GSE31456)/Homer | 1.00E-38 | -8.80E+01 | 0 | 7099 | 41.51% | 10056.6 | 36.68% | 4.83% | 1.13 |
| Smad2(MAD)/ES-SMAD2-ChIP-Seq(GSE29422)/Homer | 1.00E-08 | -1.89E+01 | 0 | 2039 | 11.92% | 2895.5 | 10.56% | 1.36% | 1.13 |
| Hoxc9(Homeobox)/Ainv15-Hoxc9-ChIP-Seq(GSE21812)/Homer | 1.00E-08 | -1.96E+01 | 0 | 2190 | 12.81% | 3117.8 | 11.37% | 1.44% | 1.13 |
| CHR/Cell-Cycle-Exp/Homer | 1.00E-11 | -2.70E+01 | 0 | 3001 | 17.55% | 4274.4 | 15.59% | 1.96% | 1.13 |
| STAT6/Macrophage-Stat6-ChIP-Seq(GSE38377)/Homer | 1.00E-06 | -1.52E+01 | 0 | 1744 | 10.20% | 2487.2 | 9.07% | 1.13% | 1.12 |
| Tcf3(HMG)/mES-Tcf3-ChIP-Seq(GSE11724)/Homer | 1.00E-03 | -7.10E+00 | 0.0028 | 745 | 4.36% | 1064.7 | 3.88% | 0.48% | 1.12 |
| Gata2(Zf)/K562-GATA2-ChIP-Seq(GSE18829)/Homer | 1.00E-06 | -1.58E+01 | 0 | 1901 | 11.12% | 2720.6 | 9.92% | 1.20% | 1.12 |
| Nkx2.5(Homeobox)/HL1-Nkx2.5.biotin-ChIP-Seq(GSE21529)/Homer | 1.00E-16 | -3.77E+01 | 0 | 4315 | 25.23% | 6178.6 | 22.54% | 2.69% | 1.12 |
| Tcf4(HMG)/Hct116-Tcf4-ChIP-Seq(SRA012054)/Homer | 1.00E-04 | -1.06E+01 | 0.0001 | 1291 | 7.55% | 1852.1 | 6.76% | 0.79% | 1.12 |
| Sox6(HMG)/Myotubes-Sox6-ChIP-Seq(GSE32627)/Homer | 1.00E-17 | -4.01E+01 | 0 | 4803 | 28.09% | 6907.5 | 25.19% | 2.90% | 1.12 |
| Fli1(ETS)/CD8-FLI-ChIP-Seq(GSE20898)/Homer | 1.00E-07 | -1.84E+01 | 0 | 2404 | 14.06% | 3457.3 | 12.61% | 1.45% | 1.11 |
| Gata4(Zf)/Heart-Gata4-ChIP-Seq(GSE35151)/Homer | 1.00E-10 | -2.41E+01 | 0 | 3221 | 18.84% | 4643.8 | 16.94% | 1.90% | 1.11 |
| Nkx6.1(Homeobox)/Islet-Nkx6.1-ChIP-Seq(GSE40975)/Homer | 1.00E-54 | -1.25E+02 | 0 | 10074 | 58.91% | 14525.1 | 52.98% | 5.93% | 1.11 |
| Pdx1(Homeobox)/Islet-Pdx1-ChIP-Seq(SRA008281)/Homer | 1.00E-14 | -3.24E+01 | 0 | 4218 | 24.67% | 6086 | 22.20% | 2.47% | 1.11 |
| GATA3(Zf)/iTreg-Gata3-ChIP-Seq(GSE20898)/Homer | 1.00E-17 | -4.05E+01 | 0 | 5150 | 30.12% | 7441.7 | 27.14% | 2.98% | 1.11 |
| STAT6(Stat)/CD4-Stat6-ChIP-Seq(GSE22104)/Homer | 1.00E-05 | -1.33E+01 | 0 | 1853 | 10.84% | 2677.7 | 9.77% | 1.07% | 1.11 |
| Oct4(POU/Homeobox)/mES-Oct4-ChIP-Seq(GSE11431)/Homer | 1.00E-05 | -1.31E+01 | 0 | 1865 | 10.91% | 2698.2 | 9.84% | 1.07% | 1.11 |
| EWS:FLI1-fusion(ETS)/SK_N_MC-EWS:FLI1-ChIP-Seq(SRA014231)/Homer | 1.00E-04 | -1.03E+01 | 0.0001 | 1415 | 8.27% | 2045.2 | 7.46% | 0.81% | 1.11 |
| Gata1(Zf)/K562-GATA1-ChIP-Seq(GSE18829)/Homer | 1.00E-04 | -1.11E+01 | 0.0001 | 1613 | 9.43% | 2338.2 | 8.53% | 0.90% | 1.11 |
| Sox3(HMG)/NPC-Sox3-ChIP-Seq(GSE33059)/Homer | 1.00E-14 | -3.30E+01 | 0 | 4703 | 27.50% | 6829.4 | 24.91% | 2.59% | 1.10 |
| bZIP:IRF/Th17-BatF-ChIP-Seq(GSE39756)/Homer | 1.00E-05 | -1.17E+01 | 0 | 1854 | 10.84% | 2700 | 9.85% | 0.99% | 1.10 |
| ERG(ETS)/VCaP-ERG-ChIP-Seq(GSE14097)/Homer | 1.00E-10 | -2.35E+01 | 0 | 3749 | 21.92% | 5465.4 | 19.93% | 1.99% | 1.10 |
| CRX(Homeobox)/Retina-Crx-ChIP-Seq(GSE20012)/Homer | 1.00E-20 | -4.66E+01 | 0 | 6520 | 38.13% | 9515.5 | 34.71% | 3.42% | 1.10 |
| NFY(CCAAT)/Promoter/Homer | 1.00E-03 | -8.35E+00 | 0.0009 | 1371 | 8.02% | 2004.9 | 7.31% | 0.71% | 1.10 |
| BMYB(HTH)/Hela-BMYB-ChIPSeq(GSE27030)/Homer | 1.00E-09 | -2.08E+01 | 0 | 3553 | 20.78% | 5196.8 | 18.95% | 1.83% | 1.10 |
| ETV1(ETS)/GIST48-ETV1-ChIP-Seq(GSE22441)/Homer | 1.00E-07 | -1.67E+01 | 0 | 2955 | 17.28% | 4326.9 | 15.78% | 1.50% | 1.10 |
| MafF(bZIP)/HepG2-MafF-ChIP-Seq(GSE31477)/Homer | 1.00E-02 | -6.83E+00 | 0.0036 | 1138 | 6.65% | 1668.2 | 6.08% | 0.57% | 1.09 |
| Nanog(Homeobox)/mES-Nanog-ChIP-Seq(GSE11724)/Homer | 1.00E-41 | -9.49E+01 | 0 | 10387 | 60.74% | 15251.5 | 55.63% | 5.11% | 1.09 |
| ETS1(ETS)/Jurkat-ETS1-ChIP-Seq(GSE17954)/Homer | 1.00E-05 | -1.33E+01 | 0 | 2511 | 14.68% | 3688.7 | 13.45% | 1.23% | 1.09 |
| EWS:ERG-fusion(ETS)/CADO_ES1-EWS:ERG-ChIP-Seq(SRA014231)/Homer | 1.00E-05 | -1.32E+01 | 0 | 2523 | 14.75% | 3709.3 | 13.53% | 1.22% | 1.09 |
| Tbx5(T-box)/HL1-Tbx5.biotin-ChIP-Seq(GSE21529)/Homer | 1.00E-13 | -3.11E+01 | 0 | 5636 | 32.96% | 8306.6 | 30.30% | 2.66% | 1.09 |
| Tbet(T-box)/CD8-Tbet-ChIP-Seq(GSE33802)/Homer | 1.00E-05 | -1.33E+01 | 0 | 2756 | 16.12% | 4068.9 | 14.84% | 1.28% | 1.09 |
| Isl1(Homeobox)/Neuron-Isl1-ChIP-Seq(GSE31456)/Homer | 1.00E-14 | -3.27E+01 | 0 | 6039 | 35.31% | 8919.4 | 32.53% | 2.78% | 1.09 |
| EBF1(EBF)/Near-E2A-ChIP-Seq(GSE21512)/Homer | 1.00E-02 | -5.03E+00 | 0.0202 | 907 | 5.30% | 1340.6 | 4.89% | 0.41% | 1.08 |
| Nkx2.1(Homeobox)/LungAC-Nkx2.1-ChIP-Seq(GSE43252)/Homer | 1.00E-11 | -2.57E+01 | 0 | 5493 | 32.12% | 8154.7 | 29.74% | 2.38% | 1.08 |
| STAT4(Stat)/CD4-Stat4-ChIP-Seq(GSE22104)/Homer | 1.00E-04 | -1.15E+01 | 0 | 2829 | 16.54% | 4210.6 | 15.36% | 1.18% | 1.08 |
| Bcl6(Zf)/Liver-Bcl6-ChIP-Seq(GSE31578)/Homer | 1.00E-05 | -1.35E+01 | 0 | 3359 | 19.64% | 5001.1 | 18.24% | 1.40% | 1.08 |
| Sox2(HMG)/mES-Sox2-ChIP-Seq(GSE11431)/Homer | 1.00E-04 | -9.60E+00 | 0.0003 | 2364 | 13.82% | 3519.6 | 12.84% | 0.98% | 1.08 |
| ZFX(Zf)/mES-Zfx-ChIP-Seq(GSE11431)/Homer | 1.00E-02 | -5.14E+00 | 0.0182 | 1205 | 7.05% | 1799.9 | 6.56% | 0.49% | 1.07 |
| GABPA(ETS)/Jurkat-GABPa-ChIP-Seq(GSE17954)/Homer | 1.00E-03 | -7.57E+00 | 0.0018 | 1922 | 11.24% | 2868.1 | 10.46% | 0.78% | 1.07 |
| AMYB(HTH)/Testes-AMYB-ChIP-Seq(GSE44588)/Homer | 1.00E-05 | -1.16E+01 | 0 | 3262 | 19.07% | 4883.6 | 17.81% | 1.26% | 1.07 |
| Rbpj1(?)/Panc1-Rbpj1-ChIP-Seq(GSE47459)/Homer | 1.00E-03 | -8.81E+00 | 0.0005 | 2724 | 15.93% | 4094.1 | 14.93% | 1.00% | 1.07 |
| BMAL1(HLH)/Liver-Bmal1-ChIP-Seq(GSE39860)/Homer | 1.00E-04 | -9.59E+00 | 0.0003 | 3039 | 17.77% | 4571 | 16.67% | 1.10% | 1.07 |
| Smad3(MAD)/NPC-Smad3-ChIP-Seq(GSE36673)/Homer | 1.00E-06 | -1.54E+01 | 0 | 4804 | 28.09% | 7230.5 | 26.37% | 1.72% | 1.07 |
| HOXA9(Homeobox)/HSC-Hoxa9-ChIP-Seq(GSE33509)/Homer | 1.00E-03 | -8.15E+00 | 0.001 | 2646 | 15.47% | 3985.2 | 14.54% | 0.93% | 1.06 |
| NFAT(RHD)/Jurkat-NFATC1-ChIP-Seq(Jolma et al.)/Homer | 1.00E-03 | -8.20E+00 | 0.001 | 2756 | 16.12% | 4156.1 | 15.16% | 0.96% | 1.06 |
| Pitx1(Homeobox)/Chicken-Pitx1-ChIP-Seq(GSE38910)/Homer | 1.00E-24 | -5.69E+01 | 0 | 11054 | 64.64% | 16668.2 | 60.80% | 3.84% | 1.06 |
| Smad4(MAD)/ESC-SMAD4-ChIP-Seq(GSE29422)/Homer | 1.00E-02 | -5.88E+00 | 0.0088 | 1912 | 11.18% | 2885.5 | 10.52% | 0.66% | 1.06 |
| MYB(HTH)/ERMYB-Myb-ChIPSeq(GSE22095)/Homer | 1.00E-04 | -1.14E+01 | 0.0001 | 3928 | 22.97% | 5929.1 | 21.63% | 1.34% | 1.06 |
| Unknown(Homeobox)/Limb-p300-ChIP-Seq/Homer | 1.00E-02 | -6.86E+00 | 0.0036 | 2639 | 15.43% | 4001.2 | 14.59% | 0.84% | 1.06 |
| PR(NR)/T47D-PR-ChIP-Seq(GSE31130)/Homer | 1.00E-04 | -1.10E+01 | 0.0001 | 4608 | 26.95% | 7005.8 | 25.55% | 1.40% | 1.05 |
| Nkx3.1(Homeobox)/LNCaP-Nkx3.1-ChIP-Seq(GSE28264)/Homer | 1.00E-04 | -1.10E+01 | 0.0001 | 5278 | 30.86% | 8064.6 | 29.41% | 1.45% | 1.05 |
| Jun-AP1(bZIP)/K562-cJun-ChIP-Seq(GSE31477)/Homer | 1.00E-02 | -4.72E+00 | 0.0264 | 2264 | 13.24% | 3463.7 | 12.63% | 0.61% | 1.05 |
| Eomes(T-box)/H9-Eomes-ChIP-Seq(GSE26097)/Homer | 1.00E-04 | -1.01E+01 | 0.0002 | 5633 | 32.94% | 8646 | 31.53% | 1.41% | 1.04 |
| AR-halfsite(NR)/LNCaP-AR-ChIP-Seq(GSE27824)/Homer | 1.00E-02 | -6.31E+00 | 0.0059 | 5868 | 34.31% | 9119.1 | 33.26% | 1.05% | 1.03 |
| Foxo1(Forkhead)/RAW-Foxo1-ChIP-Seq(Fan et al.)/Homer | 1.00E-02 | -4.79E+00 | 0.0249 | 4833 | 28.26% | 7523.3 | 27.44% | 0.82% | 1.03 |

Supplementary Table 6. Motifs enriched in all peaks unique to differentiated cells at 24 h.

| All Peaks Unique to 24 Hour Treated | P-value | Log P-value | q-value (Benjamini) | # of Target Sequences with Motif (of 15089) | % of Target Sequences with Motif | # of Background Sequences with Motif(of 29538) | % of Background Sequences with Motif | % Target >Background | Fold Target >Background |
| --- | --- | --- | --- | --- | --- | --- | --- | --- | --- |
| **REST-NRSF(Zf)/Jurkat-NRSF-ChIP-Seq/Homer** | **1.00E-02** | **-5.63E+00** | **0.0339** | **15** | **0.10%** | **13.7** | **0.05%** | **0.05%** | **2.00** |
| **IRF2(IRF)/Erythroblas-IRF2-ChIP-Seq(GSE36985)/Homer** | **1.00E-18** | **-4.16E+01** | **0** | **415** | **2.75%** | **513** | **1.74%** | **1.01%** | **1.58** |
| **ISRE(IRF)/ThioMac-LPS-exp(GSE23622)/HOMER** | **1.00E-10** | **-2.50E+01** | **0** | **265** | **1.76%** | **335.9** | **1.14%** | **0.62%** | **1.54** |
| **E2F(E2F)/Cell-Cycle-Exp/Homer** | **1.00E-02** | **-5.11E+00** | **0.051** | **49** | **0.32%** | **65.6** | **0.22%** | **0.10%** | **1.45** |
| **CTCF-SatelliteElement/CD4+-CTCF-ChIP-Seq(Barski et al.)/Homer** | **1.00E-02** | **-4.71E+00** | **0.0648** | **56** | **0.37%** | **78.5** | **0.27%** | **0.10%** | **1.37** |
| **CTCF(Zf)/CD4+-CTCF-ChIP-Seq(Barski et al.)/Homer** | **1.00E-29** | **-6.73E+01** | **0** | **1610** | **10.67%** | **2377.8** | **8.05%** | **2.62%** | **1.33** |
| **GRHL2(CP2)/HBE-GRHL2-ChIP-Seq(GSE46194)/Homer** | **1.00E-24** | **-5.71E+01** | **0** | **1410** | **9.34%** | **2090.4** | **7.08%** | **2.26%** | **1.32** |
| **BORIS(Zf)/K562-CTCFL-ChIP-Seq(GSE32465)/Homer** | **1.00E-25** | **-5.89E+01** | **0** | **1533** | **10.16%** | **2292.8** | **7.76%** | **2.40%** | **1.31** |
| **IRF1(IRF)/PBMC-IRF1-ChIP-Seq(GSE43036)/Homer** | **1.00E-07** | **-1.82E+01** | **0** | **517** | **3.43%** | **786.7** | **2.66%** | **0.77%** | **1.29** |
| GATA-DR8(Zf)/iTreg-Gata3-ChIP-Seq(GSE20898)/Homer | 1.00E-02 | -5.85E+00 | 0.0297 | 199 | 1.32% | 318.5 | 1.08% | 0.24% | 1.22 |
| GATA-IR3(Zf)/iTreg-Gata3-ChIP-Seq(GSE20898)/Homer | 1.00E-04 | -9.70E+00 | 0.0011 | 418 | 2.77% | 675.2 | 2.29% | 0.48% | 1.21 |
| PRDM1/BMI1(Zf)/Hela-PRDM1-ChIP-Seq(GSE31477)/Homer | 1.00E-06 | -1.57E+01 | 0 | 1469 | 9.73% | 2523.7 | 8.54% | 1.19% | 1.14 |
| HNF4a(NR/DR1)/HepG2-HNF4a-ChIP-Seq(GSE25021)/Homer | 1.00E-03 | -8.35E+00 | 0.0032 | 774 | 5.13% | 1336.9 | 4.53% | 0.60% | 1.13 |
| HRE(HSF)/Striatum-HSF1-ChIP-Seq(GSE38000)/Homer | 1.00E-02 | -5.63E+00 | 0.0339 | 479 | 3.17% | 828.8 | 2.81% | 0.36% | 1.13 |
| NeuroD1(bHLH)/Islet-NeuroD1-ChIP-Seq(GSE30298)/Homer | 1.00E-06 | -1.39E+01 | 0 | 1631 | 10.81% | 2847.8 | 9.64% | 1.17% | 1.12 |
| RBPJ:Ebox/Panc1-Rbpj1-ChIP-Seq(GSE47459)/Homer | 1.00E-02 | -5.20E+00 | 0.0485 | 573 | 3.80% | 1008.7 | 3.42% | 0.38% | 1.11 |
| FOXP1(Forkhead)/H9-FOXP1-ChIP-Seq(GSE31006)/Homer | 1.00E-03 | -8.77E+00 | 0.0022 | 1289 | 8.54% | 2287.1 | 7.74% | 0.80% | 1.10 |
| FOXA1(Forkhead)/MCF7-FOXA1-ChIP-Seq(GSE26831)/Homer | 1.00E-08 | -1.89E+01 | 0 | 3093 | 20.50% | 5514 | 18.67% | 1.83% | 1.10 |
| IRF4(IRF)/GM12878-IRF4-ChIP-Seq(GSE32465)/Homer | 1.00E-03 | -7.26E+00 | 0.0078 | 1143 | 7.57% | 2039.5 | 6.90% | 0.67% | 1.10 |
| PPARE(NR/DR1)/3T3L1-Pparg-ChIP-Seq(GSE13511)/Homer | 1.00E-04 | -9.80E+00 | 0.0011 | 1688 | 11.19% | 3018.3 | 10.22% | 0.97% | 1.09 |
| CEBP(bZIP)/CEBPb-ChIP-Seq(GSE21512)/Homer | 1.00E-05 | -1.30E+01 | 0.0001 | 2389 | 15.83% | 4283.8 | 14.50% | 1.33% | 1.09 |
| PRDM9(Zf)/Testis-DMC1-ChIP-Seq(GSE35498)/Homer | 1.00E-02 | -4.83E+00 | 0.0593 | 740 | 4.90% | 1326.5 | 4.49% | 0.41% | 1.09 |
| FOXA1(Forkhead)/LNCAP-FOXA1-ChIP-Seq(GSE27824)/Homer | 1.00E-08 | -1.99E+01 | 0 | 3645 | 24.16% | 6542.8 | 22.15% | 2.01% | 1.09 |
| E2A-nearPU.1(HLH)/Bcell-PU.1-ChIP-Seq(GSE21512)/Homer | 1.00E-04 | -9.66E+00 | 0.0011 | 2425 | 16.07% | 4414.2 | 14.94% | 1.13% | 1.08 |
| Fox:Ebox(Forkhead:HLH)/Panc1-Foxa2-ChIP-Seq(GSE47459)/Homer | 1.00E-04 | -1.07E+01 | 0.0005 | 2706 | 17.93% | 4926.8 | 16.68% | 1.25% | 1.07 |
| RXR(NR/DR1)/3T3L1-RXR-ChIP-Seq(GSE13511)/Homer | 1.00E-03 | -7.77E+00 | 0.0049 | 1933 | 12.81% | 3521 | 11.92% | 0.89% | 1.07 |
| NFAT(RHD)/Jurkat-NFATC1-ChIP-Seq(Jolma et al.)/Homer | 1.00E-03 | -8.37E+00 | 0.0032 | 2292 | 15.19% | 4189.1 | 14.18% | 1.01% | 1.07 |
| Gfi1b(Zf)/HPC7-Gfi1b-ChIP-Seq(GSE22178)/Homer | 1.00E-02 | -5.08E+00 | 0.051 | 1445 | 9.58% | 2654 | 8.99% | 0.59% | 1.07 |
| Foxa2(Forkhead)/Liver-Foxa2-ChIP-Seq(GSE25694)/Homer | 1.00E-03 | -7.19E+00 | 0.0081 | 2361 | 15.65% | 4348.7 | 14.72% | 0.93% | 1.06 |
| Erra(NR)/HepG2-Erra-ChIP-Seq(GSE31477)/Homer | 1.00E-04 | -9.42E+00 | 0.0012 | 3751 | 24.86% | 6954.1 | 23.54% | 1.32% | 1.06 |
| Olig2(bHLH)/Neuron-Olig2-ChIP-Seq(GSE30882)/Homer | 1.00E-03 | -7.92E+00 | 0.0045 | 3869 | 25.64% | 7221 | 24.45% | 1.19% | 1.05 |
| BMYB(HTH)/Hela-BMYB-ChIPSeq(GSE27030)/Homer | 1.00E-02 | -4.87E+00 | 0.059 | 3223 | 21.36% | 6072 | 20.56% | 0.80% | 1.04 |
| Eomes(T-box)/H9-Eomes-ChIP-Seq(GSE26097)/Homer | 1.00E-02 | -5.71E+00 | 0.0327 | 4638 | 30.74% | 8778.8 | 29.72% | 1.02% | 1.03 |
| NF1-halfsite(CTF)/LNCaP-NF1-ChIP-Seq(Unpublished)/Homer | 1.00E-02 | -4.72E+00 | 0.0648 | 3859 | 25.57% | 7306.9 | 24.74% | 0.83% | 1.03 |
| Ptf1a(HLH)/Panc1-Ptf1a-ChIP-Seq(GSE47459)/Homer | 1.00E-02 | -5.00E+00 | 0.0537 | 4618 | 30.60% | 8766.4 | 29.68% | 0.92% | 1.03 |

Supplementary Table 7. Motifs enriched in all FAIRE peaks unique to control cells at 144 h.

| All Peaks Unique to 144 Hour Control | P-value | Log P-value | q-value (Benjamini) | # of Target Sequences with Motif (of 21611) | % of Target Sequences with Motif | # of Background Sequences with Motif (of 28046) | % of Background Sequences with Motif | % Target >Background | Fold Target >Background |
| --- | --- | --- | --- | --- | --- | --- | --- | --- | --- |
| **p53(p53)/Saos-p53-ChIP-Seq(GSE15780)/Homer** | **1.00E-61** | **-1.42E+02** | **0** | **1176** | **5.44%** | **911.9** | **3.25%** | **2.19%** | **1.67** |
| **p53(p53)/Saos-p53-ChIP-Seq/Homer** | **1.00E-61** | **-1.42E+02** | **0** | **1176** | **5.44%** | **911.9** | **3.25%** | **2.19%** | **1.67** |
| **p53(p53)/mES-cMyc-ChIP-Seq(GSE11431)/Homer** | **1.00E-05** | **-1.16E+01** | **0.0002** | **132** | **0.61%** | **115.6** | **0.41%** | **0.20%** | **1.49** |
| **p63(p53)/Keratinocyte-p63-ChIP-Seq(GSE17611)/Homer** | **1.00E-76** | **-1.76E+02** | **0** | **2401** | **11.11%** | **2120.3** | **7.56%** | **3.55%** | **1.47** |
| NFkB-p65-Rel(RHD)/LPS-exp(GSE23622)/Homer | 1.00E-02 | -6.29E+00 | 0.0143 | 213 | 0.99% | 225.1 | 0.80% | 0.19% | 1.24 |
| EBF(EBF)/proBcell-EBF-ChIP-Seq(GSE21978)/Homer | 1.00E-02 | -6.84E+00 | 0.0094 | 324 | 1.50% | 353.5 | 1.26% | 0.24% | 1.19 |
| Egr2/Thymocytes-Egr2-ChIP-Seq(GSE34254)/Homer | 1.00E-02 | -5.24E+00 | 0.0354 | 251 | 1.16% | 276.5 | 0.99% | 0.17% | 1.17 |
| Oct4:Sox17/F9-Sox17-ChIP-Seq(GSE44553)/Homer | 1.00E-03 | -8.81E+00 | 0.002 | 573 | 2.65% | 638.9 | 2.28% | 0.37% | 1.16 |
| bHLHE40(HLH)/HepG2-BHLHE40-ChIP-Seq(GSE31477)/Homer | 1.00E-02 | -6.72E+00 | 0.0102 | 421 | 1.95% | 470.5 | 1.68% | 0.27% | 1.16 |
| HRE(HSF)/HepG2-HSF1-ChIP-Seq(GSE31477)/Homer | 1.00E-02 | -4.99E+00 | 0.0421 | 321 | 1.49% | 362.1 | 1.29% | 0.20% | 1.16 |
| Tcfcp2l1(CP2)/mES-Tcfcp2l1-ChIP-Seq(GSE11431)/Homer | 1.00E-02 | -6.55E+00 | 0.0117 | 482 | 2.23% | 545 | 1.94% | 0.29% | 1.15 |
| Atf4(bZIP)/MEF-Atf4-ChIP-Seq(GSE35681)/Homer | 1.00E-07 | -1.83E+01 | 0 | 1662 | 7.69% | 1885.2 | 6.72% | 0.97% | 1.14 |
| STAT6(Stat)/CD4-Stat6-ChIP-Seq(GSE22104)/Homer | 1.00E-10 | -2.38E+01 | 0 | 2256 | 10.44% | 2565.5 | 9.15% | 1.29% | 1.14 |
| ZNF143\|STAF(Zf)/CUTLL-ZNF143-ChIP-Seq(GSE29600)/Homer | 1.00E-03 | -7.10E+00 | 0.0075 | 600 | 2.78% | 684.9 | 2.44% | 0.34% | 1.14 |
| CEBP(bZIP)/CEBPb-ChIP-Seq(GSE21512)/Homer | 1.00E-14 | -3.40E+01 | 0 | 3927 | 18.18% | 4536.2 | 16.17% | 2.01% | 1.12 |
| Chop(bZIP)/MEF-Chop-ChIP-Seq(GSE35681)/Homer | 1.00E-04 | -1.12E+01 | 0.0003 | 1262 | 5.84% | 1457.5 | 5.20% | 0.64% | 1.12 |
| c-Myc(HLH)/LNCAP-cMyc-ChIP-Seq(unpublished)/Homer | 1.00E-02 | -5.57E+00 | 0.0267 | 592 | 2.74% | 688.4 | 2.45% | 0.29% | 1.12 |
| CEBP:AP1(bZIP)/ThioMac-CEBPb-ChIP-Seq(GSE21512)/Homer | 1.00E-12 | -2.94E+01 | 0 | 3703 | 17.14% | 4300.5 | 15.33% | 1.81% | 1.12 |
| AP-2gamma(AP2)/MCF7-TFAP2C-ChIP-Seq(GSE21234)/Homer | 1.00E-06 | -1.58E+01 | 0 | 2047 | 9.48% | 2380.7 | 8.49% | 0.99% | 1.12 |
| STAT6/Macrophage-Stat6-ChIP-Seq(GSE38377)/Homer | 1.00E-06 | -1.39E+01 | 0 | 2133 | 9.87% | 2506.7 | 8.94% | 0.93% | 1.10 |
| USF1(HLH)/GM12878-Usf1-ChIP-Seq(GSE32465)/Homer | 1.00E-02 | -5.99E+00 | 0.0181 | 849 | 3.93% | 1001.1 | 3.57% | 0.36% | 1.10 |
| AP-2alpha(AP2)/Hela-AP2alpha-ChIP-Seq(GSE31477)/Homer | 1.00E-03 | -8.74E+00 | 0.002 | 1551 | 7.18% | 1841.7 | 6.57% | 0.61% | 1.09 |
| EBF1(EBF)/Near-E2A-ChIP-Seq(GSE21512)/Homer | 1.00E-03 | -9.16E+00 | 0.0015 | 1835 | 8.49% | 2190.7 | 7.81% | 0.68% | 1.09 |
| Egr1(Zf)/K562-Egr1-ChIP-Seq(GSE32465)/Homer | 1.00E-02 | -5.03E+00 | 0.0415 | 940 | 4.35% | 1126.7 | 4.02% | 0.33% | 1.08 |
| Cdx2(Homeobox)/mES-Cdx2-ChIP-Seq(GSE14586)/Homer | 1.00E-05 | -1.36E+01 | 0 | 3399 | 15.73% | 4093.2 | 14.59% | 1.14% | 1.08 |
| NPAS2(HLH)/Liver-NPAS2-ChIP-Seq(GSE39860)/Homer | 1.00E-04 | -9.34E+00 | 0.0013 | 2390 | 11.06% | 2883.5 | 10.28% | 0.78% | 1.08 |
| Bcl6(Zf)/Liver-Bcl6-ChIP-Seq(GSE31578)/Homer | 1.00E-06 | -1.49E+01 | 0 | 4603 | 21.31% | 5594.1 | 19.94% | 1.37% | 1.07 |
| Phox2a(Homeobox)/Neuron-Phox2a-ChIP-Seq(GSE31456)/Homer | 1.00E-02 | -6.39E+00 | 0.0133 | 1957 | 9.06% | 2383.1 | 8.50% | 0.56% | 1.07 |
| TEAD(TEA)/Fibroblast-PU.1-ChIP-Seq(Unpublished)/Homer | 1.00E-03 | -7.80E+00 | 0.0048 | 3080 | 14.26% | 3779.8 | 13.47% | 0.79% | 1.06 |
| TEAD4(TEA)/Tropoblast-Tead4-ChIP-Seq(GSE37350)/Homer | 1.00E-03 | -7.76E+00 | 0.0048 | 3208 | 14.85% | 3942.9 | 14.06% | 0.79% | 1.06 |
| BMAL1(HLH)/Liver-Bmal1-ChIP-Seq(GSE39860)/Homer | 1.00E-04 | -9.72E+00 | 0.001 | 4455 | 20.62% | 5491.9 | 19.58% | 1.04% | 1.05 |
| Lhx2(Homeobox)/HFSC-Lhx2-ChIP-Seq(GSE48068)/Homer | 1.00E-04 | -9.75E+00 | 0.001 | 4687 | 21.70% | 5786.6 | 20.63% | 1.07% | 1.05 |
| Rbpj1(?)/Panc1-Rbpj1-ChIP-Seq(GSE47459)/Homer | 1.00E-03 | -7.45E+00 | 0.0062 | 3991 | 18.47% | 4944.6 | 17.63% | 0.84% | 1.05 |
| ZFX(Zf)/mES-Zfx-ChIP-Seq(GSE11431)/Homer | 1.00E-02 | -4.61E+00 | 0.0568 | 2378 | 11.01% | 2950.5 | 10.52% | 0.49% | 1.05 |
| HOXD13(Homeobox)/Chicken-Hoxd13-ChIP-Seq(GSE38910)/Homer | 1.00E-03 | -7.41E+00 | 0.0062 | 4954 | 22.93% | 6175.7 | 22.01% | 0.92% | 1.04 |
| Smad4(MAD)/ESC-SMAD4-ChIP-Seq(GSE29422)/Homer | 1.00E-02 | -5.17E+00 | 0.0368 | 3287 | 15.21% | 4096.3 | 14.60% | 0.61% | 1.04 |
| Smad2(MAD)/ES-SMAD2-ChIP-Seq(GSE29422)/Homer | 1.00E-02 | -4.77E+00 | 0.0497 | 3335 | 15.44% | 4167.3 | 14.86% | 0.58% | 1.04 |
| Nkx3.1(Homeobox)/LNCaP-Nkx3.1-ChIP-Seq(GSE28264)/Homer | 1.00E-03 | -7.18E+00 | 0.0072 | 6714 | 31.08% | 8439.1 | 30.08% | 1.00% | 1.03 |
| Smad3(MAD)/NPC-Smad3-ChIP-Seq(GSE36673)/Homer | 1.00E-03 | -7.22E+00 | 0.0072 | 7177 | 33.22% | 9034.4 | 32.21% | 1.01% | 1.03 |
| Sox6(HMG)/Myotubes-Sox6-ChIP-Seq(GSE32627)/Homer | 1.00E-02 | -5.33E+00 | 0.0332 | 5572 | 25.79% | 7020.6 | 25.03% | 0.76% | 1.03 |
| Isl1(Homeobox)/Neuron-Isl1-ChIP-Seq(GSE31456)/Homer | 1.00E-02 | -4.97E+00 | 0.0421 | 6945 | 32.15% | 8799.9 | 31.37% | 0.78% | 1.02 |
| Pitx1(Homeobox)/Chicken-Pitx1-ChIP-Seq(GSE38910)/Homer | 1.00E-03 | -8.81E+00 | 0.002 | 13346 | 61.78% | 16992.9 | 60.57% | 1.21% | 1.02 |
| Nkx6.1(Homeobox)/Islet-Nkx6.1-ChIP-Seq(GSE40975)/Homer | 1.00E-02 | -6.17E+00 | 0.0157 | 10818 | 50.07% | 13773 | 49.10% | 0.97% | 1.02 |

Supplementary Table 8. Motifs enriched in all FAIRE peaks unique to differentiated cells at 144 h.

| All Peaks Unique to 144 Hour Treated | P-value | log P-pvalue | q-value (Benjamini) | # Target Sequences with Motif | % of Targets Sequences with Motif | # Background Sequences with Motif | % of Background Sequences with Motif | % Target >Background | Fold Target >Background |
| --- | --- | --- | --- | --- | --- | --- | --- | --- | --- |
| **REST-NRSF(Zf)/Jurkat-NRSF-ChIP-Seq/Homer** | **1.00E-02** | **-6.90E+00** | **0.0103** | **15** | **0.13%** | **20.3** | **0.05%** | **0.08%** | **2.60** |
| **CTCF-SatelliteElement/CD4+-CTCF-ChIP-Seq(Barski et al.)/Homer** | **1.00E-08** | **-1.90E+01** | **0** | **56** | **0.50%** | **80.5** | **0.21%** | **0.29%** | **2.38** |
| **RARg(NR)/ES-RARg-ChIP-Seq(GSE30538)/Homer** | **1.00E-05** | **-1.30E+01** | **0** | **40** | **0.36%** | **60.8** | **0.16%** | **0.20%** | **2.25** |
| **BORIS(Zf)/K562-CTCFL-ChIP-Seq(GSE32465)/Homer** | **1.00E-41** | **-9.51E+01** | **0** | **1403** | **12.62%** | **3363.9** | **8.77%** | **3.85%** | **1.44** |
| **CTCF(Zf)/CD4+-CTCF-ChIP-Seq(Barski et al.)/Homer** | **1.00E-39** | **-9.08E+01** | **0** | **1512** | **13.60%** | **3715.4** | **9.69%** | **3.91%** | **1.40** |
| **TR4(NR/DR1)/Hela-TR4-ChIP-Seq(GSE24685)/Homer** | **1.00E-03** | **-7.17E+00** | **0.0082** | **117** | **1.05%** | **296.3** | **0.77%** | **0.28%** | **1.36** |
| **ISRE(IRF)/ThioMac-LPS-exp(GSE23622)/HOMER** | **1.00E-04** | **-9.22E+00** | **0.0013** | **184** | **1.65%** | **476.5** | **1.24%** | **0.41%** | **1.33** |
| **GATA-IR4(Zf)/iTreg-Gata3-ChIP-Seq(GSE20898)/Homer** | **1.00E-04** | **-1.09E+01** | **0.0003** | **236** | **2.12%** | **615.4** | **1.60%** | **0.52%** | **1.33** |
| **IRF2(IRF)/Erythroblas-IRF2-ChIP-Seq(GSE36985)/Homer** | **1.00E-04** | **-9.35E+00** | **0.0012** | **268** | **2.41%** | **729.6** | **1.90%** | **0.51%** | **1.27** |
| **GATA-DR4(Zf)/iTreg-Gata3-ChIP-Seq(GSE20898)/Homer** | **1.00E-02** | **-6.86E+00** | **0.0104** | **207** | **1.86%** | **572.1** | **1.49%** | **0.37%** | **1.25** |
| Gata1(Zf)/K562-GATA1-ChIP-Seq(GSE18829)/Homer | 1.00E-10 | -2.53E+01 | 0 | 1188 | 10.68% | 3384.2 | 8.82% | 1.86% | 1.21 |
| IRF1(IRF)/PBMC-IRF1-ChIP-Seq(GSE43036)/Homer | 1.00E-03 | -8.45E+00 | 0.0026 | 381 | 3.43% | 1093.1 | 2.85% | 0.58% | 1.20 |
| GATA-DR8(Zf)/iTreg-Gata3-ChIP-Seq(GSE20898)/Homer | 1.00E-02 | -4.72E+00 | 0.0686 | 165 | 1.48% | 470.9 | 1.23% | 0.25% | 1.20 |
| Nur77(NR)/K562-NR4A1-ChIP-Seq(GSE31363)/Homer | 1.00E-03 | -7.95E+00 | 0.0041 | 362 | 3.26% | 1041.6 | 2.72% | 0.54% | 1.20 |
| Gata2(Zf)/K562-GATA2-ChIP-Seq(GSE18829)/Homer | 1.00E-10 | -2.48E+01 | 0 | 1347 | 12.11% | 3897.8 | 10.16% | 1.95% | 1.19 |
| HNF4a(NR/DR1)/HepG2-HNF4a-ChIP-Seq(GSE25021)/Homer | 1.00E-04 | -1.02E+01 | 0.0006 | 613 | 5.51% | 1801.5 | 4.70% | 0.81% | 1.17 |
| Gata4(Zf)/Heart-Gata4-ChIP-Seq(GSE35151)/Homer | 1.00E-11 | -2.64E+01 | 0 | 2095 | 18.84% | 6282.4 | 16.38% | 2.46% | 1.15 |
| Foxa2(Forkhead)/Liver-Foxa2-ChIP-Seq(GSE25694)/Homer | 1.00E-10 | -2.47E+01 | 0 | 2024 | 18.20% | 6085 | 15.87% | 2.33% | 1.15 |
| FOXA1(Forkhead)/MCF7-FOXA1-ChIP-Seq(GSE26831)/Homer | 1.00E-11 | -2.61E+01 | 0 | 2632 | 23.67% | 8051 | 20.99% | 2.68% | 1.13 |
| ELF1(ETS)/Jurkat-ELF1-ChIP-Seq(SRA014231)/Homer | 1.00E-03 | -7.48E+00 | 0.0063 | 814 | 7.32% | 2508.8 | 6.54% | 0.78% | 1.12 |
| E2A-nearPU.1(HLH)/Bcell-PU.1-ChIP-Seq(GSE21512)/Homer | 1.00E-05 | -1.31E+01 | 0 | 1759 | 15.82% | 5470.7 | 14.27% | 1.55% | 1.11 |
| FOXA1(Forkhead)/LNCAP-FOXA1-ChIP-Seq(GSE27824)/Homer | 1.00E-09 | -2.25E+01 | 0 | 3046 | 27.39% | 9507.9 | 24.79% | 2.60% | 1.10 |
| HNF6(Homeobox)/Liver-Hnf6-ChIP-Seq(ERP000394)/Homer | 1.00E-02 | -6.58E+00 | 0.0126 | 898 | 8.08% | 2808.7 | 7.32% | 0.76% | 1.10 |
| ELF5(ETS)/T47D-ELF5-ChIP-Seq(GSE30407)/Homer | 1.00E-05 | -1.16E+01 | 0.0001 | 1776 | 15.97% | 5567.4 | 14.52% | 1.45% | 1.10 |
| Fox:Ebox(Forkhead:HLH)/Panc1-Foxa2-ChIP-Seq(GSE47459)/Homer | 1.00E-05 | -1.28E+01 | 0 | 2107 | 18.95% | 6633.8 | 17.30% | 1.65% | 1.10 |
| FOXP1(Forkhead)/H9-FOXP1-ChIP-Seq(GSE31006)/Homer | 1.00E-02 | -6.82E+00 | 0.0104 | 1076 | 9.68% | 3389.3 | 8.84% | 0.84% | 1.10 |
| Erra(NR)/HepG2-Erra-ChIP-Seq(GSE31477)/Homer | 1.00E-07 | -1.65E+01 | 0 | 2760 | 24.82% | 8707 | 22.70% | 2.12% | 1.09 |
| GATA3(Zf)/iTreg-Gata3-ChIP-Seq(GSE20898)/Homer | 1.00E-07 | -1.79E+01 | 0 | 3043 | 27.37% | 9613 | 25.07% | 2.30% | 1.09 |
| PRDM1/BMI1(Zf)/Hela-PRDM1-ChIP-Seq(GSE31477)/Homer | 1.00E-02 | -5.12E+00 | 0.0509 | 1090 | 9.80% | 3492.5 | 9.11% | 0.69% | 1.08 |
| NeuroD1(bHLH)/Islet-NeuroD1-ChIP-Seq(GSE30298)/Homer | 1.00E-02 | -4.85E+00 | 0.0643 | 1134 | 10.20% | 3649.2 | 9.52% | 0.68% | 1.07 |
| Foxo1(Forkhead)/RAW-Foxo1-ChIP-Seq(Fan et al.)/Homer | 1.00E-02 | -6.58E+00 | 0.0126 | 3548 | 31.91% | 11731.1 | 30.59% | 1.32% | 1.04 |
| EHF(ETS)/LoVo-EHF-ChIP-Seq(GSE49402)/Homer | 1.00E-02 | -4.71E+00 | 0.0686 | 2833 | 25.48% | 9397.1 | 24.50% | 0.98% | 1.04 |
| Olig2(bHLH)/Neuron-Olig2-ChIP-Seq(GSE30882)/Homer | 1.00E-02 | -4.77E+00 | 0.0675 | 2851 | 25.64% | 9455.6 | 24.66% | 0.98% | 1.04 |

Supplementary Table 9. Motifs enriched in FAIRE peaks within 25 kb of genes downregulated after 24 h differentiation.

| Peaks within 25 kb of Genes Downregulated after 24 h Differemtaiation | P-value | Log P-value | q-value (Benjamini) | # of Target Sequences with Motif (of 497) | % of Target Sequences with Motif | # of Background Sequences with Motif (of 28492) | % of Background Sequences with Motif | % Target >Background | Fold Target >Background |
| --- | --- | --- | --- | --- | --- | --- | --- | --- | --- |
| **Hnf1(Homeobox)/Liver-Foxa2-Chip-Seq(GSE25694)/Homer** | **1.00E-02** | **-4.91E+00** | **0.1992** | **23** | **4.63%** | **750.3** | **2.63%** | **2.00%** | **1.76** |
| **Ets1-distal(ETS)/CD4+-PolII-ChIP-Seq(Barski et al.)/Homer** | **1.00E-03** | **-8.18E+00** | **0.0381** | **50** | **10.06%** | **1706.9** | **5.99%** | **4.07%** | **1.68** |
| **Elk4(ETS)/Hela-Elk4-ChIP-Seq(GSE31477)/Homer** | **1.00E-03** | **-7.66E+00** | **0.0386** | **58** | **11.67%** | **2113.5** | **7.42%** | **4.25%** | **1.57** |
| **EWS:FLI1-fusion(ETS)/SK_N_MC-EWS:FLI1-ChIP-Seq(SRA014231)/Homer** | **1.00E-02** | **-4.92E+00** | **0.1992** | **68** | **13.68%** | **2891.5** | **10.15%** | **3.53%** | **1.35** |
| **EWS:ERG-fusion(ETS)/CADO_ES1-EWS:ERG-ChIP-Seq(SRA014231)/Homer** | **1.00E-03** | **-7.08E+00** | **0.0516** | **104** | **20.93%** | **4424.9** | **15.53%** | **5.40%** | **1.35** |
| **Fli1(ETS)/CD8-FLI-ChIP-Seq(GSE20898)/Homer** | **1.00E-02** | **-6.18E+00** | **0.0851** | **116** | **23.34%** | **5168.9** | **18.14%** | **5.20%** | **1.29** |
| **GABPA(ETS)/Jurkat-GABPa-ChIP-Seq(GSE17954)/Homer** | **1.00E-02** | **-4.69E+00** | **0.206** | **94** | **18.91%** | **4257.5** | **14.94%** | **3.97%** | **1.27** |
| **Jun-AP1(bZIP)/K562-cJun-ChIP-Seq(GSE31477)/Homer** | **1.00E-02** | **-5.37E+00** | **0.1438** | **111** | **22.33%** | **5034.1** | **17.67%** | **4.66%** | **1.26** |
| BATF(bZIP)/Th17-BATF-ChIP-Seq(GSE39756)/Homer | 1.00E-03 | -8.77E+00 | 0.0381 | 211 | 42.45% | 9844 | 34.55% | 7.90% | 1.23 |
| AP-1(bZIP)/ThioMac-PU.1-ChIP-Seq(GSE21512)/Homer | 1.00E-02 | -6.78E+00 | 0.0557 | 213 | 42.86% | 10288.6 | 36.11% | 6.75% | 1.19 |
| Atf3(bZIP)/GBM-ATF3-ChIP-Seq(GSE33912)/Homer | 1.00E-02 | -5.56E+00 | 0.1349 | 204 | 41.05% | 10023.4 | 35.18% | 5.87% | 1.17 |

Supplementary Table 10. Motifs enriched in FAIRE peaks within 25 kb of genes upregulated after 24 h differentiation.

| Peaks within 25 kb of Genes Upregulated in 24 h Treated | P-value | Log P-value | q-value (Benjamini) | # of Target Sequences with Motif (of 561) | % of Target Sequences with Motif | # of Background Sequences with Motif (of 26997) | % of Background Sequences with Motif | % Target >Background | Fold Target >Background |
| --- | --- | --- | --- | --- | --- | --- | --- | --- | --- |
| **GRHL2(CP2)/HBE-GRHL2-ChIP-Seq(GSE46194)/Homer** | **1.00E-07** | **-1.65E+01** | **0** | **75** | **13.37%** | **1887.1** | **6.99%** | **6.38%** | **1.91** |
| **PPARE(NR/DR1)/3T3L1-Pparg-ChIP-Seq(GSE13511)/Homer** | **1.00E-03** | **-7.43E+00** | **0.0726** | **92** | **16.40%** | **3160.1** | **11.71%** | **4.69%** | **1.40** |
| **CEBP(bZIP)/CEBPb-ChIP-Seq(GSE21512)/Homer** | **1.00E-03** | **-7.44E+00** | **0.0726** | **98** | **17.47%** | **3410.2** | **12.63%** | **4.84%** | **1.38** |
| **GATA-IR3(Zf)/iTreg-Gata3-ChIP-Seq(GSE20898)/Homer** | **1.00E-02** | **-4.75E+00** | **0.3555** | **19** | **3.39%** | **494.6** | **1.83%** | **1.56%** | **1.85** |
| **Foxh1(Forkhead)/hESC-FOXH1-ChIP-Seq(GSE29422)/Homer** | **1.00E-02** | **-5.01E+00** | **0.3294** | **64** | **11.41%** | **2245.4** | **8.32%** | **3.09%** | **1.37** |
| **HOXA9(Homeobox)/HSC-Hoxa9-ChIP-Seq(GSE33509)/Homer** | **1.00E-02** | **-5.81E+00** | **0.1853** | **78** | **13.90%** | **2744** | **10.16%** | **3.74%** | **1.37** |

Supplementary Table 11. Motifs enriched in FAIRE peaks within 25 kb of genes downregulated after 144 h differentiation.

| Peaks within 25 kb of Genes Downregulated in 144 h Treated | P-value | log P-pvalue | q-value (Benjamini) | # Target Sequences with Motif | % of Targets Sequences with Motif | # Background Sequences with Motif | % of Background Sequences with Motif | % Target >Background | Fold Target >Background |
| --- | --- | --- | --- | --- | --- | --- | --- | --- | --- |
| **NFkB-p65-Rel(RHD)/LPS-exp(GSE23622)/Homer** | **0.01** | **-5.093** | **0.0839** | **18** | **1.63%** | **227.9** | **0.84%** | **0.79%** | **1.94** |
| **NF1:FOXA1/LNCAP-FOXA1-ChIP-Seq(GSE27824)/Homer** | **0.01** | **-5.153** | **0.0837** | **20** | **1.82%** | **261** | **0.96%** | **0.86%** | **1.90** |
| **ETS:RUNX/Jurkat-RUNX1-ChIP-Seq(GSE17954)/Homer** | **0.01** | **-5.026** | **0.085** | **26** | **2.36%** | **375.7** | **1.38%** | **0.98%** | **1.71** |
| **Ets1-distal(ETS)/CD4+-PolII-ChIP-Seq(Barski et al.)/Homer** | **0.0001** | **-9.385** | **0.0102** | **103** | **9.36%** | **1738.3** | **6.38%** | **2.98%** | **1.47** |
| **Hnf1(Homeobox)/Liver-Foxa2-Chip-Seq(GSE25694)/Homer** | **0.01** | **-4.661** | **0.1097** | **44** | **4.00%** | **746.4** | **2.74%** | **1.26%** | **1.46** |
| **Elk4(ETS)/Hela-Elk4-ChIP-Seq(GSE31477)/Homer** | **0.01** | **-6.548** | **0.0392** | **115** | **10.45%** | **2149.1** | **7.88%** | **2.57%** | **1.33** |
| **ETS(ETS)/Promoter/Homer** | **0.01** | **-4.727** | **0.1089** | **77** | **6.99%** | **1442.7** | **5.29%** | **1.70%** | **1.32** |
| **Elk1(ETS)/Hela-Elk1-ChIP-Seq(GSE31477)/Homer** | **0.01** | **-6.238** | **0.0437** | **119** | **10.81%** | **2257.6** | **8.28%** | **2.53%** | **1.31** |
| **STAT6/Macrophage-Stat6-ChIP-Seq(GSE38377)/Homer** | **0.01** | **-6.15** | **0.0437** | **122** | **11.08%** | **2329.4** | **8.54%** | **2.54%** | **1.30** |
| **STAT6(Stat)/CD4-Stat6-ChIP-Seq(GSE22104)/Homer** | **0.01** | **-5.529** | **0.0697** | **122** | **11.08%** | **2374** | **8.71%** | **2.37%** | **1.27** |
| **PU.1(ETS)/ThioMac-PU.1-ChIP-Seq(GSE21512)/Homer** | **0.01** | **-5.356** | **0.0726** | **128** | **11.63%** | **2521.2** | **9.25%** | **2.38%** | **1.26** |
| **p63(p53)/Keratinocyte-p63-ChIP-Seq(GSE17611)/Homer** | **0.01** | **-4.671** | **0.1097** | **109** | **9.90%** | **2151.4** | **7.89%** | **2.01%** | **1.25** |
| **ELF1(ETS)/Jurkat-ELF1-ChIP-Seq(SRA014231)/Homer** | **0.01** | **-4.65** | **0.1097** | **110** | **9.99%** | **2175.7** | **7.98%** | **2.01%** | **1.25** |
| Fli1(ETS)/CD8-FLI-ChIP-Seq(GSE20898)/Homer | 0.001 | -9.089 | 0.0102 | 258 | 23.43% | 5159.8 | 18.92% | 4.51% | 1.24 |
| EWS:ERG-fusion(ETS)/CADO_ES1-EWS:ERG-ChIP-Seq(SRA014231)/Homer | 0.01 | -6.417 | 0.0402 | 215 | 19.53% | 4402.7 | 16.15% | 3.38% | 1.21 |
| ETS1(ETS)/Jurkat-ETS1-ChIP-Seq(GSE17954)/Homer | 0.001 | -7.377 | 0.022 | 252 | 22.89% | 5167 | 18.95% | 3.94% | 1.21 |
| GABPA(ETS)/Jurkat-GABPa-ChIP-Seq(GSE17954)/Homer | 0.01 | -5.978 | 0.048 | 206 | 18.71% | 4236.3 | 15.54% | 3.17% | 1.20 |
| ETV1(ETS)/GIST48-ETV1-ChIP-Seq(GSE22441)/Homer | 0.001 | -7.864 | 0.0158 | 306 | 27.79% | 6374.9 | 23.38% | 4.41% | 1.19 |
| BATF(bZIP)/Th17-BATF-ChIP-Seq(GSE39756)/Homer | 0.0001 | -10.09 | 0.0102 | 425 | 38.60% | 8974.2 | 32.91% | 5.69% | 1.17 |
| Atf3(bZIP)/GBM-ATF3-ChIP-Seq(GSE33912)/Homer | 0.0001 | -9.82 | 0.0102 | 433 | 39.33% | 9190.3 | 33.71% | 5.62% | 1.17 |
| Bcl6(Zf)/Liver-Bcl6-ChIP-Seq(GSE31578)/Homer | 0.01 | -5.509 | 0.0697 | 258 | 23.43% | 5490.7 | 20.14% | 3.29% | 1.16 |
| AP-1(bZIP)/ThioMac-PU.1-ChIP-Seq(GSE21512)/Homer | 0.0001 | -9.488 | 0.0102 | 440 | 39.96% | 9390 | 34.44% | 5.52% | 1.16 |
| ERG(ETS)/VCaP-ERG-ChIP-Seq(GSE14097)/Homer | 0.001 | -7.342 | 0.022 | 360 | 32.70% | 7698 | 28.23% | 4.47% | 1.16 |
| Jun-AP1(bZIP)/K562-cJun-ChIP-Seq(GSE31477)/Homer | 0.01 | -4.617 | 0.1097 | 221 | 20.07% | 4724.6 | 17.33% | 2.74% | 1.16 |

Supplementary Table 12. Motifs enriched in FAIRE peaks within 25 kb of genes upregulated after 144 h differentiation.

| Peaks within 25 kb of Genes Upregulated in 144 h Treated | P-value | Log P-value | q-value (Benjamini) | # of Target Sequences with Motif (of 918) | % of Target Sequences with Motif | # of Background Sequences with Motif (of 27685) | % of Background Sequences with Motif | % Target >Background | Fold Target >Background |
| --- | --- | --- | --- | --- | --- | --- | --- | --- | --- |
| **LXRE(NR/DR4)/BLRP(RAW)-LXRb-ChIP-Seq(GSE21512)/Homer** | **1.00E-02** | **-5.89E+00** | **0.0567** | **13** | **1.42%** | **157.2** | **0.57%** | **0.85%** | **2.49** |
| **GRHL2(CP2)/HBE-GRHL2-ChIP-Seq(GSE46194)/Homer** | **1.00E-15** | **-3.46E+01** | **0** | **134** | **14.60%** | **1932.4** | **6.98%** | **7.62%** | **2.09** |
| **GATA-IR3(Zf)/iTreg-Gata3-ChIP-Seq(GSE20898)/Homer** | **1.00E-03** | **-7.24E+00** | **0.0195** | **34** | **3.70%** | **559.9** | **2.02%** | **1.68%** | **1.83** |
| **FOXP1(Forkhead)/H9-FOXP1-ChIP-Seq(GSE31006)/Homer** | **1.00E-03** | **-8.66E+00** | **0.0061** | **97** | **10.57%** | **2012.3** | **7.27%** | **3.30%** | **1.45** |
| **NF1(CTF)/LNCAP-NF1-ChIP-Seq(Unpublished)/Homer** | **1.00E-03** | **-8.55E+00** | **0.0061** | **105** | **11.44%** | **2222.3** | **8.03%** | **3.41%** | **1.42** |
| **FOXA1(Forkhead)/MCF7-FOXA1-ChIP-Seq(GSE26831)/Homer** | **1.00E-05** | **-1.38E+01** | **0.0001** | **215** | **23.42%** | **4760.2** | **17.21%** | **6.21%** | **1.36** |
| **FOXA1(Forkhead)/LNCAP-FOXA1-ChIP-Seq(GSE27824)/Homer** | **1.00E-06** | **-1.50E+01** | **0** | **252** | **27.45%** | **5675.9** | **20.52%** | **6.93%** | **1.34** |
| **Foxa2(Forkhead)/Liver-Foxa2-ChIP-Seq(GSE25694)/Homer** | **1.00E-04** | **-9.68E+00** | **0.0038** | **169** | **18.41%** | **3822.8** | **13.82%** | **4.59%** | **1.33** |
| **ELF5(ETS)/T47D-ELF5-ChIP-Seq(GSE30407)/Homer** | **1.00E-04** | **-9.67E+00** | **0.0038** | **179** | **19.50%** | **4091** | **14.79%** | **4.71%** | **1.32** |
| **CEBP(bZIP)/CEBPb-ChIP-Seq(GSE21512)/Homer** | **1.00E-03** | **-6.99E+00** | **0.0226** | **158** | **17.21%** | **3743.7** | **13.53%** | **3.68%** | **1.27** |
| Fox:Ebox(Forkhead:HLH)/Panc1-Foxa2-ChIP-Seq(GSE47459)/Homer | 1.00E-02 | -6.47E+00 | 0.0347 | 183 | 19.93% | 4480.6 | 16.20% | 3.73% | 1.23 |
| RXR(NR/DR1)/3T3L1-RXR-ChIP-Seq(GSE13511)/Homer | 1.00E-02 | -5.30E+00 | 0.0947 | 148 | 16.12% | 3631 | 13.12% | 3.00% | 1.23 |
| Maz(Zf)/HepG2-Maz-ChIP-Seq(GSE31477)/Homer | 1.00E-02 | -5.16E+00 | 0.0947 | 168 | 18.30% | 4201.6 | 15.19% | 3.11% | 1.20 |
| Foxo1(Forkhead)/RAW-Foxo1-ChIP-Seq(Fan et al.)/Homer | 1.00E-04 | -9.56E+00 | 0.0038 | 320 | 34.86% | 8025.8 | 29.01% | 5.85% | 1.20 |
| E2A-nearPU.1(HLH)/Bcell-PU.1-ChIP-Seq(GSE21512)/Homer | 1.00E-02 | -5.28E+00 | 0.0947 | 185 | 20.15% | 4664.3 | 16.86% | 3.29% | 1.20 |
| MYB(HTH)/ERMYB-Myb-ChIPSeq(GSE22095)/Homer | 1.00E-02 | -4.73E+00 | 0.1359 | 241 | 26.25% | 6326.3 | 22.87% | 3.38% | 1.15 |
| Erra(NR)/HepG2-Erra-ChIP-Seq(GSE31477)/Homer | 1.00E-02 | -4.63E+00 | 0.1418 | 261 | 28.43% | 6917 | 25.00% | 3.43% | 1.14 |
| EHF(ETS)/LoVo-EHF-ChIP-Seq(GSE49402)/Homer | 1.00E-02 | -4.62E+00 | 0.1418 | 269 | 29.30% | 7149.2 | 25.84% | 3.46% | 1.13 |
