# Supplementary material for: Heterarchy of transcription factors driving basal and luminal cell phenotypes in human urothelium
Source: Cell Death Differ. 2017 Mar 10;24(5):809–18. doi: 10.1038/cdd.2017.10 (PMC5423105; doi:10.1038/cdd.2017.10)
Supplement: Supplementary Tables 13-16 [file cdd201710x9.docx]

Supplementary Table 13. Motifs enriched in all FAIRE peaks containing FOXA1 unique to control cells at 24 h.

| FOXA1 Co-occuring Motifs 24 h Control | P-value | log P-pvalue | q-value (Benjamini) | # Target Sequences with Motif | % of Targets Sequences with Motif | # Background Sequences with Motif | % of Background Sequences with Motif | % Target > Background | Ratio Enrichment : Background |
| --- | --- | --- | --- | --- | --- | --- | --- | --- | --- |
| FOXA1(Forkhead)/MCF7-FOXA1-ChIP-Seq(GSE26831)/Homer | 1e-3777 | -8.70E+03 | 0 | 7362 | 85.46% | 5601.5 | 19.61% | 65.85% | 4.36 |
| FOXA1(Forkhead)/LNCAP-FOXA1-ChIP-Seq(GSE27824)/Homer | 1e-5468 | -1.26E+04 | 0 | 8615 | 100.00% | 6622.6 | 23.19% | 76.81% | 4.31 |
| Foxa2(Forkhead)/Liver-Foxa2-ChIP-Seq(GSE25694)/Homer | 1e-1759 | -4.05E+03 | 0 | 4744 | 55.07% | 3939.9 | 13.79% | 41.28% | 3.99 |
| FOXP1(Forkhead)/H9-FOXP1-ChIP-Seq(GSE31006)/Homer | 1e-543 | -1.25E+03 | 0 | 2217 | 25.73% | 2247.8 | 7.87% | 17.86% | 3.27 |
| Fox:Ebox(Forkhead:HLH)/Panc1-Foxa2-ChIP-Seq(GSE47459)/Homer | 1e-935 | -2.16E+03 | 0 | 3880 | 45.04% | 4357.5 | 15.26% | 29.78% | 2.95 |
| NF1:FOXA1/LNCAP-FOXA1-ChIP-Seq(GSE27824)/Homer | 1.00E-26 | -6.14E+01 | 0 | 169 | 1.96% | 215.5 | 0.75% | 1.21% | 2.61 |
| FOXA1:AR/LNCAP-AR-ChIP-Seq(GSE27824)/Homer | 1.00E-28 | -6.61E+01 | 0 | 240 | 2.79% | 352.6 | 1.23% | 1.56% | 2.27 |
| Pax7(Paired/Homeobox)/Myoblast-Pax7-ChIP-Seq(GSE25064)/Homer | 1.00E-17 | -3.94E+01 | 0 | 244 | 2.83% | 446.5 | 1.56% | 1.27% | 1.81 |
| Foxo1(Forkhead)/RAW-Foxo1-ChIP-Seq(Fan et al.)/Homer | 1e-393 | -9.07E+02 | 0 | 4118 | 47.80% | 7531.7 | 26.37% | 21.43% | 1.81 |
| Oct4(POU/Homeobox)/mES-Oct4-ChIP-Seq(GSE11431)/Homer | 1.00E-62 | -1.44E+02 | 0 | 1275 | 14.80% | 2619 | 9.17% | 5.63% | 1.61 |
| OCT4-SOX2-TCF-NANOG((POU/Homeobox/HMG)/mES-Oct4-ChIP-Seq(GSE11431)/Homer | 1.00E-22 | -5.13E+01 | 0 | 530 | 6.15% | 1123.4 | 3.93% | 2.22% | 1.56 |
| Foxh1(Forkhead)/hESC-FOXH1-ChIP-Seq(GSE29422)/Homer | 1.00E-52 | -1.22E+02 | 0 | 1264 | 14.67% | 2704.1 | 9.47% | 5.20% | 1.55 |
| Pax7-longest(Paired/Homeobox)/Myoblast-Pax7-ChIP-Seq(GSE25064)/Homer | 1.00E-03 | -8.76E+00 | 0.0004 | 85 | 0.99% | 185.1 | 0.65% | 0.34% | 1.52 |
| Oct2(POU/Homeobox)/Bcell-Oct2-ChIP-Seq(GSE21512)/Homer | 1.00E-30 | -7.06E+01 | 0 | 827 | 9.60% | 1810.4 | 6.34% | 3.26% | 1.51 |
| PAX5-shortForm(Paired/Homeobox)/GM12878-PAX5-ChIP-Seq(GSE32465)/Homer | 1.00E-03 | -7.01E+00 | 0.0021 | 71 | 0.82% | 158.2 | 0.55% | 0.27% | 1.49 |
| Egr2/Thymocytes-Egr2-ChIP-Seq(GSE34254)/Homer | 1.00E-02 | -6.87E+00 | 0.0024 | 78 | 0.91% | 178.6 | 0.63% | 0.28% | 1.44 |
| X-box(HTH)/NPC-H3K4me1-ChIP-Seq(GSE16256)/Homer | 1.00E-04 | -9.70E+00 | 0.0002 | 132 | 1.53% | 307.5 | 1.08% | 0.45% | 1.42 |
| Hnf1(Homeobox)/Liver-Foxa2-Chip-Seq(GSE25694)/Homer | 1.00E-12 | -2.80E+01 | 0 | 475 | 5.51% | 1125.8 | 3.94% | 1.57% | 1.40 |
| Srebp2(HLH)/HepG2-Srebp2-ChIP-Seq(GSE31477)/Homer | 1.00E-02 | -5.99E+00 | 0.0053 | 79 | 0.92% | 187.9 | 0.66% | 0.26% | 1.39 |
| Nrf2(bZIP)/Lymphoblast-Nrf2-ChIP-Seq(GSE37589)/Homer | 1.00E-05 | -1.28E+01 | 0 | 205 | 2.38% | 487.7 | 1.71% | 0.67% | 1.39 |
| HNF6(Homeobox)/Liver-Hnf6-ChIP-Seq(ERP000394)/Homer | 1.00E-23 | -5.37E+01 | 0 | 960 | 11.14% | 2296.8 | 8.04% | 3.10% | 1.39 |
| Srebp1a(HLH)/HepG2-Srebp1a-ChIP-Seq(GSE31477)/Homer | 1.00E-04 | -9.38E+00 | 0.0002 | 154 | 1.79% | 371.3 | 1.30% | 0.49% | 1.38 |
| c-Myc(HLH)/LNCAP-cMyc-ChIP-Seq(unpublished)/Homer | 1.00E-05 | -1.15E+01 | 0 | 214 | 2.48% | 524 | 1.83% | 0.65% | 1.36 |
| Pax8(Paired/Homeobox)/Thyroid-Pax8-ChIP-Seq(GSE26938)/Homer | 1.00E-04 | -1.03E+01 | 0.0001 | 206 | 2.39% | 511.3 | 1.79% | 0.60% | 1.34 |
| CLOCK(HLH)/Liver-Clock-ChIP-Seq(GSE39860)/Homer | 1.00E-07 | -1.74E+01 | 0 | 386 | 4.48% | 962.6 | 3.37% | 1.11% | 1.33 |
| Jun-AP1(bZIP)/K562-cJun-ChIP-Seq(GSE31477)/Homer | 1.00E-25 | -5.93E+01 | 0 | 1372 | 15.93% | 3442.3 | 12.05% | 3.88% | 1.32 |
| EBF(EBF)/proBcell-EBF-ChIP-Seq(GSE21978)/Homer | 1.00E-02 | -5.19E+00 | 0.0106 | 89 | 1.03% | 222.3 | 0.78% | 0.25% | 1.32 |
| Hoxb4(Homeobox)/ES-Hoxb4-ChIP-Seq(GSE34014)/Homer | 1.00E-06 | -1.54E+01 | 0 | 369 | 4.28% | 933.3 | 3.27% | 1.01% | 1.31 |
| Egr1(Zf)/K562-Egr1-ChIP-Seq(GSE32465)/Homer | 1.00E-05 | -1.17E+01 | 0 | 290 | 3.37% | 740.2 | 2.59% | 0.78% | 1.30 |
| Phox2a(Homeobox)/Neuron-Phox2a-ChIP-Seq(GSE31456)/Homer | 1.00E-16 | -3.88E+01 | 0 | 1111 | 12.90% | 2870.6 | 10.05% | 2.85% | 1.28 |
| VDR(NR/DR3)/GM10855-VDR+vitD-ChIP-Seq(GSE22484)/Homer | 1.00E-02 | -5.94E+00 | 0.0055 | 137 | 1.59% | 354.4 | 1.24% | 0.35% | 1.28 |
| HOXA2(Homeobox)/mES-Hoxa2-ChIP-Seq(Donaldson et al.)/Homer | 1.00E-02 | -6.36E+00 | 0.0038 | 159 | 1.85% | 415 | 1.45% | 0.40% | 1.28 |
| PRDM14(Zf)/H1-PRDM14-ChIP-Seq(GSE22767)/Homer | 1.00E-04 | -9.74E+00 | 0.0002 | 268 | 3.11% | 697.2 | 2.44% | 0.67% | 1.27 |
| TRa(NR)/C17.2-TRa-ChIP-Seq(GSE38347)/Homer | 1.00E-04 | -9.51E+00 | 0.0002 | 260 | 3.02% | 676.8 | 2.37% | 0.65% | 1.27 |
| Bach2(bZIP)/OCILy7-Bach2-ChIP-Seq(GSE44420)/Homer | 1.00E-10 | -2.52E+01 | 0 | 794 | 9.22% | 2077.3 | 7.27% | 1.95% | 1.27 |
| NF-E2(bZIP)/K562-NFE2-ChIP-Seq(GSE31477)/Homer | 1.00E-03 | -8.97E+00 | 0.0003 | 245 | 2.84% | 638.5 | 2.24% | 0.60% | 1.27 |
| BATF(bZIP)/Th17-BATF-ChIP-Seq(GSE39756)/Homer | 1.00E-49 | -1.13E+02 | 0 | 3001 | 34.83% | 7865.4 | 27.54% | 7.29% | 1.26 |
| Atf3(bZIP)/GBM-ATF3-ChIP-Seq(GSE33912)/Homer | 1.00E-47 | -1.09E+02 | 0 | 3012 | 34.96% | 7939.1 | 27.79% | 7.17% | 1.26 |
| Pbx3(Homeobox)/GM12878-PBX3-ChIP-Seq(GSE32465)/Homer | 1.00E-03 | -7.30E+00 | 0.0016 | 207 | 2.40% | 545.3 | 1.91% | 0.49% | 1.26 |
| MafK(bZIP)/C2C12-MafK-ChIP-Seq(GSE36030)/Homer | 1.00E-05 | -1.31E+01 | 0 | 432 | 5.01% | 1143.3 | 4.00% | 1.01% | 1.25 |
| Sox2(HMG)/mES-Sox2-ChIP-Seq(GSE11431)/Homer | 1.00E-17 | -3.92E+01 | 0 | 1365 | 15.84% | 3625.5 | 12.69% | 3.15% | 1.25 |
| Mef2c(MADS)/GM12878-Mef2c-ChIP-Seq(GSE32465)/Homer | 1.00E-12 | -2.99E+01 | 0 | 1077 | 12.50% | 2867.7 | 10.04% | 2.46% | 1.25 |
| Mef2a(MADS)/HL1-Mef2a.biotin-ChIP-Seq(GSE21529/Homer | 1.00E-10 | -2.34E+01 | 0 | 864 | 10.03% | 2307 | 8.08% | 1.95% | 1.24 |
| Ets1-distal(ETS)/CD4+-PolII-ChIP-Seq(Barski et al.)/Homer | 1.00E-05 | -1.37E+01 | 0 | 499 | 5.79% | 1335.1 | 4.67% | 1.12% | 1.24 |
| CArG(MADS)/PUER-Srf-ChIP-Seq(Sullivan et al.)/Homer | 1.00E-04 | -1.04E+01 | 0.0001 | 367 | 4.26% | 983.1 | 3.44% | 0.82% | 1.24 |
| AP-1(bZIP)/ThioMac-PU.1-ChIP-Seq(GSE21512)/Homer | 1.00E-41 | -9.54E+01 | 0 | 3060 | 35.52% | 8217 | 28.77% | 6.75% | 1.23 |
| STAT6/Macrophage-Stat6-ChIP-Seq(GSE38377)/Homer | 1.00E-10 | -2.37E+01 | 0 | 928 | 10.77% | 2495.8 | 8.74% | 2.03% | 1.23 |
| USF1(HLH)/GM12878-Usf1-ChIP-Seq(GSE32465)/Homer | 1.00E-03 | -8.04E+00 | 0.0008 | 283 | 3.28% | 762.1 | 2.67% | 0.61% | 1.23 |
| PAX3:FKHR-fusion(Paired/Homeobox)/Rh4-PAX3:FKHR-ChIP-Seq(GSE19063)/Homer | 1.00E-04 | -1.03E+01 | 0.0001 | 389 | 4.52% | 1050.9 | 3.68% | 0.84% | 1.23 |
| Tcf3(HMG)/mES-Tcf3-ChIP-Seq(GSE11724)/Homer | 1.00E-04 | -1.01E+01 | 0.0001 | 402 | 4.67% | 1091.6 | 3.82% | 0.85% | 1.22 |
| ZFX(Zf)/mES-Zfx-ChIP-Seq(GSE11431)/Homer | 1.00E-07 | -1.62E+01 | 0 | 694 | 8.06% | 1888.5 | 6.61% | 1.45% | 1.22 |
| Elk4(ETS)/Hela-Elk4-ChIP-Seq(GSE31477)/Homer | 1.00E-05 | -1.36E+01 | 0 | 573 | 6.65% | 1559 | 5.46% | 1.19% | 1.22 |
| bHLHE40(HLH)/HepG2-BHLHE40-ChIP-Seq(GSE31477)/Homer | 1.00E-02 | -4.64E+00 | 0.0177 | 152 | 1.76% | 414.7 | 1.45% | 0.31% | 1.21 |
| EBF1(EBF)/Near-E2A-ChIP-Seq(GSE21512)/Homer | 1.00E-05 | -1.19E+01 | 0 | 523 | 6.07% | 1432.4 | 5.01% | 1.06% | 1.21 |
| Tbx20(T-box)/Heart-Tbx20-ChIP-Seq(GSE29636)/Homer | 1.00E-02 | -5.27E+00 | 0.0099 | 198 | 2.30% | 544.2 | 1.91% | 0.39% | 1.20 |
| Sox6(HMG)/Myotubes-Sox6-ChIP-Seq(GSE32627)/Homer | 1.00E-26 | -6.03E+01 | 0 | 2615 | 30.35% | 7212.5 | 25.25% | 5.10% | 1.20 |
| Cdx2(Homeobox)/mES-Cdx2-ChIP-Seq(GSE14586)/Homer | 1.00E-16 | -3.75E+01 | 0 | 1782 | 20.68% | 4918.5 | 17.22% | 3.46% | 1.20 |
| STAT6(Stat)/CD4-Stat6-ChIP-Seq(GSE22104)/Homer | 1.00E-08 | -1.90E+01 | 0 | 971 | 11.27% | 2691.2 | 9.42% | 1.85% | 1.20 |
| TATA-Box(TBP)/Promoter/Homer | 1.00E-19 | -4.40E+01 | 0 | 2155 | 25.01% | 5986.1 | 20.96% | 4.05% | 1.19 |
| Usf2(HLH)/C2C12-Usf2-ChIP-Seq(GSE36030)/Homer | 1.00E-02 | -5.65E+00 | 0.0071 | 246 | 2.86% | 684.6 | 2.40% | 0.46% | 1.19 |
| GRE/RAW264.7-GRE-ChIP-Seq(Unpublished)/Homer | 1.00E-02 | -5.50E+00 | 0.008 | 242 | 2.81% | 674.9 | 2.36% | 0.45% | 1.19 |
| Tcf4(HMG)/Hct116-Tcf4-ChIP-Seq(SRA012054)/Homer | 1.00E-05 | -1.29E+01 | 0 | 684 | 7.94% | 1906.6 | 6.68% | 1.26% | 1.19 |
| Sox3(HMG)/NPC-Sox3-ChIP-Seq(GSE33059)/Homer | 1.00E-22 | -5.26E+01 | 0 | 2573 | 29.87% | 7177.8 | 25.13% | 4.74% | 1.19 |
| Elk1(ETS)/Hela-Elk1-ChIP-Seq(GSE31477)/Homer | 1.00E-04 | -1.06E+01 | 0.0001 | 558 | 6.48% | 1558.7 | 5.46% | 1.02% | 1.19 |
| Pdx1(Homeobox)/Islet-Pdx1-ChIP-Seq(SRA008281)/Homer | 1.00E-17 | -4.09E+01 | 0 | 2183 | 25.34% | 6116.8 | 21.41% | 3.93% | 1.18 |
| ZNF711(Zf)/SH-SY5Y-ZNF711-ChIP-Seq(GSE20673)/Homer | 1.00E-06 | -1.56E+01 | 0 | 890 | 10.33% | 2494.7 | 8.73% | 1.60% | 1.18 |
| Oct4:Sox17/F9-Sox17-ChIP-Seq(GSE44553)/Homer | 1.00E-02 | -6.09E+00 | 0.0049 | 306 | 3.55% | 860.5 | 3.01% | 0.54% | 1.18 |
| HOXD13(Homeobox)/Chicken-Hoxd13-ChIP-Seq(GSE38910)/Homer | 1.00E-21 | -4.93E+01 | 0 | 2611 | 30.31% | 7341.7 | 25.70% | 4.61% | 1.18 |
| bZIP:IRF/Th17-BatF-ChIP-Seq(GSE39756)/Homer | 1.00E-06 | -1.58E+01 | 0 | 959 | 11.13% | 2705 | 9.47% | 1.66% | 1.18 |
| STAT5(Stat)/mCD4+-Stat5-ChIP-Seq(GSE12346)/Homer | 1.00E-03 | -8.98E+00 | 0.0003 | 524 | 6.08% | 1480.3 | 5.18% | 0.90% | 1.17 |
| Fli1(ETS)/CD8-FLI-ChIP-Seq(GSE20898)/Homer | 1.00E-09 | -2.21E+01 | 0 | 1369 | 15.89% | 3868.4 | 13.54% | 2.35% | 1.17 |
| NFAT:AP1/Jurkat-NFATC1-ChIP-Seq(Jolma et al.)/Homer | 1.00E-02 | -6.05E+00 | 0.0051 | 341 | 3.96% | 968.3 | 3.39% | 0.57% | 1.17 |
| Arnt:Ahr(bHLH)/MCF7-Arnt-ChIP-Seq(Lo et al.)/Homer | 1.00E-03 | -7.84E+00 | 0.001 | 461 | 5.35% | 1307 | 4.58% | 0.77% | 1.17 |
| ERG(ETS)/VCaP-ERG-ChIP-Seq(GSE14097)/Homer | 1.00E-13 | -3.17E+01 | 0 | 2078 | 24.12% | 5925.8 | 20.75% | 3.37% | 1.16 |
| BMYB(HTH)/Hela-BMYB-ChIPSeq(GSE27030)/Homer | 1.00E-12 | -2.93E+01 | 0 | 1956 | 22.70% | 5580.5 | 19.54% | 3.16% | 1.16 |
| Unknown-ESC-element/mES-Nanog-ChIP-Seq(GSE11724)/Homer | 1.00E-02 | -5.86E+00 | 0.0059 | 361 | 4.19% | 1033.7 | 3.62% | 0.57% | 1.16 |
| Smad2(MAD)/ES-SMAD2-ChIP-Seq(GSE29422)/Homer | 1.00E-06 | -1.52E+01 | 0 | 1123 | 13.04% | 3223.1 | 11.28% | 1.76% | 1.16 |
| ETV1(ETS)/GIST48-ETV1-ChIP-Seq(GSE22441)/Homer | 1.00E-09 | -2.30E+01 | 0 | 1669 | 19.37% | 4787.1 | 16.76% | 2.61% | 1.16 |
| Hoxc9(Homeobox)/Ainv15-Hoxc9-ChIP-Seq(GSE21812)/Homer | 1.00E-06 | -1.49E+01 | 0 | 1101 | 12.78% | 3160.7 | 11.07% | 1.71% | 1.15 |
| Lhx2(Homeobox)/HFSC-Lhx2-ChIP-Seq(GSE48068)/Homer | 1.00E-13 | -3.22E+01 | 0 | 2270 | 26.35% | 6520.7 | 22.83% | 3.52% | 1.15 |
| ETS1(ETS)/Jurkat-ETS1-ChIP-Seq(GSE17954)/Homer | 1.00E-07 | -1.81E+01 | 0 | 1397 | 16.22% | 4023.8 | 14.09% | 2.13% | 1.15 |
| Rbpj1(?)/Panc1-Rbpj1-ChIP-Seq(GSE47459)/Homer | 1.00E-07 | -1.83E+01 | 0 | 1470 | 17.06% | 4249.6 | 14.88% | 2.18% | 1.15 |
| EWS:ERG-fusion(ETS)/CADO_ES1-EWS:ERG-ChIP-Seq(SRA014231)/Homer | 1.00E-07 | -1.65E+01 | 0 | 1354 | 15.72% | 3919.4 | 13.72% | 2.00% | 1.15 |
| MafA(bZIP)/Islet-MafA-ChIP-Seq(GSE30298)/Homer | 1.00E-04 | -1.11E+01 | 0 | 927 | 10.76% | 2689.7 | 9.42% | 1.34% | 1.14 |
| STAT1(Stat)/HelaS3-STAT1-ChIP-Seq(GSE12782)/Homer | 1.00E-02 | -6.39E+00 | 0.0038 | 492 | 5.71% | 1429.6 | 5.00% | 0.71% | 1.14 |
| AP-2alpha(AP2)/Hela-AP2alpha-ChIP-Seq(GSE31477)/Homer | 1.00E-02 | -5.44E+00 | 0.0084 | 409 | 4.75% | 1190.4 | 4.17% | 0.58% | 1.14 |
| Lhx3(Homeobox)/Neuron-Lhx3-ChIP-Seq(GSE31456)/Homer | 1.00E-21 | -4.85E+01 | 0 | 3523 | 40.89% | 10260.7 | 35.92% | 4.97% | 1.14 |
| PU.1(ETS)/ThioMac-PU.1-ChIP-Seq(GSE21512)/Homer | 1.00E-03 | -7.83E+00 | 0.001 | 660 | 7.66% | 1923.1 | 6.73% | 0.93% | 1.14 |
| NFY(CCAAT)/Promoter/Homer | 1.00E-04 | -9.44E+00 | 0.0002 | 809 | 9.39% | 2355.8 | 8.25% | 1.14% | 1.14 |
| PR(NR)/T47D-PR-ChIP-Seq(GSE31130)/Homer | 1.00E-13 | -3.08E+01 | 0 | 2541 | 29.50% | 7402.4 | 25.92% | 3.58% | 1.14 |
| Nkx3.1(Homeobox)/LNCaP-Nkx3.1-ChIP-Seq(GSE28264)/Homer | 1.00E-14 | -3.37E+01 | 0 | 2868 | 33.29% | 8395.3 | 29.39% | 3.90% | 1.13 |
| BMAL1(HLH)/Liver-Bmal1-ChIP-Seq(GSE39860)/Homer | 1.00E-07 | -1.78E+01 | 0 | 1690 | 19.62% | 4950.7 | 17.33% | 2.29% | 1.13 |
| EWS:FLI1-fusion(ETS)/SK_N_MC-EWS:FLI1-ChIP-Seq(SRA014231)/Homer | 1.00E-03 | -8.46E+00 | 0.0005 | 780 | 9.05% | 2285.3 | 8.00% | 1.05% | 1.13 |
| Smad4(MAD)/ESC-SMAD4-ChIP-Seq(GSE29422)/Homer | 1.00E-04 | -1.14E+01 | 0 | 1102 | 12.79% | 3231.5 | 11.31% | 1.48% | 1.13 |
| CRX(Homeobox)/Retina-Crx-ChIP-Seq(GSE20012)/Homer | 1.00E-17 | -4.02E+01 | 0 | 3355 | 38.94% | 9850.3 | 34.49% | 4.45% | 1.13 |
| Smad3(MAD)/NPC-Smad3-ChIP-Seq(GSE36673)/Homer | 1.00E-12 | -2.82E+01 | 0 | 2614 | 30.34% | 7682.4 | 26.90% | 3.44% | 1.13 |
| Max(HLH)/K562-Max-ChIP-Seq(GSE31477)/Homer | 1.00E-02 | -5.15E+00 | 0.011 | 470 | 5.46% | 1387.8 | 4.86% | 0.60% | 1.12 |
| Bcl6(Zf)/Liver-Bcl6-ChIP-Seq(GSE31578)/Homer | 1.00E-07 | -1.65E+01 | 0 | 1751 | 20.33% | 5170.8 | 18.10% | 2.23% | 1.12 |
| Maz(Zf)/HepG2-Maz-ChIP-Seq(GSE31477)/Homer | 1.00E-02 | -5.03E+00 | 0.0122 | 464 | 5.39% | 1372 | 4.80% | 0.59% | 1.12 |
| CHR/Cell-Cycle-Exp/Homer | 1.00E-05 | -1.35E+01 | 0 | 1465 | 17.01% | 4330.7 | 15.16% | 1.85% | 1.12 |
| Nkx6.1(Homeobox)/Islet-Nkx6.1-ChIP-Seq(GSE40975)/Homer | 1.00E-31 | -7.27E+01 | 0 | 5031 | 58.40% | 14874.2 | 52.07% | 6.33% | 1.12 |
| Nkx2.5(Homeobox)/HL1-Nkx2.5.biotin-ChIP-Seq(GSE21529)/Homer | 1.00E-09 | -2.10E+01 | 0 | 2211 | 25.66% | 6537.7 | 22.89% | 2.77% | 1.12 |
| GATA3(Zf)/iTreg-Gata3-ChIP-Seq(GSE20898)/Homer | 1.00E-10 | -2.49E+01 | 0 | 2563 | 29.75% | 7582.3 | 26.55% | 3.20% | 1.12 |
| KLF5(Zf)/LoVo-KLF5-ChIP-Seq(GSE49402)/Homer | 1.00E-02 | -6.57E+00 | 0.0032 | 674 | 7.82% | 1995.5 | 6.99% | 0.83% | 1.12 |
| Unknown(Homeobox)/Limb-p300-ChIP-Seq/Homer | 1.00E-05 | -1.18E+01 | 0 | 1363 | 15.82% | 4047.2 | 14.17% | 1.65% | 1.12 |
| Nanog(Homeobox)/mES-Nanog-ChIP-Seq(GSE11724)/Homer | 1.00E-32 | -7.56E+01 | 0 | 5378 | 62.43% | 16006.7 | 56.04% | 6.39% | 1.11 |
| Gfi1b(Zf)/HPC7-Gfi1b-ChIP-Seq(GSE22178)/Homer | 1.00E-02 | -6.84E+00 | 0.0025 | 778 | 9.03% | 2316.5 | 8.11% | 0.92% | 1.11 |
| STAT4(Stat)/CD4-Stat4-ChIP-Seq(GSE22104)/Homer | 1.00E-05 | -1.21E+01 | 0 | 1474 | 17.11% | 4391.6 | 15.37% | 1.74% | 1.11 |
| GABPA(ETS)/Jurkat-GABPa-ChIP-Seq(GSE17954)/Homer | 1.00E-03 | -8.88E+00 | 0.0004 | 1079 | 12.52% | 3217.8 | 11.27% | 1.25% | 1.11 |
| HOXA9(Homeobox)/HSC-Hoxa9-ChIP-Seq(GSE33509)/Homer | 1.00E-04 | -1.03E+01 | 0.0001 | 1361 | 15.80% | 4076.5 | 14.27% | 1.53% | 1.11 |
| MyoG(HLH)/C2C12-MyoG-ChIP-Seq(GSE36024)/Homer | 1.00E-02 | -5.37E+00 | 0.009 | 675 | 7.84% | 2028.4 | 7.10% | 0.74% | 1.10 |
| TEAD(TEA)/Fibroblast-PU.1-ChIP-Seq(Unpublished)/Homer | 1.00E-03 | -8.58E+00 | 0.0005 | 1185 | 13.76% | 3561.9 | 12.47% | 1.29% | 1.10 |
| Nkx2.1(Homeobox)/LungAC-Nkx2.1-ChIP-Seq(GSE43252)/Homer | 1.00E-09 | -2.08E+01 | 0 | 2845 | 33.02% | 8575.5 | 30.02% | 3.00% | 1.10 |
| AMYB(HTH)/Testes-AMYB-ChIP-Seq(GSE44588)/Homer | 1.00E-05 | -1.20E+01 | 0 | 1777 | 20.63% | 5359.4 | 18.76% | 1.87% | 1.10 |
| Stat3+il21(Stat)/CD4-Stat3-ChIP-Seq(GSE19198)/Homer | 1.00E-02 | -5.68E+00 | 0.007 | 804 | 9.33% | 2429.3 | 8.50% | 0.83% | 1.10 |
| Gata2(Zf)/K562-GATA2-ChIP-Seq(GSE18829)/Homer | 1.00E-02 | -6.39E+00 | 0.0038 | 921 | 10.69% | 2781.8 | 9.74% | 0.95% | 1.10 |
| SPDEF(ETS)/VCaP-SPDEF-ChIP-Seq(SRA014231)/Homer | 1.00E-03 | -7.53E+00 | 0.0013 | 1197 | 13.89% | 3628.9 | 12.70% | 1.19% | 1.09 |
| Gata4(Zf)/Heart-Gata4-ChIP-Seq(GSE35151)/Homer | 1.00E-03 | -8.94E+00 | 0.0004 | 1551 | 18.00% | 4719.2 | 16.52% | 1.48% | 1.09 |
| Isl1(Homeobox)/Neuron-Isl1-ChIP-Seq(GSE31456)/Homer | 1.00E-08 | -1.89E+01 | 0 | 3031 | 35.18% | 9222.3 | 32.29% | 2.89% | 1.09 |
| Gata1(Zf)/K562-GATA1-ChIP-Seq(GSE18829)/Homer | 1.00E-02 | -4.82E+00 | 0.0148 | 782 | 9.08% | 2384.8 | 8.35% | 0.73% | 1.09 |
| TEAD4(TEA)/Tropoblast-Tead4-ChIP-Seq(GSE37350)/Homer | 1.00E-02 | -5.91E+00 | 0.0056 | 1141 | 13.24% | 3498 | 12.25% | 0.99% | 1.08 |
| PU.1-IRF(ETS:IRF)/Bcell-PU.1-ChIP-Seq(GSE21512)/Homer | 1.00E-03 | -8.15E+00 | 0.0007 | 1706 | 19.80% | 5241.7 | 18.35% | 1.45% | 1.08 |
| Pitx1(Homeobox)/Chicken-Pitx1-ChIP-Seq(GSE38910)/Homer | 1.00E-18 | -4.33E+01 | 0 | 5602 | 65.03% | 17232.2 | 60.33% | 4.70% | 1.08 |
| Ptf1a(HLH)/Panc1-Ptf1a-ChIP-Seq(GSE47459)/Homer | 1.00E-03 | -7.59E+00 | 0.0012 | 1868 | 21.68% | 5782.5 | 20.24% | 1.44% | 1.07 |
| CEBP:AP1(bZIP)/ThioMac-CEBPb-ChIP-Seq(GSE21512)/Homer | 1.00E-02 | -5.59E+00 | 0.0074 | 1435 | 16.66% | 4456 | 15.60% | 1.06% | 1.07 |
| Erra(NR)/HepG2-Erra-ChIP-Seq(GSE31477)/Homer | 1.00E-02 | -6.04E+00 | 0.0051 | 1594 | 18.50% | 4952.5 | 17.34% | 1.16% | 1.07 |
| Tbet(T-box)/CD8-Tbet-ChIP-Seq(GSE33802)/Homer | 1.00E-02 | -5.14E+00 | 0.011 | 1374 | 15.95% | 4275.6 | 14.97% | 0.98% | 1.07 |
| NFAT(RHD)/Jurkat-NFATC1-ChIP-Seq(Jolma et al.)/Homer | 1.00E-02 | -4.87E+00 | 0.0142 | 1371 | 15.91% | 4276.1 | 14.97% | 0.94% | 1.06 |
| EHF(ETS)/LoVo-EHF-ChIP-Seq(GSE49402)/Homer | 1.00E-02 | -6.36E+00 | 0.0038 | 1906 | 22.12% | 5950.7 | 20.83% | 1.29% | 1.06 |
| Tbx5(T-box)/HL1-Tbx5.biotin-ChIP-Seq(GSE21529)/Homer | 1.00E-04 | -9.68E+00 | 0.0002 | 2903 | 33.70% | 9071.6 | 31.76% | 1.94% | 1.06 |
| Olig2(bHLH)/Neuron-Olig2-ChIP-Seq(GSE30882)/Homer | 1.00E-02 | -5.79E+00 | 0.0063 | 2028 | 23.54% | 6369.7 | 22.30% | 1.24% | 1.06 |
| MYB(HTH)/ERMYB-Myb-ChIPSeq(GSE22095)/Homer | 1.00E-02 | -5.62E+00 | 0.0072 | 2059 | 23.90% | 6477.2 | 22.68% | 1.22% | 1.05 |
| Eomes(T-box)/H9-Eomes-ChIP-Seq(GSE26097)/Homer | 1.00E-03 | -7.67E+00 | 0.0011 | 2859 | 33.19% | 9002.1 | 31.52% | 1.67% | 1.05 |
| AR-halfsite(NR)/LNCaP-AR-ChIP-Seq(GSE27824)/Homer | 1.00E-03 | -7.95E+00 | 0.0009 | 3125 | 36.27% | 9862.6 | 34.53% | 1.74% | 1.05 |
| SCL(HLH)/HPC7-Scl-ChIP-Seq(GSE13511)/Homer | 1.00E-04 | -9.52E+00 | 0.0002 | 3721 | 43.19% | 11759.2 | 41.17% | 2.02% | 1.05 |

Supplementary Table 14. Motifs enriched in all FAIRE peaks containing FOXA1 unique to differentiated cells at 24 h.

| FOXA1 Co-occuring Motifs 24 h Control | P-value | log P-pvalue | q-value (Benjamini) | # Target Sequences with Motif | % of Targets Sequences with Motif | # Background Sequences with Motif | % of Background Sequences with Motif | % Target > Background | Ratio Enrichment : Background |
| --- | --- | --- | --- | --- | --- | --- | --- | --- | --- |
| FOXA1(Forkhead)/MCF7-FOXA1-ChIP-Seq(GSE26831)/Homer | 1e-3771 | -8.68E+03 | 0 | 6077 | 84.76% | 4428.6 | 14.91% | 69.85% | 5.68 |
| FOXA1(Forkhead)/LNCAP-FOXA1-ChIP-Seq(GSE27824)/Homer | 1e-5400 | -1.24E+04 | 0 | 7170 | 100.00% | 5242 | 17.65% | 82.35% | 5.67 |
| Foxa2(Forkhead)/Liver-Foxa2-ChIP-Seq(GSE25694)/Homer | 1e-1900 | -4.38E+03 | 0 | 4179 | 58.28% | 3564.5 | 12.00% | 46.28% | 4.86 |
| FOXP1(Forkhead)/H9-FOXP1-ChIP-Seq(GSE31006)/Homer | 1e-601 | -1.39E+03 | 0 | 1947 | 27.15% | 2056.3 | 6.92% | 20.23% | 3.92 |
| Fox:Ebox(Forkhead:HLH)/Panc1-Foxa2-ChIP-Seq(GSE47459)/Homer | 1e-1288 | -2.97E+03 | 0 | 3789 | 52.85% | 4207.7 | 14.17% | 38.68% | 3.73 |
| EBNA1(EBV virus)/Raji-EBNA1-ChIP-Seq(GSE30709)/Homer | 1.00E-02 | -5.93E+00 | 0.0103 | 14 | 0.20% | 24.4 | 0.08% | 0.12% | 2.50 |
| NF1:FOXA1/LNCAP-FOXA1-ChIP-Seq(GSE27824)/Homer | 1.00E-20 | -4.83E+01 | 0 | 166 | 2.32% | 298.3 | 1.00% | 1.32% | 2.32 |
| FOXA1:AR/LNCAP-AR-ChIP-Seq(GSE27824)/Homer | 1.00E-15 | -3.63E+01 | 0 | 153 | 2.13% | 303.9 | 1.02% | 1.11% | 2.09 |
| Foxo1(Forkhead)/RAW-Foxo1-ChIP-Seq(Fan et al.)/Homer | 1e-375 | -8.65E+02 | 0 | 3729 | 52.01% | 8499.6 | 28.62% | 23.39% | 1.82 |
| Oct2(POU/Homeobox)/Bcell-Oct2-ChIP-Seq(GSE21512)/Homer | 1.00E-19 | -4.45E+01 | 0 | 548 | 7.64% | 1517.8 | 5.11% | 2.53% | 1.50 |
| Oct4:Sox17/F9-Sox17-ChIP-Seq(GSE44553)/Homer | 1.00E-07 | -1.82E+01 | 0 | 241 | 3.36% | 684.4 | 2.30% | 1.06% | 1.46 |
| GATA-DR8(Zf)/iTreg-Gata3-ChIP-Seq(GSE20898)/Homer | 1.00E-03 | -8.82E+00 | 0.001 | 111 | 1.55% | 319.5 | 1.08% | 0.47% | 1.44 |
| Oct4(POU/Homeobox)/mES-Oct4-ChIP-Seq(GSE11431)/Homer | 1.00E-21 | -4.98E+01 | 0 | 810 | 11.30% | 2382.9 | 8.02% | 3.28% | 1.41 |
| Foxh1(Forkhead)/hESC-FOXH1-ChIP-Seq(GSE29422)/Homer | 1.00E-21 | -4.97E+01 | 0 | 952 | 13.28% | 2889.4 | 9.73% | 3.55% | 1.36 |
| ISRE(IRF)/ThioMac-LPS-exp(GSE23622)/HOMER | 1.00E-03 | -7.24E+00 | 0.0036 | 117 | 1.63% | 355.5 | 1.20% | 0.43% | 1.36 |
| CEBP(bZIP)/CEBPb-ChIP-Seq(GSE21512)/Homer | 1.00E-12 | -2.81E+01 | 0 | 1355 | 18.90% | 4680.4 | 15.76% | 3.14% | 1.20 |
| TRa(NR)/C17.2-TRa-ChIP-Seq(GSE38347)/Homer | 1.00E-02 | -6.36E+00 | 0.0072 | 308 | 4.30% | 1077.9 | 3.63% | 0.67% | 1.18 |
| Nur77(NR)/K562-NR4A1-ChIP-Seq(GSE31363)/Homer | 1.00E-02 | -5.02E+00 | 0.0216 | 235 | 3.28% | 825.5 | 2.78% | 0.50% | 1.18 |
| RBPJ:Ebox/Panc1-Rbpj1-ChIP-Seq(GSE47459)/Homer | 1.00E-02 | -5.19E+00 | 0.0191 | 250 | 3.49% | 880 | 2.96% | 0.53% | 1.18 |
| RUNX(Runt)/HPC7-Runx1-ChIP-Seq(GSE22178)/Homer | 1.00E-06 | -1.58E+01 | 0 | 982 | 13.70% | 3472.7 | 11.69% | 2.01% | 1.17 |
| PRDM1/BMI1(Zf)/Hela-PRDM1-ChIP-Seq(GSE31477)/Homer | 1.00E-04 | -1.10E+01 | 0.0001 | 720 | 10.04% | 2562.8 | 8.63% | 1.41% | 1.16 |
| GATA-IR3(Zf)/iTreg-Gata3-ChIP-Seq(GSE20898)/Homer | 1.00E-05 | -1.17E+01 | 0.0001 | 230 | 3.21% | 709 | 2.39% | 0.82% | 1.34 |
| IRF2(IRF)/Erythroblas-IRF2-ChIP-Seq(GSE36985)/Homer | 1.00E-03 | -9.08E+00 | 0.0008 | 174 | 2.43% | 538.2 | 1.81% | 0.62% | 1.34 |
| GRHL2(CP2)/HBE-GRHL2-ChIP-Seq(GSE46194)/Homer | 1.00E-10 | -2.51E+01 | 0 | 640 | 8.93% | 2032.7 | 6.85% | 2.08% | 1.30 |
| Bcl6(Zf)/Liver-Bcl6-ChIP-Seq(GSE31578)/Homer | 1.00E-10 | -2.44E+01 | 0 | 1669 | 23.28% | 5970.9 | 20.11% | 3.17% | 1.16 |
| Tbx20(T-box)/Heart-Tbx20-ChIP-Seq(GSE29636)/Homer | 1.00E-04 | -1.04E+01 | 0.0002 | 252 | 3.51% | 804.2 | 2.71% | 0.80% | 1.30 |
| Tlx?(NR)/NPC-H3K4me1-ChIP-Seq(GSE16256)/Homer | 1.00E-02 | -6.29E+00 | 0.0076 | 430 | 6.00% | 1548.8 | 5.22% | 0.78% | 1.15 |
| NF1(CTF)/LNCAP-NF1-ChIP-Seq(Unpublished)/Homer | 1.00E-07 | -1.78E+01 | 0 | 512 | 7.14% | 1659 | 5.59% | 1.55% | 1.28 |
| EKLF(Zf)/Erythrocyte-Klf1-ChIP-Seq(GSE20478)/Homer | 1.00E-03 | -7.23E+00 | 0.0036 | 204 | 2.85% | 671.7 | 2.26% | 0.59% | 1.26 |
| Nr5a2(NR)/mES-Nr5a2-ChIP-Seq(GSE19019)/Homer | 1.00E-02 | -5.22E+00 | 0.0187 | 347 | 4.84% | 1252.8 | 4.22% | 0.62% | 1.15 |
| RUNX-AML(Runt)/CD4+-PolII-ChIP-Seq(Barski et al.)/Homer | 1.00E-05 | -1.25E+01 | 0 | 993 | 13.85% | 3588.3 | 12.08% | 1.77% | 1.15 |
| HNF6(Homeobox)/Liver-Hnf6-ChIP-Seq(ERP000394)/Homer | 1.00E-03 | -7.93E+00 | 0.0021 | 612 | 8.54% | 2215.7 | 7.46% | 1.08% | 1.14 |
| RUNX2(Runt)/PCa-RUNX2-ChIP-Seq(GSE33889)/Homer | 1.00E-05 | -1.32E+01 | 0 | 1113 | 15.52% | 4040.2 | 13.61% | 1.91% | 1.14 |
| STAT1(Stat)/HelaS3-STAT1-ChIP-Seq(GSE12782)/Homer | 1.00E-02 | -5.45E+00 | 0.0158 | 420 | 5.86% | 1530.2 | 5.15% | 0.71% | 1.14 |
| AP-2alpha(AP2)/Hela-AP2alpha-ChIP-Seq(GSE31477)/Homer | 1.00E-03 | -7.05E+00 | 0.004 | 584 | 8.15% | 2128 | 7.17% | 0.98% | 1.14 |
| MyoG(HLH)/C2C12-MyoG-ChIP-Seq(GSE36024)/Homer | 1.00E-04 | -9.51E+00 | 0.0006 | 848 | 11.83% | 3096.3 | 10.43% | 1.40% | 1.13 |
| Stat3+il21(Stat)/CD4-Stat3-ChIP-Seq(GSE19198)/Homer | 1.00E-03 | -9.20E+00 | 0.0007 | 839 | 11.70% | 3069.7 | 10.34% | 1.36% | 1.13 |
| EBF1(EBF)/Near-E2A-ChIP-Seq(GSE21512)/Homer | 1.00E-03 | -7.42E+00 | 0.0031 | 675 | 9.41% | 2474.9 | 8.33% | 1.08% | 1.13 |
| Myf5(bHLH)/GM-Myf5-ChIP-Seq(GSE24852)/Homer | 1.00E-02 | -6.39E+00 | 0.0072 | 569 | 7.94% | 2088 | 7.03% | 0.91% | 1.13 |
| MyoD(HLH)/Myotube-MyoD-ChIP-Seq(GSE21614)/Homer | 1.00E-02 | -6.58E+00 | 0.0062 | 603 | 8.41% | 2215.4 | 7.46% | 0.95% | 1.13 |
| IRF4(IRF)/GM12878-IRF4-ChIP-Seq(GSE32465)/Homer | 1.00E-02 | -5.99E+00 | 0.0098 | 571 | 7.96% | 2106.1 | 7.09% | 0.87% | 1.12 |
| AP-2gamma(AP2)/MCF7-TFAP2C-ChIP-Seq(GSE21234)/Homer | 1.00E-03 | -7.60E+00 | 0.0027 | 756 | 10.54% | 2787.6 | 9.39% | 1.15% | 1.12 |
| PPARE(NR/DR1)/3T3L1-Pparg-ChIP-Seq(GSE13511)/Homer | 1.00E-03 | -7.22E+00 | 0.0036 | 753 | 10.50% | 2786.5 | 9.38% | 1.12% | 1.12 |
| CEBP:AP1(bZIP)/ThioMac-CEBPb-ChIP-Seq(GSE21512)/Homer | 1.00E-05 | -1.16E+01 | 0.0001 | 1262 | 17.60% | 4670.3 | 15.73% | 1.87% | 1.12 |
| KLF5(Zf)/LoVo-KLF5-ChIP-Seq(GSE49402)/Homer | 1.00E-03 | -9.07E+00 | 0.0008 | 981 | 13.68% | 3631.9 | 12.23% | 1.45% | 1.12 |
| STAT4(Stat)/CD4-Stat4-ChIP-Seq(GSE22104)/Homer | 1.00E-05 | -1.18E+01 | 0.0001 | 1293 | 18.03% | 4788.8 | 16.13% | 1.90% | 1.12 |
| Arnt:Ahr(bHLH)/MCF7-Arnt-ChIP-Seq(Lo et al.)/Homer | 1.00E-02 | -5.17E+00 | 0.0191 | 512 | 7.14% | 1898.9 | 6.39% | 0.75% | 1.12 |
| Gfi1b(Zf)/HPC7-Gfi1b-ChIP-Seq(GSE22178)/Homer | 1.00E-02 | -6.49E+00 | 0.0067 | 734 | 10.24% | 2733 | 9.20% | 1.04% | 1.11 |
| Ap4(HLH)/AML-Tfap4-ChIP-Seq(GSE45738)/Homer | 1.00E-03 | -9.09E+00 | 0.0008 | 1075 | 14.99% | 4002.1 | 13.48% | 1.51% | 1.11 |
| PR(NR)/T47D-PR-ChIP-Seq(GSE31130)/Homer | 1.00E-08 | -2.07E+01 | 0 | 2324 | 32.41% | 8658.8 | 29.16% | 3.25% | 1.11 |
| RXR(NR/DR1)/3T3L1-RXR-ChIP-Seq(GSE13511)/Homer | 1.00E-03 | -7.16E+00 | 0.0037 | 840 | 11.72% | 3131.8 | 10.55% | 1.17% | 1.11 |
| Nr5a2(NR)/Pancreas-LRH1-ChIP-Seq(GSE34295)/Homer | 1.00E-02 | -4.73E+00 | 0.0278 | 500 | 6.97% | 1864.2 | 6.28% | 0.69% | 1.11 |
| NeuroD1(bHLH)/Islet-NeuroD1-ChIP-Seq(GSE30298)/Homer | 1.00E-02 | -6.20E+00 | 0.0082 | 713 | 9.94% | 2659.4 | 8.96% | 0.98% | 1.11 |
| Atf4(bZIP)/MEF-Atf4-ChIP-Seq(GSE35681)/Homer | 1.00E-02 | -4.76E+00 | 0.0272 | 518 | 7.22% | 1934.5 | 6.51% | 0.71% | 1.11 |
| NF1-halfsite(CTF)/LNCaP-NF1-ChIP-Seq(Unpublished)/Homer | 1.00E-06 | -1.48E+01 | 0 | 1816 | 25.33% | 6781.3 | 22.84% | 2.49% | 1.11 |
| E2A(HLH)/proBcell-E2A-ChIP-Seq(GSE21978)/Homer | 1.00E-03 | -8.09E+00 | 0.0019 | 1015 | 14.16% | 3795.5 | 12.78% | 1.38% | 1.11 |
| Mef2c(MADS)/GM12878-Mef2c-ChIP-Seq(GSE32465)/Homer | 1.00E-02 | -5.27E+00 | 0.018 | 637 | 8.88% | 2388.1 | 8.04% | 0.84% | 1.10 |
| Erra(NR)/HepG2-Erra-ChIP-Seq(GSE31477)/Homer | 1.00E-05 | -1.23E+01 | 0 | 1718 | 23.96% | 6465.5 | 21.77% | 2.19% | 1.10 |
| Olig2(bHLH)/Neuron-Olig2-ChIP-Seq(GSE30882)/Homer | 1.00E-05 | -1.36E+01 | 0 | 1914 | 26.69% | 7209.8 | 24.28% | 2.41% | 1.10 |
| Ptf1a(HLH)/Panc1-Ptf1a-ChIP-Seq(GSE47459)/Homer | 1.00E-06 | -1.40E+01 | 0 | 2135 | 29.78% | 8085.1 | 27.23% | 2.55% | 1.09 |
| E2A-nearPU.1(HLH)/Bcell-PU.1-ChIP-Seq(GSE21512)/Homer | 1.00E-02 | -6.39E+00 | 0.0072 | 1021 | 14.24% | 3876.3 | 13.05% | 1.19% | 1.09 |
| RUNX1(Runt)/Jurkat-RUNX1-ChIP-Seq(GSE29180)/Homer | 1.00E-03 | -7.78E+00 | 0.0023 | 1326 | 18.49% | 5045.4 | 16.99% | 1.50% | 1.09 |
| Atoh1(bHLH)/Cerebellum-Atoh1-ChIP-Seq(GSE22111)/Homer | 1.00E-02 | -5.44E+00 | 0.0158 | 886 | 12.36% | 3372.8 | 11.36% | 1.00% | 1.09 |
| Rbpj1(?)/Panc1-Rbpj1-ChIP-Seq(GSE47459)/Homer | 1.00E-03 | -7.46E+00 | 0.0031 | 1417 | 19.76% | 5422.5 | 18.26% | 1.50% | 1.08 |
| ZNF711(Zf)/SH-SY5Y-ZNF711-ChIP-Seq(GSE20673)/Homer | 1.00E-02 | -6.07E+00 | 0.0092 | 1134 | 15.82% | 4340.1 | 14.62% | 1.20% | 1.08 |
| ELF5(ETS)/T47D-ELF5-ChIP-Seq(GSE30407)/Homer | 1.00E-02 | -5.92E+00 | 0.0103 | 1128 | 15.73% | 4322.8 | 14.56% | 1.17% | 1.08 |
| BMYB(HTH)/Hela-BMYB-ChIPSeq(GSE27030)/Homer | 1.00E-03 | -8.03E+00 | 0.002 | 1702 | 23.74% | 6547 | 22.05% | 1.69% | 1.08 |
| Sox2(HMG)/mES-Sox2-ChIP-Seq(GSE11431)/Homer | 1.00E-02 | -5.38E+00 | 0.0165 | 1125 | 15.69% | 4332.3 | 14.59% | 1.10% | 1.08 |
| CRX(Homeobox)/Retina-Crx-ChIP-Seq(GSE20012)/Homer | 1.00E-05 | -1.26E+01 | 0 | 2615 | 36.47% | 10076.6 | 33.93% | 2.54% | 1.07 |
| GATA3(Zf)/iTreg-Gata3-ChIP-Seq(GSE20898)/Homer | 1.00E-03 | -7.90E+00 | 0.0021 | 1869 | 26.07% | 7227.6 | 24.34% | 1.73% | 1.07 |
| Sox3(HMG)/NPC-Sox3-ChIP-Seq(GSE33059)/Homer | 1.00E-03 | -8.80E+00 | 0.001 | 2149 | 29.97% | 8325.2 | 28.04% | 1.93% | 1.07 |
| SCL(HLH)/HPC7-Scl-ChIP-Seq(GSE13511)/Homer | 1.00E-08 | -1.93E+01 | 0 | 3866 | 53.92% | 14999.9 | 50.51% | 3.41% | 1.07 |
| EHF(ETS)/LoVo-EHF-ChIP-Seq(GSE49402)/Homer | 1.00E-03 | -7.43E+00 | 0.0031 | 1893 | 26.40% | 7345 | 24.74% | 1.66% | 1.07 |
| AMYB(HTH)/Testes-AMYB-ChIP-Seq(GSE44588)/Homer | 1.00E-02 | -5.08E+00 | 0.0207 | 1641 | 22.89% | 6432 | 21.66% | 1.23% | 1.06 |
| AR-halfsite(NR)/LNCaP-AR-ChIP-Seq(GSE27824)/Homer | 1.00E-04 | -1.07E+01 | 0.0002 | 3263 | 45.51% | 12803.6 | 43.12% | 2.39% | 1.06 |
| Nkx3.1(Homeobox)/LNCaP-Nkx3.1-ChIP-Seq(GSE28264)/Homer | 1.00E-02 | -6.60E+00 | 0.0062 | 2369 | 33.04% | 9319.6 | 31.38% | 1.66% | 1.05 |
| Nkx2.1(Homeobox)/LungAC-Nkx2.1-ChIP-Seq(GSE43252)/Homer | 1.00E-02 | -5.49E+00 | 0.0154 | 2568 | 35.82% | 10192.3 | 34.32% | 1.50% | 1.04 |
| Nanog(Homeobox)/mES-Nanog-ChIP-Seq(GSE11724)/Homer | 1.00E-02 | -4.95E+00 | 0.023 | 4349 | 60.66% | 17587.8 | 59.23% | 1.43% | 1.02 |

Supplementary Table 15. Motifs enriched in all FAIRE peaks containing FOXA1 unique to control cells at 144 h.

| FOXA1 Co-occuring Motifs 144 h Control | P-value | log P-pvalue | q-value (Benjamini) | # Target Sequences with Motif | % of Targets Sequences with Motif | # Background Sequences with Motif | % of Background Sequences with Motif | % Target > Background | Ratio Enrichment : Background |
| --- | --- | --- | --- | --- | --- | --- | --- | --- | --- |
| FOXA1(Forkhead)/LNCAP-FOXA1-ChIP-Seq(GSE27824)/Homer | 1e-6885 | -1.59E+04 | 0 | 10082 | 100.00% | 5679.7 | 20.75% | 79.25% | 4.82 |
| FOXA1(Forkhead)/MCF7-FOXA1-ChIP-Seq(GSE26831)/Homer | 1e-4593 | -1.06E+04 | 0 | 8518 | 84.49% | 4923.4 | 17.99% | 66.50% | 4.70 |
| Foxa2(Forkhead)/Liver-Foxa2-ChIP-Seq(GSE25694)/Homer | 1e-2120 | -4.88E+03 | 0 | 5666 | 56.20% | 3846 | 14.05% | 42.15% | 4.00 |
| FOXP1(Forkhead)/H9-FOXP1-ChIP-Seq(GSE31006)/Homer | 1e-715 | -1.65E+03 | 0 | 2714 | 26.92% | 2141.4 | 7.82% | 19.10% | 3.44 |
| Fox:Ebox(Forkhead:HLH)/Panc1-Foxa2-ChIP-Seq(GSE47459)/Homer | 1e-1344 | -3.10E+03 | 0 | 4917 | 48.77% | 4201.8 | 15.35% | 33.42% | 3.18 |
| NF1:FOXA1/LNCAP-FOXA1-ChIP-Seq(GSE27824)/Homer | 1.00E-23 | -5.42E+01 | 0 | 228 | 2.26% | 293.3 | 1.07% | 1.19% | 2.11 |
| FOXA1:AR/LNCAP-AR-ChIP-Seq(GSE27824)/Homer | 1.00E-20 | -4.64E+01 | 0 | 238 | 2.36% | 332.3 | 1.21% | 1.15% | 1.95 |
| Foxo1(Forkhead)/RAW-Foxo1-ChIP-Seq(Fan et al.)/Homer | 1e-453 | -1.05E+03 | 0 | 5102 | 50.61% | 7919.5 | 28.94% | 21.67% | 1.75 |
| p53(p53)/Saos-p53-ChIP-Seq(GSE15780)/Homer | 1.00E-26 | -6.19E+01 | 0 | 486 | 4.82% | 780.2 | 2.85% | 1.97% | 1.69 |
| p53(p53)/Saos-p53-ChIP-Seq/Homer | 1.00E-26 | -6.19E+01 | 0 | 486 | 4.82% | 780.2 | 2.85% | 1.97% | 1.69 |
| Oct4:Sox17/F9-Sox17-ChIP-Seq(GSE44553)/Homer | 1.00E-16 | -3.70E+01 | 0 | 372 | 3.69% | 641.1 | 2.34% | 1.35% | 1.58 |
| Oct4(POU/Homeobox)/mES-Oct4-ChIP-Seq(GSE11431)/Homer | 1.00E-52 | -1.21E+02 | 0 | 1326 | 13.15% | 2346.6 | 8.57% | 4.58% | 1.53 |
| p63(p53)/Keratinocyte-p63-ChIP-Seq(GSE17611)/Homer | 1.00E-32 | -7.46E+01 | 0 | 1019 | 10.11% | 1888 | 6.90% | 3.21% | 1.47 |
| Oct2(POU/Homeobox)/Bcell-Oct2-ChIP-Seq(GSE21512)/Homer | 1.00E-21 | -4.85E+01 | 0 | 792 | 7.86% | 1520.9 | 5.56% | 2.30% | 1.41 |
| Foxh1(Forkhead)/hESC-FOXH1-ChIP-Seq(GSE29422)/Homer | 1.00E-32 | -7.59E+01 | 0 | 1394 | 13.83% | 2748 | 10.04% | 3.79% | 1.38 |
| X-box(HTH)/NPC-H3K4me1-ChIP-Seq(GSE16256)/Homer | 1.00E-04 | -9.49E+00 | 0.0003 | 176 | 1.75% | 354.5 | 1.30% | 0.45% | 1.35 |
| Rfx2(HTH)/LoVo-RFX2-ChIP-Seq(GSE49402)/Homer | 1.00E-02 | -4.93E+00 | 0.0171 | 103 | 1.02% | 217.3 | 0.79% | 0.23% | 1.29 |
| Hnf1(Homeobox)/Liver-Foxa2-Chip-Seq(GSE25694)/Homer | 1.00E-06 | -1.59E+01 | 0 | 435 | 4.31% | 916.3 | 3.35% | 0.96% | 1.29 |
| OCT4-SOX2-TCF-NANOG((POU/Homeobox/HMG)/mES-Oct4-ChIP-Seq(GSE11431)/Homer | 1.00E-07 | -1.64E+01 | 0 | 482 | 4.78% | 1025.5 | 3.75% | 1.03% | 1.27 |
| Egr2/Thymocytes-Egr2-ChIP-Seq(GSE34254)/Homer | 1.00E-02 | -4.97E+00 | 0.0166 | 114 | 1.13% | 243.7 | 0.89% | 0.24% | 1.27 |
| BATF(bZIP)/Th17-BATF-ChIP-Seq(GSE39756)/Homer | 1.00E-53 | -1.23E+02 | 0 | 3745 | 37.15% | 8194.1 | 29.94% | 7.21% | 1.24 |
| Rfx1(HTH)/NPC-H3K4me1-ChIP-Seq(GSE16256)/Homer | 1.00E-03 | -7.46E+00 | 0.0018 | 234 | 2.32% | 510.4 | 1.87% | 0.45% | 1.24 |
| Tbx20(T-box)/Heart-Tbx20-ChIP-Seq(GSE29636)/Homer | 1.00E-03 | -8.38E+00 | 0.0009 | 292 | 2.90% | 642.7 | 2.35% | 0.55% | 1.23 |
| Pax8(Paired/Homeobox)/Thyroid-Pax8-ChIP-Seq(GSE26938)/Homer | 1.00E-03 | -8.17E+00 | 0.001 | 287 | 2.85% | 632.5 | 2.31% | 0.54% | 1.23 |
| Atf3(bZIP)/GBM-ATF3-ChIP-Seq(GSE33912)/Homer | 1.00E-51 | -1.20E+02 | 0 | 3793 | 37.62% | 8348.8 | 30.50% | 7.12% | 1.23 |
| EBF1(EBF)/Near-E2A-ChIP-Seq(GSE21512)/Homer | 1.00E-08 | -1.98E+01 | 0 | 791 | 7.85% | 1744.6 | 6.37% | 1.48% | 1.23 |
| Tcfcp2l1(CP2)/mES-Tcfcp2l1-ChIP-Seq(GSE11431)/Homer | 1.00E-02 | -6.76E+00 | 0.0034 | 220 | 2.18% | 483.8 | 1.77% | 0.41% | 1.23 |
| AP-1(bZIP)/ThioMac-PU.1-ChIP-Seq(GSE21512)/Homer | 1.00E-51 | -1.19E+02 | 0 | 3855 | 38.24% | 8517 | 31.12% | 7.12% | 1.23 |
| Bach2(bZIP)/OCILy7-Bach2-ChIP-Seq(GSE44420)/Homer | 1.00E-10 | -2.49E+01 | 0 | 1050 | 10.41% | 2328 | 8.51% | 1.90% | 1.22 |
| Egr1(Zf)/K562-Egr1-ChIP-Seq(GSE32465)/Homer | 1.00E-04 | -1.00E+01 | 0.0002 | 423 | 4.20% | 946 | 3.46% | 0.74% | 1.21 |
| AP-2gamma(AP2)/MCF7-TFAP2C-ChIP-Seq(GSE21234)/Homer | 1.00E-07 | -1.75E+01 | 0 | 855 | 8.48% | 1929.6 | 7.05% | 1.43% | 1.20 |
| PAX5(Paired/Homeobox)/GM12878-PAX5-ChIP-Seq(GSE32465)/Homer | 1.00E-03 | -8.02E+00 | 0.0011 | 348 | 3.45% | 785 | 2.87% | 0.58% | 1.20 |
| Bcl6(Zf)/Liver-Bcl6-ChIP-Seq(GSE31578)/Homer | 1.00E-18 | -4.25E+01 | 0 | 2266 | 22.48% | 5181 | 18.93% | 3.55% | 1.19 |
| CRE(bZIP)/Promoter/Homer | 1.00E-02 | -4.86E+00 | 0.0181 | 212 | 2.10% | 485 | 1.77% | 0.33% | 1.19 |
| ZFX(Zf)/mES-Zfx-ChIP-Seq(GSE11431)/Homer | 1.00E-08 | -1.87E+01 | 0 | 1062 | 10.53% | 2431.9 | 8.89% | 1.64% | 1.18 |
| TEAD4(TEA)/Tropoblast-Tead4-ChIP-Seq(GSE37350)/Homer | 1.00E-11 | -2.66E+01 | 0 | 1558 | 15.45% | 3580.6 | 13.08% | 2.37% | 1.18 |
| Jun-AP1(bZIP)/K562-cJun-ChIP-Seq(GSE31477)/Homer | 1.00E-12 | -2.96E+01 | 0 | 1725 | 17.11% | 3964.5 | 14.49% | 2.62% | 1.18 |
| Chop(bZIP)/MEF-Chop-ChIP-Seq(GSE35681)/Homer | 1.00E-04 | -1.05E+01 | 0.0001 | 612 | 6.07% | 1411.3 | 5.16% | 0.91% | 1.18 |
| Atf4(bZIP)/MEF-Atf4-ChIP-Seq(GSE35681)/Homer | 1.00E-05 | -1.29E+01 | 0 | 797 | 7.91% | 1843.3 | 6.73% | 1.18% | 1.18 |
| AP-2alpha(AP2)/Hela-AP2alpha-ChIP-Seq(GSE31477)/Homer | 1.00E-04 | -1.04E+01 | 0.0001 | 630 | 6.25% | 1459 | 5.33% | 0.92% | 1.17 |
| Mef2c(MADS)/GM12878-Mef2c-ChIP-Seq(GSE32465)/Homer | 1.00E-06 | -1.57E+01 | 0 | 1033 | 10.25% | 2399.2 | 8.77% | 1.48% | 1.17 |
| CEBP(bZIP)/CEBPb-ChIP-Seq(GSE21512)/Homer | 1.00E-12 | -2.89E+01 | 0 | 1931 | 19.15% | 4499 | 16.44% | 2.71% | 1.16 |
| MafK(bZIP)/C2C12-MafK-ChIP-Seq(GSE36030)/Homer | 1.00E-03 | -8.74E+00 | 0.0006 | 558 | 5.53% | 1300.9 | 4.75% | 0.78% | 1.16 |
| c-Myc(HLH)/LNCAP-cMyc-ChIP-Seq(unpublished)/Homer | 1.00E-02 | -5.02E+00 | 0.0162 | 283 | 2.81% | 661.8 | 2.42% | 0.39% | 1.16 |
| CEBP:AP1(bZIP)/ThioMac-CEBPb-ChIP-Seq(GSE21512)/Homer | 1.00E-11 | -2.60E+01 | 0 | 1827 | 18.12% | 4271.2 | 15.61% | 2.51% | 1.16 |
| KLF5(Zf)/LoVo-KLF5-ChIP-Seq(GSE49402)/Homer | 1.00E-06 | -1.58E+01 | 0 | 1136 | 11.27% | 2658.2 | 9.71% | 1.56% | 1.16 |
| Hoxb4(Homeobox)/ES-Hoxb4-ChIP-Seq(GSE34014)/Homer | 1.00E-02 | -6.49E+00 | 0.0042 | 435 | 4.31% | 1023.6 | 3.74% | 0.57% | 1.15 |
| Sox2(HMG)/mES-Sox2-ChIP-Seq(GSE11431)/Homer | 1.00E-07 | -1.68E+01 | 0 | 1549 | 15.36% | 3698.8 | 13.51% | 1.85% | 1.14 |
| STAT6(Stat)/CD4-Stat6-ChIP-Seq(GSE22104)/Homer | 1.00E-05 | -1.16E+01 | 0 | 1058 | 10.49% | 2526.3 | 9.23% | 1.26% | 1.14 |
| Ap4(HLH)/AML-Tfap4-ChIP-Seq(GSE45738)/Homer | 1.00E-06 | -1.43E+01 | 0 | 1342 | 13.31% | 3209.5 | 11.73% | 1.58% | 1.13 |
| MyoD(HLH)/Myotube-MyoD-ChIP-Seq(GSE21614)/Homer | 1.00E-03 | -8.09E+00 | 0.0011 | 716 | 7.10% | 1712.3 | 6.26% | 0.84% | 1.13 |
| RUNX-AML(Runt)/CD4+-PolII-ChIP-Seq(Barski et al.)/Homer | 1.00E-05 | -1.26E+01 | 0 | 1198 | 11.88% | 2869.3 | 10.48% | 1.40% | 1.13 |
| NF1(CTF)/LNCAP-NF1-ChIP-Seq(Unpublished)/Homer | 1.00E-02 | -6.81E+00 | 0.0033 | 584 | 5.79% | 1397.3 | 5.11% | 0.68% | 1.13 |
| Maz(Zf)/HepG2-Maz-ChIP-Seq(GSE31477)/Homer | 1.00E-03 | -8.37E+00 | 0.0009 | 764 | 7.58% | 1830.2 | 6.69% | 0.89% | 1.13 |
| Sox3(HMG)/NPC-Sox3-ChIP-Seq(GSE33059)/Homer | 1.00E-14 | -3.34E+01 | 0 | 3034 | 30.09% | 7284 | 26.61% | 3.48% | 1.13 |
| ZNF711(Zf)/SH-SY5Y-ZNF711-ChIP-Seq(GSE20673)/Homer | 1.00E-05 | -1.33E+01 | 0 | 1335 | 13.24% | 3209.6 | 11.73% | 1.51% | 1.13 |
| Mef2a(MADS)/HL1-Mef2a.biotin-ChIP-Seq(GSE21529/Homer | 1.00E-03 | -8.81E+00 | 0.0006 | 872 | 8.65% | 2099.5 | 7.67% | 0.98% | 1.13 |
| STAT4(Stat)/CD4-Stat4-ChIP-Seq(GSE22104)/Homer | 1.00E-07 | -1.72E+01 | 0 | 1764 | 17.50% | 4247.7 | 15.52% | 1.98% | 1.13 |
| STAT6/Macrophage-Stat6-ChIP-Seq(GSE38377)/Homer | 1.00E-04 | -9.93E+00 | 0.0002 | 1010 | 10.02% | 2433.8 | 8.89% | 1.13% | 1.13 |
| n-Myc(HLH)/mES-nMyc-ChIP-Seq(GSE11431)/Homer | 1.00E-02 | -5.85E+00 | 0.0075 | 551 | 5.47% | 1330.5 | 4.86% | 0.61% | 1.13 |
| Myf5(bHLH)/GM-Myf5-ChIP-Seq(GSE24852)/Homer | 1.00E-02 | -6.75E+00 | 0.0034 | 697 | 6.91% | 1688.6 | 6.17% | 0.74% | 1.12 |
| Cdx2(Homeobox)/mES-Cdx2-ChIP-Seq(GSE14586)/Homer | 1.00E-06 | -1.50E+01 | 0 | 1725 | 17.11% | 4184.7 | 15.29% | 1.82% | 1.12 |
| Sox6(HMG)/Myotubes-Sox6-ChIP-Seq(GSE32627)/Homer | 1.00E-10 | -2.42E+01 | 0 | 2903 | 28.79% | 7089.8 | 25.90% | 2.89% | 1.11 |
| Nkx3.1(Homeobox)/LNCaP-Nkx3.1-ChIP-Seq(GSE28264)/Homer | 1.00E-12 | -2.88E+01 | 0 | 3388 | 33.60% | 8286.6 | 30.28% | 3.32% | 1.11 |
| RUNX(Runt)/HPC7-Runx1-ChIP-Seq(GSE22178)/Homer | 1.00E-03 | -8.82E+00 | 0.0006 | 1156 | 11.47% | 2832.1 | 10.35% | 1.12% | 1.11 |
| Phox2a(Homeobox)/Neuron-Phox2a-ChIP-Seq(GSE31456)/Homer | 1.00E-03 | -7.47E+00 | 0.0018 | 991 | 9.83% | 2433.8 | 8.89% | 0.94% | 1.11 |
| TEAD(TEA)/Fibroblast-PU.1-ChIP-Seq(Unpublished)/Homer | 1.00E-04 | -1.05E+01 | 0.0001 | 1452 | 14.40% | 3565.3 | 13.03% | 1.37% | 1.11 |
| PU.1(ETS)/ThioMac-PU.1-ChIP-Seq(GSE21512)/Homer | 1.00E-02 | -6.61E+00 | 0.0038 | 902 | 8.95% | 2221.2 | 8.12% | 0.83% | 1.10 |
| Tcf12(HLH)/GM12878-Tcf12-ChIP-Seq(GSE32465)/Homer | 1.00E-02 | -6.42E+00 | 0.0045 | 925 | 9.17% | 2285.5 | 8.35% | 0.82% | 1.10 |
| Ets1-distal(ETS)/CD4+-PolII-ChIP-Seq(Barski et al.)/Homer | 1.00E-02 | -4.81E+00 | 0.019 | 661 | 6.56% | 1636.2 | 5.98% | 0.58% | 1.10 |
| Pdx1(Homeobox)/Islet-Pdx1-ChIP-Seq(SRA008281)/Homer | 1.00E-06 | -1.45E+01 | 0 | 2320 | 23.01% | 5748.7 | 21.00% | 2.01% | 1.10 |
| HNF6(Homeobox)/Liver-Hnf6-ChIP-Seq(ERP000394)/Homer | 1.00E-02 | -5.85E+00 | 0.0075 | 921 | 9.14% | 2288.5 | 8.36% | 0.78% | 1.09 |
| MyoG(HLH)/C2C12-MyoG-ChIP-Seq(GSE36024)/Homer | 1.00E-02 | -6.37E+00 | 0.0046 | 1019 | 10.11% | 2531.8 | 9.25% | 0.86% | 1.09 |
| Rbpj1(?)/Panc1-Rbpj1-ChIP-Seq(GSE47459)/Homer | 1.00E-04 | -1.09E+01 | 0.0001 | 1874 | 18.59% | 4659.7 | 17.03% | 1.56% | 1.09 |
| RUNX2(Runt)/PCa-RUNX2-ChIP-Seq(GSE33889)/Homer | 1.00E-03 | -7.79E+00 | 0.0014 | 1347 | 13.36% | 3353.6 | 12.25% | 1.11% | 1.09 |
| MafA(bZIP)/Islet-MafA-ChIP-Seq(GSE30298)/Homer | 1.00E-03 | -7.29E+00 | 0.0021 | 1239 | 12.29% | 3083.9 | 11.27% | 1.02% | 1.09 |
| Smad4(MAD)/ESC-SMAD4-ChIP-Seq(GSE29422)/Homer | 1.00E-03 | -8.71E+00 | 0.0006 | 1521 | 15.09% | 3786.8 | 13.84% | 1.25% | 1.09 |
| Gfi1b(Zf)/HPC7-Gfi1b-ChIP-Seq(GSE22178)/Homer | 1.00E-02 | -6.09E+00 | 0.006 | 1011 | 10.03% | 2517.6 | 9.20% | 0.83% | 1.09 |
| TATA-Box(TBP)/Promoter/Homer | 1.00E-05 | -1.29E+01 | 0 | 2404 | 23.84% | 6004.1 | 21.94% | 1.90% | 1.09 |
| Atf1(bZIP)/K562-ATF1-ChIP-Seq(GSE31477)/Homer | 1.00E-02 | -6.18E+00 | 0.0056 | 1101 | 10.92% | 2750.3 | 10.05% | 0.87% | 1.09 |
| PR(NR)/T47D-PR-ChIP-Seq(GSE31130)/Homer | 1.00E-06 | -1.52E+01 | 0 | 3101 | 30.76% | 7795.2 | 28.48% | 2.28% | 1.08 |
| Smad2(MAD)/ES-SMAD2-ChIP-Seq(GSE29422)/Homer | 1.00E-03 | -7.07E+00 | 0.0026 | 1524 | 15.12% | 3836.6 | 14.02% | 1.10% | 1.08 |
| bZIP:IRF/Th17-BatF-ChIP-Seq(GSE39756)/Homer | 1.00E-02 | -4.78E+00 | 0.0192 | 989 | 9.81% | 2494.7 | 9.11% | 0.70% | 1.08 |
| Atoh1(bHLH)/Cerebellum-Atoh1-ChIP-Seq(GSE22111)/Homer | 1.00E-02 | -5.41E+00 | 0.0114 | 1148 | 11.39% | 2894.7 | 10.58% | 0.81% | 1.08 |
| RUNX1(Runt)/Jurkat-RUNX1-ChIP-Seq(GSE29180)/Homer | 1.00E-03 | -7.15E+00 | 0.0024 | 1651 | 16.38% | 4168.1 | 15.23% | 1.15% | 1.08 |
| Stat3+il21(Stat)/CD4-Stat3-ChIP-Seq(GSE19198)/Homer | 1.00E-02 | -4.98E+00 | 0.0166 | 1049 | 10.40% | 2646.1 | 9.67% | 0.73% | 1.08 |
| BMAL1(HLH)/Liver-Bmal1-ChIP-Seq(GSE39860)/Homer | 1.00E-03 | -8.35E+00 | 0.0009 | 2115 | 20.98% | 5359.9 | 19.58% | 1.40% | 1.07 |
| E2A(HLH)/proBcell-E2A-ChIP-Seq(GSE21978)/Homer | 1.00E-02 | -5.09E+00 | 0.0154 | 1225 | 12.15% | 3106.1 | 11.35% | 0.80% | 1.07 |
| Lhx2(Homeobox)/HFSC-Lhx2-ChIP-Seq(GSE48068)/Homer | 1.00E-03 | -8.84E+00 | 0.0006 | 2302 | 22.83% | 5840.4 | 21.34% | 1.49% | 1.07 |
| Olig2(bHLH)/Neuron-Olig2-ChIP-Seq(GSE30882)/Homer | 1.00E-04 | -9.91E+00 | 0.0002 | 2593 | 25.72% | 6581.8 | 24.05% | 1.67% | 1.07 |
| Smad3(MAD)/NPC-Smad3-ChIP-Seq(GSE36673)/Homer | 1.00E-05 | -1.33E+01 | 0 | 3364 | 33.37% | 8541.6 | 31.21% | 2.16% | 1.07 |
| AMYB(HTH)/Testes-AMYB-ChIP-Seq(GSE44588)/Homer | 1.00E-03 | -8.35E+00 | 0.0009 | 2254 | 22.36% | 5727.5 | 20.93% | 1.43% | 1.07 |
| Unknown(Homeobox)/Limb-p300-ChIP-Seq/Homer | 1.00E-02 | -5.16E+00 | 0.0144 | 1393 | 13.82% | 3547.6 | 12.96% | 0.86% | 1.07 |
| SCL(HLH)/HPC7-Scl-ChIP-Seq(GSE13511)/Homer | 1.00E-08 | -2.07E+01 | 0 | 5065 | 50.24% | 12933.6 | 47.26% | 2.98% | 1.06 |
| HOXD13(Homeobox)/Chicken-Hoxd13-ChIP-Seq(GSE38910)/Homer | 1.00E-03 | -7.86E+00 | 0.0013 | 2500 | 24.80% | 6395.7 | 23.37% | 1.43% | 1.06 |
| NF1-halfsite(CTF)/LNCaP-NF1-ChIP-Seq(Unpublished)/Homer | 1.00E-02 | -6.73E+00 | 0.0035 | 2314 | 22.95% | 5937.5 | 21.69% | 1.26% | 1.06 |
| BMYB(HTH)/Hela-BMYB-ChIPSeq(GSE27030)/Homer | 1.00E-02 | -6.68E+00 | 0.0036 | 2331 | 23.12% | 5984.4 | 21.87% | 1.25% | 1.06 |
| Tbx5(T-box)/HL1-Tbx5.biotin-ChIP-Seq(GSE21529)/Homer | 1.00E-04 | -1.09E+01 | 0.0001 | 3881 | 38.49% | 9991.4 | 36.51% | 1.98% | 1.05 |
| ETS1(ETS)/Jurkat-ETS1-ChIP-Seq(GSE17954)/Homer | 1.00E-02 | -4.68E+00 | 0.0209 | 1808 | 17.93% | 4664.3 | 17.04% | 0.89% | 1.05 |
| Eomes(T-box)/H9-Eomes-ChIP-Seq(GSE26097)/Homer | 1.00E-03 | -8.41E+00 | 0.0009 | 3378 | 33.51% | 8721.2 | 31.86% | 1.65% | 1.05 |
| MYB(HTH)/ERMYB-Myb-ChIPSeq(GSE22095)/Homer | 1.00E-02 | -5.85E+00 | 0.0075 | 2542 | 25.21% | 6577 | 24.03% | 1.18% | 1.05 |
| Nkx2.1(Homeobox)/LungAC-Nkx2.1-ChIP-Seq(GSE43252)/Homer | 1.00E-03 | -8.01E+00 | 0.0011 | 3502 | 34.74% | 9067 | 33.13% | 1.61% | 1.05 |
| CRX(Homeobox)/Retina-Crx-ChIP-Seq(GSE20012)/Homer | 1.00E-03 | -7.55E+00 | 0.0017 | 3756 | 37.25% | 9766.5 | 35.68% | 1.57% | 1.04 |
| ERG(ETS)/VCaP-ERG-ChIP-Seq(GSE14097)/Homer | 1.00E-02 | -5.00E+00 | 0.0165 | 2672 | 26.50% | 6958.8 | 25.43% | 1.07% | 1.04 |
| Ptf1a(HLH)/Panc1-Ptf1a-ChIP-Seq(GSE47459)/Homer | 1.00E-02 | -4.74E+00 | 0.0198 | 2687 | 26.65% | 7009.6 | 25.61% | 1.04% | 1.04 |
| Nanog(Homeobox)/mES-Nanog-ChIP-Seq(GSE11724)/Homer | 1.00E-05 | -1.36E+01 | 0 | 6224 | 61.73% | 16265.4 | 59.43% | 2.30% | 1.04 |
| Pitx1(Homeobox)/Chicken-Pitx1-ChIP-Seq(GSE38910)/Homer | 1.00E-06 | -1.40E+01 | 0 | 6358 | 63.06% | 16623.1 | 60.74% | 2.32% | 1.04 |
| AR-halfsite(NR)/LNCaP-AR-ChIP-Seq(GSE27824)/Homer | 1.00E-02 | -6.50E+00 | 0.0042 | 4173 | 41.39% | 10930.9 | 39.94% | 1.45% | 1.04 |
| Nkx6.1(Homeobox)/Islet-Nkx6.1-ChIP-Seq(GSE40975)/Homer | 1.00E-03 | -8.09E+00 | 0.0011 | 5263 | 52.20% | 13819.9 | 50.49% | 1.71% | 1.03 |

Supplementary Table 16. Motifs enriched in all FAIRE peaks containing FOXA1 unique to differentiated cells at 144 h.

| FOXA1 Co-occuring Motifs 144 h Control | P-value | log P-pvalue | q-value (Benjamini) | # Target Sequences with Motif | % of Targets Sequences with Motif | # Background Sequences with Motif | % of Background Sequences with Motif | % Target > Background | Ratio Enrichment : Background |
| --- | --- | --- | --- | --- | --- | --- | --- | --- | --- |
| FOXA1(Forkhead)/LNCAP-FOXA1-ChIP-Seq(GSE27824)/Homer | 1e-5489 | -1.26E+04 | 0 | 7778 | 100.00% | 7535.2 | 19.69% | 80.31% | 5.08 |
| FOXA1(Forkhead)/MCF7-FOXA1-ChIP-Seq(GSE26831)/Homer | 1e-3844 | -8.85E+03 | 0 | 6630 | 85.24% | 6363.6 | 16.63% | 68.61% | 5.13 |
| Foxa2(Forkhead)/Liver-Foxa2-ChIP-Seq(GSE25694)/Homer | 1e-1924 | -4.43E+03 | 0 | 4545 | 58.43% | 4993.7 | 13.05% | 45.38% | 4.48 |
| Foxo1(Forkhead)/RAW-Foxo1-ChIP-Seq(Fan et al.)/Homer | 1e-392 | -9.03E+02 | 0 | 4086 | 52.53% | 11282.5 | 29.48% | 23.05% | 1.78 |
| Fox:Ebox(Forkhead:HLH)/Panc1-Foxa2-ChIP-Seq(GSE47459)/Homer | 1e-1215 | -2.80E+03 | 0 | 4000 | 51.43% | 5774.4 | 15.09% | 36.34% | 3.41 |
| FOXP1(Forkhead)/H9-FOXP1-ChIP-Seq(GSE31006)/Homer | 1e-613 | -1.41E+03 | 0 | 2170 | 27.90% | 2948.8 | 7.71% | 20.19% | 3.62 |
| Foxh1(Forkhead)/hESC-FOXH1-ChIP-Seq(GSE29422)/Homer | 1.00E-24 | -5.70E+01 | 0 | 1064 | 13.68% | 3818.2 | 9.98% | 3.70% | 1.37 |
| Oct4(POU/Homeobox)/mES-Oct4-ChIP-Seq(GSE11431)/Homer | 1.00E-26 | -6.16E+01 | 0 | 933 | 12.00% | 3213.1 | 8.40% | 3.60% | 1.43 |
| Oct2(POU/Homeobox)/Bcell-Oct2-ChIP-Seq(GSE21512)/Homer | 1.00E-24 | -5.75E+01 | 0 | 620 | 7.97% | 1973.9 | 5.16% | 2.81% | 1.54 |
| Ets1-distal(ETS)/CD4+-PolII-ChIP-Seq(Barski et al.)/Homer | 1.00E-08 | -1.90E+01 | 0 | 589 | 7.57% | 2286.2 | 5.97% | 1.60% | 1.27 |
| Oct4:Sox17/F9-Sox17-ChIP-Seq(GSE44553)/Homer | 1.00E-04 | -1.04E+01 | 0.0002 | 245 | 3.15% | 925 | 2.42% | 0.73% | 1.30 |
| EHF(ETS)/LoVo-EHF-ChIP-Seq(GSE49402)/Homer | 1.00E-18 | -4.32E+01 | 0 | 2175 | 27.96% | 9016.6 | 23.56% | 4.40% | 1.19 |
| FOXA1:AR/LNCAP-AR-ChIP-Seq(GSE27824)/Homer | 1.00E-21 | -4.99E+01 | 0 | 183 | 2.35% | 402.5 | 1.05% | 1.30% | 2.24 |
| ETV1(ETS)/GIST48-ETV1-ChIP-Seq(GSE22441)/Homer | 1.00E-14 | -3.30E+01 | 0 | 1929 | 24.80% | 8088.5 | 21.14% | 3.66% | 1.17 |
| GABPA(ETS)/Jurkat-GABPa-ChIP-Seq(GSE17954)/Homer | 1.00E-11 | -2.55E+01 | 0 | 1293 | 16.62% | 5322.4 | 13.91% | 2.71% | 1.19 |
| ERG(ETS)/VCaP-ERG-ChIP-Seq(GSE14097)/Homer | 1.00E-09 | -2.19E+01 | 0 | 2289 | 29.43% | 10064.2 | 26.30% | 3.13% | 1.12 |
| NF1:FOXA1/LNCAP-FOXA1-ChIP-Seq(GSE27824)/Homer | 1.00E-21 | -5.00E+01 | 0 | 176 | 2.26% | 379.7 | 0.99% | 1.27% | 2.28 |
| GATA-IR4(Zf)/iTreg-Gata3-ChIP-Seq(GSE20898)/Homer | 1.00E-03 | -7.36E+00 | 0.0028 | 159 | 2.04% | 599.1 | 1.57% | 0.47% | 1.30 |
| SPDEF(ETS)/VCaP-SPDEF-ChIP-Seq(SRA014231)/Homer | 1.00E-07 | -1.82E+01 | 0 | 1433 | 18.42% | 6143.6 | 16.06% | 2.36% | 1.15 |
| ISRE(IRF)/ThioMac-LPS-exp(GSE23622)/HOMER | 1.00E-02 | -6.16E+00 | 0.008 | 131 | 1.68% | 496.8 | 1.30% | 0.38% | 1.29 |
| ETS1(ETS)/Jurkat-ETS1-ChIP-Seq(GSE17954)/Homer | 1.00E-07 | -1.77E+01 | 0 | 1544 | 19.85% | 6674.4 | 17.44% | 2.41% | 1.14 |
| GATA-DR8(Zf)/iTreg-Gata3-ChIP-Seq(GSE20898)/Homer | 1.00E-03 | -8.82E+00 | 0.0008 | 129 | 1.66% | 453.3 | 1.18% | 0.48% | 1.41 |
| E2A-nearPU.1(HLH)/Bcell-PU.1-ChIP-Seq(GSE21512)/Homer | 1.00E-06 | -1.60E+01 | 0 | 1169 | 15.03% | 4977.3 | 13.01% | 2.02% | 1.16 |
| Ptf1a(HLH)/Panc1-Ptf1a-ChIP-Seq(GSE47459)/Homer | 1.00E-06 | -1.55E+01 | 0 | 2304 | 29.62% | 10343.8 | 27.03% | 2.59% | 1.10 |
| TR4(NR/DR1)/Hela-TR4-ChIP-Seq(GSE24685)/Homer | 1.00E-04 | -1.02E+01 | 0.0002 | 87 | 1.12% | 271 | 0.71% | 0.41% | 1.58 |
| EWS:ERG-fusion(ETS)/CADO_ES1-EWS:ERG-ChIP-Seq(SRA014231)/Homer | 1.00E-06 | -1.50E+01 | 0 | 1394 | 17.92% | 6052.3 | 15.82% | 2.10% | 1.13 |
| GATA3(Zf)/iTreg-Gata3-ChIP-Seq(GSE20898)/Homer | 1.00E-06 | -1.50E+01 | 0 | 2163 | 27.81% | 9688.2 | 25.32% | 2.49% | 1.10 |
| IRF2(IRF)/Erythroblas-IRF2-ChIP-Seq(GSE36985)/Homer | 1.00E-02 | -6.44E+00 | 0.0063 | 192 | 2.47% | 758.5 | 1.98% | 0.49% | 1.25 |
| PPARE(NR/DR1)/3T3L1-Pparg-ChIP-Seq(GSE13511)/Homer | 1.00E-06 | -1.42E+01 | 0 | 824 | 10.59% | 3438.6 | 8.99% | 1.60% | 1.18 |
| Erra(NR)/HepG2-Erra-ChIP-Seq(GSE31477)/Homer | 1.00E-06 | -1.41E+01 | 0 | 1854 | 23.84% | 8249.4 | 21.56% | 2.28% | 1.11 |
| MyoG(HLH)/C2C12-MyoG-ChIP-Seq(GSE36024)/Homer | 1.00E-05 | -1.30E+01 | 0 | 901 | 11.58% | 3820.2 | 9.98% | 1.60% | 1.16 |
| Olig2(bHLH)/Neuron-Olig2-ChIP-Seq(GSE30882)/Homer | 1.00E-05 | -1.27E+01 | 0 | 2075 | 26.68% | 9355.8 | 24.45% | 2.23% | 1.09 |
| EWS:FLI1-fusion(ETS)/SK_N_MC-EWS:FLI1-ChIP-Seq(SRA014231)/Homer | 1.00E-05 | -1.26E+01 | 0 | 905 | 11.64% | 3851 | 10.06% | 1.58% | 1.16 |
| Fli1(ETS)/CD8-FLI-ChIP-Seq(GSE20898)/Homer | 1.00E-05 | -1.17E+01 | 0.0001 | 1513 | 19.45% | 6721.5 | 17.57% | 1.88% | 1.11 |
| CRX(Homeobox)/Retina-Crx-ChIP-Seq(GSE20012)/Homer | 1.00E-04 | -1.12E+01 | 0.0001 | 2861 | 36.78% | 13202.7 | 34.50% | 2.28% | 1.07 |
| NeuroD1(bHLH)/Islet-NeuroD1-ChIP-Seq(GSE30298)/Homer | 1.00E-04 | -1.12E+01 | 0.0001 | 798 | 10.26% | 3395.4 | 8.87% | 1.39% | 1.16 |
| Elk4(ETS)/Hela-Elk4-ChIP-Seq(GSE31477)/Homer | 1.00E-04 | -1.11E+01 | 0.0001 | 666 | 8.56% | 2791 | 7.29% | 1.27% | 1.17 |
| Sox3(HMG)/NPC-Sox3-ChIP-Seq(GSE33059)/Homer | 1.00E-04 | -1.09E+01 | 0.0001 | 2332 | 29.98% | 10662.5 | 27.86% | 2.12% | 1.08 |
| Sox2(HMG)/mES-Sox2-ChIP-Seq(GSE11431)/Homer | 1.00E-04 | -1.08E+01 | 0.0001 | 1230 | 15.81% | 5419.1 | 14.16% | 1.65% | 1.12 |
| ELF5(ETS)/T47D-ELF5-ChIP-Seq(GSE30407)/Homer | 1.00E-16 | -3.87E+01 | 0 | 1361 | 17.50% | 5380.5 | 14.06% | 3.44% | 1.24 |
| GATA-IR3(Zf)/iTreg-Gata3-ChIP-Seq(GSE20898)/Homer | 1.00E-03 | -8.09E+00 | 0.0014 | 278 | 3.57% | 1108.7 | 2.90% | 0.67% | 1.23 |
| E2A(HLH)/proBcell-E2A-ChIP-Seq(GSE21978)/Homer | 1.00E-04 | -1.01E+01 | 0.0002 | 1096 | 14.09% | 4813.9 | 12.58% | 1.51% | 1.12 |
| ETS(ETS)/Promoter/Homer | 1.00E-03 | -9.11E+00 | 0.0006 | 437 | 5.62% | 1800 | 4.70% | 0.92% | 1.20 |
| SCL(HLH)/HPC7-Scl-ChIP-Seq(GSE13511)/Homer | 1.00E-03 | -9.10E+00 | 0.0006 | 4056 | 52.15% | 19151.5 | 50.05% | 2.10% | 1.04 |
| PR(NR)/T47D-PR-ChIP-Seq(GSE31130)/Homer | 1.00E-03 | -9.04E+00 | 0.0006 | 2436 | 31.32% | 11251.3 | 29.40% | 1.92% | 1.07 |
| Tbx5(T-box)/HL1-Tbx5.biotin-ChIP-Seq(GSE21529)/Homer | 1.00E-03 | -8.86E+00 | 0.0008 | 3149 | 40.49% | 14721 | 38.47% | 2.02% | 1.05 |
| ELF1(ETS)/Jurkat-ELF1-ChIP-Seq(SRA014231)/Homer | 1.00E-06 | -1.54E+01 | 0 | 625 | 8.04% | 2511 | 6.56% | 1.48% | 1.23 |
| KLF5(Zf)/LoVo-KLF5-ChIP-Seq(GSE49402)/Homer | 1.00E-03 | -8.47E+00 | 0.0011 | 990 | 12.73% | 4374.2 | 11.43% | 1.30% | 1.11 |
| NF1(CTF)/LNCAP-NF1-ChIP-Seq(Unpublished)/Homer | 1.00E-03 | -8.42E+00 | 0.0011 | 518 | 6.66% | 2185 | 5.71% | 0.95% | 1.17 |
| Gata4(Zf)/Heart-Gata4-ChIP-Seq(GSE35151)/Homer | 1.00E-03 | -8.31E+00 | 0.0012 | 1391 | 17.88% | 6275.2 | 16.40% | 1.48% | 1.09 |
| Gfi1b(Zf)/HPC7-Gfi1b-ChIP-Seq(GSE22178)/Homer | 1.00E-03 | -8.31E+00 | 0.0012 | 811 | 10.43% | 3542.6 | 9.26% | 1.17% | 1.13 |
| AMYB(HTH)/Testes-AMYB-ChIP-Seq(GSE44588)/Homer | 1.00E-03 | -8.27E+00 | 0.0012 | 1817 | 23.36% | 8310.6 | 21.72% | 1.64% | 1.08 |
| Tbx20(T-box)/Heart-Tbx20-ChIP-Seq(GSE29636)/Homer | 1.00E-02 | -6.61E+00 | 0.0057 | 247 | 3.18% | 999 | 2.61% | 0.57% | 1.22 |
| PU.1(ETS)/ThioMac-PU.1-ChIP-Seq(GSE21512)/Homer | 1.00E-07 | -1.79E+01 | 0 | 776 | 9.98% | 3138.2 | 8.20% | 1.78% | 1.22 |
| Mef2c(MADS)/GM12878-Mef2c-ChIP-Seq(GSE32465)/Homer | 1.00E-03 | -7.67E+00 | 0.0021 | 726 | 9.33% | 3167.2 | 8.28% | 1.05% | 1.13 |
| RXR(NR/DR1)/3T3L1-RXR-ChIP-Seq(GSE13511)/Homer | 1.00E-09 | -2.15E+01 | 0 | 954 | 12.27% | 3868.1 | 10.11% | 2.16% | 1.21 |
| AR-halfsite(NR)/LNCaP-AR-ChIP-Seq(GSE27824)/Homer | 1.00E-03 | -7.35E+00 | 0.0028 | 3428 | 44.07% | 16171.9 | 42.26% | 1.81% | 1.04 |
| TRa(NR)/C17.2-TRa-ChIP-Seq(GSE38347)/Homer | 1.00E-03 | -7.67E+00 | 0.0021 | 312 | 4.01% | 1268.6 | 3.32% | 0.69% | 1.21 |
| Elk1(ETS)/Hela-Elk1-ChIP-Seq(GSE31477)/Homer | 1.00E-02 | -6.58E+00 | 0.0058 | 650 | 8.36% | 2849.4 | 7.45% | 0.91% | 1.12 |
| OCT4-SOX2-TCF-NANOG((POU/Homeobox/HMG)/mES-Oct4-ChIP-Seq(GSE11431)/Homer | 1.00E-02 | -6.54E+00 | 0.0059 | 325 | 4.18% | 1353 | 3.54% | 0.64% | 1.18 |
| Gata2(Zf)/K562-GATA2-ChIP-Seq(GSE18829)/Homer | 1.00E-02 | -6.49E+00 | 0.0062 | 868 | 11.16% | 3875.6 | 10.13% | 1.03% | 1.10 |
| GRHL2(CP2)/HBE-GRHL2-ChIP-Seq(GSE46194)/Homer | 1.00E-07 | -1.70E+01 | 0 | 815 | 10.48% | 3334.1 | 8.71% | 1.77% | 1.20 |
| Nr5a2(NR)/mES-Nr5a2-ChIP-Seq(GSE19019)/Homer | 1.00E-02 | -6.25E+00 | 0.0076 | 380 | 4.89% | 1610.1 | 4.21% | 0.68% | 1.16 |
| PU.1-IRF(ETS:IRF)/Bcell-PU.1-ChIP-Seq(GSE21512)/Homer | 1.00E-02 | -6.20E+00 | 0.0078 | 1675 | 21.54% | 7734.8 | 20.21% | 1.33% | 1.07 |
| HNF6(Homeobox)/Liver-Hnf6-ChIP-Seq(ERP000394)/Homer | 1.00E-06 | -1.47E+01 | 0 | 703 | 9.04% | 2877.6 | 7.52% | 1.52% | 1.20 |
| Znf263(Zf)/K562-Znf263-ChIP-Seq(GSE31477)/Homer | 1.00E-02 | -6.07E+00 | 0.0086 | 1192 | 15.33% | 5429.4 | 14.19% | 1.14% | 1.08 |
| EBF1(EBF)/Near-E2A-ChIP-Seq(GSE21512)/Homer | 1.00E-02 | -6.07E+00 | 0.0086 | 666 | 8.56% | 2942 | 7.69% | 0.87% | 1.11 |
| Gata1(Zf)/K562-GATA1-ChIP-Seq(GSE18829)/Homer | 1.00E-02 | -5.94E+00 | 0.0096 | 754 | 9.69% | 3360.2 | 8.78% | 0.91% | 1.10 |
| BATF(bZIP)/Th17-BATF-ChIP-Seq(GSE39756)/Homer | 1.00E-02 | -5.87E+00 | 0.0101 | 2733 | 35.14% | 12874.6 | 33.65% | 1.49% | 1.04 |
| BMYB(HTH)/Hela-BMYB-ChIPSeq(GSE27030)/Homer | 1.00E-02 | -5.81E+00 | 0.0105 | 1869 | 24.03% | 8691 | 22.71% | 1.32% | 1.06 |
| AP-1(bZIP)/ThioMac-PU.1-ChIP-Seq(GSE21512)/Homer | 1.00E-02 | -5.77E+00 | 0.0108 | 2845 | 36.58% | 13426.7 | 35.09% | 1.49% | 1.04 |
| Maz(Zf)/HepG2-Maz-ChIP-Seq(GSE31477)/Homer | 1.00E-02 | -5.73E+00 | 0.0111 | 664 | 8.54% | 2945.8 | 7.70% | 0.84% | 1.11 |
| Atoh1(bHLH)/Cerebellum-Atoh1-ChIP-Seq(GSE22111)/Homer | 1.00E-02 | -5.49E+00 | 0.014 | 952 | 12.24% | 4315.2 | 11.28% | 0.96% | 1.09 |
| HNF4a(NR/DR1)/HepG2-HNF4a-ChIP-Seq(GSE25021)/Homer | 1.00E-02 | -5.44E+00 | 0.0144 | 402 | 5.17% | 1734.4 | 4.53% | 0.64% | 1.14 |
| NFY(CCAAT)/Promoter/Homer | 1.00E-02 | -5.36E+00 | 0.0155 | 847 | 10.89% | 3824 | 9.99% | 0.90% | 1.09 |
| Rfx5(HTH)/GM12878-Rfx5-ChIP-Seq(GSE31477)/Homer | 1.00E-02 | -5.21E+00 | 0.0177 | 345 | 4.44% | 1478.9 | 3.86% | 0.58% | 1.15 |
| Nanog(Homeobox)/mES-Nanog-ChIP-Seq(GSE11724)/Homer | 1.00E-02 | -5.20E+00 | 0.0177 | 4714 | 60.61% | 22648.4 | 59.19% | 1.42% | 1.02 |
| Nkx2.1(Homeobox)/LungAC-Nkx2.1-ChIP-Seq(GSE43252)/Homer | 1.00E-02 | -5.15E+00 | 0.0183 | 2738 | 35.20% | 12948.4 | 33.84% | 1.36% | 1.04 |
| Bach2(bZIP)/OCILy7-Bach2-ChIP-Seq(GSE44420)/Homer | 1.00E-02 | -5.14E+00 | 0.0183 | 823 | 10.58% | 3719.5 | 9.72% | 0.86% | 1.09 |
| RUNX-AML(Runt)/CD4+-PolII-ChIP-Seq(Barski et al.)/Homer | 1.00E-02 | -5.06E+00 | 0.0195 | 964 | 12.39% | 4392.2 | 11.48% | 0.91% | 1.08 |
| Nkx3.1(Homeobox)/LNCaP-Nkx3.1-ChIP-Seq(GSE28264)/Homer | 1.00E-02 | -5.06E+00 | 0.0195 | 2545 | 32.72% | 12015.7 | 31.40% | 1.32% | 1.04 |
| Myf5(bHLH)/GM-Myf5-ChIP-Seq(GSE24852)/Homer | 1.00E-02 | -5.01E+00 | 0.02 | 594 | 7.64% | 2644.4 | 6.91% | 0.73% | 1.11 |
| Pitx1(Homeobox)/Chicken-Pitx1-ChIP-Seq(GSE38910)/Homer | 1.00E-02 | -4.86E+00 | 0.0229 | 4838 | 62.20% | 23287.3 | 60.86% | 1.34% | 1.02 |
| Bcl6(Zf)/Liver-Bcl6-ChIP-Seq(GSE31578)/Homer | 1.00E-02 | -4.62E+00 | 0.0289 | 1685 | 21.66% | 7876.7 | 20.58% | 1.08% | 1.05 |
