# Supplementary material for: Heterarchy of transcription factors driving basal and luminal cell phenotypes in human urothelium
Source: Cell Death Differ. 2017 Mar 10;24(5):809–18. doi: 10.1038/cdd.2017.10 (PMC5423105; doi:10.1038/cdd.2017.10)
Supplement: Supplementary Tables 17-20 [file cdd201710x10.docx]

Supplementary Table 17. Motifs enriched in all FAIRE peaks containing P63 unique to control cells at 24 h.

| P63 Co-occuring Motifs 24 h Control | P-value | log P-pvalue | q-value (Benjamini) | # Target Sequences with Motif | % of Targets Sequences with Motif | # Background Sequences with Motif | % of Background Sequences with Motif | % Target > Background | Ratio Enrichment : Background |
| --- | --- | --- | --- | --- | --- | --- | --- | --- | --- |
| p63(p53)/Keratinocyte-p63-ChIP-Seq(GSE17611)/Homer | 1e-3305 | -7.61E+03 | 0 | 3085 | 99.97% | 2755.1 | 8.46% | 91.51% | 11.82 |
| p53(p53)/Saos-p53-ChIP-Seq(GSE15780)/Homer | 1e-857 | -1.97E+03 | 0 | 1247 | 40.41% | 1345.1 | 4.13% | 36.28% | 9.78 |
| p53(p53)/Saos-p53-ChIP-Seq/Homer | 1e-857 | -1.97E+03 | 0 | 1247 | 40.41% | 1345.1 | 4.13% | 36.28% | 9.78 |
| Tcfcp2l1(CP2)/mES-Tcfcp2l1-ChIP-Seq(GSE11431)/Homer | 1.00E-210 | -4.84E+02 | 0 | 435 | 14.10% | 687.5 | 2.11% | 11.99% | 6.68 |
| p53(p53)/mES-cMyc-ChIP-Seq(GSE11431)/Homer | 1.00E-31 | -7.34E+01 | 0 | 93 | 3.01% | 212.8 | 0.65% | 2.36% | 4.63 |
| ZFX(Zf)/mES-Zfx-ChIP-Seq(GSE11431)/Homer | 1.00E-17 | -3.94E+01 | 0 | 623 | 20.19% | 4724.9 | 14.51% | 5.68% | 1.39 |
| BMAL1(HLH)/Liver-Bmal1-ChIP-Seq(GSE39860)/Homer | 1.00E-11 | -2.60E+01 | 0 | 867 | 28.09% | 7428.4 | 22.82% | 5.27% | 1.23 |
| ZNF711(Zf)/SH-SY5Y-ZNF711-ChIP-Seq(GSE20673)/Homer | 1.00E-10 | -2.38E+01 | 0 | 733 | 23.75% | 6195 | 19.03% | 4.72% | 1.25 |
| Smad3(MAD)/NPC-Smad3-ChIP-Seq(GSE36673)/Homer | 1.00E-08 | -2.00E+01 | 0 | 1297 | 42.03% | 11999.2 | 36.86% | 5.17% | 1.14 |
| Smad2(MAD)/ES-SMAD2-ChIP-Seq(GSE29422)/Homer | 1.00E-07 | -1.63E+01 | 0 | 692 | 22.42% | 6072.9 | 18.66% | 3.76% | 1.20 |
| Smad4(MAD)/ESC-SMAD4-ChIP-Seq(GSE29422)/Homer | 1.00E-07 | -1.62E+01 | 0 | 698 | 22.62% | 6137.5 | 18.85% | 3.77% | 1.20 |
| AR-halfsite(NR)/LNCaP-AR-ChIP-Seq(GSE27824)/Homer | 1.00E-05 | -1.31E+01 | 0 | 1539 | 49.87% | 14879.5 | 45.71% | 4.16% | 1.09 |
| STAT6/Macrophage-Stat6-ChIP-Seq(GSE38377)/Homer | 1.00E-04 | -1.05E+01 | 0.0005 | 318 | 10.30% | 2678.1 | 8.23% | 2.07% | 1.25 |
| ZNF143\|STAF(Zf)/CUTLL-ZNF143-ChIP-Seq(GSE29600)/Homer | 1.00E-04 | -1.05E+01 | 0.0005 | 150 | 4.86% | 1123.1 | 3.45% | 1.41% | 1.41 |
| HOXD13(Homeobox)/Chicken-Hoxd13-ChIP-Seq(GSE38910)/Homer | 1.00E-04 | -1.03E+01 | 0.0006 | 611 | 19.80% | 5545.4 | 17.04% | 2.76% | 1.16 |
| Pitx1(Homeobox)/Chicken-Pitx1-ChIP-Seq(GSE38910)/Homer | 1.00E-04 | -1.01E+01 | 0.0007 | 1856 | 60.14% | 18435.9 | 56.63% | 3.51% | 1.06 |
| STAT6(Stat)/CD4-Stat6-ChIP-Seq(GSE22104)/Homer | 1.00E-03 | -9.20E+00 | 0.0015 | 317 | 10.27% | 2718.3 | 8.35% | 1.92% | 1.23 |
| ETV1(ETS)/GIST48-ETV1-ChIP-Seq(GSE22441)/Homer | 1.00E-03 | -7.82E+00 | 0.0055 | 824 | 26.70% | 7838.8 | 24.08% | 2.62% | 1.11 |
| Pax8(Paired/Homeobox)/Thyroid-Pax8-ChIP-Seq(GSE26938)/Homer | 1.00E-03 | -7.78E+00 | 0.0055 | 166 | 5.38% | 1341.4 | 4.12% | 1.26% | 1.31 |
| MafA(bZIP)/Islet-MafA-ChIP-Seq(GSE30298)/Homer | 1.00E-03 | -7.69E+00 | 0.0056 | 518 | 16.79% | 4760.9 | 14.63% | 2.16% | 1.15 |
| NPAS2(HLH)/Liver-NPAS2-ChIP-Seq(GSE39860)/Homer | 1.00E-03 | -7.67E+00 | 0.0056 | 452 | 14.65% | 4106.1 | 12.61% | 2.04% | 1.16 |
| Egr2/Thymocytes-Egr2-ChIP-Seq(GSE34254)/Homer | 1.00E-03 | -7.60E+00 | 0.0056 | 72 | 2.33% | 501.5 | 1.54% | 0.79% | 1.51 |
| Pdx1(Homeobox)/Islet-Pdx1-ChIP-Seq(SRA008281)/Homer | 1.00E-03 | -7.49E+00 | 0.006 | 641 | 20.77% | 6005.8 | 18.45% | 2.32% | 1.13 |
| TRa(NR)/C17.2-TRa-ChIP-Seq(GSE38347)/Homer | 1.00E-03 | -7.35E+00 | 0.0066 | 171 | 5.54% | 1401.8 | 4.31% | 1.23% | 1.29 |
| PR(NR)/T47D-PR-ChIP-Seq(GSE31130)/Homer | 1.00E-03 | -7.04E+00 | 0.0086 | 996 | 32.27% | 9657.6 | 29.67% | 2.60% | 1.09 |
| Arnt:Ahr(bHLH)/MCF7-Arnt-ChIP-Seq(Lo et al.)/Homer | 1.00E-02 | -6.76E+00 | 0.011 | 268 | 8.68% | 2348.7 | 7.22% | 1.46% | 1.20 |
| Ets1-distal(ETS)/CD4+-PolII-ChIP-Seq(Barski et al.)/Homer | 1.00E-02 | -6.16E+00 | 0.0193 | 242 | 7.84% | 2123.9 | 6.52% | 1.32% | 1.20 |
| ETS1(ETS)/Jurkat-ETS1-ChIP-Seq(GSE17954)/Homer | 1.00E-02 | -5.81E+00 | 0.0263 | 661 | 21.42% | 6323.7 | 19.43% | 1.99% | 1.10 |
| Elk4(ETS)/Hela-Elk4-ChIP-Seq(GSE31477)/Homer | 1.00E-02 | -5.73E+00 | 0.0275 | 308 | 9.98% | 2788.4 | 8.57% | 1.41% | 1.16 |
| Max(HLH)/K562-Max-ChIP-Seq(GSE31477)/Homer | 1.00E-02 | -5.68E+00 | 0.028 | 261 | 8.46% | 2330.4 | 7.16% | 1.30% | 1.18 |
| GABPA(ETS)/Jurkat-GABPa-ChIP-Seq(GSE17954)/Homer | 1.00E-02 | -5.59E+00 | 0.0296 | 558 | 18.08% | 5295.9 | 16.27% | 1.81% | 1.11 |
| Egr1(Zf)/K562-Egr1-ChIP-Seq(GSE32465)/Homer | 1.00E-02 | -5.58E+00 | 0.0296 | 214 | 6.93% | 1878.7 | 5.77% | 1.16% | 1.20 |
| GATA3(Zf)/iTreg-Gata3-ChIP-Seq(GSE20898)/Homer | 1.00E-02 | -5.50E+00 | 0.0306 | 739 | 23.95% | 7143.4 | 21.94% | 2.01% | 1.09 |
| Cdx2(Homeobox)/mES-Cdx2-ChIP-Seq(GSE14586)/Homer | 1.00E-02 | -5.08E+00 | 0.045 | 401 | 12.99% | 3750.3 | 11.52% | 1.47% | 1.13 |
| Tbet(T-box)/CD8-Tbet-ChIP-Seq(GSE33802)/Homer | 1.00E-02 | -5.06E+00 | 0.045 | 515 | 16.69% | 4899.8 | 15.05% | 1.64% | 1.11 |
| Elk1(ETS)/Hela-Elk1-ChIP-Seq(GSE31477)/Homer | 1.00E-02 | -4.97E+00 | 0.0477 | 314 | 10.17% | 2890.6 | 8.88% | 1.29% | 1.15 |
| EWS:FLI1-fusion(ETS)/SK_N_MC-EWS:FLI1-ChIP-Seq(SRA014231)/Homer | 1.00E-02 | -4.85E+00 | 0.052 | 392 | 12.70% | 3675.5 | 11.29% | 1.41% | 1.12 |
| Fli1(ETS)/CD8-FLI-ChIP-Seq(GSE20898)/Homer | 1.00E-02 | -4.85E+00 | 0.052 | 663 | 21.48% | 6420.2 | 19.72% | 1.76% | 1.09 |
| ERG(ETS)/VCaP-ERG-ChIP-Seq(GSE14097)/Homer | 1.00E-02 | -4.83E+00 | 0.052 | 951 | 30.82% | 9383.6 | 28.83% | 1.99% | 1.07 |
| TEAD4(TEA)/Tropoblast-Tead4-ChIP-Seq(GSE37350)/Homer | 1.00E-02 | -4.81E+00 | 0.052 | 514 | 16.66% | 4907.3 | 15.08% | 1.58% | 1.10 |
| EWS:ERG-fusion(ETS)/CADO_ES1-EWS:ERG-ChIP-Seq(SRA014231)/Homer | 1.00E-02 | -4.77E+00 | 0.052 | 537 | 17.40% | 5143.5 | 15.80% | 1.60% | 1.10 |

Supplementary Table 18. Motifs enriched in all FAIRE peaks containing P63 unique to differentiated cells at 24 h.

| P63 Co-occuring Motifs 24 h Differentiated | P-value | log P-pvalue | q-value (Benjamini) | # Target Sequences with Motif | % of Targets Sequences with Motif | # Background Sequences with Motif | % of Background Sequences with Motif | % Target > Background | Ratio Enrichment : Background |
| --- | --- | --- | --- | --- | --- | --- | --- | --- | --- |
| p63(p53)/Keratinocyte-p63-ChIP-Seq(GSE17611)/Homer | 1e-4376 | -1.01E+04 | 0 | 3775 | 99.95% | 1882.1 | 6.90% | 93.05% | 14.49 |
| p53(p53)/Saos-p53-ChIP-Seq(GSE15780)/Homer | 1e-1204 | -2.77E+03 | 0 | 1548 | 40.98% | 919.3 | 3.37% | 37.61% | 12.16 |
| p53(p53)/Saos-p53-ChIP-Seq/Homer | 1e-1204 | -2.77E+03 | 0 | 1548 | 40.98% | 919.3 | 3.37% | 37.61% | 12.16 |
| Tcfcp2l1(CP2)/mES-Tcfcp2l1-ChIP-Seq(GSE11431)/Homer | 1.00E-261 | -6.01E+02 | 0 | 545 | 14.43% | 595.7 | 2.18% | 12.25% | 6.62 |
| p53(p53)/mES-cMyc-ChIP-Seq(GSE11431)/Homer | 1.00E-53 | -1.24E+02 | 0 | 130 | 3.44% | 165.3 | 0.61% | 2.83% | 5.64 |
| GRHL2(CP2)/HBE-GRHL2-ChIP-Seq(GSE46194)/Homer | 1.00E-20 | -4.63E+01 | 0 | 415 | 10.99% | 1868.4 | 6.85% | 4.14% | 1.60 |
| TRa(NR)/C17.2-TRa-ChIP-Seq(GSE38347)/Homer | 1.00E-04 | -1.06E+01 | 0.0004 | 225 | 5.96% | 1232.1 | 4.52% | 1.44% | 1.32 |
| GATA-IR3(Zf)/iTreg-Gata3-ChIP-Seq(GSE20898)/Homer | 1.00E-02 | -5.73E+00 | 0.0257 | 105 | 2.78% | 574.7 | 2.11% | 0.67% | 1.32 |
| ZFX(Zf)/mES-Zfx-ChIP-Seq(GSE11431)/Homer | 1.00E-12 | -2.91E+01 | 0 | 816 | 21.60% | 4642.6 | 17.02% | 4.58% | 1.27 |
| FOXP1(Forkhead)/H9-FOXP1-ChIP-Seq(GSE31006)/Homer | 1.00E-04 | -9.49E+00 | 0.001 | 330 | 8.74% | 1934.6 | 7.09% | 1.65% | 1.23 |
| ZNF711(Zf)/SH-SY5Y-ZNF711-ChIP-Seq(GSE20673)/Homer | 1.00E-11 | -2.65E+01 | 0 | 1002 | 26.53% | 5941.7 | 21.79% | 4.74% | 1.22 |
| CEBP(bZIP)/CEBPb-ChIP-Seq(GSE21512)/Homer | 1.00E-05 | -1.34E+01 | 0 | 611 | 16.18% | 3681.2 | 13.50% | 2.68% | 1.20 |
| NF1-halfsite(CTF)/LNCaP-NF1-ChIP-Seq(Unpublished)/Homer | 1.00E-11 | -2.74E+01 | 0 | 1221 | 32.33% | 7406.2 | 27.16% | 5.17% | 1.19 |
| Arnt:Ahr(bHLH)/MCF7-Arnt-ChIP-Seq(Lo et al.)/Homer | 1.00E-03 | -7.46E+00 | 0.0062 | 345 | 9.13% | 2094.2 | 7.68% | 1.45% | 1.19 |
| BMAL1(HLH)/Liver-Bmal1-ChIP-Seq(GSE39860)/Homer | 1.00E-08 | -2.03E+01 | 0 | 1029 | 27.24% | 6296.1 | 23.09% | 4.15% | 1.18 |
| Gata1(Zf)/K562-GATA1-ChIP-Seq(GSE18829)/Homer | 1.00E-02 | -6.53E+00 | 0.0124 | 333 | 8.82% | 2045.2 | 7.50% | 1.32% | 1.18 |
| Smad4(MAD)/ESC-SMAD4-ChIP-Seq(GSE29422)/Homer | 1.00E-06 | -1.60E+01 | 0 | 910 | 24.09% | 5620.5 | 20.61% | 3.48% | 1.17 |
| Gata2(Zf)/K562-GATA2-ChIP-Seq(GSE18829)/Homer | 1.00E-02 | -6.73E+00 | 0.0109 | 372 | 9.85% | 2300.1 | 8.43% | 1.42% | 1.17 |
| Fox:Ebox(Forkhead:HLH)/Panc1-Foxa2-ChIP-Seq(GSE47459)/Homer | 1.00E-04 | -9.75E+00 | 0.0008 | 713 | 18.88% | 4499.1 | 16.50% | 2.38% | 1.14 |
| E2A-nearPU.1(HLH)/Bcell-PU.1-ChIP-Seq(GSE21512)/Homer | 1.00E-03 | -9.09E+00 | 0.0014 | 724 | 19.17% | 4601.5 | 16.87% | 2.30% | 1.14 |
| Gata4(Zf)/Heart-Gata4-ChIP-Seq(GSE35151)/Homer | 1.00E-03 | -7.30E+00 | 0.0069 | 573 | 15.17% | 3642.8 | 13.36% | 1.81% | 1.14 |
| Foxa2(Forkhead)/Liver-Foxa2-ChIP-Seq(GSE25694)/Homer | 1.00E-03 | -7.04E+00 | 0.0083 | 594 | 15.73% | 3797.6 | 13.93% | 1.80% | 1.13 |
| GATA3(Zf)/iTreg-Gata3-ChIP-Seq(GSE20898)/Homer | 1.00E-04 | -9.35E+00 | 0.0011 | 849 | 22.48% | 5451.4 | 19.99% | 2.49% | 1.12 |
| AR-halfsite(NR)/LNCaP-AR-ChIP-Seq(GSE27824)/Homer | 1.00E-12 | -2.77E+01 | 0 | 1985 | 52.55% | 12766.1 | 46.81% | 5.74% | 1.12 |
| E2A(HLH)/proBcell-E2A-ChIP-Seq(GSE21978)/Homer | 1.00E-03 | -7.24E+00 | 0.007 | 738 | 19.54% | 4780.6 | 17.53% | 2.01% | 1.11 |
| FOXA1(Forkhead)/LNCAP-FOXA1-ChIP-Seq(GSE27824)/Homer | 1.00E-03 | -7.92E+00 | 0.0043 | 845 | 22.37% | 5489.4 | 20.13% | 2.24% | 1.11 |
| FOXA1(Forkhead)/MCF7-FOXA1-ChIP-Seq(GSE26831)/Homer | 1.00E-02 | -6.59E+00 | 0.0121 | 702 | 18.59% | 4563.4 | 16.73% | 1.86% | 1.11 |
| PR(NR)/T47D-PR-ChIP-Seq(GSE31130)/Homer | 1.00E-05 | -1.17E+01 | 0.0001 | 1224 | 32.41% | 7957.2 | 29.18% | 3.23% | 1.11 |
| Smad3(MAD)/NPC-Smad3-ChIP-Seq(GSE36673)/Homer | 1.00E-07 | -1.70E+01 | 0 | 1644 | 43.53% | 10698 | 39.23% | 4.30% | 1.11 |
| CEBP:AP1(bZIP)/ThioMac-CEBPb-ChIP-Seq(GSE21512)/Homer | 1.00E-02 | -5.58E+00 | 0.0286 | 600 | 15.89% | 3908.7 | 14.33% | 1.56% | 1.11 |
| Smad2(MAD)/ES-SMAD2-ChIP-Seq(GSE29422)/Homer | 1.00E-03 | -7.77E+00 | 0.0047 | 877 | 23.22% | 5719.1 | 20.97% | 2.25% | 1.11 |
| PU.1-IRF(ETS:IRF)/Bcell-PU.1-ChIP-Seq(GSE21512)/Homer | 1.00E-02 | -5.60E+00 | 0.0286 | 840 | 22.24% | 5578 | 20.45% | 1.79% | 1.09 |
| CRX(Homeobox)/Retina-Crx-ChIP-Seq(GSE20012)/Homer | 1.00E-02 | -6.17E+00 | 0.0172 | 1233 | 32.64% | 8311.1 | 30.48% | 2.16% | 1.07 |

Supplementary Table 19. Motifs enriched in all FAIRE peaks containing P63 unique to control cells at 144 h.

| P63 Co-occuring Motifs 144 h Control | P-value | log P-pvalue | q-value (Benjamini) | # Target Sequences with Motif | % of Targets Sequences with Motif | # Background Sequences with Motif | % of Background Sequences with Motif | % Target > Background | Ratio Enrichment : Background |
| --- | --- | --- | --- | --- | --- | --- | --- | --- | --- |
| p63(p53)/Keratinocyte-p63-ChIP-Seq(GSE17611)/Homer | 1e-6076 | -1.40E+04 | 0 | 4453 | 99.93% | 1323.9 | 4.30% | 95.63% | 23.24 |
| p53(p53)/Saos-p53-ChIP-Seq(GSE15780)/Homer | 1e-1883 | -4.34E+03 | 0 | 1863 | 41.81% | 609.7 | 1.98% | 39.83% | 21.12 |
| p53(p53)/Saos-p53-ChIP-Seq/Homer | 1e-1883 | -4.34E+03 | 0 | 1863 | 41.81% | 609.7 | 1.98% | 39.83% | 21.12 |
| Tcfcp2l1(CP2)/mES-Tcfcp2l1-ChIP-Seq(GSE11431)/Homer | 0.00E+00 | -7.10E+02 | 0 | 595 | 13.35% | 557.1 | 1.81% | 11.54% | 7.38 |
| p53(p53)/mES-cMyc-ChIP-Seq(GSE11431)/Homer | 1.00E-57 | -1.32E+02 | 0 | 129 | 2.89% | 143.5 | 0.47% | 2.42% | 6.15 |

Supplementary Table 20. Motifs enriched in all FAIRE peaks containing P63 unique to differentiated cells at 144 h.

| P63 Co-occuring Motifs 144 h Differentiated | P-value | log P-pvalue | q-value (Benjamini) | # Target Sequences with Motif | % of Targets Sequences with Motif | # Background Sequences with Motif | % of Background Sequences with Motif | % Target > Background | Ratio Enrichment : Background |
| --- | --- | --- | --- | --- | --- | --- | --- | --- | --- |
| p63(p53)/Keratinocyte-p63-ChIP-Seq(GSE17611)/Homer | 1e-2649 | -6.10E+03 | 0 | 2556 | 99.96% | 3608.8 | 9.16% | 90.80% | 10.91 |
| p53(p53)/Saos-p53-ChIP-Seq(GSE15780)/Homer | 1e-560 | -1.29E+03 | 0 | 935 | 36.57% | 1789 | 4.54% | 32.03% | 8.06 |
| p53(p53)/Saos-p53-ChIP-Seq/Homer | 1e-560 | -1.29E+03 | 0 | 935 | 36.57% | 1789 | 4.54% | 32.03% | 8.06 |
| Tcfcp2l1(CP2)/mES-Tcfcp2l1-ChIP-Seq(GSE11431)/Homer | 1.00E-169 | -3.91E+02 | 0 | 374 | 14.63% | 936.2 | 2.38% | 12.25% | 6.15 |
| p53(p53)/mES-cMyc-ChIP-Seq(GSE11431)/Homer | 1.00E-26 | -6.17E+01 | 0 | 80 | 3.13% | 274.8 | 0.70% | 2.43% | 4.47 |
| ZNF711(Zf)/SH-SY5Y-ZNF711-ChIP-Seq(GSE20673)/Homer | 1.00E-09 | -2.08E+01 | 0 | 656 | 25.66% | 8148.5 | 20.69% | 4.97% | 1.24 |
| ZFX(Zf)/mES-Zfx-ChIP-Seq(GSE11431)/Homer | 1.00E-08 | -2.04E+01 | 0 | 523 | 20.45% | 6293.6 | 15.98% | 4.47% | 1.28 |
| BMAL1(HLH)/Liver-Bmal1-ChIP-Seq(GSE39860)/Homer | 1.00E-07 | -1.61E+01 | 0 | 715 | 27.96% | 9253.2 | 23.50% | 4.46% | 1.19 |
| GRHL2(CP2)/HBE-GRHL2-ChIP-Seq(GSE46194)/Homer | 1.00E-06 | -1.46E+01 | 0 | 305 | 11.93% | 3548.9 | 9.01% | 2.92% | 1.32 |
| AR-halfsite(NR)/LNCaP-AR-ChIP-Seq(GSE27824)/Homer | 1.00E-06 | -1.45E+01 | 0 | 1311 | 51.27% | 18279.4 | 46.42% | 4.85% | 1.10 |
| TRa(NR)/C17.2-TRa-ChIP-Seq(GSE38347)/Homer | 1.00E-05 | -1.38E+01 | 0 | 163 | 6.37% | 1703.2 | 4.33% | 2.04% | 1.47 |
| GATA3(Zf)/iTreg-Gata3-ChIP-Seq(GSE20898)/Homer | 1.00E-05 | -1.33E+01 | 0 | 631 | 24.68% | 8209.7 | 20.85% | 3.83% | 1.18 |
| Gata4(Zf)/Heart-Gata4-ChIP-Seq(GSE35151)/Homer | 1.00E-05 | -1.18E+01 | 0.0001 | 430 | 16.82% | 5422.2 | 13.77% | 3.05% | 1.22 |
| Fox:Ebox(Forkhead:HLH)/Panc1-Foxa2-ChIP-Seq(GSE47459)/Homer | 1.00E-05 | -1.17E+01 | 0.0002 | 522 | 20.41% | 6743.8 | 17.13% | 3.28% | 1.19 |
| Foxa2(Forkhead)/Liver-Foxa2-ChIP-Seq(GSE25694)/Homer | 1.00E-04 | -1.11E+01 | 0.0003 | 448 | 17.52% | 5721.7 | 14.53% | 2.99% | 1.21 |
| Gata1(Zf)/K562-GATA1-ChIP-Seq(GSE18829)/Homer | 1.00E-04 | -9.74E+00 | 0.0009 | 246 | 9.62% | 2961.8 | 7.52% | 2.10% | 1.28 |
| EHF(ETS)/LoVo-EHF-ChIP-Seq(GSE49402)/Homer | 1.00E-04 | -9.72E+00 | 0.0009 | 724 | 28.31% | 9829.4 | 24.96% | 3.35% | 1.13 |
| Gata2(Zf)/K562-GATA2-ChIP-Seq(GSE18829)/Homer | 1.00E-04 | -9.53E+00 | 0.001 | 276 | 10.79% | 3386.1 | 8.60% | 2.19% | 1.25 |
| ELF5(ETS)/T47D-ELF5-ChIP-Seq(GSE30407)/Homer | 1.00E-04 | -9.27E+00 | 0.0012 | 450 | 17.60% | 5865.7 | 14.90% | 2.70% | 1.18 |
| Arnt:Ahr(bHLH)/MCF7-Arnt-ChIP-Seq(Lo et al.)/Homer | 1.00E-03 | -9.04E+00 | 0.0015 | 245 | 9.58% | 2982.5 | 7.57% | 2.01% | 1.27 |
| NF1-halfsite(CTF)/LNCaP-NF1-ChIP-Seq(Unpublished)/Homer | 1.00E-03 | -9.01E+00 | 0.0015 | 800 | 31.29% | 11017.2 | 27.98% | 3.31% | 1.12 |
| FOXA1(Forkhead)/LNCAP-FOXA1-ChIP-Seq(GSE27824)/Homer | 1.00E-03 | -8.45E+00 | 0.0024 | 626 | 24.48% | 8489.9 | 21.56% | 2.92% | 1.14 |
| ETV1(ETS)/GIST48-ETV1-ChIP-Seq(GSE22441)/Homer | 1.00E-03 | -8.05E+00 | 0.0034 | 705 | 27.57% | 9691.2 | 24.61% | 2.96% | 1.12 |
| PU.1-IRF(ETS:IRF)/Bcell-PU.1-ChIP-Seq(GSE21512)/Homer | 1.00E-03 | -7.76E+00 | 0.0044 | 580 | 22.68% | 7871.7 | 19.99% | 2.69% | 1.13 |
| PR(NR)/T47D-PR-ChIP-Seq(GSE31130)/Homer | 1.00E-03 | -7.43E+00 | 0.0059 | 830 | 32.46% | 11614.5 | 29.49% | 2.97% | 1.10 |
| Smad4(MAD)/ESC-SMAD4-ChIP-Seq(GSE29422)/Homer | 1.00E-03 | -7.36E+00 | 0.006 | 575 | 22.49% | 7832.2 | 19.89% | 2.60% | 1.13 |
| E2A-nearPU.1(HLH)/Bcell-PU.1-ChIP-Seq(GSE21512)/Homer | 1.00E-02 | -6.85E+00 | 0.0096 | 495 | 19.36% | 6702.6 | 17.02% | 2.34% | 1.14 |
| Smad3(MAD)/NPC-Smad3-ChIP-Seq(GSE36673)/Homer | 1.00E-02 | -6.59E+00 | 0.0121 | 1068 | 41.77% | 15299.2 | 38.85% | 2.92% | 1.08 |
| GFY-Staf/Promoters/Homer | 1.00E-02 | -6.52E+00 | 0.0125 | 45 | 1.76% | 427.4 | 1.09% | 0.67% | 1.61 |
| FOXA1(Forkhead)/MCF7-FOXA1-ChIP-Seq(GSE26831)/Homer | 1.00E-02 | -6.06E+00 | 0.0192 | 515 | 20.14% | 7067.4 | 17.95% | 2.19% | 1.12 |
| FOXP1(Forkhead)/H9-FOXP1-ChIP-Seq(GSE31006)/Homer | 1.00E-02 | -5.95E+00 | 0.0208 | 243 | 9.50% | 3132.2 | 7.95% | 1.55% | 1.19 |
| Nr5a2(NR)/mES-Nr5a2-ChIP-Seq(GSE19019)/Homer | 1.00E-02 | -5.64E+00 | 0.0272 | 158 | 6.18% | 1956.5 | 4.97% | 1.21% | 1.24 |
| GATA-IR4(Zf)/iTreg-Gata3-ChIP-Seq(GSE20898)/Homer | 1.00E-02 | -5.54E+00 | 0.0291 | 48 | 1.88% | 488.7 | 1.24% | 0.64% | 1.52 |
| ERG(ETS)/VCaP-ERG-ChIP-Seq(GSE14097)/Homer | 1.00E-02 | -4.98E+00 | 0.0496 | 814 | 31.83% | 11647.1 | 29.58% | 2.25% | 1.08 |
| GATA:SCL/Ter119-SCL-ChIP-Seq(GSE18720)/Homer | 1.00E-02 | -4.94E+00 | 0.0504 | 54 | 2.11% | 582.4 | 1.48% | 0.63% | 1.43 |
| Ets1-distal(ETS)/CD4+-PolII-ChIP-Seq(Barski et al.)/Homer | 1.00E-02 | -4.74E+00 | 0.0599 | 197 | 7.70% | 2560.9 | 6.50% | 1.20% | 1.18 |
